# Cycloaddition Reactions of Sulfo-Biginelli Derived Thiadiazines

**DOI:** 10.1021/acs.orglett.6c02685

**Published:** 2026-07-20

**Authors:** Clare L. Hill, Michael Sambor, Angela Yang, Peter Wipf

**Affiliations:** † Department of Chemistry, 6614 University of Pittsburgh, Pittsburgh, Pennsylvania 15260, United States; ‡ Department of Pharmaceutical Sciences, School of Pharmacy, 6614 University of Pittsburgh, Pittsburgh, Pennsylvania 15260, United States

## Abstract

Modified Sulfo-Biginelli conditions, including a new one-pot process from sulfamide and α,β-unsaturated carbonyl compounds, were developed to access diverse 1,2,6-thiadiazine-1,1-dioxides. These electron-deficient heterocyclic building blocks underwent highly regio- and stereoselective [4 + 2] and [3 + 2] cycloadditions with azomethine ylides, alleneoates, and methylene cyclopropanes to provide fused, bridged and spirocyclic thiadiazines. Chiral phosphine catalysis can be used to generate enantiomerically enriched annulation products. This study illustrates the synthetic versatility of cyclic sulfamides and their potential for the discovery of novel heterocyclic scaffolds.

## Graphical abstract

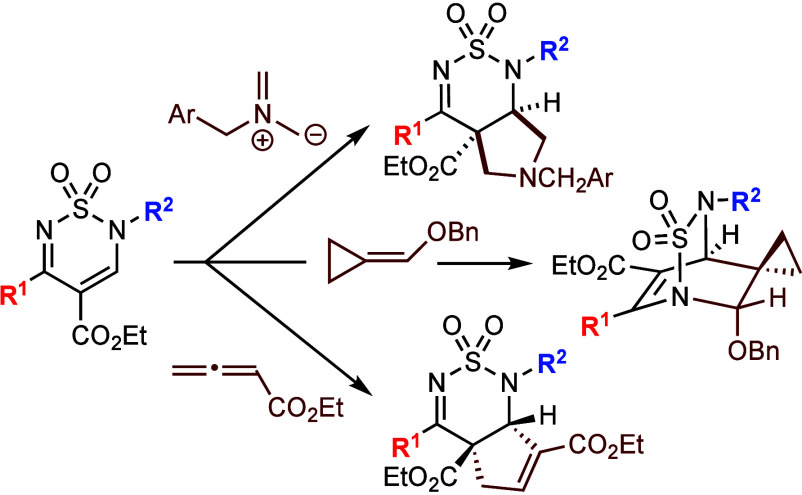

Dihydropyrimidinones (DHPs) have demonstrated a wide range of biological properties, including antimicrobial, anti-inflammatory, and anticancer effects, with additional applications in materials and agrochemical research.^1^ Since DHPs are readily prepared by a Biginelli reaction from aldehydes, β-ketoesters, and ureas, this 3-component condensation has become one of the most popular multicomponent reactions, and new variants continue to be developed.^2,3^ A significant expansion of the scope of the traditional Biginelli reaction has been the use of isosteric replacements of the urea component with guanidines,^4^ thioureas,^5^ selenoureas,^6^ and aminoazoles.^7^ Sulfamides^8,9^ for the preparation of dihydro-1,2,6-thiadiazines (DHTs) have only recently been more broadly investigated in the Sulfo-Biginelli reaction, in part due to their reduced reactivity in the 3-component condensation versus the corresponding ureas.^10–12^ The scope of this reaction has been extended by the use of a dithiatetrazocane precursor,^11,13^ microwave heating,^14^ mechanochemical conditions with sulfonimidamides,^15^ and the addition of TMS-Cl.^12^

DHTs can also be obtained by condensation of sulfamides with monoketones,^9a^ diketones,^9b^ and 3,3-dialkoxypropanoates,^10,11^ or cyclizations of *N*-arylsulfamide imines^16^ and alkynyl sulfamides,^17^ but there has been a notable lack of further derivatization of these heterocycles. We envisioned an opportunity to generate new fused and bridged DHTs by cycloaddition reactions on their oxidized derivatives, 1,2,6-thiadiazine-1,1-dioxides. The use of 1,3-dipoles,^18^ sulfur ylides,^19^ and allenoates^20^ with imines^19,20^ and unsaturated sultams^21^ provided valuable precedence for this plan.

Previously, our group reported a TFA/1,1,1,3,3,3-hexafluoro-2-propanol (HFIP)-mediated preparation of DHTs using dimer **1** for increased reproducibility and yield (Scheme 1, conditions A).^11^ Further investigation into this transformation revealed a new, Cs_2_CO_3_-mediated variant (Scheme 1, conditions B), offering an attractive alternative protocol for the Sulfo-Biginelli reaction by avoiding the use of a strong acid. A regioselective *N*-functionalization^11^ of products **2**–**7** was accomplished by leveraging the difference in p*K*_a_ of the sulfonamide nitrogens (calc. Δp*K*_a_ = 4.5) in a Mitsunobu reaction with di-*tert*-butyl azodicarboxylate (DBAD) or by the corresponding *N*-acylation with Boc_2_O. Dehydrogenation of intermediates **8**–**17** to 1,2,6-thiadiazine-1,1-dioxides **18**–**27** employed a hypervalent iodine oxidation with (diacetoxyiodo)­benzene (PIDA) and *N*-hydroxysuccinimide (NHS),^15,22^ thus completing the synthesis of **18**–**27** in an overall 3 steps from commercially available alkyl, aryl, and heteroaryl aldehydes and **1**. Furthermore, the Cs_2_CO_3_-mediated reaction conditions allowed for the development of a new one-pot synthesis of thiadiazines from alkoxyacrylates or alkoxyacrylonitriles (Scheme 2). Treatment of ethyl 2-benzoyl-3-ethoxyacrylate with sulfamide and cesium carbonate in a solution of acetonitrile followed by heating at reflux for 2 h and subsequent addition of cinnamyl bromide provided thiadiazine **28** in 38% yield after chromatographic purification. Similarly, the nitrile-substituted, *N*(2)-methylated thiadiazine **29** was obtained in 57% yield from 2-benzoyl-3-ethoxyacrylonitrile when the cyclization with sulfamide was conducted in HFIP, followed by addition of methyl iodide and heating in acetonitrile. Combined, the stepwise approach from dimer **1** and the one-pot synthesis from α,β-unsaturated carbonyl compounds provide complementary and versatile access routes to 1,2,6-thiadiazine-1,1-dioxides.

**Scheme 1:**
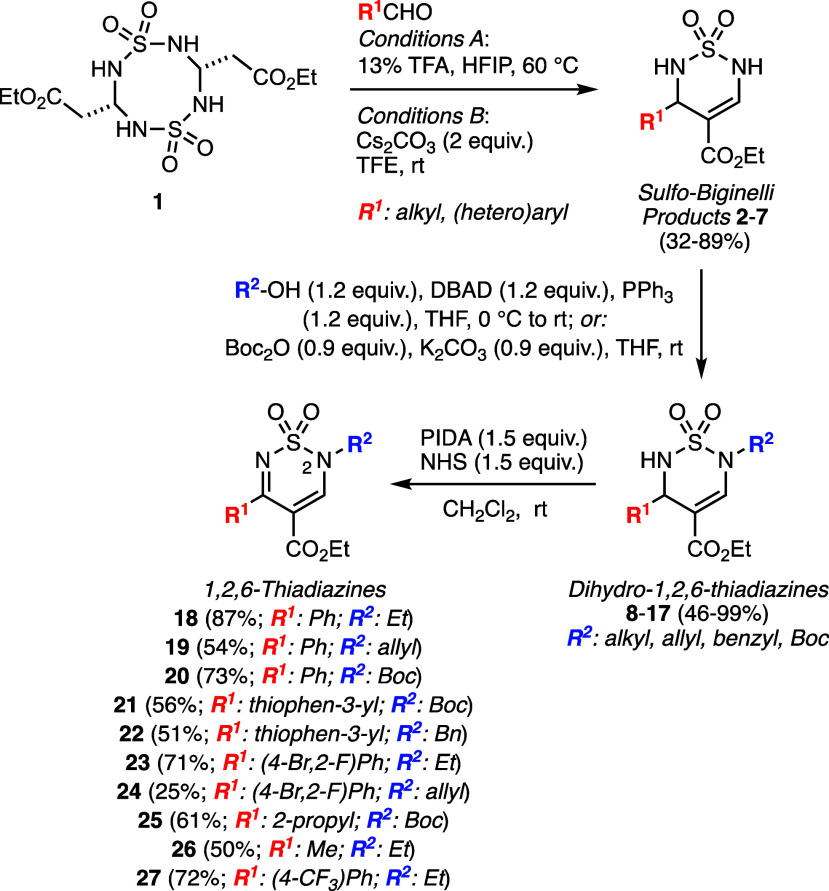
Preparation of Dihydro-1,2,6-thiadiazines **2**–**17** and 1,2,6-Thiadiazines **18**–**29**

**Scheme 2:**
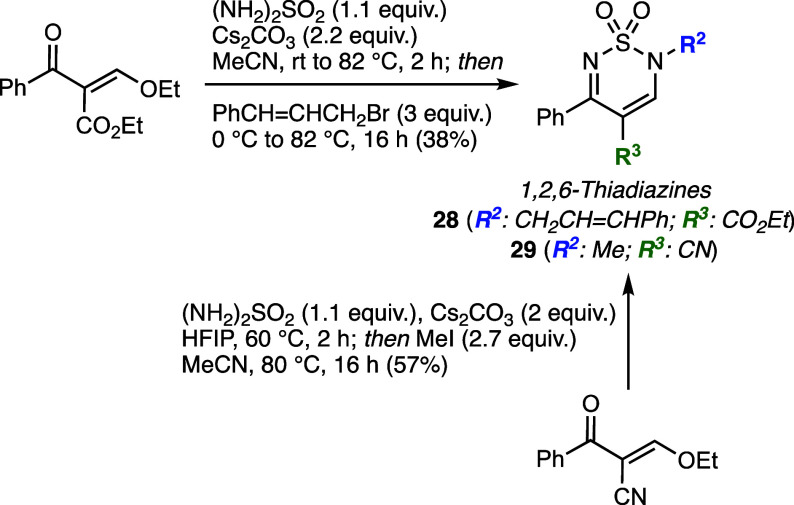
One-Pot Synthesis of *N*(2)-Substituted 1,2,6-Thiadiazine-1,1-dioxides **28** and **29**

Investigations into the reactivity of 1,2,6-thiadiazine-1,1-dioxides **18**-**29** began with attempts to leverage their innate electron-deficiency. We hypothesized that a 1,3-dipolar cycloaddition would proceed in high regio- and diastereoselectivity with nucleophilic azomethine ylides based on the known reactivity of these 1,3-dipoles with electron-deficient alkenes (Scheme 3).^23^

**Scheme 3:**
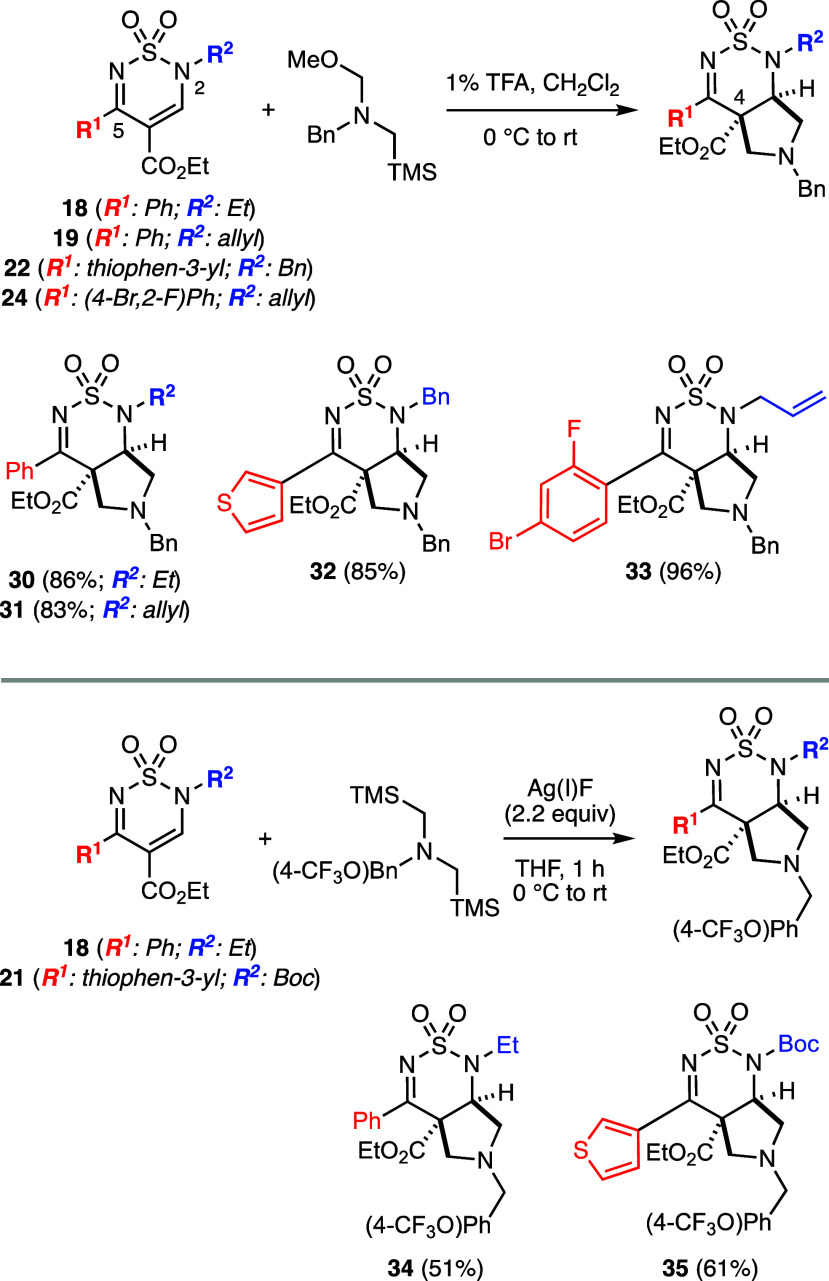
1,3-Dipolar Cycloadditions of 1,2,6-Thiadiazine-1,1-dioxides **18**, **19**, **21**, **22**, and **24** with Azomethine Ylides

After an *in situ* formation of the corresponding azomethine ylide from *N*-benzyl-1-methoxy-*N*-((trimethylsilyl)­methyl)­methanamine under 1% TFA conditions, the addition reaction to thiadiazines **18**, **19**, **22** and **24** proceeded with exclusive *syn*-selectivity to provide the fused bicyclic DHTs **30**-**33** in 83–96% yield. The reaction was tolerant to aryl and heteroaryl substitution at the *C*(5)-position of the 1,2,6-thiadiazine, introducing a quaternary carbon at *C*(4) of the resulting tetrahydropyrrolo 1,2,6-thiadiazine dioxides. *N*-Allyl and *N*-benzyl protecting groups in **19**, **22**, and **24** were also tolerated. For the acid labile Boc-protected substrate **21**, the use of a bis-silyl azomethine ylide precursor and Ag­(I)F provided cycloadduct **35** in 61% yield.^18^ These conditions also delivered the *N*-ethyl product **34** in 51% yield. However, substrates bearing an alkyl group at *C*(5) led to decomposition under either reaction conditions, most likely due to their ease of deprotonation and enamide formation.

For 1,3-dipolar cycloadditions introducing a carbocycle onto the 1,2,6-thiadiazine-1,1-dioxide scaffold, the phosphine-catalyzed annulation of allenoates^20^ was particularly attractive, since this reaction has also been documented on relevant electron-deficient substrates. Initial attempts using ethyl 2-ethyl-5-(4-(trifluoromethyl)­phenyl)-2*H*-1,2,6-thiadiazine-4-carboxylate 1,1-dioxide (**27**) and ethyl 2,3-butadienoate indicated that the selection of the Lewis base was critical for conversion, since the reactions with amines such as 1,4-diazabicyclo[2.2.2]­octane (DABCO) and pyridine were unsuccessful. The softer nucleophile triphenylphosphine, however, efficiently catalyzed the formal cycloaddition with the allenoate at 20 mol % loading (Scheme 4). The s*yn*-annulation occurred with exclusive regio- and diastereoselectivity, affording the carbocyclic analogs of the pyrrolidine series, cyclopentenes **36**–**39** in high yields. The structure of **37** was assigned by X-ray crystallographic analysis (Figure 1).

**Scheme 4:**
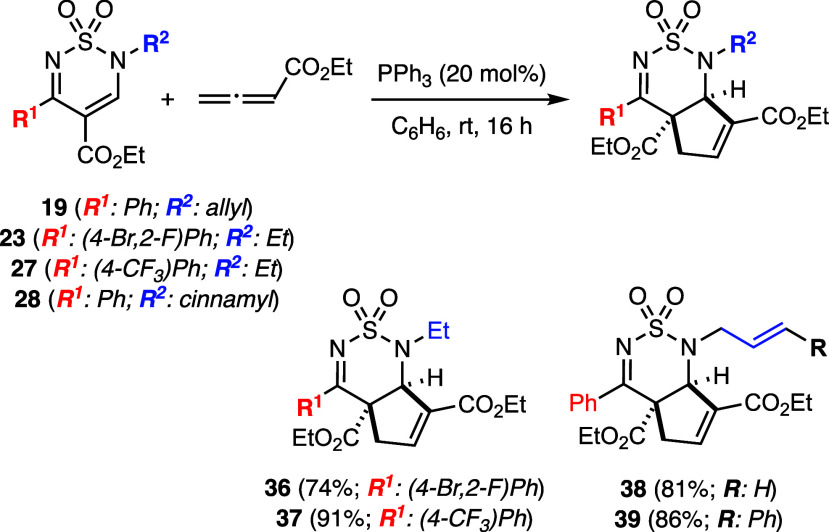
Annulation of 1,2,6-Thiadiazine-1,1-dioxides 19, 23, 27, and 28 with Allenoates

**Figure 1:**
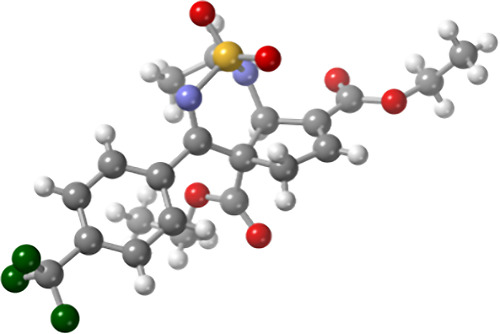
X-ray structure of **37** (CCDC Deposition 2501331).

The successful phosphine-catalyzed annulation of allenoates to 1,2,6-thiadiazine-1,1-dioxides offered the opportunity to test an asymmetric variant using chiral phosphine catalysts.^24^ Treatment of thiadiazine **28** with (*R*)-SITCP (**40**)^25^ provided the enantiomerically enriched tetrahydrocyclopenta­[*c*]­[1,2,6]­thiadiazine 2,2-dioxide (*R,R*)-**39** in 78% yield and an *ee* of 91% by HPLC analysis on chiral stationary phase (Scheme 5). In the presence of the *endo*-4-methoxyphenyl Kwon phosphine **41**,^26^ (*S,S*)-**39** was isolated in 20% yield and an *ee* of 68%, and Kagan’s (+)-DIOP **42** ^27^ provided this product in 51% yield and 32% *ee*.

**Scheme 5:**
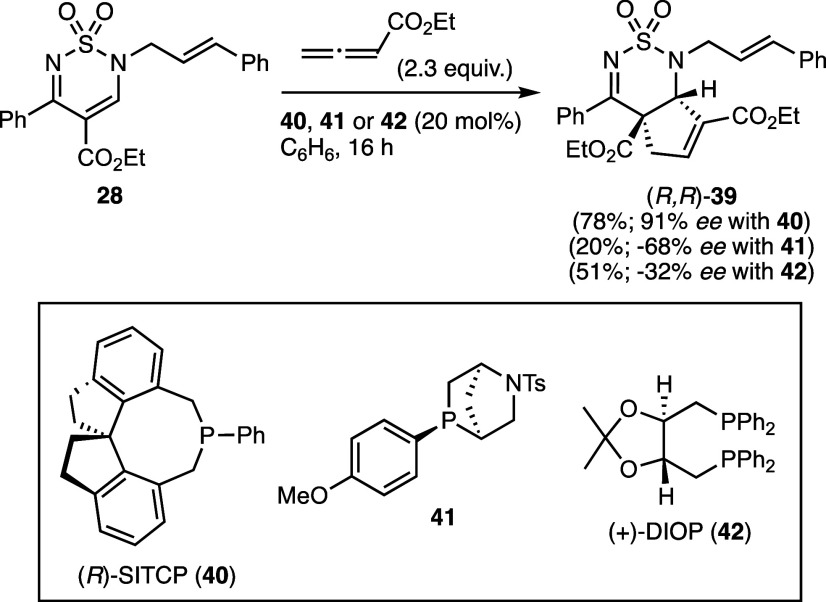
Catalytic Asymmetric [3 + 2] Annulation of 1,2,6-Thiadiazine-1,1-dioxide **28** with Allenoate^a^

a Chiral phosphine **40** provides (*R,R*)-**39** in 91% *ee*.

These asymmetric thiadiazine conversions confirm that highly enantioenriched annulation products can be obtained for potential biological applications of DHT heterocycles. The absolute configuration of the major enantiomer of **39** formed was assigned based on *ab initio* calculations of specific rotation values.^28^

Another unique, bridged bicyclic DHT scaffold was accessible from the cycloaddition with a methylene cyclopropane (MCP) substrate.^29^ While we initially expected this reaction to provide the [2 + 2] azetidine product **44**, both thermal (Table 1, entry 1) and Ag­(I)-catalyzed (Table 1, entry 4) conditions with thiadiazine **18** proceeded through an inverse electron-demand Diels–Alder (IEDDA) reaction to give the [4 + 2] product **43** exclusively in 31% and 15% yield, respectively, as a single diastereomer. We hypothesized that the steric congestion and the unique electronic properties of the 1,2,6-thiadiazine-1,1-dioxide scaffold prevented azetidine formation and instead favored the formation of the bridged IEDDA product. To the best of our knowledge, this is the first report of this spiro­[cyclopropane-1,5′-[2]­thia­[1,3]­diazabicyclo[2.2.2]­octane] scaffold. Efforts to optimize the conversion to **43** demonstrated a significant improvement in yield with excess dienophile, providing product in 74% (Table 1, entry 2). An optimal conversion of 92% was achieved under neat reaction conditions at 100 °C (Table 1, entry 3). In contrast, the use of traditional Lewis acid catalysts was unsuccessful (entries 5–7).

**Table 1:**
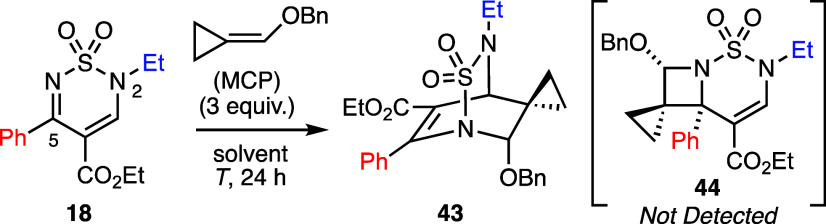
Catalyst Screening and Reaction Optimization for the [4 + 2] Cycloaddition of Methylene Cyclopropane (MCP) to Thiadiazine **18** ^a^

entry	solvent	catalyst (10 mol %)	equiv. dienophile	*T* (°C)	yield^b^ (%)
1	MeCN		1.5	80	31
2	MeCN		3.0	80	74
3	neat		3.0	100	92
4	THF	AgSbF_6_	1.0	rt	15
5	THF	Sc(OTf)_3_	1.0	rt	–
6	toluene	BH_3_·THF	1.5	100	–
7	CH_2_Cl_2_	Cu(SbF_6_)_2_	1.5	–78 to rt	–

a Reactions performed on ≥ 0.2 mmol scale.

b Thiadiazine **18** was recovered for entries with low or no product formation.

Under the optimized reaction conditions, the scope of the IEDDA reaction proved to be quite broad. We found that aryl, heterocyclic, and alkyl substitutions at *C­(*5) of the thiadiazine were well tolerated and afforded the desired bridged DHTs in >75% yield as single diastereomers (Figure 2). Alkyl-, allyl-, benzyl-, and Boc-substitutions at *N*(2) were also tolerated, and the corresponding products **45** to **51** were obtained in high yields. However, some limitations in the dienophile were observed. Attempts to substitute MCP with ethyl vinyl ether, dihydrofuran, and benzyl (*E*)-prop-1-en-1-ylcarbamate proved unsuccessful, providing only unreacted thiadiazine. MCPs without electron-donating ether oxygen substituents, such as ethyl 2-cyclopropylideneacetate and 2-cyclopropylideneethan-1-ol, also did not provide any product with thiadiazine **18**. Accordingly, the IEDDA reaction requires an alkene moiety as dienophile that is not only electron-rich, but also activated by strain,^30^ as present in benzyloxy methylene cyclopropane.

**Figure 2:**
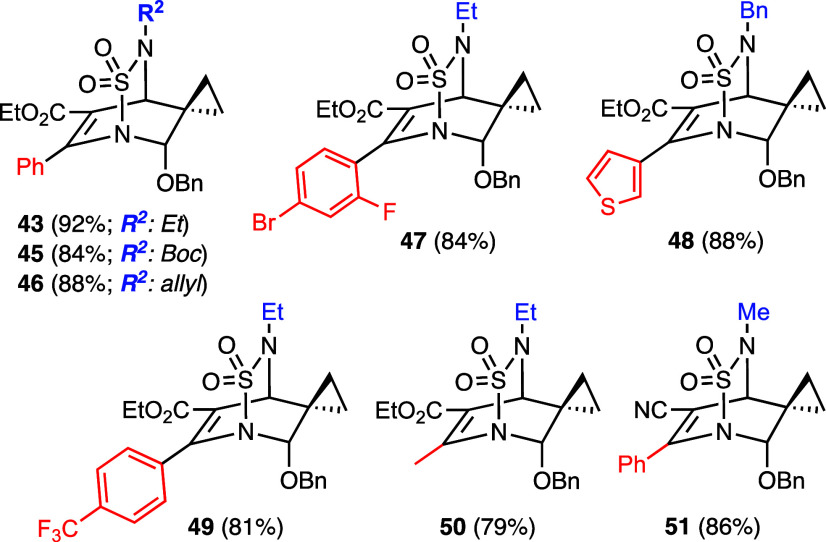
Scope of IEDDA reactions of 1,2,6-thiadiazine-1,1-dioxides with MCP.

IEDDA products have a potential for SO_2_NR^2^ extrusion by a retro-hetero-Diels–Alder reaction. In order to probe this pathway, **43** was heated in mesitylene at 160 °C for 8 h; however, no decomposition was observed, demonstrating the high thermal stability of these novel heterocycles.

The feasibility of further conversions of the thiadiazine cycloadducts was demonstrated by the removal of the protecting groups on **30**, **35**, **39**, and **45** (Scheme 6). The Boc-deprotection of DHT **35** was accomplished by treatment with 25% TFA to afford **52** in 88% yield. While the cyclopropylalkylamine **45** was not tolerant of acid-mediated or thermal deprotection conditions,^31^ removal of the Boc group on this substrate was successful under basic conditions^32^ by reacting **45** with Cs_2_CO_3_ and imidazole in acetonitrile at 70 °C to afford **56** in 64% yield. The hydrogenolysis of benzylated pyrrolidine **30** provided the reduced thiadiazinane **53** in 94% as a 10:1 ratio of diasteromers. The major stereoisomer was assigned using 2D NOESY NMR. Alternatively, treatment of **30** with 1-chloroethyl chloroformate and 2,6-lutidine^33^ followed by methanolysis chemoselectively removed the *N*-benzyl group and led to the DHT hydrochloride **54** in 72% yield. Finally, the deallylation of **39** was readily accomplished under Pd(0)-catalysis in the presence of 1,3-dimethylbarbituric acid to give **55** in 73% yield.

**Scheme 6:**
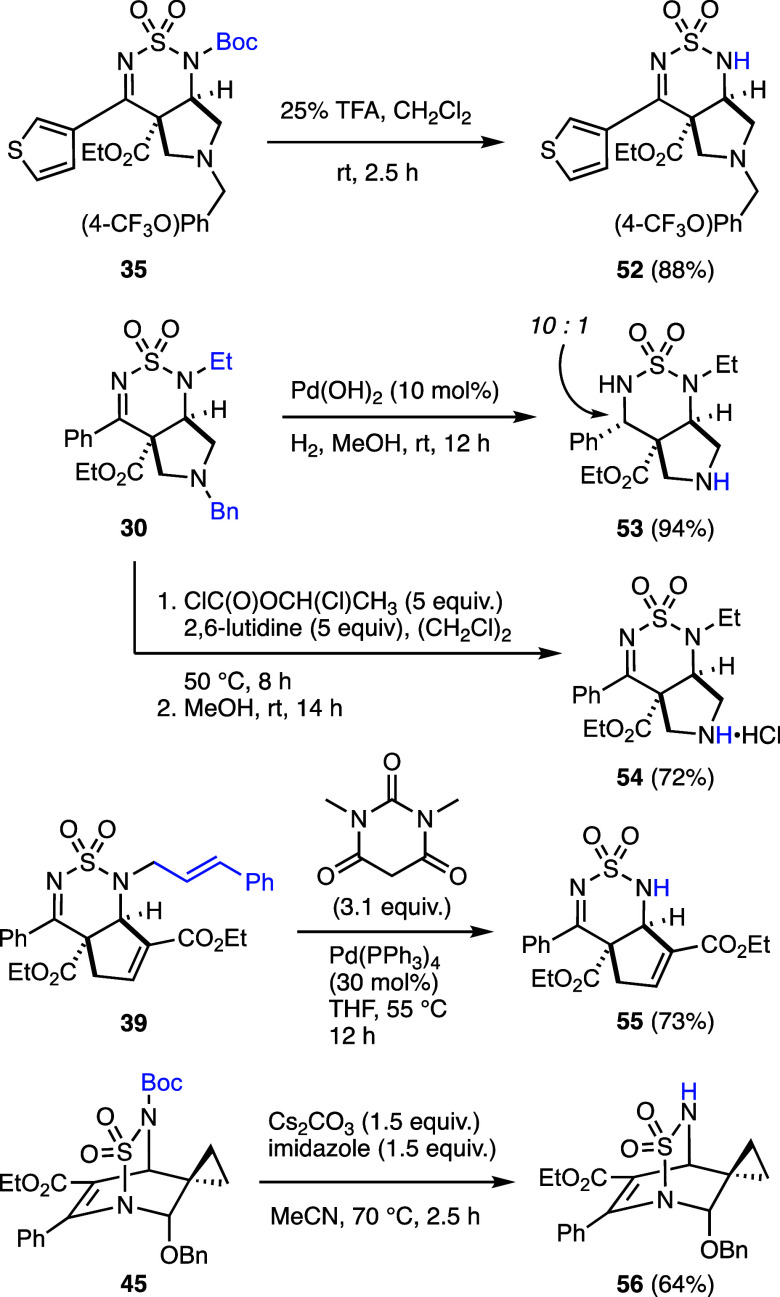
Deprotection of Cycloadducts

In conclusion, we have demonstrated new variants of the Sulfo-Biginelli reaction, in particular a one-pot synthesis from sulfamide and α,β-unsaturated carbonyl compounds, and investigated selective cycloadditions of 1,2,6-thiadiazine-1,1-dioxides.^34^ The 1,3-dipolar cycloaddition with azomethine ylides provided fused pyrrolidine DHTs in high yields and stereoselectivities, and allenoates reacted under phosphine catalysis to generate the corresponding cyclopentene-fused heterocycles. Both processes occur selectively by *syn*-additions and are tolerant of substituent variations on the thiadiazine. The [3 + 2] annulation of 1,2,6-thiadiazine-1,1-dioxides with allenoate could also be performed in an asymmetric fashion using catalytic amounts of chiral phosphines. The diastereoselective IEDDA reaction required the use of an electron-rich methylene cyclopropane to provide unprecedented spiro­[cyclopropane-1,5′-[2]­thia­[1,3]­diazabicyclo[2.2.2]­octane] heterocycles. Interestingly, the corresponding urea-containing scaffold, i.e. the 1,3-diazabicyclo[2.2.2]­octan-2-one, was suggested as a target in 1958,^35^ but has remained synthetically elusive. This discrepancy highlights the increased tolerance for angle strain of cyclic sulfamides versus ureas.

This study showcases the utility of 1,2,6-thiadiazine-1,1-dioxides as versatile precursors of highly functionalized heterocyclic ring systems that are of potential value as pharmaceutical and agrochemical building blocks.^36,37^ Moreover, it suggests a plethora of additional, yet to be discovered synthetic conversions of 1,2,6-thiadiazines.

## Supplementary Material



## Data Availability

The data underlying this study are available in the published article and its online Supporting Information.

## Notes

Experimental details including specific rotation calculations, HPLC traces for **39**, and ^1^H and ^13^C NMR spectra for new synthetic intermediates and products (PDF)
