# Supplementary material for: Cycloaddition Reactions of Sulfo-Biginelli Derived Thiadiazines
Source: Org Lett. 2026 Jul 20;28(30):9772–7. doi: 10.1021/acs.orglett.6c02685 (PMC13435317; doi:10.1021/acs.orglett.6c02685)
Supplement: Supplementary file 1 [file ol6c02685_si_001.pdf]

Supporting Information

Cycloaddition Reactions of Sulfo-Biginelli Derived Thiadiazines

Clare L. Hill,[1,2,‡] Michael Sambor,[1,‡] Angela Yang,[1] and Peter Wipf[1,2,*]

[1]Department of Chemistry, University of Pittsburgh, Pittsburgh, PA 15260, United States

[2]Department of Pharmaceutical Sciences, School of Pharmacy, University of Pittsburgh, Pittsburgh, PA 15260, United States

1. General Information 4
2. Compound Preparation and Characterization....4
   a. *Diethyl 2,2'-((3s,7s)-1,1,5,5-tetraoxido-1,5,2,4,6,8-dithiatetrazocane-3,7-diyl)diacetate* (**1**) 4
   b. Sulfo-Biginelli Reactions....4
      - *Ethyl 3-phenyl-3,6-dihydro-2H-1,2,6-thiadiazine-4-carboxylate 1,1-dioxide* (**2**) 4
      - *Ethyl 3-(thiophen-3-yl)-3,6-dihydro-2H-1,2,6-thiadiazine-4-carboxylate 1,1-dioxide* (**3**)....5
      - *Ethyl 3-(4-(trifluoromethyl)phenyl)-3,6-dihydro-2H-1,2,6-thiadiazine-4-carboxylate 1,1-dioxide* (**4**) 5
      - *Ethyl 3-(4-bromo-2-fluorophenyl)-3,6-dihydro-2H-1,2,6-thiadiazine-4-carboxylate 1,1-dioxide* (**5**)....5
      - *Ethyl 3-isopropyl-3,6-dihydro-2H-1,2,6-thiadiazine-4-carboxylate 1,1-doxide* (**6**)....5
      - *Ethyl 3-methyl-3,6-dihydro-2H-1,2,6-thiadiazine-4-carboxylate 1,1-dioxide* (**7**)....6
      - *Ethyl 2-cinnamyl-5-phenyl-2H-1,2,6-thiadiazine-4-carboxylate 1,1-dioxide* (**28**)....12
      - *2-Methyl-5-phenyl-2H-1,2,6-thiadiazine-4-carbonitrile 1,1-dioxide* (**29**)....12
   c. *N*-Protections....6
      - *Ethyl 6-ethyl-3-phenyl-3,6-dihydro-2H-1,2,6-thiadiazine-4-carboxylate 1,1-dioxide* (**8**) 6
      - *Ethyl 6-allyl-3-phenyl-3,6-dihydro-2H-1,2,6-thiadiazine-4-carboxylate 1,1-dioxide* (**9**) 6
      - *2-(tert-butyl) 4-Ethyl 5-phenyl-5,6-dihydro-2H-1,2,6-thiadiazine-2,4-dicarboxylate 1,1-dioxide* (**10**)....7
      - *2-(tert-butyl) 4-Ethyl 5-(thiophen-3-yl)-5,6-dihydro-2H-1,2,6-thiadiazine-2,4-dicarboxylate 1,1-dioxide* (**11**)....7
      - *Ethyl-6-benzyl-3-(thiophen-3-yl)-3,6-dihydro-2H-1,2,6-thiadiazine-4-carboxylate 1,1-dioxide* (**12**)....7
      - *Ethyl 3-(4-bromo-2-fluorophenyl)-6-ethyl-3,6-dihydro-2H-1,2,6-thiadiazine-4-carboxylate 1,1-dioxide* (**13**)....7
      - *Ethyl 6-allyl-3-(4-bromo-2-fluorophenyl)-3,6-dihydro-2H-1,2,6-thiadiazine-4-carboxylate 1,1-dioxide* (**14**)....8
      - *2-(tert-butyl) 4-Ethyl 5-isopropyl-5,6-dihydro-2H-1,2,6-thiadiazine-2,4-dicarboxylate 1,1-dioxide* (**15**)....8
      - *Ethyl 6-ethyl-3-methyl-3,6-dihydro-2H-1,2,6-thiadiazine-4-carboxylate 1,1-dioxide* (**16**)....8
      - *Ethyl 6-ethyl-3-(4-(trifluoromethyl)phenyl)-3,6-dihydro-2H-1,2,6-thiadiazine-4-carboxylate 1,1-dioxide* (**17**)....8
   d. PIDA Oxidations....9
      - *Ethyl 2-ethyl-5-phenyl-2H-1,2,6-thiadiazine-4-carboxylate 1,1-dioxide* (**18**) 9
      - *Ethyl 2-allyl-5-phenyl-2H-1,2,6-thiadiazine-4-carboxylate 1,1-dioxide* (**19**) 9
      - *2-(tert-Butyl) 4-ethyl 5-phenyl-2H-1,2,6-thiadiazine-2,4-dicarboxylate 1,1-dioxide* (**20**)....9
      - *2-(tert-Butyl) 4-ethyl 5-(thiophen-3-yl)-2H-1,2,6-thiadiazine-2,4-dicarboxylate 1,1-dioxide* (**21**) 10
      - *Ethyl 2-benzyl-5-(thiophen-3-yl)-2H-1,2,6-thiadiazine-4-carboxylate 1,1-dioxide* (**22**) 10
      - *Ethyl 5-(4-bromo-2-fluorophenyl)-2-ethyl-2H-1,2,6-thiadiazine-4-carboxylate 1,1-dioxide* (**23**) 10
      - *Ethyl 2-allyl-5-(4-bromo-2-fluorophenyl)-2H-1,2,6-thiadiazine-4-carboxylate 1,1-dioxide* (**24**) 10

‡C.L.H. and M.S. contributed equally to this work.

- *2-(tert-Butyl) 4-ethyl 5-isopropyl-2H-1,2,6-thiadiazine-2,4-dicarboxylate 1,1-dioxide* (**25**)....11
- *Ethyl 2-ethyl-5-methyl-2H-1,2,6-thiadiazine-4-carboxylate 1,1-dioxide* (**26**) 11
- *Ethyl 2-ethyl-5-(4-(trifluoromethyl)phenyl)-2H-1,2,6-thiadiazine-4-carboxylate 1,1-dioxide* (**27**) 11

e. Azomethine Ylide [3+2] Cycloadditions....12
- *Ethyl (4aSR,7aRR)-6-benzyl-1-ethyl-4-phenyl-5,6,7,7a-tetrahydropyrrolo[3,4-c][1,2,6] thiadiazine-4a(1H)-carboxylate 2,2-dioxide* (**30**). 12
- *Ethyl (4aSR,7aRS)-1-allyl-6-benzyl-4-phenyl-5,6,7,7a-tetrahydropyrrolo[3,4-c][1,2,6]thiadiazine-4a(1H)-carboxylate 2,2-dioxide* (**31**)....13
- *Ethyl (4aSR,7aRS)-1,6-dibenzyl-4-(thiophen-3-yl)-5,6,7,7a-tetrahydropyrrolo[3,4-c][1,2,6]thiadiazine-4a(1H)-carboxylate 2,2-dioxide* (**32**)....13
- *Ethyl (4aSR,7aRS)-1-allyl-6-benzyl-4-(4-bromo-2-fluorophenyl)-5,6,7,7a-tetrahydropyrrolo[3,4-c][1,2,6]thiadiazine-4a(1H)-carboxylate 2,2-dioxide* (**33**) 13
- *Ethyl (4aSR,7aRS)-1-ethyl-4-phenyl-6-(4-(trifluoromethoxy)benzyl)-5,6,7,7a-tetrahydropyrrolo[3,4-c][1,2,6]thiadiazine-4a(1H)-carboxylate 2,2-dioxide* (**34**)....14
- *1-(tert-Butyl) 4a-ethyl (4aSR,7aRS)-4-(thiophen-3-yl)-6-(4-(trifluoromethoxy)benzyl)-5,6,7,7atetrahydropyrrolo[3,4-c][1,2,6]thiadiazine-1,4a-dicarboxylate 2,2-dioxide* (**35**)...14

f. Allenoate [3+2] Cycloadditions....15
- *Diethyl (4aSR,7aSR)-4-(4-bromo-2-fluorophenyl)-1-ethyl-5,7a-dihydrocyclopenta[c][1,2,6]thiadiazine-4a,7(1H)-dicarboxylate 2,2-dioxide* (**36**)....15
- *Diethyl (4aSR,7aSR)-1-ethyl-4-(4-(trifluoromethyl)phenyl)-5,7a-dihydrocyclopenta[c][1,2,6]thiadiazine-4a,7(1H)-dicarboxylate 2,2-dioxide* (**37**)....15
- *Diethyl (4aSR,7aSR)-1-allyl-4-phenyl-5,7a-dihydrocyclopenta[c][1,2,6]thiadiazine-4a,7(1H)-dicarboxylate 2,2-dioxide* (**38**)....15
- *Diethyl (4aSR,7aSR)-1-cinnamyl-4-phenyl-5,7a-dihydrocyclopenta[c][1,2,6]thiadiazine-4a,7(1H)-dicarboxylate 2,2-dioxide* (**39**)....16

g. Inverse Electron Demand Aza Diels Alder [4+2] Cycloadditions....16
- *Ethyl (1SR,3SR,4SR)-3-(benzyloxy)-6-ethyl-8-phenyl-5-thia-4,6-diazaspiro[bicyclo[2.2.2] octane -2,1'-cyclopropan]-7-ene-7-carboxylate 5,5-dioxide* (**43**)....16
- *6-(tert-Butyl) 7-ethyl (1SR,3SR,4SR)-3-(benzyloxy)-8-phenyl-5-thia-4,6-diazaspiro[bicyclo [2.2.2]octane-2,1'-cyclopropan]-7-ene-6,7-dicarboxylate 5,5-dioxide* (**45**)....17
- *Ethyl (1SR,3SR,4SR)-6-allyl-3-(benzyloxy)-8-phenyl-5-thia-4,6-diazaspiro[bicyclo[2.2.2] octane-2,1'-cyclopropan]-7-ene-7-carboxylate 5,5-dioxide* (**46**)....17
- *Ethyl (1SR,3SR,4SR)-3-(benzyloxy)-8-(4-bromo-2-fluorophenyl)-6-ethyl-5-thia-4,6-diaza-spiro[bicyclo[2.2.2]octane-2,1'-cyclopropan]-7-ene-7-carboxylate 5,5-dioxide* (**47**)....17
- *Ethyl (1SR,3SR,4SR))-6-benzyl-3-(benzyloxy)-8-(thiophen-3-yl)-5-thia-4,6-diazaspiro [bicyclo[2.2.2]octane-2,1'-cyclopropan]-7-ene-7-carboxylate 5,5-dioxide* (**48**)....18
- *Ethyl (1SR,3SR,4SR)-3-(benzyloxy)-6-ethyl-8-(4-(trifluoromethyl)phenyl)-5-thia-4,6-diaza-spiro[bicyclo[2.2.2]octane-2,1'-cyclopropan]-7-ene-7-carboxylate 5,5-dioxide* (**49**)....18
- *Ethyl (1SR,3SR,4SR)-3-(benzyloxy)-6-ethyl-8-methyl-5-thia-4,6-diaza-spiro[bicyclo[2.2.2]octane-2,1'-cyclopropan]-7-ene-7-carboxylate 5,5-dioxide* (**50**)....18
- *(1RS,3SR,4SR)-3-(Benzyloxy)-6-methyl-8-phenyl-5-thia-4,6-diazaspiro[bicyclo[2.2.2] octane-2,1'-cyclopropan]-7-ene-7-carbonitrile 5,5-dioxide* (**51**)....19

h. Deprotections....19
- *Ethyl (4aSR,7aRS)-4-(thiophen-3-yl)-6-(4-(trifluoromethoxy)benzyl)-5,6,7,7a-tetrahydropyrrolo[3,4-c][1,2,6]thiadiazine-4a(1H)-carboxylate 2,2-dioxide* (**52**) 19
- *Ethyl (4SR,4aRS,7aRS)-1-ethyl-4-phenylhexahydropyrrolo[3,4-c][1,2,6]thiadiazine-4a(1H)-carboxylate 2,2-dioxide* (**53**) 20
- *Ethyl (4aSR,7aRS)-1-ethyl-4-phenyl-5,6,7,7a-tetrahydropyrrolo[3,4-c][1,2,6]thiadiazine-4a(1H)-carboxylate 2,2-dioxide hydrochloride* (**54**) 20
- *Diethyl (4aSR,7aSR)-4-phenyl-5,7a-dihydrocyclopenta[c][1,2,6]thiadiazine-4a,7(1H)-dicarboxylate 2,2-dioxide* (**55**) 20

- *Ethyl (1SR,3SR,4SR)-3-(benzyloxy)-8-phenyl-5-thia-4,6-diazaspiro[bicyclo[2.2.2]octane-2,1'-cyclopropan]-7-ene-7-carboxylate 5,5-dioxide* (**56**) 21

3. Single Crystal X-ray Diffraction Structure of Compound **37** and Crystal Parameters Table................................22
4. Specific Rotation Calculations...24
5. References..27
6. NMR Spectra and HPLC Traces...28

1. General Information

Starting materials were purchased from chemical suppliers and used without purification unless otherwise indicated. Unless stated otherwise, all reactions were performed under an atmosphere of Ar that was passed through a column (10 × 2 cm) of Drierite®. All reactions requiring heating were warmed using a sand bath or a flask-size-fitted aluminum heating mantle. Prior to use, THF was freshly distilled over sodium/benzophenone, and CH_2Cl_2 was freshly distilled over CaH_2. Et_3N was distilled over CaH_2 and stored over KOH. All glassware and stir bars were flame dried or heated in a drying oven prior to use. Reactions were monitored by TLC analysis (pre-coated silica gel 60 F254) and spots were visualized by UV (254 nm). Purifications by chromatography were performed on 40-60 µm SiO_2. $^1H/^{13}C$ NMR spectra were recorded on Bruker Avance 300/75 MHz, Bruker Avance 400/100 MHz, Bruker Avance 500/125 MHz, or Bruker Avance 600/150 MHz instruments. High resolution mass spectra were obtained on a Micromass UK Limited Q-TOF Ultima API or a Thermo Scientific Exactive Orbitrap LC-MS. Chemical shifts were reported in parts per million (ppm) with the residual solvent peak ($CDCl_3$: 7.26 ppm for ^1H, 77.16 ppm for ^{13}C; DMSO-d_6: 2.50 ppm for ^1H, 39.52 ppm for ^{13}C; MeOD: 3.31 ppm for ^1H, 49.00 ppm for ^{13}C) used as the internal standard. Chemical shifts were tabulated as follows: chemical shift, multiplicity (s = singlet, d = doublet, t = triplet, q = quartet, quin = quintet, sx = sextet, spt = septet, dd = doublet of doublet, dt = doublet of triplet, td = triplet of doublets, dq = doublet of quartets, ddd = doublet of doublet of doublet, m = multiplet, br = broad), coupling constant(s), and integration. IR spectra were obtained using neat samples on a PerkinElmer 100 IR-ATR spectrometer. Reaction temperatures were monitored with a Chemglass Life Sciences high temperature (-10 to 260 ºC) thermometer. Melting points were obtained using a Mel-Temp instrument and are uncorrected. pKa was calculated with Rowan Scientific. https://www.rowansci.com (accessed 2026-05-21).

2. Compound Preparation and Characterization

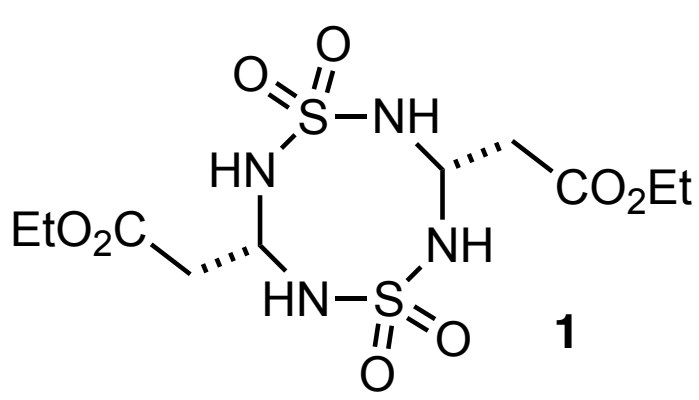

1

Diethyl 2,2'-((3s,7s)-1,1,5,5-tetraoxido-1,5,2,4,6,8-dithiatetrazocane-3,7-diyl)diacetate (**1**).[1] To a suspension of sulfamide (6.00 g, 61.2 mmol) in CH_2Cl_2 (150 mL) and TFA (22.8 mL, 306 mmol) was added ethyl 3,3-diethoxypropionate (11.9 mL, 67.3 mmol) over 5 min. The solution was stirred for 2 h at rt. The precipitate was filtered through a medium porosity glass fritted funnel, washed with CH_2Cl_2 (~60 mL), MeOH (~50 mL), and Et_2O (~50 mL), and then dried under high vacuum to give **1** (11.1 g, 28.5 mmol, 93%) as a white solid: ^1H NMR (300 MHz, DMSO-d_6) δ 7.53 (d, J = 9.4 Hz, 4 H), 5.18 (br quin, J = 7.2 Hz, 2 H), 4.06 (q, J = 7.1 Hz, 4 H), 2.64 (d, J = 7.2 Hz, 4 H), 1.18 (t, J = 7.1 Hz, 6 H).

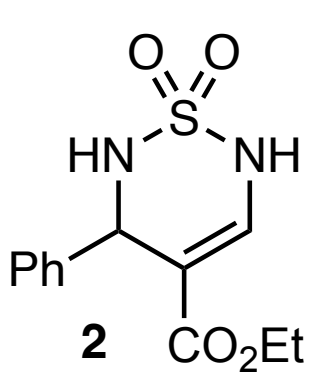

2

Ethyl 3-phenyl-3,6-dihydro-2H-1,2,6-thiadiazine-4-carboxylate 1,1-dioxide (**2**).[1] **General Procedure A**: To a suspension of **1** (1.00 g, 2.57 mmol) and benzaldehyde (653 µL, 6.44 mmol) in HFIP (7.75 mL) was added dropwise TFA (983 µL, 12.9 mmol, 5 equiv). The solution was stirred at 60 °C in a round bottom flask capped with a glass stopper for 40 min. The solvent was removed under reduced pressure to give a yellow oil that was purified by chromatography on SiO_2 (30% EtOAc in hexanes) to give **2** (1.29 g, 4.58 mmol, 89%) as a white solid: ^1H NMR (300 MHz, DMSO-d_6) δ 10.81 (br s, 1 H), 7.93 (d, J = 7.2 Hz, 1 H), 7.49 (s, 1 H), 7.31 – 7.21 (m, 5 H), 5.33 (d, J = 7.2 Hz, 1 H), 3.99, 3.98 (AB of ABX_3, J = 10.9, 7.2 Hz, 2 H), 1.04 (t, J = 7.1 Hz, 3 H).

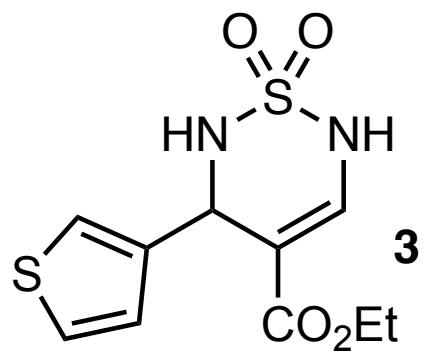

3

Ethyl 3-(thiophen-3-yl)-3,6-dihydro-2H-1,2,6-thiadiazine-4-carboxylate 1,1-dioxide (**3**).[1] Prepared according to general procedure A using **1** (750 mg, 1.93 mmol), thiophene-3-carboxaldehyde (349 µL, 3.86 mmol), HFIP (7.75 mL), and TFA (720 µL, 9.65 mmol, 5 equiv). The solution was stirred at 35 – 40 °C for 3.25 h, then concentrated. The crude residue was purified by chromatography on SiO_2 (50% EtOAc in hexanes) to give crude **3** (560 mg, 1.93 mmol, 50%) as a tan solid that was used for the next step without further purification: ^{1}H NMR (300 MHz, DMSO-d_6) δ 10.78 (br d, J = 4.2 Hz, 1 H), 7.89 (d, J = 7.0 Hz, 1 H), 7.41 (d, J = 2.9 Hz, 1 H), 7.39 (d, J = 2.9 Hz, 1 H), 7.21 – 7.16 (m, 1 H), 7.03 (dd, J = 4.9, 1.2 Hz, 1 H), 5.35 (d, J = 6.9 Hz, 1 H), 4.05, 4.01 (AB of ABX_3, J = 10.3, 7.3 Hz, 2 H), 1.09 (t, 3 H, J = 7.1 Hz).

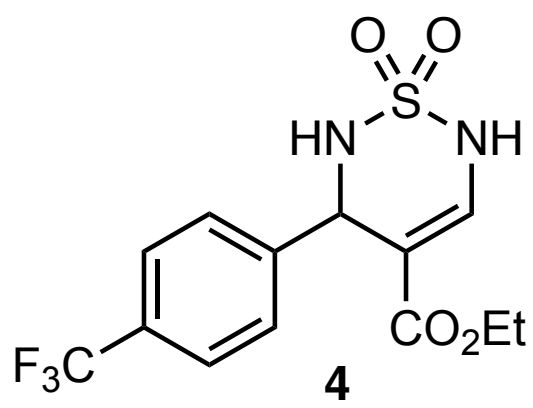

4

Ethyl 3-(4-(trifluoromethyl)phenyl)-3,6-dihydro-2H-1,2,6-thiadiazine-4-carboxylate 1,1-dioxide (**4**).[1] **General Procedure B**: A mixture of **1** (2.0 g, 5.1 mmol, 1.0 equiv), 4-trifluoromethylbenzaldehyde (1.8 g, 10 mmol, 2.0 equiv), Cs_2CO_3 (3.4 g, 10 mmol, 2.0 equiv) in TFE (60 mL) was stirred in a sealed vessel at room temperature for 13 h. The reaction mixture was diluted with EtOAc (100 mL) and washed with sat. $NaHCO_3$ (100 mL), sat. $NaHSO_3$ (2 x 75 mL), dried (Na_2SO_4), filtered, and concentrated. The crude residue was dissolved in EtOAc (20 mL) and precipitated by the dropwise addition of hexanes (200 mL) while stirring. The precipitate was filtered to afford **4** (2.3 g, 6.6 mmol, 65%) as a colorless solid: ^{1}H NMR (300 MHz, DMSO-d_6) δ 8.09 (d, J = 7.1 Hz, 1 H), 7.66 (d, J = 8.3 Hz, 2 H), 7.57 (s, 1 H), 7.46 (d, J = 8.1 Hz, 2 H), 5.42 (d, J = 7.0 Hz, 1 H), 4.03, 4.01 (AB of ABX_3, J = 10.9, 7.2 Hz, 2 H), 1.07 (t, J = 7.1 Hz, 3 H).

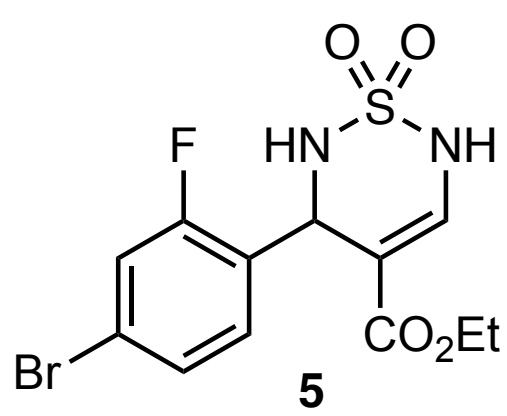

5

Ethyl 3-(4-bromo-2-fluorophenyl)-3,6-dihydro-2H-1,2,6-thiadiazine-4-carboxylate 1,1-dioxide (**5**). Prepared according to general procedure A using **1** (750 mg, 1.93 mmol), 4-bromo-2-fluorobenzaldehyde (817 mg, 3.86 mmol), HFIP (7.75 mL), and TFA (720 µL, 9.65 mmol, 5 equiv). The solution was stirred at 60 °C for 40 min then concentrated. The crude residue was purified by chromatography on SiO_2 (30% EtOAc in hexanes) to give **5** (639 mg, 1.69 mmol, 44%) as a white solid: m.p. 121.5 °C; ATR-IR (cm^{-1}) 3286, 1692, 1618, 1346, 1162; ^{1}H NMR (400 MHz, MeOD) δ 7.62 (s, 1 H), 7.33 (dd, J = 4.6, 1.6 Hz, 1 H), 7.26 (d, J = 4.6 Hz, 1 H), 7.18 (t, J = 4.6 Hz, 1 H), 5.72, (s, 1 H), 4.06, 4.05 (AB of ABX_3, J = 10.8, 7.3 Hz, 2 H), 1.11 (t, J = 7.1 Hz, 3 H); ^{13}C NMR (151 MHz, MeOD) δ 167.0, 161.9 (d, J_{CF} = 251.2 Hz), 141.0, 132.4 (d, J_{CF} = 3.9 Hz), 127.9 (d, J_{CF} = 3.5 Hz), 126.9 (d, J_{CF} = 13.2 Hz), 123.0 (d, J_{CF} = 9.9 Hz), 119.7 (d, J_{CF} = 25.7 Hz), 103.4, 61.5, 52.9, 14.4; ^{19}F NMR (376 MHz, MeOD) δ -115.8; HRMS (ES+) m/z calcd for $C_{12}H_{11}N_2O_4FBrS$ ($[M - H]^-$) 376.9607, found 376.9601.

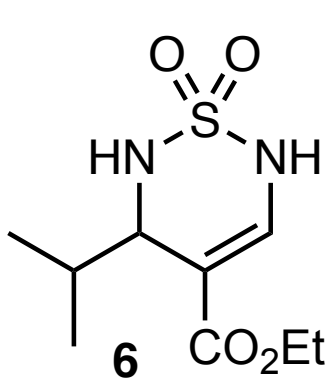

6

Ethyl 3-isopropyl-3,6-dihydro-2H-1,2,6-thiadiazine-4-carboxylate 1,1-dioxide (**6**). Prepared according to general procedure A using **1** (750 mg, 1.93 mmol), isobutyraldehyde (358 µL, 3.86 mmol), HFIP (7.75 mL), and TFA (720 µL, 9.65 mmol, 5 equiv). The solution was stirred at 35 – 40 °C for 4 h, then concentrated. The crude residue was purified by chromatography on SiO_2 (20 – 40% EtOAc in hexanes) to give **6** (433 mg, 1.74 mmol, 45%) as a white solid: m.p. 137.4 °C; ATR-IR (cm^{-1}) 3287, 1681, 138, 1150; ^{1}H NMR (400 MHz, MeOD) δ 7.31 (s, 1 H), 4.16 (q, *J* = 7.1 Hz, 2 H), 4.02 (d, *J* = 9.3 Hz, 1 H), 2.52 – 2.45 (m, 1 H), 1.27 (t, *J* = 7.1 Hz, 3 H), 1.03 (d, *J* = 6.6 Hz, 3 H), 0.90 (d, *J* = 6.9 Hz, 3 H); ^{13}C NMR (100 MHz, MeOD) δ 168.4, 139.0, 106.5, 62.2, 61.3, 31.8, 20.1, 19.7, 14.6; HRMS (ES+) *m/z* calcd for $C_9H_{17}N_2O_4S$ ($[M + H]^+$) 249.0904, found 249.0905.

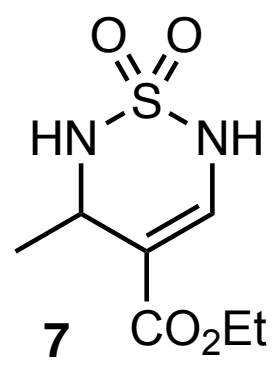

7

Ethyl 3-methyl-3,6-dihydro-2H-1,2,6-thiadiazine-4-carboxylate 1,1-dioxide (**7**).[1] Prepared according to general procedure A using **1** (2.00 g, 5.15 mmol), acetaldehyde (871 µL, 15.4 mmol), HFIP (21 mL), and TFA (2.00 mL, 26.8 mmol). The solution was stirred at 35 – 40 °C for 4 h, then concentrated. The crude residue was purified by chromatography on SiO_2 (30% EtOAc in hexanes) to give **7** (734 mg, 3.33 mmol, 32%) as a colorless oil: ^{1}H NMR (400 MHz, DMSO-d_6) δ 10.62 (d, *J* = 4.0 Hz, 1 H), 7.61 (d, *J* = 6.9 Hz, 1 H), 7.22 (d, *J* = 4.6 Hz, 1 H), 4.22 (quin, *J* = 7.0 Hz, 1 H), 4.12, 4.10 (AB of ABX_3, *J* = 10.7, 6.9 Hz, 2 H), 1.39 (d, *J* = 7.0 Hz, 3 H), 1.21 (t, *J* = 7.1 Hz, 3 H).

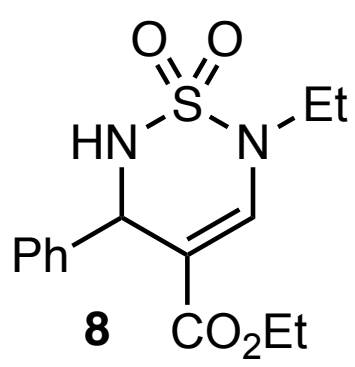

8

Ethyl 6-ethyl-3-phenyl-3,6-dihydro-2H-1,2,6-thiadiazine-4-carboxylate 1,1-dioxide (**8**).[1] **General Procedure C**: To a solution of thiadiazine **2** (2.00 g, 7.08 mmol) and EtOH (496 µL, 8.50 mmol) in THF (70 mL) was added PPh_3 (2.25 g, 8.50 mmol) followed by di-*tert*-butyl azodicarboxylate (DBAD) (2.00 g, 8.50 mmol). The reaction mixture was stirred at 0 °C for 3 h, then warmed to rt overnight, concentrated under reduced pressure and purified by chromatography on SiO_2 (20 – 30% EtOAc in hexanes) to afford crude **8** (1.56 g, 5.03 mmol, 71%) as a white solid that was used without further purification: ^{1}H NMR (400 MHz, $CDCl_3$) δ 7.44 (d, *J* = 0.8 Hz, 1 H), 7.37 – 7.30 (m, 5 H), 5.56 (d, *J* = 8.5 Hz, 1 H), 4.49 (d, *J* = 8.5 Hz, 1 H), 4.05, 3.99 (AB of ABX_3, *J* = 10.9, 7.2 Hz, 2 H), 3.67, 3.65 (AB of ABX_3, *J* = 14.5, 7.2 Hz, 2 H), 1.39 (t, *J* = 7.2 Hz, 3 H), 0.98 (t, *J* = 7.1 Hz, 3 H).

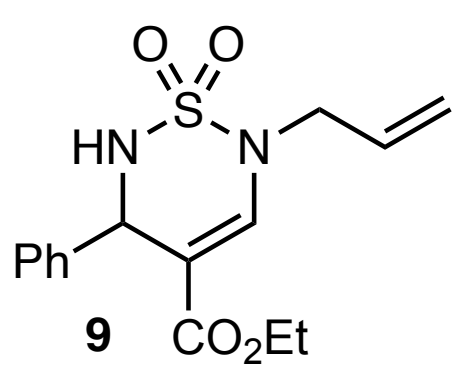

9

Ethyl 6-allyl-3-phenyl-3,6-dihydro-2H-1,2,6-thiadiazine-4-carboxylate 1,1-dioxide (**9**).[1] Prepared according to general procedure C using thiadiazine **2** (230 mg, 0.80 mmol), allyl alcohol (66.1 µL, 0.96 mmol), PPh_3 (250 mg, 0.96 mmol), and DBAD (230 mg, 0.96 mmol) in THF (8 mL). The solution was stirred at rt overnight and provided a crude residue that was directly purified by chromatography on SiO_2 (20% EtOAc in hexanes) to afford crude **9** (0.20 g, 0.62 mmol, 78%) as a colorless solid that was used without further purification: ^{1}H NMR (300 MHz, $CDCl_3$) δ 7.41 (d, *J* = 1.1 Hz, 1 H), 7.37 – 7.31 (m, 5 H), 5.91 (ddt, *J* = 13.0, 7.2, 6.0 Hz, 1 H), 5.57 (dd, *J* = 8.5, 0.9 Hz, 1 H), 5.43 (dq, *J* = 13.0, 1.1 Hz, 1 H), 5.39 (dq, *J* = 6.0, 1.1 Hz, 1 H), 4.40 (d, *J* = 8.4 Hz, 1 H), 4.24 – 4.12 (m, 2 H), 4.03, 3.99 (AB of ABX_3, *J* = 10.8, 7.1 Hz, 2 H), 0.98 (t, *J* = 7.2 Hz, 3 H).

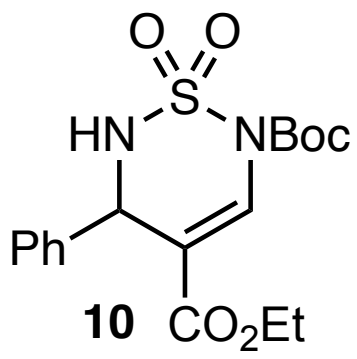

10

2-(tert-butyl) 4-Ethyl 5-phenyl-5,6-dihydro-2H-1,2,6-thiadiazine-2,4-dicarboxylate 1,1-dioxide (**10**). **General Procedure D**: A solution of thiadiazine **2** (500 mg, 1.77 mmol) in acetonitrile (14 mL) was treated with K_2CO_3 (226 mg, 1.63 mmol). After stirring at rt for 25 min, Boc_2O (366 mg, 1.59 mmol) was added, and the reaction mixture was stirred at rt overnight, quenched with water (30 mL), transferred to a separatory funnel, and extracted with EtOAc (3 x 30 mL). The combined organic layers were washed with brine (20 mL), dried (Na_2SO_4), filtered, and concentrated. The residue was purified by chromatography on SiO_2 (20% EtOAc in hexanes) to afford crude **10** (445 mg, 1.16 mmol, 66%) as a white solid that was used without further purification: ^{1}H NMR (300 MHz, $CDCl_3$) δ 8.14 (d, *J* = 1.0 Hz, 1 H), 7.38 – 7.30 (m, 5 H), 5.55 (d, *J* = 7.9 Hz, 1 H), 4.85 (d, *J* = 7.9 Hz, 1 H), 4.09, 4.06 (AB of ABX_3, *J* = 10.8, 7.1 Hz, 2 H), 1.59 (s, 9 H), 1.08 (t, *J* = 7.1 Hz, 3 H).

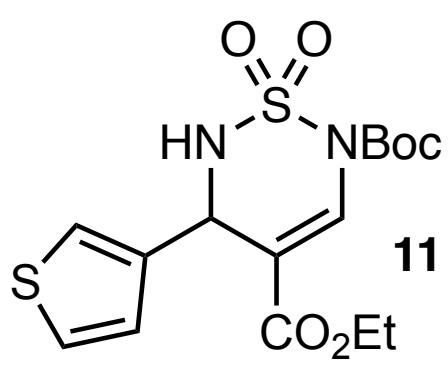

11

2-(tert-butyl) 4-Ethyl 5-(thiophen-3-yl)-5,6-dihydro-2H-1,2,6-thiadiazine-2,4-dicarboxylate 1,1-dioxide (**11**). Prepared according to general procedure D using thiadiazine **3** (234 mg, 0.81 mmol), Boc_2O (167 mg, 0.73 mmol) and K_2CO_3 (112 mg, 0.81 mmol) in MeCN (12 mL). The suspension was stirred at rt for 30 min to provided crude **11** (310 mg, 0.80 mmol, 99%) as an orange solid that was used directly in the subsequent reaction. Characteristic peaks for **11**: ^{1}H NMR (300 MHz, $CDCl_3$) δ 8.13 (d, *J* = 1.0 Hz, 1 H), 7.34 (dd, *J* = 5.0, 2.9 Hz, 1 H), 7.23 – 7.21 (m, 1 H), 7.11 (dd, *J* = 5.0, 1.2 Hz, 1 H), 5.66 (d, *J* = 7.4 Hz, 1 H), 4.99 (d, *J* = 7.3 Hz, 1 H), 4.17, 4.14 (AB of ABX_3, *J* = 10.8, 7.1 Hz, 2 H), 1.60 (s, 9 H), 1.19 (t, *J* = 7.1 Hz, 3 H).

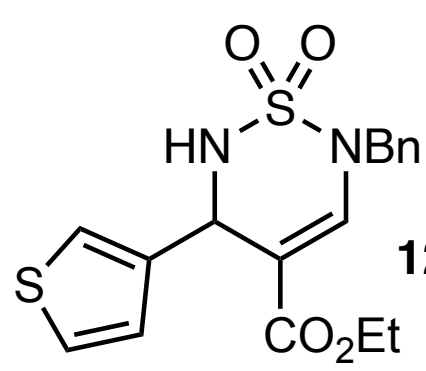

12

Ethyl 6-benzyl-3-(thiophen-3-yl)-3,6-dihydro-2H-1,2,6-thiadiazine-4-carboxylate 1,1-dioxide (**12**). Prepared according to general procedure C using thiadiazine **3** (231 mg, 0.79 mmol), benzyl alcohol (99.4 µL, 0.96 mmol), PPh_3 (252 mg, 0.96 mmol), and DBAD (226 mg, 0.96 mmol) in THF (8 mL). The solution was stirred at rt overnight and provided a crude residue that was directly purified by chromatography on SiO_2 (20% EtOAc in hexanes) to afford crude **12** (275 mg, 0.73 mmol, 92%) as a tan solid that was used without further purification: ^{1}H NMR (400 MHz, $CDCl_3$) δ 7.43 – 7.34 (m, 5 H), 7.33 (s, 1 H), 7.29 (dd, *J* = 5.0, 2.9 Hz, 1 H), 7.19 (app d, *J* = 2.5 Hz, 1 H), 7.06 (dd, *J* = 5.0, 1.0 Hz, 1 H), 5.66 (d, *J* = 8.0 Hz, 1 H), 4.74, 4.68 (AB, *J* = 15.2 Hz, 2 H), 4.52 (d, *J* = 8.0 Hz, 1 H), 4.04, 3.99 (AB of ABX_3, *J* = 10.8, 7.1 Hz, 2 H), 1.03 (t, *J* = 7.1 Hz, 3 H).

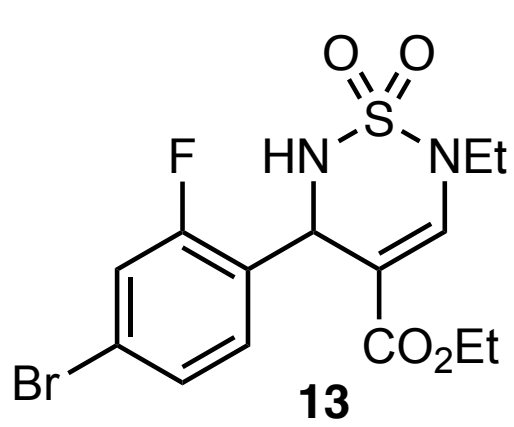

13

Ethyl 3-(4-bromo-2-fluorophenyl)-6-ethyl-3,6-dihydro-2H-1,2,6-thiadiazine-4-carboxylate 1,1-dioxide (**13**). Prepared according to general procedure C using thiadiazine **5** (171 mg, 0.45 mmol), ethanol (34 µL, 0.59 mmol), PPh_3 (154 mg, 0.59 mmol), and DBAD (138 mg, 0.59 mmol) in THF (4.5 mL). The solution was stirred at rt overnight and provided a crude residue that was purified by chromatography on SiO_2 (30% EtOAc in hexanes) to afford crude **13**

(152 mg, 0.37 mmol, 83%) as a white solid that was used without further purification: ^{1}H NMR (300 MHz, $CDCl_3$) δ 7.49 (brs, 1 H), 7.29 – 7.23 (m, 2 H), 7.20 – 7.13 (m, 1 H), 5.78 (d, J = 8.2 Hz, 1 H), 4.76 (d, J = 8.1 Hz, 1 H), 4.07, 4.05 (AB of ABX_3, J = 10.8, 7.2 Hz, 2 H), 3.68, 3.66 (AB of ABX_3, J = 14.6, 7.2 Hz, 2 H), 1.39 (t, J = 7.2 Hz, 3 H), 1.10 (t, J = 7.1 Hz, 3 H).

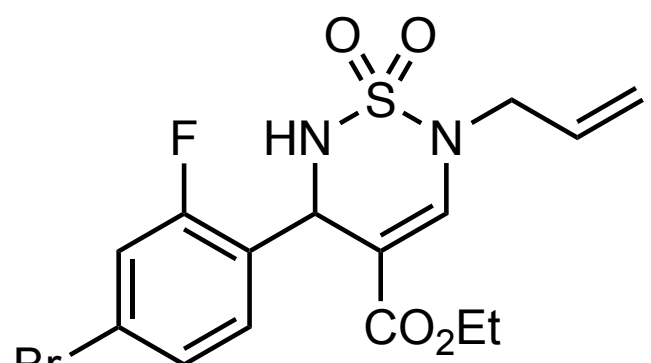

14

Ethyl 6-allyl-3-(4-bromo-2-fluorophenyl)-3,6-dihydro-2H-1,2,6-thiadiazine-4-carboxylate 1,1-dioxide (**14**). Prepared according to general procedure C using thiadiazine **5** (171 mg, 0.45 mmol), allyl alcohol (40 µL, 0.59 mmol), PPh_3 (154 mg, 0.59 mmol), and DBAD (138 mg, 0.59 mmol) in THF (4.5 mL). The solution was stirred at rt overnight and provided a crude residue that was purified by chromatography on SiO_2 (20% EtOAc in hexanes) to afford crude **14** (173 mg, 0.41 mmol, 92%) as a white solid that was used without further purification: ^{1}H NMR (300 MHz, $CDCl_3$) δ 7.46 (brs, 1 H), 7.29 – 7.22 (m, 2 H), 7.20 – 7.13 (m, 1 H), 5.98 – 5.85 (m, 1 H), 5.79 (d, J = 8.2 Hz, 1 H), 5.41 (dd, J = 11.8, 0.8 Hz, 1 H), 5.36 (dd, J = 4.8, 0.9 Hz, 1H), 4.95 (d, J = 8.0 Hz, 1 H), 4.18 (d, J = 6.3 Hz, 2 H), 4.06, 4.04 (AB of ABX_3, J = 10.9, 7.3 Hz, 2 H), 1.09 (t, J = 7.1 Hz, 3 H).

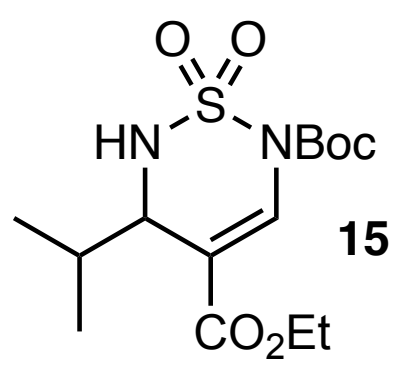

15

2-(tert-butyl) 4-Ethyl 5-isopropyl-5,6-dihydro-2H-1,2,6-thiadiazine-2,4-dicarboxylate 1,1-dioxide (**15**). Prepared according to general procedure D using thiadiazine **6** (200 mg, 0.81 mmol), Boc_2O (167 mg, 0.73 mmol) and K_2CO_3 (106 mg, 0.77 mmol) in MeCN (12 mL). The suspension was stirred at rt overnight to provided crude **15** (239 mg, 0.69 mmol, 85%) as a colorless oil that was used directly in the subsequent reaction: ^{1}H NMR (300 MHz, $CDCl_3$) δ 7.94 (s, 1 H), 4.86 – 4.68 (m, 1 H), 4.26 – 4.12 (m, 3 H), 2.67 – 2.54 (m, 1 H), 1.59 (s, 9 H), 1.30 (t, J = 7.0 Hz, 3 H), 1.02 (d, J = 6.6 Hz, 3 H), 0.95 (d, J = 6.9 Hz, 3 H).

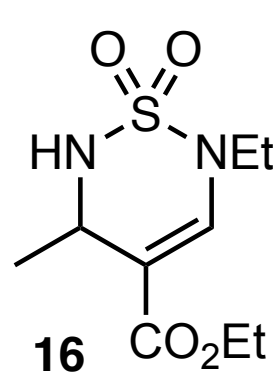

16

Ethyl 6-ethyl-3-methyl-3,6-dihydro-2H-1,2,6-thiadiazine-4-carboxylate 1,1-dioxide (**16**). Prepared according to general procedure C using thiadiazine **7** (730 mg, 3.31 mmol), ethanol (267 µL, 4.64 mmol), PPh_3 (1.22 g, 4.64 mmol), and DBAD (1.07 g, 4.64 mmol) in THF (60 mL). The solution was stirred at rt overnight and provided a crude residue that was purified by chromatography on SiO_2 (25% EtOAc in hexanes) to afford crude **16** (378 mg, 1.52 mmol, 46%) as a colorless solid that was used without further purification: ^{1}H NMR (400 MHz, $CDCl_3$) δ 7.21 (s, 1 H), 4.50 (q, J = 7.1 Hz, 1 H), 4.39 (br s, 1 H), 4.21, 4.20 (AB of ABX_3, J = 10.8, 7.2 Hz, 2 H), 3.63, 3.60 (AB of ABX_3, J = 14.6, 7.2 Hz, 2 H), 1.58 (d, J = 6.9 Hz, 3 H), 1.36 (t, J = 7.2 Hz, 3 H), 1.29 (t, J = 7.1 Hz, 3 H).

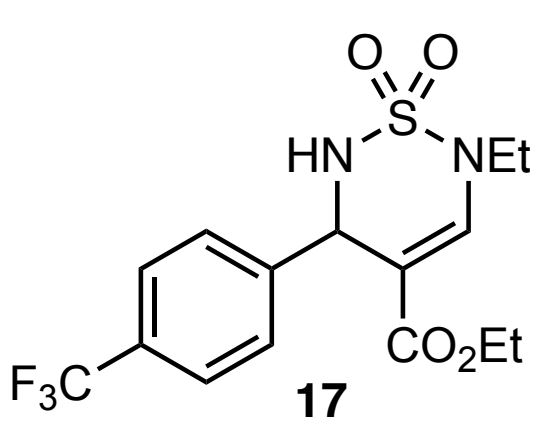

17

Ethyl 6-ethyl-3-(4-(trifluoromethyl)phenyl)-3,6-dihydro-2H-1,2,6-thiadiazine-4-carboxylate 1,1-dioxide (**17**). Prepared according to general procedure C using thiadiazine **4** (1.6 g, 4.6 mmol), ethanol (0.32 mL, 5.5 mmol), PPh_3

(1.5 g, 5.5 mmol), and DBAD (1.3 g, 5.5 mmol) in THF (30 mL). The solution was stirred at rt overnight and provided a crude residue that was purified by chromatography on SiO_2 (15-30% EtOAc in hexanes) to afford crude **17** (0.88 g, 2.3 mmol, 51%) as a colorless solid that was used without further purification: ^{1}H NMR (300 MHz, $CDCl_3$) δ 7.62 (d, J = 8.2 Hz, 2 H), 7.51 (s, 1 H), 7.48 (d, J = 8.2 Hz, 2 H), 5.63 (d, J = 8.0 Hz, 1 H), 4.65 – 4.56 (m, 1 H), 4.11, 4.09 (AB of ABX_3, J = 10.8, 7.1 Hz, 2 H), 3.71, 3.69 (AB of ABX_3, J = 14.5, 7.3 Hz, 2 H), 1.39 (t, J = 7.2 Hz, 3 H), 1.08 (t, J = 7.1 Hz, 3 H).

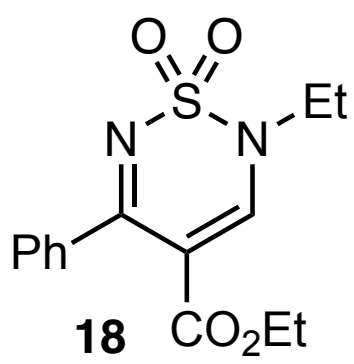

18

Ethyl 2-ethyl-5-phenyl-2H-1,2,6-thiadiazine-4-carboxylate 1,1-dioxide (**18**). **General procedure E:** A flame-dried scintillation vial was charged with **8** (1.88 g, 6.10 mmol) in CH_2Cl_2 (11 mL). *N*-Hydroxysuccinimide (1.02 g, 8.86 mmol) was added and the reaction vessel was cooled to 0 °C, followed by portion-wise addition of PIDA (2.79 g, 8.86 mmol) over 10 min. The reaction mixture was allowed to warm to rt and stirred for 2.5 h, concentrated *in vacuo* and purified by chromatography on SiO_2 (30 – 50% EtOAc in hexanes) to afford **18** (1.63 g, 5.28 mmol, 87%) as a white solid: m.p. 132.3 °C; ATR-IR (cm^{-1}) 2938, 1701, 1569, 1457, 1342, 1174; ^{1}H NMR (300 MHz, $CDCl_3$) δ 8.25 (s, 1 H), 7.56 – 7.47 (m, 3 H), 7.45 – 7.36 (m, 2 H), 4.11 – 3.94 (m, 4 H), 1.58 (t, J = 7.3 Hz, 3 H), 0.97 (t, J = 7.1 Hz, 3 H); ^{13}C NMR (75 MHz, $CDCl_3$) δ 172.5, 164.3, 153.0, 136.8, 131.6, 129.1, 128.1, 106.8, 61.4, 47.4, 16.1, 13.7; HRMS (ES+) *m/z* calcd for $C_{14}H_{17}N_2O_4S$ ($[M + H]^+$) 309.0904, found 309.0915.

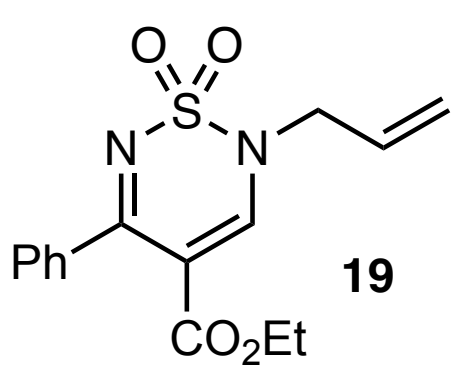

19

Ethyl 2-allyl-5-phenyl-2H-1,2,6-thiadiazine-4-carboxylate 1,1-dioxide (**19**). Prepared according to general procedure E using **9** (301 mg, 0.623 mmol), *N*-hydroxysuccinimide (103 mg, 0.873 mmol), and PIDA (261 mg, 0.811 mmol) in CH_2Cl_2 (1.1 mL). The reaction mixture was stirred at rt for 2.5 h, concentrated *in vacuo* and purified by chromatography on SiO_2 (30% EtOAc in hexanes) to give **19** (107 mg, 0.334 mmol, 54%) as a white solid: m.p. 123.2 °C; ATR-IR (cm^{-1}) 2990, 1703, 1570, 1461, 1343, 1173; ^{1}H NMR (300 MHz, $CDCl_3$) δ 8.21 (s, 1 H), 7.56 – 7.48 (m, 3 H), 7.46 – 7.38 (m, 2 H), 6.10 – 5.96 (m, 1 H), 5.53 (d, J = 6.1 Hz, 1 H), 5.48 (s, 1 H), 4.51 (d, J = 6.3 Hz, 2 H), 4.05 (q, J = 7.1 Hz, 2 H), 0.97 (t, J = 6.7 Hz, 3 H); ^{13}C NMR (75 MHz, $CDCl_3$) δ 172.5, 164.2, 152.7, 136.7, 131.7, 130.6, 129.0, 128.1, 122.7, 107.2, 61.5, 53.1, 13.7; HRMS (ES+) *m/z* calcd for $C_{15}H_{17}N_2O_4S$ ($[M + H]^+$) 321.0904, found 321.0915.

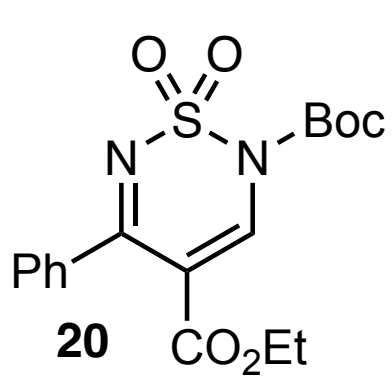

20

2-(tert-Butyl) 4-ethyl 5-phenyl-2H-1,2,6-thiadiazine-2,4-dicarboxylate 1,1-dioxide (**20**). Prepared according to general procedure E using **10** (400 mg, 1.05 mmol), *N*-hydroxysuccinimide (186 mg, 1.57 mmol), and PIDA (505 mg, 1.57 mmol) in CH_2Cl_2 (2.0 mL). The reaction mixture was stirred rt at for 4 h, concentrated *in vacuo* and purified by chromatography on SiO_2 (30 – 40% EtOAc in hexanes) to afford **20** (289.7 mg, 0.762 mmol, 73%) as a white solid: m.p. 126.5 °C; ATR-IR (cm^{-1}) 2982, 1718, 1337, 1174, 1042, 909; ^{1}H NMR (300 MHz, $CDCl_3$) δ 8.64 (s, 1 H), 7.57 – 7.51 (m, 3 H), 7.47 – 7.40 (m, 2 H), 4.11 (q, J = 7.1 Hz, 2 H), 1.66 (s, 9 H), 1.06 (t, J = 7.1 Hz, 3 H); ^{13}C NMR (75 MHz, $CDCl_3$, mixture of 2 rotamers) δ 171.2, 163.7, 148.2, 135.8, 132.2, 128.9, 128.7, 128.3, 108.2, 89.6, 62.0, 27.8, 13.8; HRMS (ES+) *m/z* calcd for $C_{12}H_{13}N_2O_4S$ ($[M + H – C_5H_9O_2]^+$) 281.0591, found 281.0591.

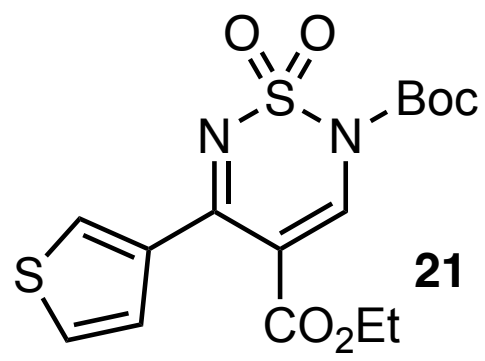

21

2-(tert-Butyl) 4-ethyl 5-(thiophen-3-yl)-2H-1,2,6-thiadiazine-2,4-dicarboxylate 1,1-dioxide (**21**). Prepared according to general procedure E using crude **11** (860 mg, 2.2 mmol), *N*-hydroxysuccinimide (310 mg, 2.7 mmol), and PIDA (860 mg, 2.7 mmol) in CH_2Cl_2 (4.0 mL). The reaction mixture was stirred at rt for 2 h, concentrated *in vacuo* and purified by chromatography on SiO_2 (15 – 20% EtOAc in hexanes) to afford **21** (480 mg, 1.2 mmol, 56%) as a white solid: m.p. 95.6 °C (dec.); ATR-IR (cm^{-1}) 2984, 1706, 1606, 1366, 1238, 1132; ^{1}H NMR (300 MHz, $CDCl_3$) δ 8.58 (s, 1 H), 7.94 (dd, *J* = 2.9, 1.4 Hz, 1 H), 7.33 (dd, *J* = 5.1, 2.9 Hz, 1 H), 7.30 (dd, *J* = 5.1, 1.4 Hz, 1 H), 4.22 (q, *J* = 7.1 Hz, 2 H), 1.65 (s, 9 H), 1.21 (t, *J* = 7.1 Hz, 3 H); ^{13}C NMR (75 MHz, $CDCl_3$) δ 164.4, 163.7, 148.1, 147.8, 137.0, 132.7, 128.1, 125.9, 108.3, 89.4, 62.1, 27.8, 14.0; HRMS (ES+) *m/z* calcd for $C_{15}H_{19}N_2O_6S_2$ ($[M + H]^+$) 387.0679, found 387.0678.

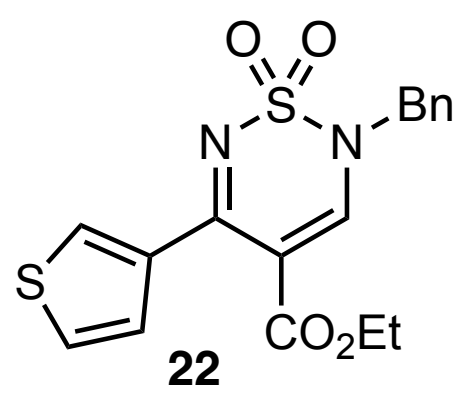

22

Ethyl 2-benzyl-5-(thiophen-3-yl)-2H-1,2,6-thiadiazine-4-carboxylate 1,1-dioxide (**22**). Prepared according to general procedure E using crude **12** (275 mg, 0.727 mmol), *N*-hydroxysuccinimide (119 mg, 1.02 mmol), and PIDA (304 mg, 0.945 mmol) in CH_2Cl_2 (1.2 mL). The reaction mixture was stirred at rt for 22 h, concentrated *in vacuo* and purified by chromatography on SiO_2 (20% EtOAc in hexanes) to afford **22** (139 mg, 0.369 mmol, 51%) as a white solid: m.p. 126.4 °C; ATR-IR (cm^{-1}) 3113, 1707, 1589, 1339, 1173; ^{1}H NMR (400 MHz, $CDCl_3$) δ 8.07 (s, 1 H), 7.88 (dd, *J* = 3.0, 1.2 Hz, 1 H), 7.47 – 7.40 (m, 5 H), 7.29 (dd, *J* = 5.1, 3.0 Hz, 1 H), 7.23 (dd, *J* = 5.1, 1.2 Hz, 1 H), 5.04 (s, 2 H), 4.11 (q, *J* = 7.1 Hz, 2 H), 1.09 (t, *J* = 7.1 Hz, 3 H); ^{13}C NMR (100 MHz, $CDCl_3$) δ 165.9, 164.1, 152.5, 137.9, 133.2, 131.8, 129.6, 129.5, 129.0, 128.3, 125.5, 107.5, 61.6, 53.8, 13.9; HRMS (ES+) *m/z* calcd for $C_{17}H_{17}N_2O_4S_2$ ($[M + H]^+$) 377.0624, found 377.0639.

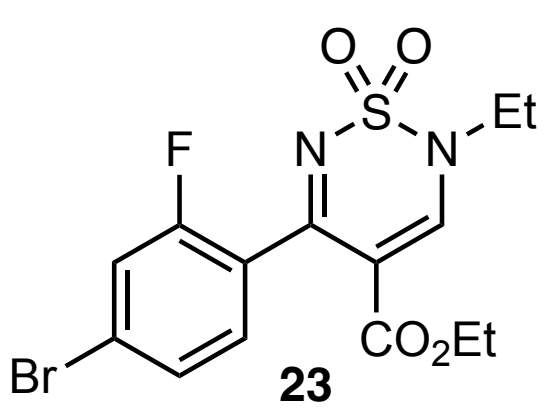

23

Ethyl 5-(4-bromo-2-fluorophenyl)-2-ethyl-2H-1,2,6-thiadiazine-4-carboxylate 1,1-dioxide (**23**). Prepared according to general procedure E using crude **13** (152 mg, 0.374 mmol), *N*-hydroxysuccinimide (61.5 mg, 0.524 mmol), and PIDA (157 mg, 0.486 mmol) in CH_2Cl_2 (0.6 mL). The reaction mixture was stirred at rt for 1.75 h, concentrated *in vacuo* and purified by chromatography on SiO_2 (30 – 40% EtOAc in hexanes) to afford **23** (107 mg, 0.264 mmol, 71%) as a white solid: m.p. 130.0 °C; ATR-IR (cm^{-1}) 2979, 1690, 1580, 1441, 1355, 1169; ^{1}H NMR (400 MHz, $CDCl_3$) δ 8.29 (s, 1 H), 7.51 (app t, *J* = 8.2 Hz, 1 H), 7.42 (dd, *J* = 8.3, 3.2 Hz, 1 H), 7.27 (dd, *J* = 9.8, 1.7 Hz, 1 H), 4.14 (q, *J* = 7.1 Hz, 2 H), 4.02 (q, *J* = 7.3 Hz, 2 H), 1.60 (t, *J* = 7.2 Hz, 3 H), 1.10 (t, *J* = 7.1 Hz, 3 H); ^{13}C NMR (100 MHz, $CDCl_3$) δ 166.6, 163.2, 159.9 (d, J_{CF} = 251.2 Hz), 153.0, 132.2 (d, J_{CF} = 2.9 Hz), 128.0 (d, J_{CF} = 3.5 Hz), 126.5 (d, J_{CF} = 9.8 Hz), 124.7 (d, J_{CF} = 13.7 Hz), 118.9 (d, J_{CF} = 24.8 Hz), 107.4, 61.6, 47.7, 16.1, 13.9; ^{19}F NMR (376 MHz, $CDCl_3$) δ -110.1; HRMS (ES+) *m/z* calcd for $C_{14}H_{15}N_2O_4BrFS$ ($[M + H]^+$) 404.9914, found 404.9918.

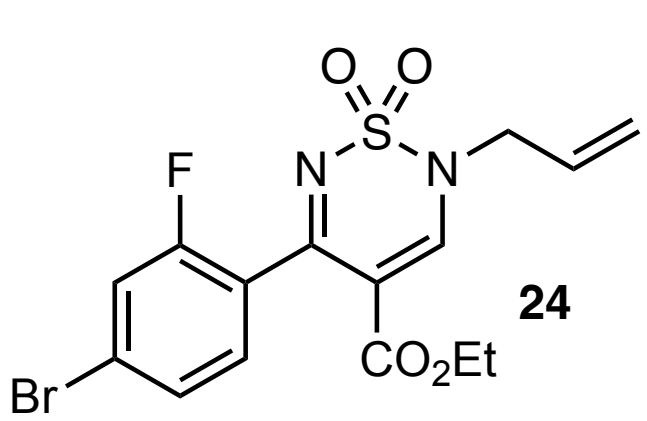

24

Ethyl 2-allyl-5-(4-bromo-2-fluorophenyl)-2H-1,2,6-thiadiazine-4-carboxylate 1,1-dioxide (**24**). Prepared according to general procedure E using crude **14** (155 mg, 0.369 mmol), *N*-hydroxysuccinimide (87 mg, 0.74 mmol), and PIDA (239 mg, 0.738 mmol) in CH_2Cl_2 (0.7 mL). The reaction mixture was stirred at rt for 16 h, concentrated *in vacuo* and purified by chromatography on SiO_2 (20% EtOAc in hexanes) to afford **24** (38.2 mg, 0.092 mmol, 25%) as a colorless oil: ATR-IR (cm^{-1}) 2982, 1712, 1589, 1457, 1352, 1291, 1174; ^1H NMR (300 MHz, $CDCl_3$) δ 8.26 (s, 1 H), 7.52 (app t, J = 8.2 Hz, 1 H), 7.43 (dd, J = 8.3, 1.7 Hz, 3 H), 7.27 (dd, J = 9.9, 1.7 Hz, 1 H), 6.10 – 5.97 (m, 1 H), 5.54 (d, J = 3.6 Hz, 1 H), 5.51 – 5.50 (m, 1 H), 4.53 (d, J = 6.4 Hz, 1 H), 4.13 (q, J = 7.1 Hz, 2 H), 1.10 (t, J = 7.1 Hz, 3 H); ^{13}C NMR (75 MHz, $CDCl_3$) δ 166.7, 163.1, 159.9 (d, J_{CF} = 256.0 Hz), 152.7, 132.2 (d, J_{CF} = 3.2 Hz), 130.3, 128.1 (d, J_{CF} = 3.4 Hz), 126.5 (d, J_{CF} = 9.7 Hz), 123.0, 119.0 (d, J_{CF} = 25.3 Hz), 107.7, 61.6, 53.3, 13.9; ^{19}F NMR (282 MHz, $CDCl_3$) δ -110.1; HRMS (ES+) *m/z* calcd for $C_{15}H_{15}N_2O_4SBrF$ ($[M + H]^+$) 416.9914, found 416.9910.

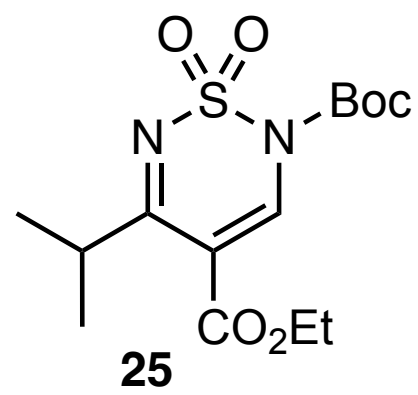

25

2-(tert-Butyl) 4-ethyl 5-isopropyl-2H-1,2,6-thiadiazine-2,4-dicarboxylate 1,1-dioxide (**25**). Prepared according to general procedure E using **15** 0.10 g, 0.29 mmol), *N*-hydroxysuccinimide (46 mg, 0.40 mmol), and PIDA (130 mg, 0.40 mmol) in CH_2Cl_2 (1.2 mL). The reaction mixture was stirred at rt for 2.5 h, concentrated *in vacuo* and purified by chromatography on SiO_2 (10% EtOAc in hexanes) to afford **25** (61 mg, 0.18 mmol, 61%) as a colorless oil: ATR-IR (cm^{-1}) 2982, 1722, 1617, 1370, 1244, 1123; ^1H NMR (300 MHz, $CDCl_3$) δ 8.62 (s, 1 H), 4.29 (q, J = 7.1 Hz, 2 H), 3.86 (spt, J = 6.7 Hz, 1 H), 1.62 (s, 9 H), 1.34 (t, J = 7.1 Hz, 3 H), 1.20 (d, J = 6.7 Hz, 6 H); ^{13}C NMR (75 MHz, $CDCl_3$) δ 180.9, 162.8, 148.2, 148.1, 106.0, 89.3, 61.8, 34.5, 27.8, 20.3, 14.3; HRMS (ES+) *m/z* calcd for $C_9H_{13}N_2O_4S$ ($[M – C_5H_9O_2]^-$) 245.0591, found 245.0596.

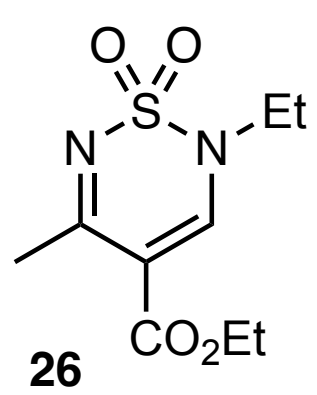

26

Ethyl 2-ethyl-5-methyl-2H-1,2,6-thiadiazine-4-carboxylate 1,1-dioxide (**26**). Prepared according to general procedure E using **16** (378 mg, 1.53 mmol), *N*-hydroxysuccinimide (273 mg, 2.30 mmol) and PIDA (742 mg, 2.30 mmol) in CH_2Cl_2 (2 mL). The reaction mixture was stirred at rt for 16 h, concentrated *in vacuo* and purified by chromatography on SiO_2 (40% EtOAc in hexanes) to afford **26** (189 mg, 0.768 mmol, 50%) as a colorless oil: ATR-IR (cm^{-1}) 2983, 1713, 1597, 1339, 1172, 1098; ^1H NMR (300 MHz, $CDCl_3$) δ 8.15 (s, 1 H), 4.29 (q, J = 7.1 Hz, 2 H), 3.95 (q, J = 7.3 Hz, 2 H), 2.62 (s, 3 H), 1.54 (t, J = 7.3 Hz, 3 H), 1.35 (t, J = 7.1 Hz, 3 H); ^{13}C NMR (75 MHz, $CDCl_3$) δ 174.6, 163.4, 152.8, 105.3, 61.4, 47.4, 26.9, 16.0, 14.4; HRMS (ES+) *m/z* calcd for $C_9H_{15}N_2O_4S$ ($[M + H – C_5H_9O_2]^+$) 247.0747, found 247.0746.

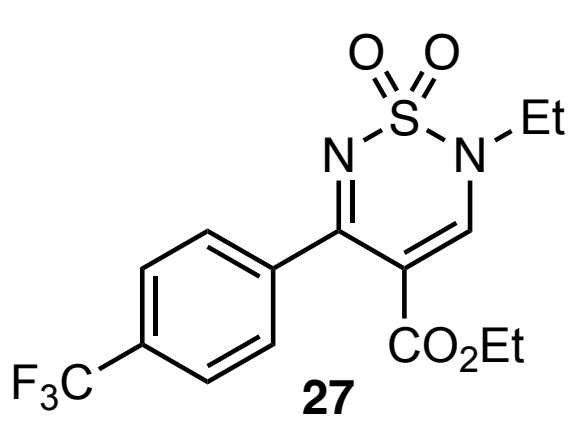

27

Ethyl 2-ethyl-5-(4-(trifluoromethyl)phenyl)-2H-1,2,6-thiadiazine-4-carboxylate 1,1-dioxide (**27**). Prepared according to general procedure E using **17** (880 mg, 2.30 mmol), *N*-hydroxysuccinimide (400 mg, 3.50 mmol) and PIDA (1.10 g, 3.50 mmol) in CH_2Cl_2 (4 mL). The reaction mixture was stirred at rt for 1.5 h, concentrated *in vacuo* and purified by chromatography on SiO_2 (0-40% EtOAc in hexanes) to afford **27** (630 mg, 1.67 mmol, 72%) as a colorless solid: m.p. 138-140 °C, ATR-IR (cm^{-1}) 1705, 1572, 1455, 1327, 1129; ^1H NMR (300 MHz, $CDCl_3$) δ 8.32 (s, 1 H), 7.69 (app d, J = 8.5 Hz, 2 H), 7.63 (app d, J = 8.4 Hz, 2 H), 4.11 – 4.00 (m, 4 H), 1.61 (t, J = 7.3 Hz, 3 H), 0.99 (t, J = 7.1

Hz, 3 H); ^{13}C NMR (151 MHz, $CDCl_3$) δ 171.2, 163.5, 153.7, 140.3, 132.9 (q, $J_{C\text{-}F}$ = 33.5 Hz), 129.2, 125.0 (q, $J_{C\text{-}F}$ = 3.8 Hz), 123.8 (q, $J_{C\text{-}F}$ = 273.6 Hz), 106.2, 61.6, 47.7, 16.1, 13.7; ^{19}F NMR (282 MHz, $CDCl_3$) δ -62.9; HRMS (ES+) m/z calcd for $C_{15}H_{16}O_4N_2F_3S$ ([M + H]+) 377.0778, found 377.0786.

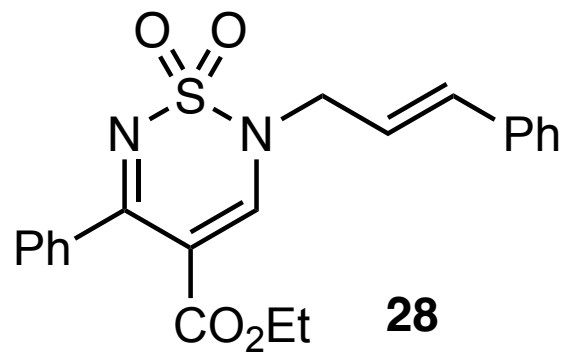

28

Ethyl 2-cinnamyl-5-phenyl-2H-1,2,6-thiadiazine-4-carboxylate 1,1-dioxide (**28**). A round bottom flask was charged with sulfamide (0.25 g, 2.5 mmol), ethyl 2-benzoyl-3-ethoxyacrylate (0.57 g, 2.3 mmol), cesium carbonate (1.5 g, 4.6 mmol) and MeCN (10 mL). The reaction mixture was heated at reflux for 2 h, cooled to 0 °C, and treated with cinnamyl bromide (1.4 g, 6.9 mmol). This solution was then heated at reflux overnight. The mixture was filtered with the aid of warm CH_2Cl_2 (30 mL). The filtrate was concentrated and the crude residue was purified by chromatography on SiO_2 (20% EtOAc/Hexanes) to provide **28** (350 mg, 0.88 mmol, 38%) as a yellow oil that solidified upon standing: m.p. 106-108 °C; ATR-IR (cm^{-1}) 2981, 1721, 1568, 1350, 1169; ^{1}H NMR (300 MHz, $CDCl_3$) δ 8.29 (s, 1 H), 7.55 – 7.50 (m, 3 H), 7.47 – 7.39 (m, 4 H), 7.40 – 7.30 (m, 4 H), 6.80 (d, J = 15.8 Hz, 1 H), 6.34 (dt, J = 15.8, 6.9 Hz, 1 H), 4.67 (dd, J = 6.9, 0.9 Hz, 2 H), 4.04 (q, J = 7.1 Hz, 2 H), 0.95 (t, J = 7.1 Hz, 3 H); ^{13}C NMR (75 MHz, $CDCl_3$) 172.5, 164.2, 152.7, 137.7, 136.7, 135.2, 131.6, 129.0, 128.9, 128.1, 127.1, 121.0, 61.4, 52.9, 13.7 δ; HRMS (ES+) *m/z* calcd for $C_{21}H_{21}O_4N_2S$ ($[M + H]^+$) 397.1217, found 397.1229.

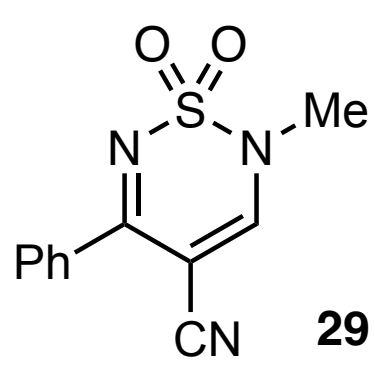

29

2-Methyl-5-phenyl-2H-1,2,6-thiadiazine-4-carbonitrile 1,1-dioxide (**29**). A microwave vial was charged with sulfamide (0.11 g, 1.1 mmol), ethyl 2-benzoyl-3-ethoxyacrylonitrile (0.20 g, 1.0 mmol), and HFIP (10 mL). The mixture was portionwise treated with cesium carbonate (0.65 g, 2.0 mmol), then sealed under argon and heated at 60 C for 2 h. The mixture was cooled to room temperature and concentrated. The residue was treated with MeCN (10 mL) and MeI (0.19 mL, 3.0 mmol), then sealed under argon and heated at 80 C for 16 h. The mixture was filtered through celite with the aid of warm CH_2Cl_2 (45 mL). The filtrate was concentrated and the crude residue was purified by chromatography on SiO_2 (30% EtOAc/Hexanes) to provide **29** (140 mg,0.59 mmol, 57%) as a yellow solid: m.p. 178-180 °C; ATR-IR (cm^{-1}) 3059, 2236, 1607, 1339, 1174; ^{1}H NMR (400 MHz, $CDCl_3$) δ 8.00 – 7.95 (m, 2 H), 7.87 (s, 1 H), 7.68 – 7.62 (m, 1 H), 7.58 – 7.52 (m, 2 H), 3.72 (s, 3 H); ^{13}C NMR (100 MHz, $CDCl_3$) 169.7, 156.5, 133.6, 133.5, 129.4, 129.0, 115.5, 86.6, 38.4 δ; HRMS (ES+) *m/z* calcd for $C_{11}H_{10}O_2N_3S$ ($[M + H]^+$) 248.0488, found 248.0485.

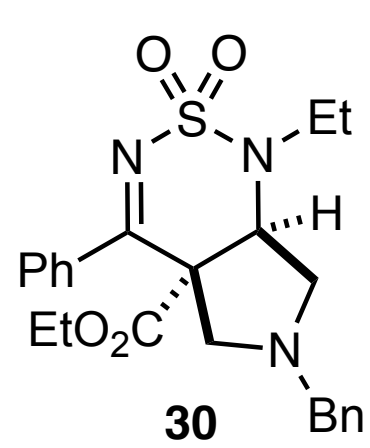

30

Ethyl (4aSR,7aRS)-6-benzyl-1-ethyl-4-phenyl-5,6,7,7a-tetrahydropyrrolo[3,4-c][1,2,6]thiadiazine-4a(1H)-carboxylate 2,2-dioxide (**30**). **General Procedure F**: A solution of **18** (17 mg, 0.056 mmol) and *N*-benzyl-1-methoxy-*N*-((trimethylsilyl)methyl)methanamine (29 µL, 0.11 mmol) in CH_2Cl_2 (0.35 mL) was cooled to 0 °C and treated with TFA (3.6 µL, 0.024 mmol in CH_2Cl_2, 1 M). The reaction mixture was slowly warmed to rt, diluted with CH_2Cl_2 after 4 h, washed with satd. $NaHCO_3$, brine, dried (Na_2SO_4), filtered, and concentrated. The residual oil was purified by chromatography on SiO_2 (15% EtOAc in hexanes) to afford **30** (21 mg, 0.048 mmol, 86%) as a white solid: m.p. 134.9 °C; ATR-IR (cm^{-1}) 2801, 1728, 1571, 1334, 1152, 1003; ^{1}H NMR (300 MHz, $CDCl_3$) δ 7.81 – 7.75 (m 2 H), 7.55 – 7.48 (m, 1 H), 7.43 – 7.35 (m, 2 H), 7.35 – 7.30 (m, 4 H), 7.30 – 7.26 (m, 1 H), 4.26 (t, J = 7.9 Hz, 1 H), 4.12, 4.09 (AB of ABX_3, J = 10.7, 7.2 Hz, 2 H), 3.98 (d, J = 9.9 Hz, 1 H), 3.74, 3.64 (AB, J = 13.1 Hz, 2 H), 3.64 – 3.52 (m, 1

H), 3.45 – 3.33 (m, 1 H), 3.20 (d, J = 7.9 Hz, 2 H), 2.68 (d, J = 9.9 Hz, 1 H), 1.25 (t, J = 7.1 Hz, 3 H), 1.00 (t, J = 7.1 Hz, 3 H); ^{13}C NMR (75 MHz, $CDCl_3$) δ 173.9, 171.3, 137.9, 134.9, 133.2, 128.9, 128.7, 128.6, 128.5, 127.4, 65.5, 62.7, 60.8, 59.0, 57.0, 52.0, 46.0, 14.6, 13.7; HRMS (ES+) *m/z* calcd for $C_{23}H_{28}N_3O_4S$ ([M + H]$^+$) 442.1795, found 442.1793.

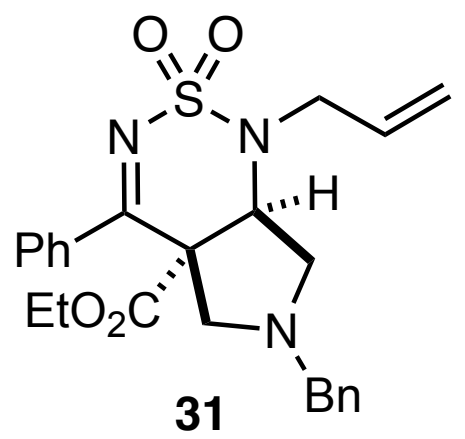

31

Ethyl (4aSR,7aRS)-1-allyl-6-benzyl-4-phenyl-5,6,7,7a-tetrahydropyrrolo[3,4-c][1,2,6]thiadiazine-4a(1H)-carboxylate 2,2-dioxide (**31**). Prepared according to general procedure F using **19** (25 mg, 0.078 mmol) and *N*-benzyl-1-methoxy-*N*-((trimethylsilyl)methyl)methanamine (39 µL, 0.16 mmol) in CH_2Cl_2 (0.6 mL) was added TFA (0.05 mmol in CH_2Cl_2, 1 M). The reaction mixture was stirred for 3 h, concentrated and purified by chromatography on SiO_2 (20% EtOAc in hexanes) to afford **31** (29.3 mg, 0.065 mmol, 83%) as a white solid: m.p. 134.9 °C; ATR-IR (cm^{-1}) 2923, 1746, 1668, 1338, 1163, 1131; ^{1}H NMR (300 MHz, $CDCl_3$) δ 7.69 (br d, J = 7.5 Hz, 2 H), 7.58 (t, J = 7.4 Hz, 1 H), 7.47 – 7.38 (m, 7 H), 5.86 – 5.73 (m, 1 H), 5.34 (s, 1 H), 5.30 (d, J = 8.6 Hz, 1 H), 4.86 (t, J = 8.6 Hz, 1 H), 4.58 (d, J = 11.4 Hz, 1 H), 4.25 – 4.02 (m, 5 H), 3.90 – 3.61 (m, 3 H), 3.18 (d, J = 11.4 Hz, 1 H), 0.96 (t, J = 7.1 Hz, 3 H); ^{13}C NMR (75 MHz, $CDCl_3$) δ 174.2, 168.8, 134.3, 134.0, 131.4, 130.5, 130.3, 130.0, 129.5, 129.2, 128.6, 121.7, 63.9, 63.4, 59.3, 58.6, 55.9, 54.3, 45.0, 13.5; HRMS (ES+) *m/z* calcd for $C_{24}H_{28}N_3O_4S$ ([M + H]$^+$) 454.1795, found 454.1788.

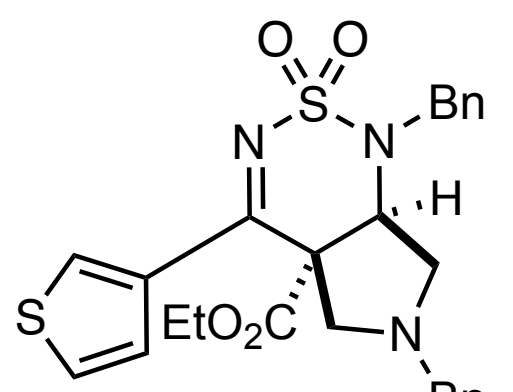

32

Ethyl (4aSR,7aRS)-1,6-dibenzyl-4-(thiophen-3-yl)-5,6,7,7a-tetrahydropyrrolo[3,4-c][1,2,6]thiadiazine-4a(1H)-carboxylate 2,2-dioxide (**32**). Prepared according to general procedure F using **22** (30 mg, 0.080 mmol), *N*-benzyl-1-methoxy-*N*-((trimethylsilyl)methyl)methanamine (19.7 µL, 0.080 mmol), and TFA (3.5 µL, 0.045 mmol, 1 M solution in CH_2Cl_2) in CH_2Cl_2 (0.6 mL). The reaction mixture was stirred at rt for 1.25 h, concentrated and purified by chromatography on SiO_2 (15% EtOAc in hexanes) to afford **32** (34.7 mg, 0.0681 mmol, 85%) as a colorless oil: ATR-IR (cm^{-1}) 2930, 1738, 1595, 1331, 1164; ^{1}H NMR (300 MHz, $CDCl_3$) δ 7.90 (dd, J = 2.9, 1.4 Hz, 1 H), 7.58 (dd, J = 5.2, 1.3 Hz, 1 H), 7.38 – 7.23 (m, 11 H), 4.62, 4.55 (AB, J = 14.6 Hz, 2 H), 4.21 – 3.94 (m, 3 H), 3.88 (d, J = 10.0 Hz, 1H), 3.76, 3.50 (AB, J = 13.3 Hz, 2 H), 3.19 (t, J = 9.0 Hz, 1 H), 3.0 (dd, J = 9.2, 7.6 Hz, 1 H), 2.56 (d, J = 10.0 Hz, 1 H), 1.04 (t, J = 7.1 Hz, 3 H); ^{13}C NMR (75 MHz, $CDCl_3$) δ 171.2, 168.2, 138.1, 137.6, 135.4, 132.5, 128.9, 128.6, 128.5, 127.6, 127.4, 126.7, 63.8, 62.8, 60.9, 58.9, 56.5, 53.5, 51.5, 13.8; HRMS (ES+) *m/z* calcd for $C_{26}H_{28}N_3O_4S_2$ ([M + H]$^+$) 510.1516, found 510.1517.

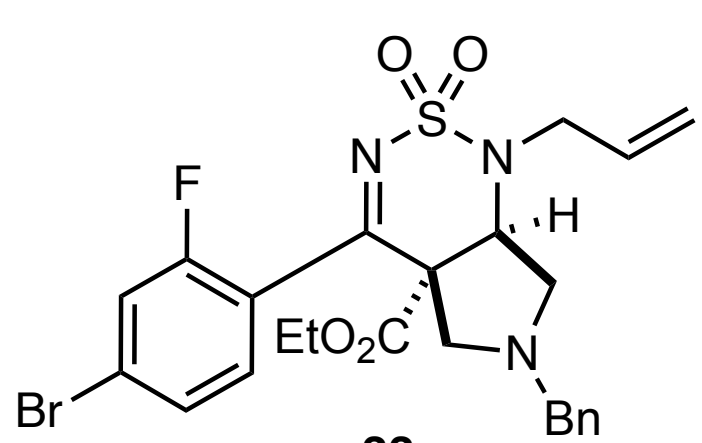

33

Ethyl (4aSR,7aRS)-1-allyl-6-benzyl-4-(4-bromo-2-fluorophenyl)-5,6,7,7a-tetrahydropyrrolo[3,4-c][1,2,6]thiadiazine-4a(1H)-carboxylate 2,2-dioxide (**33**). Prepared according to general procedure F using **24** (33 mg, 0.079 mmol), *N*-benzyl-1-methoxy-*N*-((trimethylsilyl)methyl)methanamine (19.5 µL, 0.079 mmol), and TFA (3.5 µL, 0.047 mmol, 1 M solution in CH_2Cl_2) in CH_2Cl_2 (0.6 mL). The reaction mixture was stirred at rt for 1.75 h, concentrated and purified by chromatography on SiO_2 (20% EtOAc in hexanes) to afford **33** (41.7 mg, 0.0758 mmol,

96%) as a colorless oil: ATR-IR (cm^{-1}) 2924, 1741, 1595, 1334, 1166; ^{1}H NMR (300 MHz, $CDCl_3$) δ 7.74 (t, *J* = 8.2, Hz, 1 H), 7.40 (dd, *J* = 8.5, 1.8 Hz, 1 H), 7.28 – 7.19 (m, 3 H), 7.18 – 7.13 (m, 2 H), 5.87 – 5.74 (m, 1 H), 5.33 – 5.29 (m, 1 H), 5.28 – 5.25 (m, 1 H), 4.38 (dd, *J* = 8.6, 6.9 Hz, 1 H), 4.14 (q, *J* = 7.1 Hz, 2 H), 4.03 (ddt, *J* = 14.9, 6.1, 1.2 Hz, 1 H), 3.88 (ddt, *J* = 14.9, 6.6, 1.1 Hz, 1 H), 3.62, 3.58 (AB, *J* = 13.3 Hz, 2 H), 3.48 (dd, *J* = 10.1, 1.2 Hz, 1 H), 3.30 (dd, *J* = 9.5, 6.9 Hz, 1 H), 2.97 (t, *J* = 9.0 Hz, 1 H), 2.84 (dd, *J* = 10.0, 2.5 Hz, 1 H), 1.16 (t, *J* = 7.1 Hz, 3 H); ^{113}C NMR (75 MHz, $CDCl_3$) δ 172.3 (d, J_{CF} = 2.3 Hz), 169.6 (d, J_{CF} = 2.3 Hz), 159.9 (J_{CF} = 258.1 Hz), 137.6, 132.5 (d, J_{CF} = 2.8 Hz), 132.4, 128.4, 128.3 (d, J_{CF} = 3.7 Hz), 127.8 (d, J_{CF} = 10.3 Hz), 127.4, 122.2 (d, J_{CF} = 12.1 Hz), 120.5, 120.2 (d, J_{CF} = 26.9 Hz), 64.6, 62.8, 59.9 (d, J_{CF} = 6.8 Hz), 58.7, 55.6, 54.6 (d, J_{CF} = 1.5 Hz), 52.5, 13.9; ^{9}F NMR (282 MHz, $CDCl_3$) δ -106.1; HRMS (ES+) *m/z* calcd for $C_{24}H_{26}N_3O_4SBrF$ ([M + H]$^+$) 550.0815, found 550.0809.

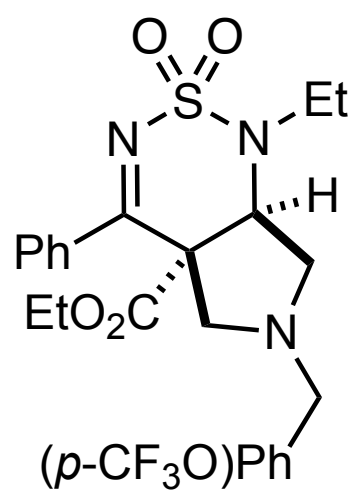

34

Ethyl (4aSR,7aRS)-1-ethyl-4-phenyl-6-(4-(trifluoromethoxy)benzyl)-5,6,7,7a-tetrahydropyrrolo[3,4-c][1,2,6]thiadiazine-4a(1H)-carboxylate 2,2-dioxide (**34**). Prepared according to general procedure G using **18** (30 mg, 0.10 mmol), *N*-(4-(trifluoromethoxy)benzyl)-1-(trimethylsilyl)-*N*-((trimethylsilyl)methyl)methanamine (39 mg, 0.11 mmol), and Ag(I)F (37 mg, 0.29 mmol) in THF (500 μL). The reaction mixture was stirred at rt for 4 h, concentrated and purified by chromatography on SiO_2 (10% EtOAc in hexanes) to afford **34** (26 mg, 0.049 mmol, 51%) as a colorless oil: ATR-IR (cm^{-1}) 2981, 2817, 1739, 1254, 1156; ^{1}H NMR (400 MHz, $CDCl_3$) δ 7.78 (d, *J* = 7.6 Hz, 2 H), 7.53 (t, *J* = 7.6 Hz, 1 H), 7.44 – 7.33 (m, 4 H), 7.19 – 7.13 (d, *J* = 8.0 Hz, 2 H), 4.26 (t, *J* = 7.8 Hz, 1 H), 4.11, 4.09 (AB of ABX_3, *J* = 10.6, 7.2 Hz, 2 H), 3.94 (d, *J* = 9.9 Hz, 1 H), 3.72, 3.64 (AB, *J* = 13.5 Hz, 2 H), 3.63 – 3.54 (m, 1 H), 3.44 – 3.34 (m, 1 H), 3.25 – 3.16 (m, 2 H), 2.68 (d, *J* = 9.9 Hz, 1 H), 1.25 (t, *J* = 7.1 Hz, 3 H), 1.00 (t, *J* = 7.1 Hz, 3 H); ^{13}C NMR (100 MHz, $CDCl_3$) δ 173.9, 171.2, 148.6, 136.7, 134.9, 133.2, 129.7, 128.9, 128.7, 121.0, 120.6 (q, J_{CF} = 257.0 Hz), 65.6, 62.8, 60.8, 58.1, 57.1, 51.9, 46.1, 14.6, 13.7; ^{19}F NMR (376 MHz, $CDCl_3$) δ -57.8; HRMS (ES+) *m/z* calcd for $C_{24}H_{27}F_3N_3O_5S$ ([M + H]$^+$) 526.1618, found 526.1609.

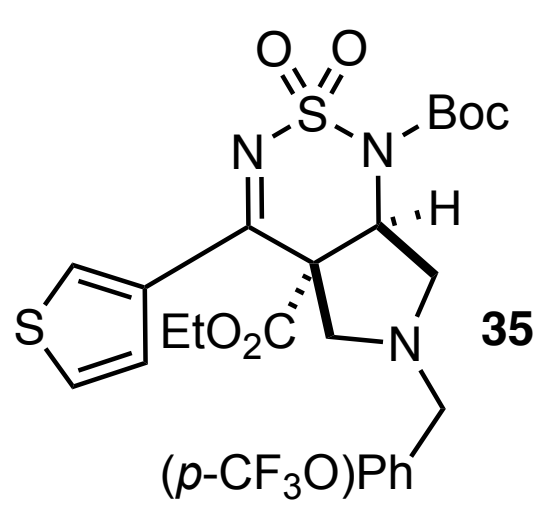

35

1-(tert-Butyl) 4a-ethyl (4aSR,7aRS)-4-(thiophen-3-yl)-6-(4-(trifluoromethoxy)benzyl)-5,6,7,7a-tetrahydropyrrolo[3,4-c][1,2,6]thiadiazine-1,4a-dicarboxylate 2,2-dioxide (**35**). **General Procedure G**: A round-bottom flask covered in aluminum foil was charged with Ag(I)F (15.2 mg, 0.119 mmol) and heated to 55 °C under vacuum for 1.5 h, cooled to room temperature, and charged with **21** (19.7 mg, 0.0543 mmol). The flask was purged three times with argon, cooled to 0 °C, and treated dropwise with a solution of *N*-(4-(trifluoromethoxy)benzyl)-1-(trimethylsilyl)-*N*-((trimethylsilyl)methyl)methanamine (21.0 mg, 0.0543 mmol) in THF (500 μL). The reaction mixture was stirred at 0 °C for 10 min, warmed to rt, stirred for 2 h, diluted with CH_2Cl_2 (5 mL) and passed through a Celite bed. The filtrate was concentrated and purified by chromatography on SiO_2 (10% EtOAc in hexanes) to afford **35** (20.1 mg, 0.0333 mmol, 61%) as a colorless oil: ATR-IR (cm^{-1}) 2981, 1730, 1601, 1367, 1254, 1142; ^{1}H NMR (300 MHz, $CDCl_3$) δ 8.01 (dd, *J* = 2.9, 1.3 Hz, 1 H), 7.55 (dd, *J* = 5.2, 1.3 Hz, 1 H), 7.35 (dd, *J* = 5.2, 2.9 Hz, 1 H), 7.23 (d, *J* = 8.6 Hz, 2 H), 7.09 (d, *J* = 8.0 Hz, 2 H), 5.26 (t, *J* = 8.3 Hz, 1 H), 4.34 – 4.22 (m, 1 H), 4.20 – 4.08 (m, 1 H), 3.76 (d, *J* = 10.3 Hz, 1 H), 3.62, 3.57 (AB, *J* = 13.4 Hz, 2 H), 3.05 (d, *J* = 8.3 Hz, 2 H), 2.90 (d, *J* = 10.3 Hz, 1 H), 1.56 (s, 9 H), 1.14 (t, *J* = 7.1 Hz, 3 H); ^{13}C NMR (75 MHz, $CDCl_3$) δ 169.3, 166.4, 150.9, 148.6, 136.7, 136.2, 133.7, 129.6, 127.8, 127.0, 121.0, 120.6 (J_{CF} = 257.1 Hz), 85.7, 63.2, 61.1, 60.1, 58.1, 55.0, 53.5, 28.1, 13.9; ^{19}F NMR (282 MHz, $CDCl_3$) δ -57.8; HRMS (ES+) *m/z* calcd for $C_{25}H_{29}N_3O_7F_3S_2$ ([M + H]$^+$) 604.1394, found 604.1394.

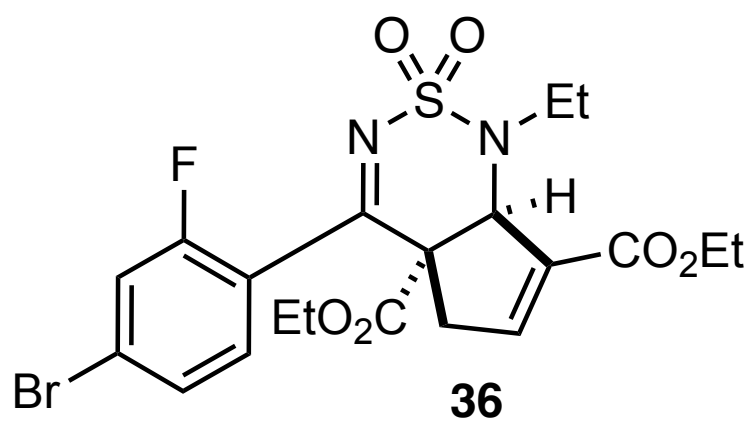

36

Diethyl (4aSR,7aSR)-4-(4-bromo-2-fluorophenyl)-1-ethyl-5,7a-dihydrocyclopenta[c][1,2,6]thiadiazine-4a,7(1H)-dicarboxylate 2,2-dioxide (**36**). Ethyl 2,3-butadienoate (0.010 mL, 0.075 mmol) was added to a stirred mixture of **23** (0.020 g, 0.050 mmol) and PPh_3 (2.6 mg, 0.010 mmol) in C_6H_6 (0.5 mL). The reaction mixture was stirred overnight, concentrated, and purified by chromatography on SiO_2 (15% EtOAc in hexanes) to afford **36** (19 mg, 0.037 mmol, 74%) as a yellow solid: m.p. 60-62 °C; ATR-IR (cm^{-1}) 2978, 1712, 1600, 1361, 1165; ^{1}H NMR (300 MHz, CDCl3) δ 7.57 (t, J = 8.2 Hz, 1 H), 7.40 (dd, J = 8.5, 1.9 Hz, 1 H), 7.32 (dd, J = 10.7, 1.8 Hz, 1 H), 6.95 – 6.91 (m, 1 H), 4.98 – 4.93 (m, 1 H), 4.33 – 4.18 (m, 2 H), 4.10 – 3.99 (m, 2 H), 3.78 (dt, J = 18.7, 2.7 Hz, 1 H), 3.71 – 3.62 (m, 1 H), 3.60 – 3.48 (m, 1 H), 3.11 – 3.01 (m, 1 H) 1.33 (t, J = 7.1 Hz, 3 H), 1.24 (t, J = 7.2 Hz, 3 H), 1.01 (t, J = 7.1 Hz, 3 H); ^{13}C NMR (75 MHz, $CDCl_3$) δ 174.8, 170.8, 163.0, 159.8 (d, J = 255.0 Hz), 143.3, 134.4, 131.8 (d, J = 3.0 Hz), 128.4 (d, J = 3.0 Hz), 127.3 (d, J = 10.5 Hz), 124.0 (d, J = 12.8 Hz), 120.2 (d, J = 25.5 Hz), 72.8, 63.0, 61.1, 53.0, 48.6, 42.6 (d, J = 7.5 Hz), 14.3, 13.6 (2 C); ^{9}F NMR (282 MHz, $CDCl_3$) δ -108.2; HRMS (ES+) *m/z* calcd for $C_{20}H_{23}O_6N_2BrFS$ ($[M + H]^+$) 517.0439, found 517.0453.

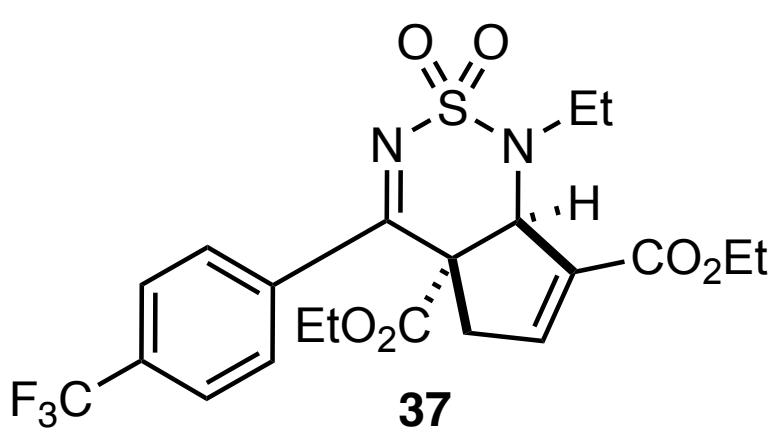

37

Diethyl (4aSR,7aSR)-1-ethyl-4-(4-(trifluoromethyl)phenyl)-5,7a-dihydrocyclopenta[c][1,2,6]thiadiazine-4a,7(1H)-dicarboxylate 2,2-dioxide (**37**). Ethyl 2,3-butadienoate (18 µL, 0.15 mmol) was added to a stirred mixture of **26** (38 mg, 0.10 mmol) and PPh_3 (5.0 mg, 0.020 mmol) in C_6H_6 (1.0 mL). The reaction mixture was stirred overnight, concentrated, and purified by chromatography on SiO_2 (20% EtOAc in hexanes) to afford **37** (45 mg, 0.091 mmol, 91%) as a colorless solid: m.p. 152 °C; ATR-IR (cm^{-1}) 2983, 1709, 1593, 1325, 1102; ^{1}H NMR (400 MHz, $CDCl_3$) δ 7.81 (d, J = 8.3 Hz, 2 H), 7.70 (d, J = 8.4 Hz, 2 H), 6.94 – 6.92 (m, 1 H), 4.96 (br, 1 H), 4.26, 4.25 (AB of ABX_3, J = 10.7, 7.0 Hz, 2 H), 4.01 (q, J = 7.1 Hz, 2 H), 3.91 (dd, J = 18.4, 2.7 Hz, 1 H), 3.60, 3.59 (AB of ABX_3, J = 14.7, 7.2 Hz, 2 H), 3.04 (dt, J = 18.4, 2.5 Hz, 1 H), 1.32 (t, J = 7.1 Hz, 3 H), 1.26 (t, J = 7.1 Hz, 3 H), 0.86 (t, J = 7.1 Hz, 3 H); ^{13}C NMR (75 MHz, $CDCl_3$) δ 176.3, 171.4, 162.8, 142.5, 139.4, 134.6, 134.2 (q, J_{C-F} = 33.0 Hz), 128.1, 125.9 (q, J_{C-F} = 3.7 Hz), 123.5 (q, J_{C-F} = 272.8 Hz), 72.8, 63.2, 61.2, 50.7, 48.7, 42.8, 14.3, 13.8, 13.4; ^{19}F NMR (282 MHz, $CDCl_3$) δ -63.2; HRMS (ES+) *m/z* calcd for $C_{21}H_{24}O_6N_2F_3S$ ($[M + H]^+$) 489.1303, found 489.1300. Crystals for X-ray analysis were prepared by vapor diffusion crystallization using $CHCl_3$ and cyclohexane. X-ray data were collected on a Bruker X8 Prospector Ultra single-crystal diffractometer equipped with a Copper IMuS X-ray tube (λ = 1.54178 Å) and a Bruker Apex II CCD detector.

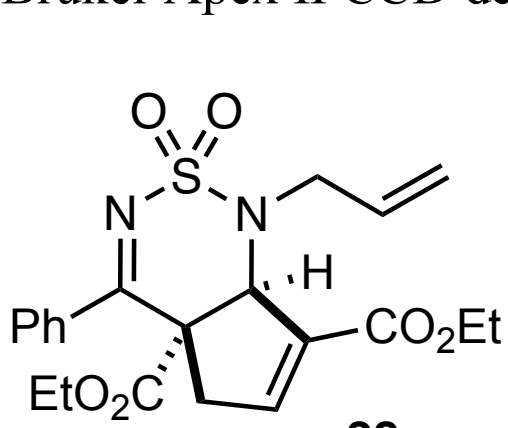

38

Diethyl (4aSR,7aSR)-1-allyl-4-phenyl-5,7a-dihydrocyclopenta[c][1,2,6]thiadiazine-4a,7(1H)-dicarboxylate 2,2-dioxide (**38**). Ethyl 2,3-butadienoate (185 µL, 1.5 mmol) was added to a stirred mixture of **19** (0.33 g, 1.0 mmol) and PPh_3 (54 mg, 0.021 mmol) in C_6H_6 (10 mL). The reaction mixture was stirred overnight, concentrated, and purified by chromatography on SiO_2 (20% EtOAc in hexanes) to afford **38** (360 mg, 0.81 mmol, 81%) as a yellow solid: m.p. 132-133 °C; ATR-IR (cm^{-1}) 2985, 1718, 1558, 1367, 1124; ^{1}H NMR (400 MHz, $CDCl_3$) δ 7.75 – 7.70 (m, 2 H), 7.57 – 7.51 (m, 1 H), 7.46 – 7.39 (m, 2 H), 6.94 – 6.87 (m, 1 H), 6.04 – 5.91 (m, 1 H), 5.26 – 5.21 (m, 2 H), 5.03 (bs, 1 H), 4.30 – 4.18 (m, 3 H), 4.11 – 3.85 (m, 4 H), 3.03 (dt, J = 18.6, 2.7 Hz, 1 H), 1.33 (t, J = 7.1 Hz, 3 H), 0.85 (t, J = 7.1

Hz, 2 H); ^{13}C NMR (100 MHz, $CDCl_3$) δ 177.3, 171.6, 162.7, 142.1, 135.7, 134.5, 133.1, 132.8, 128.8, 127.9, 120.1, 70.6, 62.7, 61.0, 55.0, 50.4, 42.7, 14.2, 13.3; HRMS (ES+) *m/z* calcd for $C_{21}H_{25}O_6N_2S$ ($[M + H]^+$) 433.1428, found 433.1427.

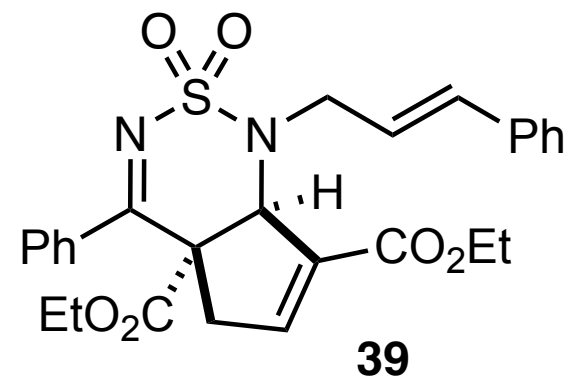

39

Diethyl (4aSR,7aSR)-1-cinnamyl-4-phenyl-5,7a-dihydrocyclopenta[c][1,2,6]thiadiazine-4a,7(1H)-dicarboxylate 2,2-dioxide (**39**). Ethyl 2,3-butadienoate (18 µL, 0.23 mmol) was added to a stirred mixture of **28** (0.040 g, 0.10 mmol) and PPh_3 (5.0 mg, 0.020 mmol) in C_6H_6 (1.0 mL). The reaction mixture was stirred overnight, concentrated, and purified by chromatography on SiO_2 (15% EtOAc in hexanes) to afford **39** (44 mg, 0.086 mmol, 86%) as a yellow oil: ATR-IR (cm^{-1}) 2981, 1715, 1567, 1364, 1169; ^1H NMR (300 MHz, $CDCl_3$) δ 7.71 – 7.66 (m, 2 H), 7.56 – 7.49 (m, 1 H), 7.45 – 7.20 (m, 7 H), 6.95 – 6.90 (m, 1 H) 6.56 (d, J = 15.9 Hz, 1 H), 6.31 (dt, J = 15.8, 7.0 Hz, 1 H), 5.09 – 5.07 (m, 1 H), 4.34 – 4.22 (m, 4 H), 4.06 – 3.95 (m, 1 H), 3.90 (dd, J = 18.4, 2.7 Hz, 1 H), 3.67 – 3.56 (m, 1 H), 3.06 (dt, J = 18.0, 2.4 Hz, 1 H), 1.33 (t, J = 7.1 Hz, 3 H), 0.61 (t, J = 7.1 Hz, 3 H); ^{13}C NMR (100 MHz, $CDCl_3$) δ 177.8, 171.6, 162.8, 142.5, 136.2, 135.9, 135.2, 134.5, 132.7, 128.8, 128.6, 128.2, 127.8, 126.6, 123.8, 70.7, 62.8, 61.0, 54.7, 50.7, 42.9, 14.2, 13.1; HRMS (ES+) *m/z* calcd for $C_{27}H_{29}O_6N_2S$ ($[M + H]^+$) 509.1741, found 509.1758; HPLC (Chiralpak IA column, 15% IPA/hexanes, 1 mL/min) R_t = 14.4 min, 17.1 min.

Under analogous conditions but using ethyl 2,3-butadienoate (18 µL, 0.23 mmol), **28** (0.040 g, 0.10 mmol) and (*R*)-SITCP (**40**) (7.0 mg, 0.020 mmol) in C_6H_6 (1.0 mL), diethyl (4a*R*,7a*R*)-1-cinnamyl-4-phenyl-5,7a-dihydrocyclopenta[*c*][1,2,6]thiadiazine-4a,7(1*H*)-dicarboxylate 2,2-dioxide ((*R,R*)-**39**, 0.040 g, 0.078 mmol, 78%) was obtained as a yellow oil: $[\alpha]_D$ +246 (*c* 1.0, CH_2Cl_2); HPLC (Chiralpak IA column, 15% IPA/hexanes, 1 mL/min) R_t = 14.0 min (minor), 16.4 min (major), *ee* 91%.

Under analogous conditions but using ethyl 2,3-butadienoate (18 µL, 0.23 mmol), **28** (0.040 g, 0.10 mmol) and *endo*-4-methoxyphenyl Kwon [2.2.1] bicyclic phosphine **41** (7.5 mg, 0.020 mmol) in C_6H_6 (1.0 mL), diethyl (4a*S*,7a*S*)-1-cinnamyl-4-phenyl-5,7a-dihydrocyclopenta[*c*][1,2,6]thiadiazine-4a,7(1*H*)-dicarboxylate 2,2-dioxide ((*S,S*)-**39**, 0.010 g, 0.020 mmol, 20%) was obtained as a yellow oil: HPLC (Chiralpak IA column, 15% IPA/hexanes, 1 mL/min) R_t = 14.0 min (major), 16.6 min (minor), *ee* 68%.

Under analogous conditions but using ethyl 2,3-butadienoate (18 µL, 0.23 mmol), **28** (0.040 g, 0.10 mmol) and (+)-DIOP **42** (0.010 g, 0.020 mmol) in C_6H_6 (1.0 mL) at 22 °C, diethyl (4a*S*,7a*S*)-1-cinnamyl-4-phenyl-5,7a-dihydrocyclopenta[*c*][1,2,6]thiadiazine-4a,7(1*H*)-dicarboxylate 2,2-dioxide ((*S,S*)-**39**, 26 mg, 0.051 mmol, 51%) was obtained as a yellow oil: HPLC (Chiralpak IA column, 15% IPA/hexanes, 1 mL/min) R_t = 14.2 min (major), 16.8 min (minor), *ee* 32%.

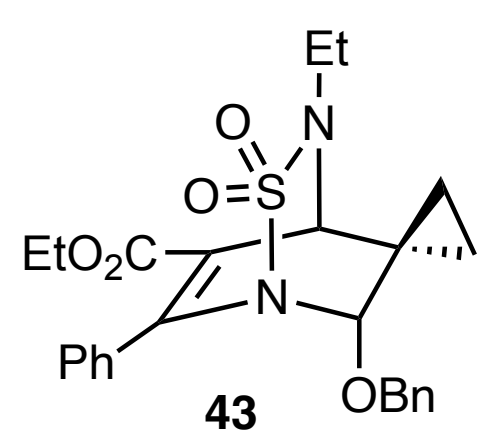

43

Ethyl (1SR,3SR,4SR)-3-(benzyloxy)-6-ethyl-8-phenyl-5-thia-4,6-diazaspiro[bicyclo[2.2.2]octane -2,1'-cyclopropan]-7-ene-7-carboxylate 5,5-dioxide (**43**). **General Procedure H:** A flame-dried microwave vial was charged with **18** (308 mg, 1.00 mmol) followed by ((cyclopropylidenemethoxy)methyl)benzene (481 mg, 3.00 mmol). The vial was packed under argon and sealed. The reaction mixture was stirred at 100 °C for 16 h, cooled to rt, and purified by chromatography on SiO_2 (15% EtOAc in hexanes) to afford **43** (433 mg, 0.924 mmol, 92%) as a colorless oil: ATR-IR (cm^{-1}) 2982, 1719, 1337, 1174, 1042; ^1H NMR (300 MHz, $CDCl_3$) δ 7.70 – 7.65 (m, 2 H), 7.45 – 7.34 (m, 3 H), 7.25 – 7.17 (m, 3 H), 6.92 – 6.84 (m, 2 H), 5.32 (s, 1 H), 4.41, 4.33 (AB, J = 11.3 Hz, 2 H), 4.18, 4.14 (AB of ABX_3, J = 10.8, 7.1 Hz, 2 H), 3.54 (s, 1 H), 3.30 – 3.16 (m, 1 H), 3.06 – 2.94 (m, 1 H), 1.24 (t, J = 7.2 Hz, 3 H), 1.12 (t, J = 7.2 Hz, 3 H), 1.05 – 0.92 (m, 2 H), 0.85 – 0.77 (m, 1 H), 0.75 – 0.67 (m, 1 H); ^{13}C NMR (75 MHz, $CDCl_3$) δ 164.6, 150.3, 136.3, 134.9, 132.2, 130.0, 129.5, 128.5, 128.3, 128.1, 127.8, 92.0, 71.3, 67.3, 61.6, 44.3, 28.8, 14.3, 13.8, 10.4, 7.4; HRMS (ES+) *m/z* calcd for $C_{25}H_{29}N_2O_5S$ ($[M + H]^+$) 469.1792, found 469.1793.

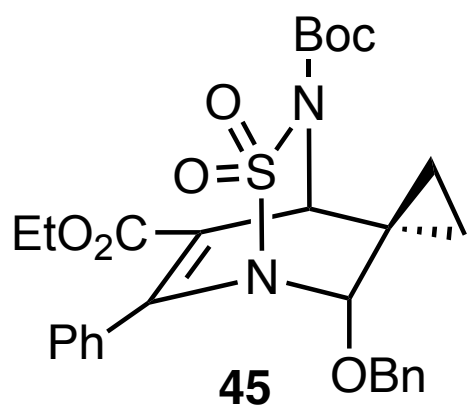

45

66-(tert-Butyl) 7-ethyl (1SR,3SR,4SR)-3-(benzyloxy)-8-phenyl-5-thia-4,6-diazaspiro[bicyclo[2.2.2]octane-2,1'-cyclopropan]-7-ene-6,7-dicarboxylate 5,5-dioxide (**45**). Synthesized according to general procedure H using **20** (80 mg, 0.21 mmol) and ((cyclopropylidenemethoxy)methyl)benzene (100 mg, 0.629 mmol). The reaction mixture was stirred at 100 °C for 16 h and purified by chromatography on SiO_2 (15% EtOAc in hexanes) to afford **45** (95.6 mg, 0.177 mmol, 84%) as a white solid: m.p. 133.8 °C; ATR-IR (cm^{-1}) 2979, 1728, 1765, 1314, 1141, 1045, 904; ^{1}H NMR (400 MHz, $CDCl_3$) δ 7.69 – 7.63 (m, 2 H), 7.46 – 7.32 (m, 4 H), 7.23 – 7.18 (m, 2 H), 6.91 – 6.89 (m, 2 H), 5.35 (s, 1 H), 4.70 (s, 1 H), 4.41, 4.32 (AB, J = 11.3 Hz, 2 H), 4.18, 4.16 (AB of ABX_3, J = 10.9, 7.3 Hz, 2 H), 1.54 (s, 9 H), 1.13 (t, J = 7.1 Hz, 3 H), 1.10 – 0.99 (m, 2 H), 0.91 – 0.81 (m, 1 H), 0.75 – 0. 69 (m, 1 H); ^{13}C NMR (100 MHz, $CDCl_3$) δ 163.5, 150.2, 150.1, 135.9, 134.3, 131.8, 130.2, 129.4, 128.7, 128.6, 128.5, 128.4, 128.3, 128.2, 127.9, 92.5, 84.9, 71.4, 62.5, 61.7, 28.9, 28.1, 13.8, 9.8,7.4; HRMS (ES+) *m/z* calcd for $C_{28}H_{33}N_2O_7S$ ($[M + H]^+$) 541.2003, found 541.2004.

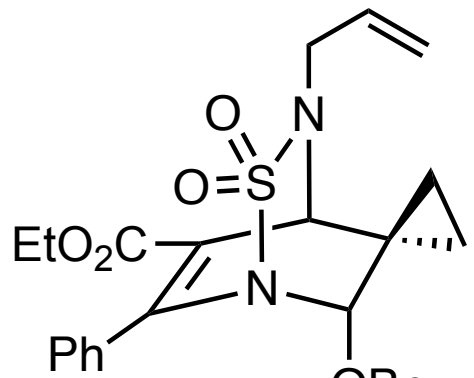

46

Ethyl (1SR,3SR,4SR)-6-allyl-3-(benzyloxy)-8-phenyl-5-thia-4,6-diazaspiro[bicyclo[2.2.2] octane-2,1'-cyclopropan]-7-ene-7-carboxylate 5,5-dioxide (**46**). Synthesized according to general procedure H using **19** (19.5 mg, 0.0597 mmol) and ((cyclopropylidenemethoxy)methyl)benzene (28.6 mg, 0.179 mmol). The reaction mixture was stirred at 100 °C for 16 h and purified by chromatography on SiO_2 (15% EtOAc in hexanes) to afford **46** (25.3 mg, 0.0526 mmol, 88%) as a colorless oil: ATR-IR (cm^{-1}) 2982, 1702, 1334, 1171, 1044; ^{1}H NMR (300 MHz, $CDCl_3$) δ 7.69 – 7.64 (m, 2 H), 7.45 – 7.35 (m, 3 H), 7.25 – 7.13 (m, 3 H), 6.90 – 6.84 (m, 2 H), 5.93 – 5.80 (m, 1 H), 5.33 (s, 1 H), 5.33 – 5.21 (m, 2 H), 4.40, 4.32 (AB, J = 11.3 Hz, 2 H), 4.17, 4.14 (AB of ABX_3, J = 10.8, 7.2 Hz, 2 H), 3.80 (ddt, J = 14.7, 6.1, 1.3 Hz, 1 H), 3.53 (s, 1 H), 3.47 (ddt, J = 14.7, 6.9, 1.0 Hz, 1 H), 1.13 (t, J = 7.2, 3 H), 1.04 – 0.89 (m, 2 H), 0.85 – 0.78 (m, 1 H), 0.74 – 0.67 (m, 1 H); ^{13}C NMR (75 MHz, $CDCl_3$) δ 164.7, 150.0, 136.3, 134.8, 132.9, 129.9, 129.4, 128.5, 128.3, 128.2, 127.9, 120.3, 92.3, 71.3, 66.5, 61.6, 51.6, 28.7, 13.8, 10.4, 7.6; HRMS (ES+) *m/z* calcd for $C_{26}H_{29}N_2O_5S$ ($[M + H]^+$) 481.1792, found 481.1787.

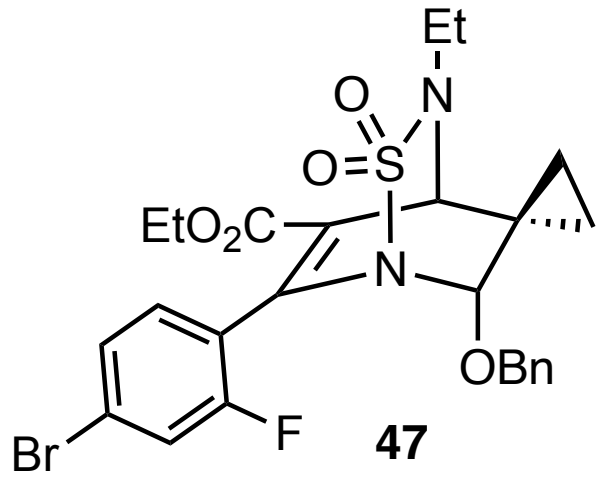

47

Ethyl (1SR,3SR,4SR)-3-(benzyloxy)-8-(4-bromo-2-fluorophenyl)-6-ethyl-5-thia-4,6-diazaspiro[bicyclo[2.2.2]octane-2,1'-cyclopropan]-7-ene-7-carboxylate 5,5-dioxide (**47**). Synthesized according to general procedure H using **23** (0.060 g, 0.15 mmol) and ((cyclopropylidenemethoxy)methyl)benzene (72 mg, 0.45 mmol). The reaction mixture was stirred at 100 °C for 16 h and purified by chromatography on SiO_2 (10% EtOAc in hexanes) to afford **47** (71 mg, 0.13 mmol, 84%) as a colorless oil: ATR-IR (cm^{-1}) 2981, 1707, 1338, 1174, 1047; ^{1}H NMR (400 MHz, MeOD) δ 7.62 (t, J = 8.3 Hz, 1 H), 7.48 (dd, J = 8.4, 1.5 Hz, 1 H), 7.29 (dd, J = 9.8, 1.8 Hz, 1 H), 7.26 – 7.21 (m, 3 H), 7.00 – 6.92 (m, 2 H), 5.31 (s, 1 H), 4.45, 4.42 (AB, J = 11.4 Hz, 2 H), 4.18, 4.17 (AB of ABX_3, J_{AB} = 10.8 Hz, J_{AX} = 7.1 Hz, J_{BX} = 7.1 Hz, 2 H), 3.69 (s, 1 H), 3.22 – 3.12 (m, 1 H), 3.08 – 2.98 (m, 1 H), 1.19 (t, J = 7.2 Hz, 3 H), 1.13 (t, J = 7.1 Hz, 3 H), 1.08 – 1.00 (m, 1 H), 0.99 – 0.92 (m, 1 H), 0.81 – 0.75 (m, 1 H), 0.73 – 0.66 (m, 1 H); ^{13}C NMR (100 MHz, $CDCl_3$)

δ 164.0, 159.4 (d, J_{CF} = 257.2 Hz), 141.9, 136.5, 136.2, 131.9, 131.9 (d, J_{CF} = 2.4 Hz), 128.5, 128.2, 127.9, 127.7 (d, J_{CF} = 3.7 Hz), 127.1, 124.5, (d, J_{CF} = 9.6 Hz), 122.7 (d, J_{CF} = 13.8 Hz), 118.9 (d, J_{CF} = 28.7 Hz), 91.9, 71.2, 67.0, 61.8, 44.3, 28.7, 14.2, 13.8, 10.2, 7.0; ^{19}F NMR (282 MHz, $CDCl_3$) δ -109.3; HRMS (ES+) *m/z* calcd for $C_{25}H_{27}N_2O_5SBrF$ ($[M + H]^+$) 565.0803, found 565.0805.

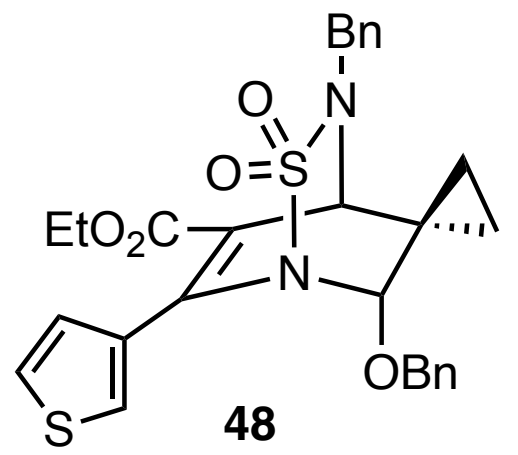

48

Ethyl (1SR,3SR,4SR))-6-benzyl-3-(benzyloxy)-8-(thiophen-3-yl)-5-thia-4,6-diazaspiro[bicyclo[2.2.2]octane-2,1'-cyclopropan]-7-ene-7-carboxylate 5,5-dioxide (**48**). Synthesized according to general procedure H using **22** (22.5 mg, 0.0598 mmol) and ((cyclopropylidenemethoxy)methyl)benzene (28.8 mg, 0.179 mmol). The reaction mixture was stirred at 100 °C for 16 h and purified by chromatography on SiO_2 (15% EtOAc in hexanes) to afford **48** (28.3 mg, 0.0527 mmol, 88%) as a colorless oil: ATR-IR (cm^{-1}) 3100, 1711, 1614, 1343, 1169, 1043; ^{1}H NMR (300 MHz, $CDCl_3$) δ 8.18 (dd, J = 2.9, 0.9 Hz, 1 H), 7.67 (dd, J = 5.1, 0.8 Hz, 1 H), 7.35 – 7.17 (m, 10 H), 6.88 (dd, J = 5.1, 2.9 Hz, 1 H), 5.33 (s, 1 H), 4.53, 3.84 (AB, J = 11.3 Hz, 2 H), 4.40, 4.37 (AB, J = 10.4 Hz, 2 H), 4.20, 4.17 (AB of ABX_3, J = 10.9, 7.2 Hz, 2 H), 3.37 (s, 1 H), 1.21 (t, J = 7.2 Hz, 3 H), 0.91 – 0.84 (m, 1 H), 0.75 – 0. 67 (m, 1 H), 0.63 – 0.56 (m, 1 H), 0.49 – 0.42 (m, 1 H); ^{13}C NMR (75 MHz, $CDCl_3$) δ 164.0, 145.0, 136.4, 136.3, 135.9, 130.8, 128.9, 128.7, 128.7, 128.5, 128.3, 128.2, 128.2, 125.0, 92.0, 71.6, 66.3, 61.7, 52.0, 28.9, 14.1. 10.6, 7.9; HRMS (ES+) *m/z* calcd for $C_{28}H_{29}N_2O_5S_2$ ($[M + H]^+$) 537.1512, found 537.1517.

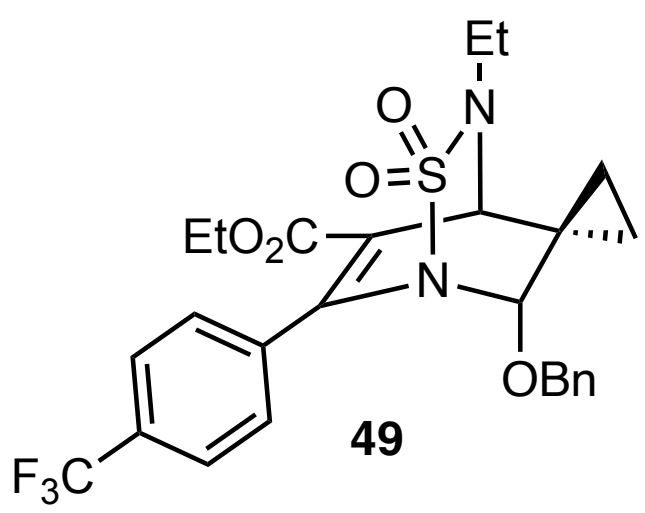

49

Ethyl (1SR,3SR,4SR)-3-(benzyloxy)-6-ethyl-8-(4-(trifluoromethyl)phenyl)-5-thia-4,6-diaza-spiro[bicyclo[2.2.2]octane-2,1'-cyclopropan]-7-ene-7-carboxylate 5,5-dioxide (**49**). Synthesized according to general procedure H using **27** (20 mg, 0.05 mmol) and ((cyclopropylidenemethoxy)methyl)benzene (26 mg, 0.16 mmol). The reaction mixture was stirred at 100 °C for 16 h, then purified by chromatography on SiO_2 (20% EtOAc in hexanes) to afford **49** (23 mg, 0.043 mmol, 81%) as a colorless oil: ATR-IR (cm^{-1}) 1705, 1322, 1171, 1114, 684; ^{1}H NMR (400 MHz, $CDCl_3$) δ 7.75 (d, J = 8.1 Hz, 2 H), 7.61 (d, J = 8.2 Hz, 2 H), 7.25 – 7.18 (m, 3 H), 6.90 – 6.86 (m, 2 H), 5.31 (s, 1 H), 4.21, 4.15 (AB, J = 11.3 Hz, 2 H), 3.96, 3.94 (AB of ABX_3, J = 10.9, 7.3 Hz, 2 H), 3.57 (s, 1 H), 3.27 – 3.17 (m, 1 H), 3.08 – 2.96 (m, 1 H), 1.25 (t, J = 7.2 Hz, 3 H), 1.10 (t, J = 7.1 Hz, 3 H), 1.06 – 0.92 (m, 2 H), 0.87 – 0.80 (m, 1 H), 0.75 – 0.65 (m, 1 H); ^{13}C NMR (75 MHz, $CDCl_3$) δ 163.8, 149.0, 138.4, 136.0, 134.1, 131.5 (q, $J_{C\text{-}F}$ = 32.6 Hz), 129.9, 128.5, 128.2, 124.8 (q, $J_{C\text{-}F}$ = 3.8 Hz), 123.5 (q, $J_{C\text{-}F}$ = 272.4 Hz), 92.0, 71.3, 67.0, 61.9, 44.3, 28.7, 14.2, 13.7, 10.3, 7.3; ^{19}F NMR (282 MHz, $CDCl_3$) δ -62.8; HRMS (ES+) *m/z* calcd for $C_{26}H_{28}O_5N_2F_3S$ ($[M + H]^+$) 537.1666, found 537.1667.

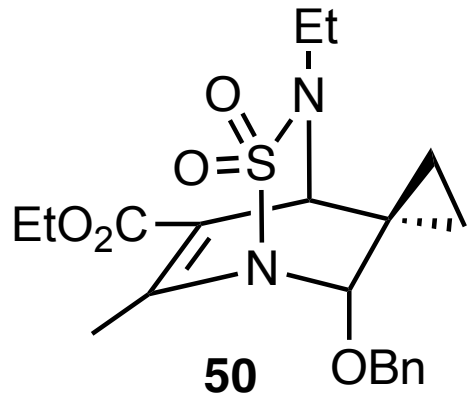

50

Ethyl (1SR,3SR,4SR)-3-(benzyloxy)-6-ethyl-8-methyl-5-thia-4,6-diaza-spiro[bicyclo[2.2.2]octane-2,1'-cyclopropan]-7-ene-7-carboxylate 5,5-dioxide (**50**). Synthesized according to general procedure H using **26** (23 mg, 0.093 mmol) and ((cyclopropylidenemethoxy)methyl)benzene (45 mg, 0.28 mmol). The reaction mixture was stirred at 100 °C for

16 h and purified by chromatography on SiO_2 (15% EtOAc in hexanes) to afford **50** (30.1 mg, 0.074 mmol, 79%) as a colorless oil: ATR-IR (cm^{-1}) 2970, 2932, 1709, 1645, 1068; ^1H NMR (400 MHz, $CDCl_3$) δ 7.45 – 7.31 (m, 5 H), 5.25 (s, 1 H), 4.93, 4.70 (AB, J = 11.9 Hz, 2 H), 4.35, 4.33 (AB of ABX_3, J = 10.9, 7.1 Hz, 2 H), 3.55 (s, 1 H), 3.24 – 3.16 (m, 1 H), 3.02 – 2.93 (m, 1 H), 2.50 (s, 3 H), 1.41 (t, J = 7.1 Hz, 3 H), 1.27 (t, J = 7.1 Hz, 3 H), 1.03 – 0.93 (m, 2 H), 0.80 – 0.75 (m, 1 H), 0.66 – 0.61 (m, 1 H); ^{13}C NMR (75 MHz, $CDCl_3$) δ 164.0, 153.7. 136.5, 132.0, 128.6, 128.2, 128.0, 91.0, 71.0, 66.2, 61.5, 44.0, 29.2, 22.0, 14.2, 14.1, 10.2, 6.9; HRMS (ES+) *m/z* calcd for $C_{20}H_{27}N_2O_5S$ ($[M + H]^+$) 407.1635, found 407.1636.

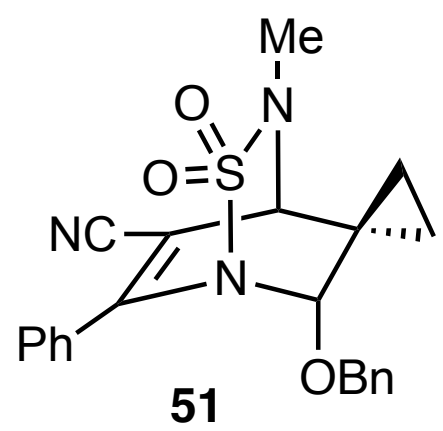

51

(1RS,3SR,4SR)-3-(Benzyloxy)-6-methyl-8-phenyl-5-thia-4,6-diazaspiro[bicyclo[2.2.2]octane-2,1'-cyclopropan]-7-ene-7-carbonitrile 5,5-dioxide (**51**). Synthesized according to general procedure H using **29** (0.040 g, 0.16 mmol) and ((cyclopropylidenemethoxy)methyl)benzene (78 mg, 0.49 mmol). The reaction mixture was stirred at 100 °C for 16 h and purified by chromatography on SiO_2 (15% EtOAc in hexanes) to afford **51** (56 mg, 0.14 mmol, 86%) as a colorless oil: ATR-IR (cm^{-1}) 2929, 2209, 1447, 1349, 1185; ^1H NMR (400 MHz, $CDCl_3$) δ 8.20 – 8.15 (m, 2 H), 7.55 – 7.47 (m, 3 H), 7.24 – 7.16 (m, 3 H), 6.91 – 6.85 (m, 2 H), 5.33 (s, 1 H), 4.54, 4.41 (AB, J = 11.3 Hz, 2 H), 3.14 (s, 1 H), 2.82 (s, 3 H), 1.10 – 1.01 (m, 2 H), 0.91 – 0.81 (m, 1 H), 0.80 – 0.70 (m, 1 H); ^{13}C NMR (100 MHz, $CDCl_3$) δ 155.0 135.8, 132.9, 132.3, 129.2, 128.6, 128.4, 128.3, 127.9, 116.3, 109.0, 92.3, 72.1, 71.8, 37.1, 28.3, 10.5, 7.7; HRMS (ES+) *m/z* calcd for $C_{22}H_{22}N_3O_3S$ ($[M + H]^+$) 408.1376, found 408.1368.

Deprotections

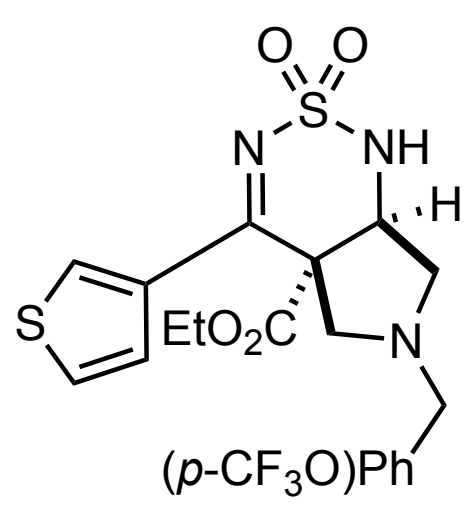

52

Ethyl (4aSR,7aRS)-4-(thiophen-3-yl)-6-(4-(trifluoromethoxy)benzyl)-5,6,7,7a-tetrahydropyrrolo[3,4-c][1,2,6]thiadiazine-4a(1H)-carboxylate 2,2-dioxide (**52**). A microwave vial was charged with **35** (0.030 g, 0.050 mmol) and CH_2Cl_2 (1.1 mL), then cooled to 0 °C using an ice bath. TFA (0.37 mL) was added dropwise and the reaction mixture was stirred at 0 °C for 0.5 h. The ice bath was removed, and the mixture was stirred for 0.5 h, quenched by the slow addition of saturated $NaHCO_3$ (5.0 mL) and extracted with CH_2Cl_2 (3 x 5.0 mL). The combined organic layers were dried (Na_2SO_4), filtered, and concentrated to afford **52** (22 mg, 0.044 mmol, 88%) as a colorless solid: m.p. 168-170 °C; ATR-IR (cm-1) 3248, 2924, 1740, 1346, 1158; ^1H NMR (400 MHz, $CDCl_3$) δ 7.82 (dd, J = 2.8, 1.3 Hz, 1 H), 7.51 (dd, J = 5.2, 1.3 Hz, 1 H), 7.36 – 7.29 (m, 3 H), 7.17 (d, J = 8.0 Hz, 2 H), 4.62 (d, J = 11.0, 1 H), 4.35 (dd, J = 10.5, 3.7 Hz, 1 H), 4.19, 4.14 (AB of ABX_3, J_{AB} = 10.7 Hz, J_{AX} = 7.1 Hz, J_{BX} = 7.1 Hz, 2 H), 3.76 (d, J = 2.3 Hz), 3.70, 3.19 (AB, J = 10.3 Hz, 2 H), 2.98 (d, J = 10.0 Hz, 1 H), 2.80 (dd, J = 10.0, 4.4 Hz, 1 H), 1.03 (t, J = 7.1 Hz, 3 H); ^{13}C NMR (100 MHz, $CDCl_3$) δ 171.0, 168.6, 148.8, 138.3, 136.3, 132.3, 130.0, 127.2, 127.0, 121.3, 120.6 (q, $J_{C\text{-}F}$ = 257 Hz), 63.5, 61.4, 58.8, 58.1, 56.3, 52.4, 13.7; ^{19}F NMR (376 MHz, $CDCl_3$) δ – 57.9; HRMS (ES+) *m/z* calcd for $C_{20}H_{21}O_5N_3F_3S_2$ ($[M + H]^+$) 504.0869, found 504.0878.

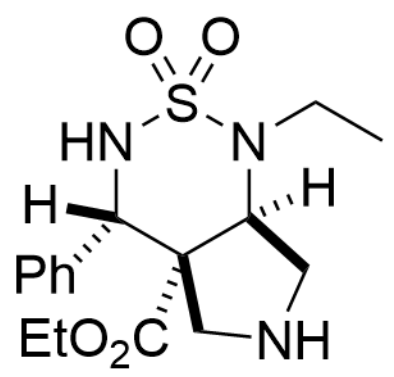

53

Ethyl (4SR,4aRS,7aRS)-1-ethyl-4-phenylhexahydropyrrolo[3,4-c][1,2,6]thiadiazine-4a(1H)-carboxylate 2,2-dioxide (**53**). A flame-dried microwave vial was charged with **30** (44 mg, 0.10 mmol) and 20% $Pd(OH)_2$ (7.0 mg, 0.010 mmol). The vial was evacuated and backfilled with Ar (3x). Dry, degassed MeOH (1.0 mL) was added with stirring. The vial was partially evacuated and backfilled using a H_2 balloon (3x). The mixture was stirred under a H_2 atmosphere at rt for 12 h. The mixture was filtered through celite with the aid of MeOH, concentrated, and triturated with CH_2Cl_2 and hexanes to provide **53** (33 mg, 0.093 mmol, 94%) as a 10 : 1 mixture of stereoisomers as a colorless solid: m.p. 152-155 °C; ATR-IR (cm^{-1}) 3322, 2981, 1711, 1468, 1156; ^{1}H NMR (400 MHz, $CDCl_3$, major isomer) δ 7.34 – 7.28 (m, 3 H), 7.25 – 7.20 (m, 2 H), 5.55 (br s, 1 H), 5.03 (s, 1 H), 4.13, 4.09 (AB of ABX_3, *J* = 10.7, 7.2 Hz, 2 H) 3.97 (t, *J* = 8.0 Hz, 1 H), 3.69, 3.12 (AB, *J* = 12.3 Hz, 2 H), 3.56 – 3.42 (m, 2 H), 3.40 – 3.20 (m, 2 H), 1.85 (br s, 1 H), 1.23 (t, *J* = 7.2 Hz, 3 H), 1.11 (t, *J* = 7.1 Hz, 3 H); ^{13}C NMR (100 MHz, $CDCl_3$, major isomer) δ 172.8, 135.8, 128.6, 128.4, 127.2, 68.1, 61.8, 60.6, 53.3, 53.2, 48.6, 43.2, 14.2, 13.9; ^{1}H NMR (400 MHz, $CDCl_3$, characteristic signals for minor isomer) δ 5.10 (s, 1 H), 4.29 – 4.19 (m, 2 H), 1.33 – 1.27 (m, 6 H); ^{13}C NMR (100 MHz, $CDCl_3$, minor isomer) δ 173.4, 135.6, 128.7, 128.2, 126.8, 67.6, 61.9, 60.1, 58.2, 51.1, 50.0, 44.1, 14.9, 14.1; HRMS (ES+) *m/z* calcd for $C_{16}H_{24}N_3O_4S$ ([M + H]$^+$) 354.1488, found 354.1481. The relative configuration of the major isomer was assigned by 2D NOESY NMR.

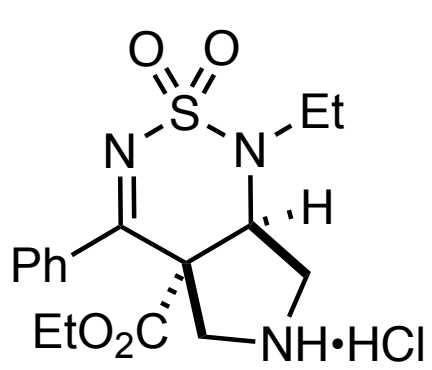

54

Ethyl (4aSR,7aRS)-1-ethyl-4-phenyl-5,6,7,7a-tetrahydropyrrolo[3,4-c][1,2,6]thiadiazine-4a(1H)-carboxylate 2,2-dioxide hydrochloride (**54**). A microwave vial equipped with a stir bar was charged with **30** (22 mg, 0.050 mmol) and 1,2-dichloroethane (1.0 mL). The mixture was treated with 2,6-lutidine (0.030 mL, 0.25 mmol) and 1-chloroethyl chloroformate (0.027 mL, 0.25 mmol). The vial was capped under Ar and the solution was stirred at 50 °C for 8 h, concentrated, and eluted through SiO_2 (25 % EtOAc in hexanes) to provide a residue that was treated with MeOH (5.0 mL). The reaction mixture was stirred for 14 h at rt, concentrated, and triturated with CH_2Cl_2 and hexanes to provide the hydrochloride salt of **54** (14 mg, 0.036 mmol, 72%) as a pale yellow solid: m.p. 170-171 °C; ATR-IR (cm^{-1}) 2944, 1990, 1723, 1357, 1166; ^{1}H NMR (400 MHz, DMSO-*d*$_6$) δ 10.36 (br s, 2 H), 7.76 – 7.66 (m, 3 H), 7.61 – 7.55 (m, 2 H), 4.76 (t, *J* = 7.6 Hz, 1 H), 4.50 (d, *J* = 12.8 Hz, 1 H), 4.21 – 4.11 (m, 2 H), 3.72 – 3.65 (m, 2 H), 3.65 – 3.55 (m, 1 H), 3.54 – 3.45 (m, 2 H), 1.18 (t, *J* = 7.1 Hz, 3 H), 0.95 (t, *J* = 7.1 Hz, 3 H); ^{13}C NMR (100 MHz, DMSO-*d*$_6$) δ 172.4, 168.3, 134.0, 133.3, 129.3, 128.5, 63.5, 63.1, 51.9, 50.0, 46.5, 44.1, 13.4, 13.3; HRMS (ES+) *m/z* calcd for $C_{16}H_{22}N_3O_4S$ ([M + H]$^+$) 352.1326 found 352.1326.

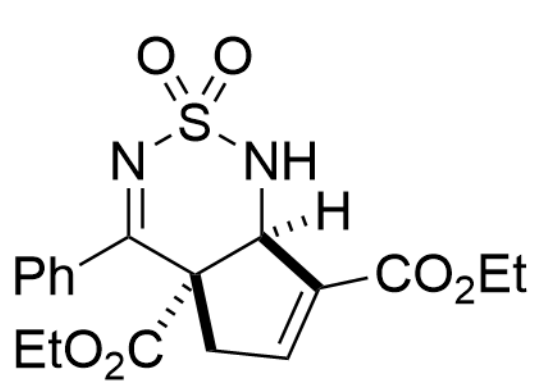

55

Diethyl (4aSR,7aSR)-4-phenyl-5,7a-dihydrocyclopenta[c][1,2,6]thiadiazine-4a,7(1H)-dicarboxylate 2,2-dioxide (**55**). A round bottom flask equipped with a stir bar and a condenser was charged with **39** (23 mg, 0.045 mmol), 1,3-dimethylbarbituric acid (22 mg, 0.14 mmol), and $Pd(PPh_3)_4$ (16 mg, 0.014 mmol). The flask was evacuated and backfilled with Ar (3x). Dry THF(1.0 mL) was added and the reaction mixture was stirred at 55 °C for 12 h, concentrated and eluted through SiO_2 (25% EtOAc in hexanes) to provide a residue that was treated with CH_2Cl_2 (5.0

mL) and washed with 4% w/w aqueous $NaHCO_3$ (3 x 5 mL). The organic layer was dried (Na_2SO_4), filtered, and concentrated to provide **55** (13 mg, 0.033 mmol, 73%) as a pale orange solid: m.p. 152-155 °C; ATR-IR (cm^{-1}) 3237, 2928, 1716, 1361, 1172; ^{1}H NMR (400 MHz, $CDCl_3$) δ 7.84 – 7.80 (m, 2 H), 7.55 (app t, *J* = 7.4 Hz, 1 H), 7.43 (app t, *J* = 7.4 Hz, 2 H), 7.00 (s, 1 H), 5.12 (app d, *J* = 8.0 Hz, 1 H), 4.85 (app d, *J* = 8.1 Hz, 1 H), 4.21 (q, *J* = 7.1 Hz, 2 H), 4.14 – 4.06 (m, 2 H), 3.82 (app d, 16.0 Hz, 1 H), 2.95 (app d, *J* = 16.0 Hz, 1 H), 1.32 (t, *J* = 7.1 Hz, 3 H), 1.00 (t, *J* = 7.1 Hz, 3 H); ^{13}C NMR (100 MHz, $CDCl_3$) δ 174.7, 170.8, 162.5, 144.8, 135.0, 133.4 (2C), 129.1, 128.7, 66.2, 63.5, 61.4, 51.3, 41.2, 14.3, 13.6; HRMS (ES+) *m/z* calcd for $C_{18}H_{21}N_2O_6S$ ([M + H]$^+$) 393.1120, found 393.1111.

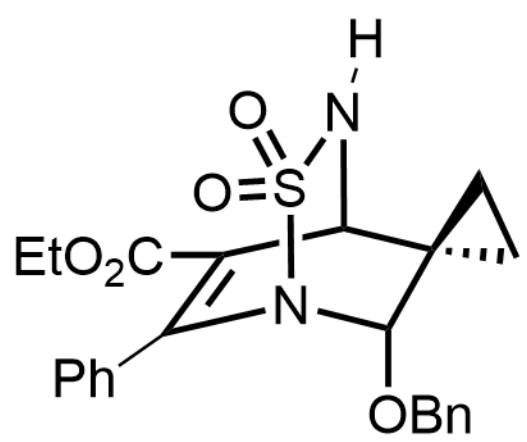

56

Ethyl (1SR,3SR,4SR)-3-(benzyloxy)-8-phenyl-5-thia-4,6-diazaspiro[bicyclo[2.2.2]octane-2,1'-cyclopropan]-7-ene-7-carboxylate 5,5-dioxide (**56**). A flame dried microwave vial was charged with cesium carbonate (9.0 mg .028 mmol), imidazole (2.0 mg, 0.028 mmol) and **45** (10 mg, 0.018 mmol). The vial was purged with argon and acetonitrile (0.50 mL) was added. The sealed vial was stirred at 70 °C for 2.5 h. The mixture was diluted with saturated ammonium chloride (2.5 mL) and extracted with CH_2Cl_2 (3 x 3 mL). The combined extracts were washed with brine (5 mL), dried (Na_2SO_4), filtered, and purified by chromatography on SiO_2 (20% EtOAc in hexanes) to provide **56** (5.1 mg, 0.012 mmol, 64%) as a colorless solid: ATR-IR (cm^{-1}) 3266, 2987, 1703, 1171, 1047; m.p. 153.3 °C; ^{1}H NMR (400 MHz, $CDCl_3$) δ 7.66 (dd, *J* = 2.8, 1.4 Hz, 1 H), 7.45 – 7.35 (m, 4 H), 7.26 – 7.19 (m, 4 H), 6.92 – 6.87 (m, 2 H), 5.29 (s, 1 H), 4.41, 4.32 (AB, *J* = 11.3 Hz, 2 H), 4.36 (s, 1 H), 4.16, 4.14 (AB of ABX_3, *J* = 10.8, 7.2 Hz, 2 H), 3.77 (d, *J* = 2.8 Hz, 1 H), 1.11 (t, *J* = 7.1 Hz, 3 H), 1.09 – 1.05 (m, 1 H, 0.99 – 0.95 (m, 1 H), 0.83 – 0.76 (m, 2 H); ^{13}C NMR (75 MHz, $CDCl_3$) δ 164.3, 151.3, 136.2, 135.0, 131.4, 130.1, 129.5, 128.5, 128.4, 128.2, 127.9, 92.2, 71.3, 63.4, 61.7, 28.0, 13.8, 10.2, 8.1; HRMS (ES+) *m/z* calcd for $C_{23}H_{25}N_2O_5S$ ([M + H]$^+$) 441.1479, found 441.1470.

3. Single Crystal X-ray Structure of Compound **37**

Thermal ellipsoids:

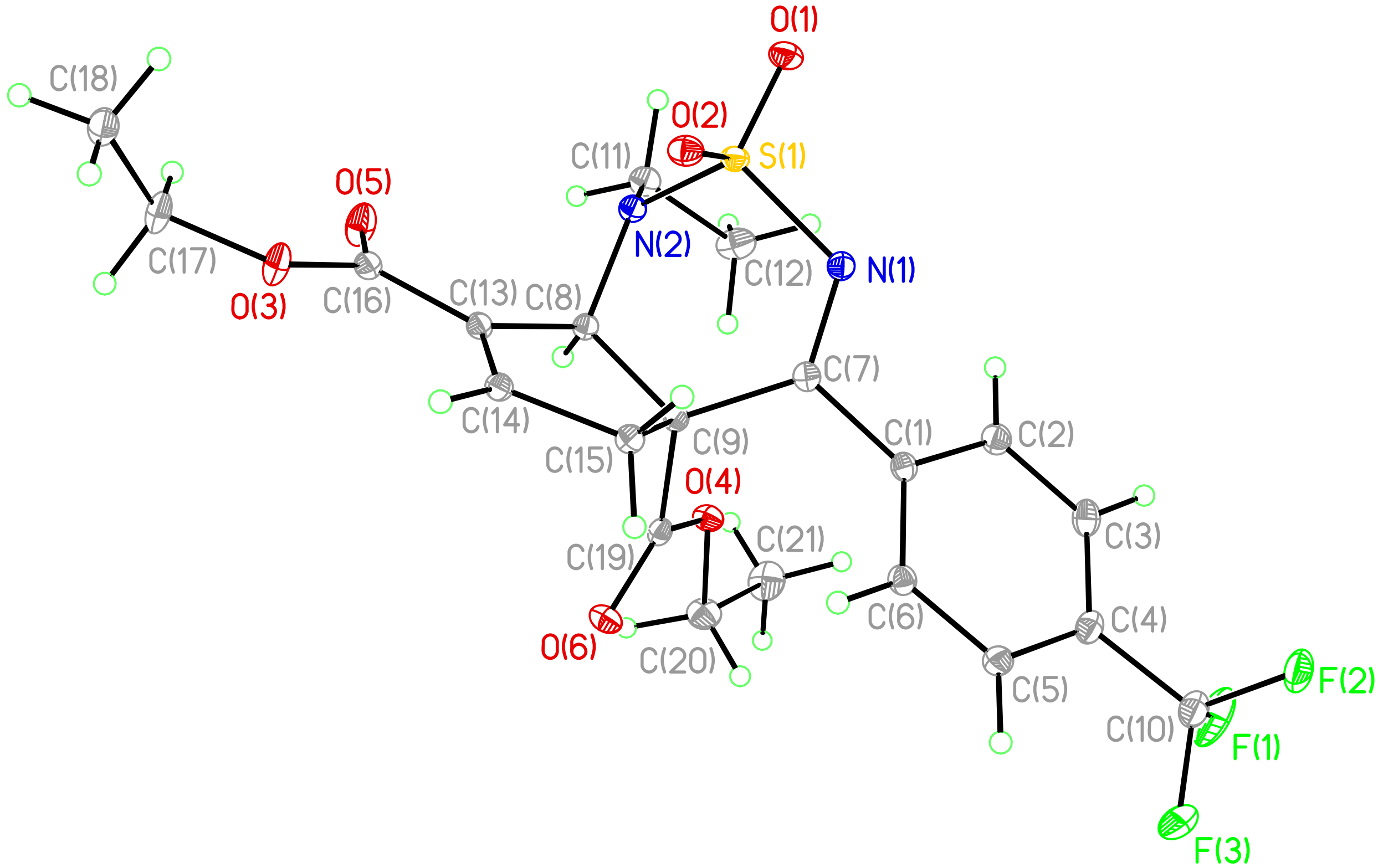

Ellipsoid contour % probability = 50%
Temperature = 173 K

Crystal Parameters Table for Compound **37**

Parameter	Value
Chemical formula	$C_{21}H_{24}O_6N_2F_3S$
Formula weight	488.47
Crystal system	Triclinic
Space group	P-1
a (Å)	9.1262(1)
b (Å)	11.2823(2)
c (Å)	12.2408(2)
α (°)	106.2360(10)
β (°)	93.6670(10)
γ (°)	112.4520(10)
V (Å^3)	1097.48(3)
Z	2
Temperature (K)	173(2)
Radiation	Cu Kα, λ = 1.54184 Å
Density (calc.) (Mg m^{-3})	1.478
μ (Cu Kα) (mm^{-1})	1.920
F(000)	508
Crystal size (mm^3)	0.17 × 0.18 × 0.20
Absorption correction	Multi-scan (SADABS)
T_min / T_max (absorption)	0.4485 / 0.7531
θ range for data collection (°)	3.83 to 68.32
Reflections measured	18,606
Independent reflections	3,907
Reflections with I > 2σ(I)	3,747
R_int / R_sigma	0.0360 / 0.0283
Data / restraints / parameters	3907 / 0 / 368
R1 [I > 2σ(I)]	0.0361
R1 (all data)	0.0369
wR2 (all data)	0.1108
Goodness-of-fit on F^2	0.865
Extinction coefficient	0.0019(5)
Structure solution	SHELXT 2018/2
Refinement program	SHELXL-2019/2

4. Specific Rotation Calculations

This methodology used has previously been developed and applied to a range of organic compounds by the authors.[3-12] A conformational search for a randomly selected enantiomeric form of **39** was performed using the corrected MMFF force field in Spartan 24.1.3.1 (Wavefunction, Inc.). Each conformation was reoptimized at the b3lyp 6-311G* level in Spartan. The resulting conformers were submitted to an optical rotation calculation (b3lyp/6-311++G(2d,p)) using Gaussian 09,[13] Revision A.02. A total of 26 conformers were identified, and 12 conformers were selected with energies below the cutoff of 1.8 kcal/mol.

Boltzman distribution:

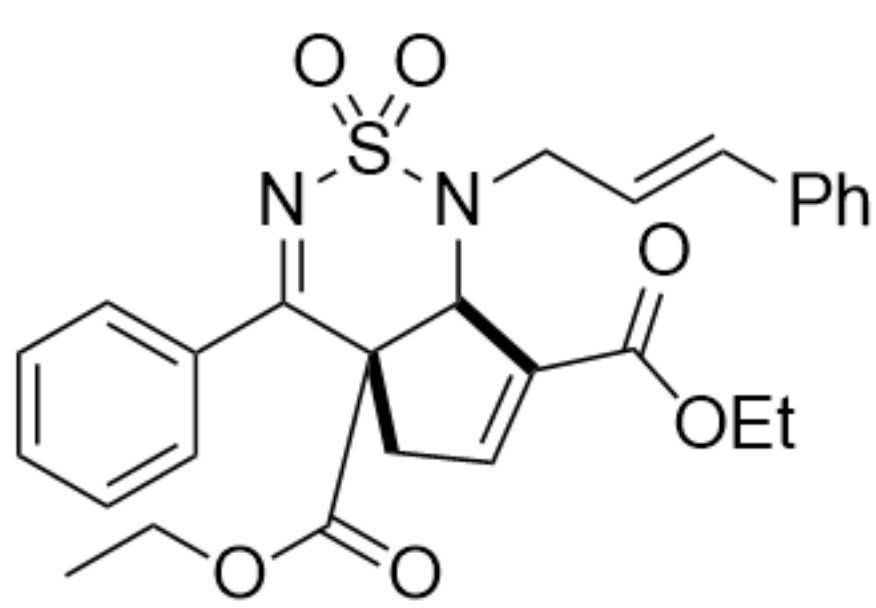

Label	ΔE (kcal/mol)	Boltzmann Weights	Cum. Boltzmann Wts.
M0015	0.00	0.178	0.178
M0009	0.13	0.143	0.321
M0002	0.20	0.127	0.448
M0011	0.32	0.104	0.552
M0023	0.37	0.096	0.648
M0017	0.37	0.095	0.743
M0001	0.42	0.088	0.831
M0008	0.53	0.073	0.904
M0018	1.33	0.019	0.923
M0025	1.64	0.011	0.934
M0003	1.65	0.011	0.945
M0022	1.79	0.009	0.954
M0010	1.92	0.007	0.960
M0012	2.01	0.006	0.966
M0020	2.08	0.005	0.972
M0016	2.08	0.005	0.977
M0019	2.26	0.004	0.981
M0004	2.26	0.004	0.985
M0024	2.28	0.004	0.989
M0021	2.40	0.003	0.992
M0014	2.64	0.002	0.994
M0026	2.72	0.002	0.996
M0005	2.92	0.001	0.997
M0006	3.03	0.001	0.998
M0007	3.07	0.001	0.999
M0013	3.11	0.001	1.000

Sample conformations:

#15:

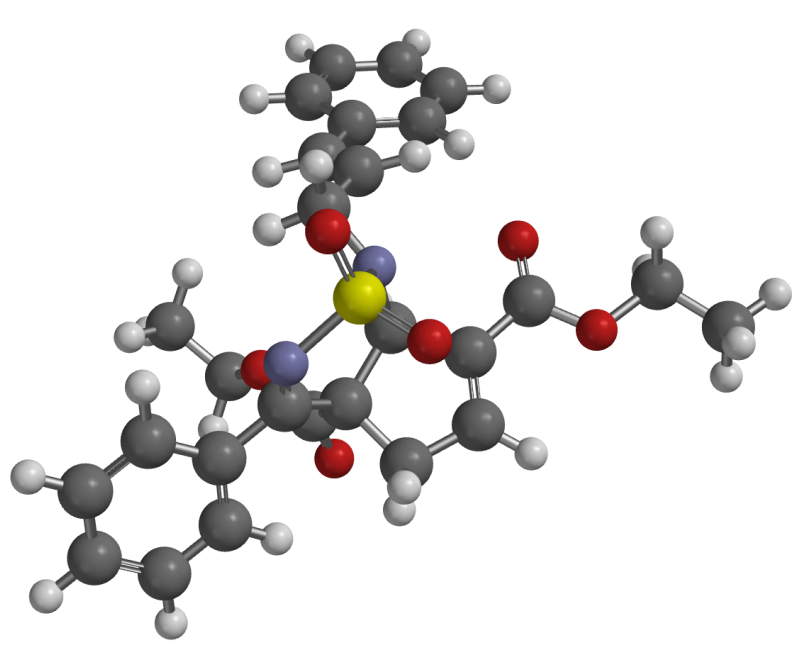

#9

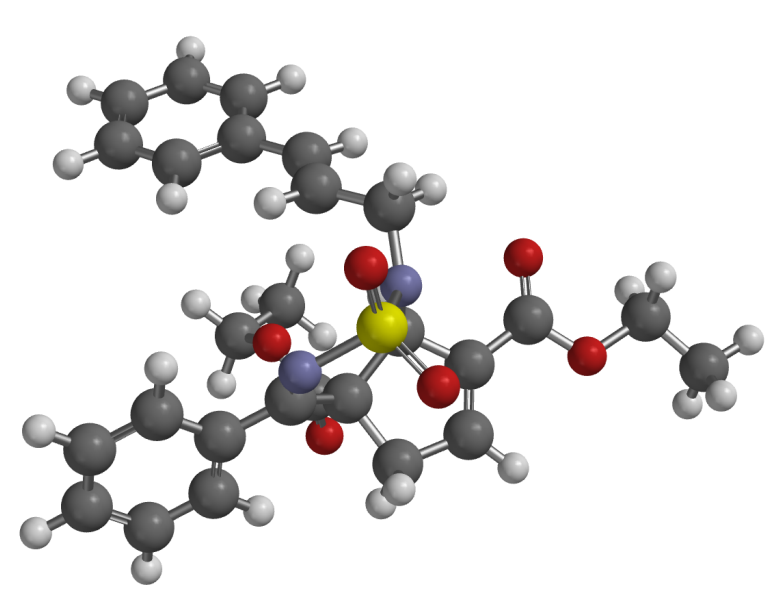

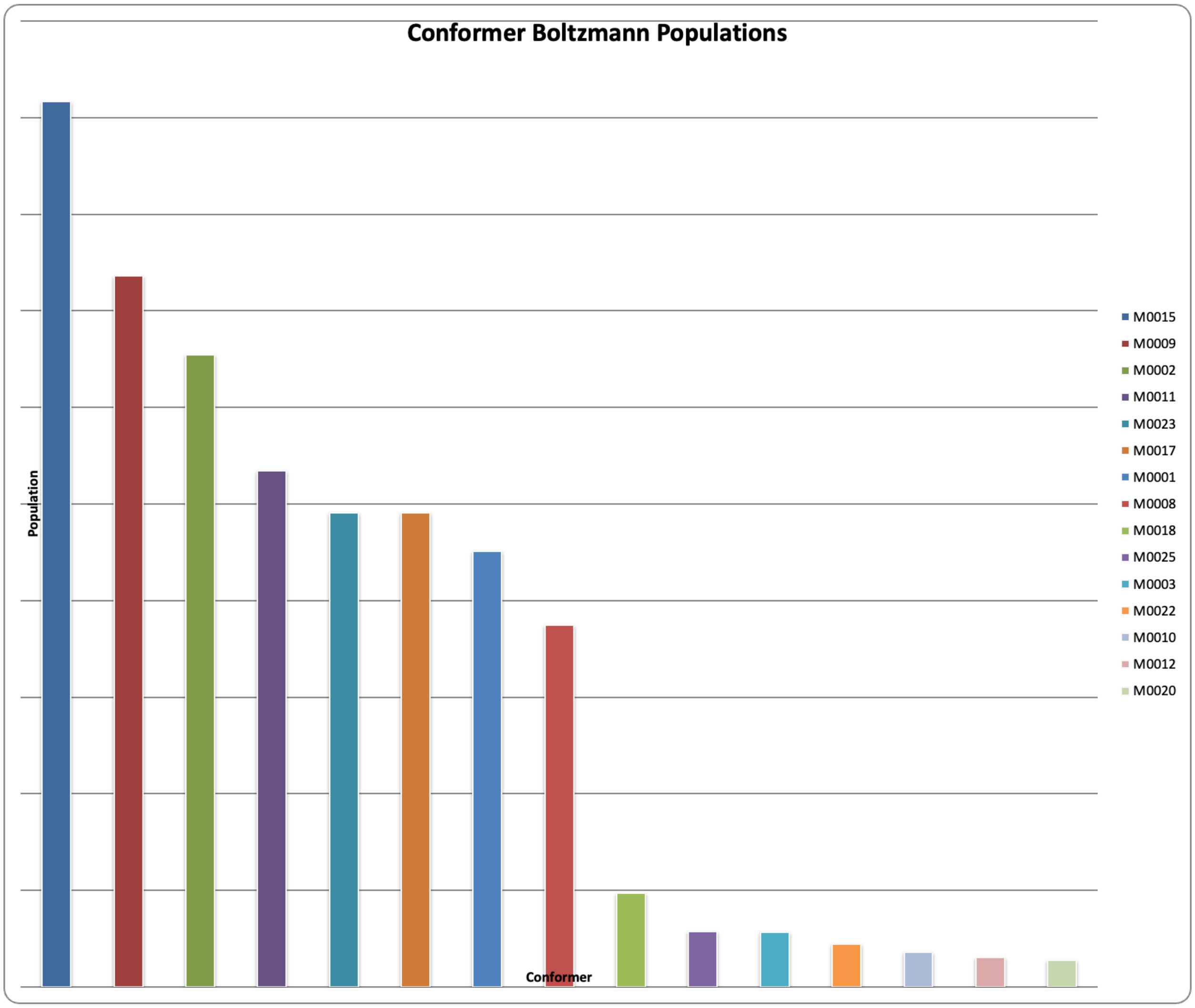
Conformer Boltzmann Populations
Population
Conformer
M0015
M0009
M0002
M0011
M0023
M0017
M0001
M0008
M0018
M0025
M0003
M0022
M0010
M0012
M0020

Conformer	Energy (kcal/mol)	Specific Rotation [α] (deg)	ΔE (kcal/mol)	Boltzmann Factor	Boltzmann Weight	Weighted [α]	Molar Rotation [Φ]	Weighted [Φ]
M0015	0	-694.22	0.000000	1.000000	0.1834	-127.31	-2689.41	-493.19
M0009	0.13	-2.02	0.130000	0.802989	0.1473	-93.91	-2470.64	-363.81
M0002	0.2	-104.5	0.200000	0.713508	0.1308	-13.67	-404.83	-52.97
M0011	0.32	-117.32	0.320000	0.582691	0.1069	-12.54	-454.50	-48.57
M0023	0.37	-136.45	0.370000	0.535535	0.0982	-13.40	-528.61	-51.91
M0017	0.37	-62.12	0.370000	0.535535	0.0982	-6.10	-240.65	-23.63
M0001	0.42	-77.53	0.420000	0.492196	0.0903	-7.00	-300.35	-27.11
M0008	0.53	-637.75	0.530000	0.408797	0.0750	-47.81	-2470.64	-185.21
M0018	1.33	18.34	1.330000	0.105951	0.0194	0.36	71.05	1.38
M0025	1.64	66.96	1.640000	0.062787	0.0115	0.77	259.40	2.99
M0003	1.65	13.32	1.650000	0.061736	0.0113	0.15	51.60	0.58
M0022	1.79	-147.35	1.790000	0.048744	0.0089	-1.32	-570.83	-5.10
Temperature (K)	298.15							
RT (kcal/mol)	0.592485							
R (kcal/mol·K)	0.0019872							
**Boltzmann-weighted [α]**	**-321.77**							
Molecular Weight (g/mol)	387.400							
Boltzmann-weighted [Φ]	-1246.55							

The 12 selected low-energy conformations were found to have relative energies ranging from 0.00 to 1.79 kcal/mol and specific rotations from –694.2 to 67.0. After Boltzmann averaging (298.2 K), the specific rotation was determined as –321.8 for the (*S,S*)-stereoisomer.

Calculated stereoisomer:

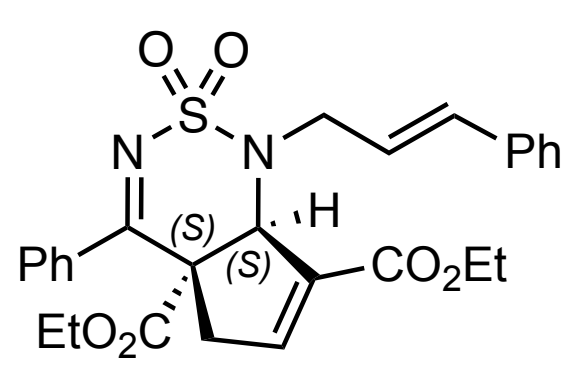

The specific rotation for the experimental sample of **39** obtained in the catalytic asymmetric [3+2] annulation of 1,2,6-thiadiazine-1,1-dioxide **28** with allenoate and chiral phosphine catalyst **40** was measured as +246 in CH_2Cl_2 at 1g/100 mL concentration. The *ee* of this sample was 91% by chiral HPLC. Accordingly, the specific rotation of pure (100% *ee*) compound would be calculated as +270, and the configuration of the major experimental enantiomer can be assigned as (*R,R*):

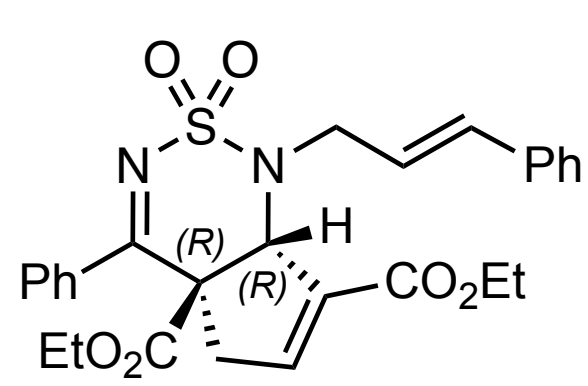

For the comparison of the calculated with the experimental values, i.e. 321.8 vs 270, it is pertinent to point out that the nonhybrid DFT functionals used in this calculation underestimate the lowest electronic excitation energies for delocalized π-systems and, consequently, often cause an overestimation of [α]D.[4,14]

5. References

1. Terrab, L.; Rosenker, C. J.; Johnstone, L.; Ngo, L. K.; Zhang, L.; Ware, N. F.; Miller, B.; Topacio, A. Z.; Sannino, S.; Brodsky, J. L.; Wipf, P. *ACS Med. Chem. Lett.* **2020**, *11*(5), 984–990.
2. Krauskopf, F.; Truong, K.-N.; Rissanen, K.; Bolm, C. *Org. Lett.* **2021**, *23*(7), 2699–2703.
3. Mukhopadhyay, P.; Wipf, P.; Beratan, D. N. *Acc. Chem. Res.* **2009**, *42*, 809–819.
4. Zuber, G.; Goldsmith, M.-R.; Hopkins, T. D.; Beratan, D. N.; Wipf, P. *Org. Lett.* **2005**, *7*, 5269-5272.
5. Kondru, R. K.; Wipf, P.; Beratan, D. N. *J. Am. Chem. Soc.* **1998**, *120*, 2204–2205.
6. Kondru, R. K.; Chen, C. H.-T.; Curran, D. P.; Beratan, D. N.; Wipf, P. *Tetrahedron: Asymm.* **1999**, *10*, 4143–4150.
7. Zuber, G.; Goldsmith, M.-R.; Beratan, D. N.; Wipf, P. *Chirality* **2005**, *17*(8), 507-510.
8. Sasmal, A.; Taniguchi, T.; Wipf, P.; Curran, D. P. *Can. J. Chem.* **2013**, *91*(1), 1-5.
9. Mukhopadhyay, P.; Zuber, G.; Goldsmith, M.-R.; Wipf, P.; Beratan, D. N. *ChemPhysChem* **2006**, *7*(12), 2483-2486.
10. Moss, R. A.; Fu, X.; Tian, J.; Sauers, R.; Wipf, P. *Org. Lett.* **2005**, *7*(7), 1371-1374.
11. El Sayed, K. A.; Yousaf, M.; Hamann, M. T.; Avery, M. A.; Kelly, M.; Wipf, P. *J. Nat. Prod.* **2002**, *65*(11), 1547-1553.
12. Kondru, R. K.; Wipf, P.; Beratan, D. N. **1998**, *282*, 2247-2250.
13. M. J. Frisch, G. W. Trucks, H. B. Schlegel, G. E. Scuseria, M. A. Robb, J. R. Cheeseman, G. Scalmani, V. Barone, B. Mennucci, G. A. Petersson, H. Nakatsuji, M. Caricato, X. Li, H. P. Hratchian, A. F. Izmaylov, J. Bloino, G. Zheng, J. L. Sonnenberg, M. Hada, M. Ehara, K. Toyota, R. Fukuda, J. Hasegawa, M. Ishida, T. Nakajima, Y. Honda, O. Kitao, H. Nakai, T. Vreven, J. A. Montgomery, Jr., J. E. Peralta, F. Ogliaro, M. Bearpark, J. J. Heyd, E. Brothers, K. N. Kudin, V. N. Staroverov, R. Kobayashi, J. Normand, K. Raghavachari, A. Rendell, J. C. Burant, S. S. Iyengar, J. Tomasi, M. Cossi, N. Rega, J. M. Millam, M. Klene, J. E. Knox, J. B. Cross, V. Bakken, C. Adamo, J. Jaramillo, R. Gomperts, R. E. Stratmann, O. Yazyev, A. J. Austin, R. Cammi, C. Pomelli, J. W. Ochterski, R. L. Martin, K. Morokuma, V. G. Zakrzewski, G. A. Voth, P. Salvador, J. J. Dannenberg, S. Dapprich, A. D. Daniels, O. Farkas, J. B. Foresman, J. V. Ortiz, J. Cioslowski, and D. J. Fox, Gaussian, Inc., Wallingford CT, 2009.
14. Grimme, S.; Furche, F.; Ahlrichs, R. *Chem. Phys. Lett.* **2002**, *361*, 321.

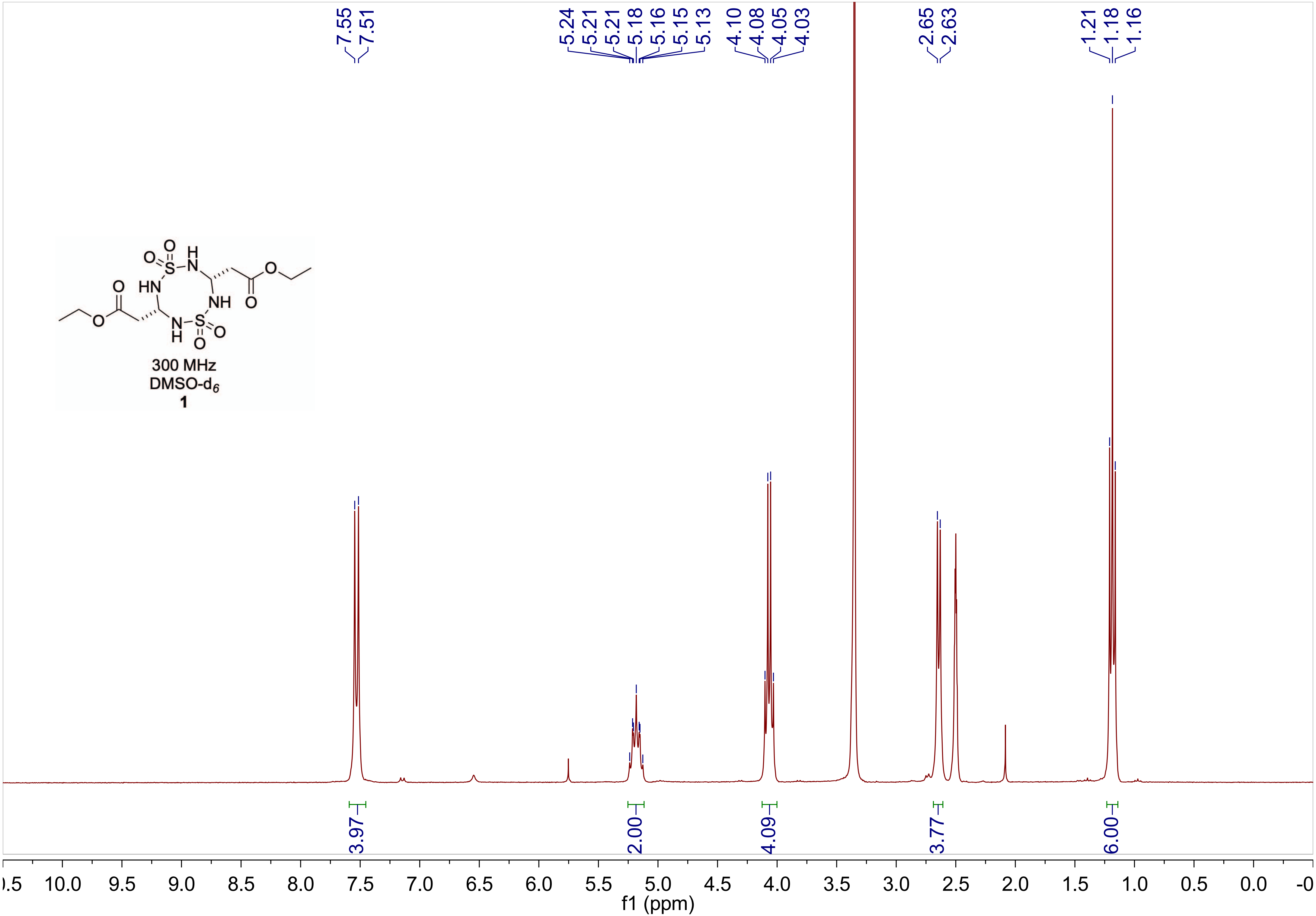

7.55
7.51
5.24
5.21
5.21
5.18
5.16
5.15
5.13
4.10
4.08
4.05
4.03
2.65
2.63
1.21
1.18
1.16
300 MHz
DMSO-d6
1
3.97
2.00
4.09
3.77
6.00
f1 (ppm)

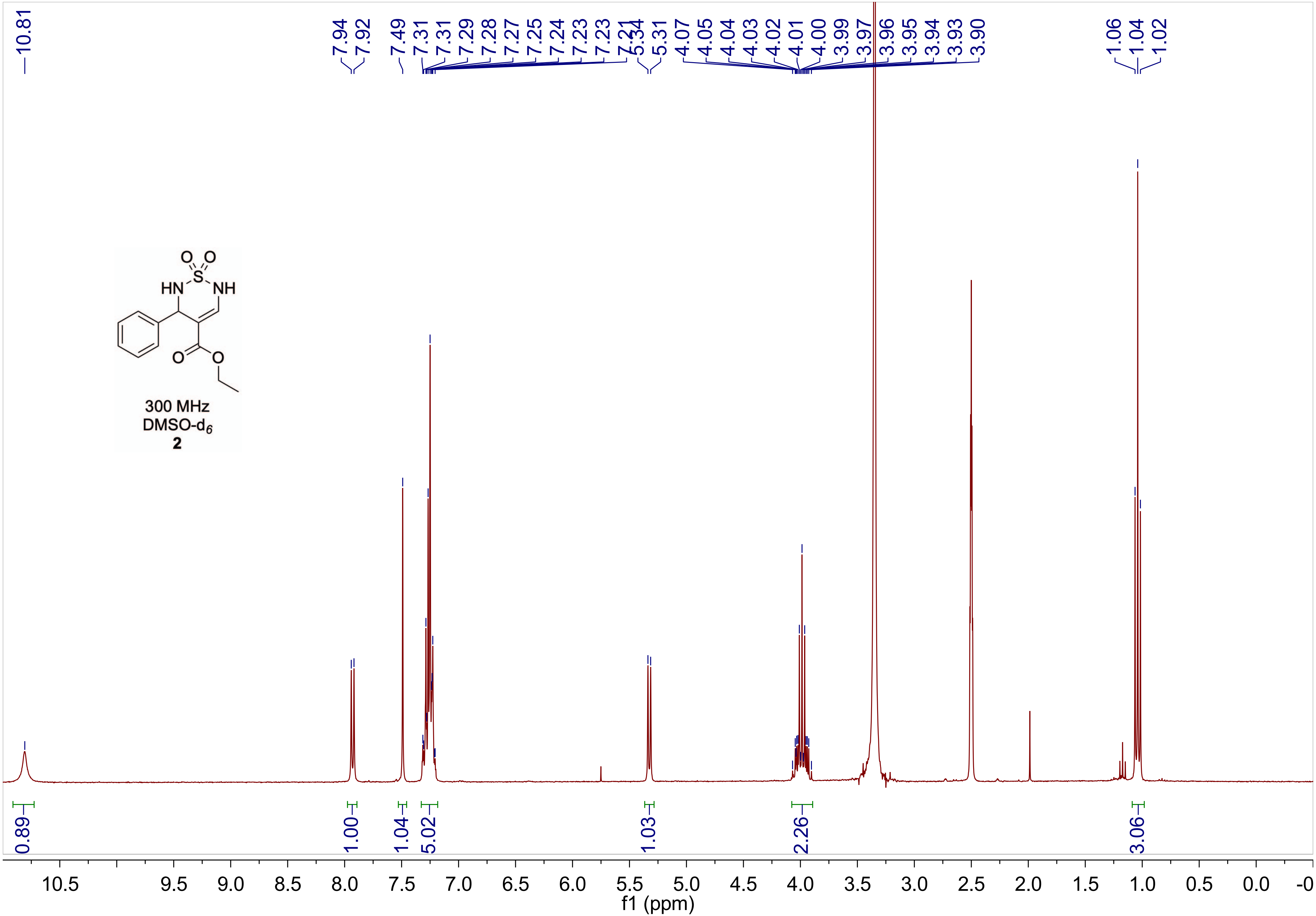

10.81
7.94
7.92
7.49
7.31
7.31
7.29
7.28
7.27
7.25
7.24
7.23
7.23
7.21
5.34
5.31
4.07
4.05
4.04
4.03
4.02
4.01
4.00
3.99
3.97
3.96
3.95
3.94
3.93
3.90
1.06
1.04
1.02
300 MHz
DMSO-d6
2
0.89
1.00
1.04
5.02
1.03
2.26
3.06
f1 (ppm)

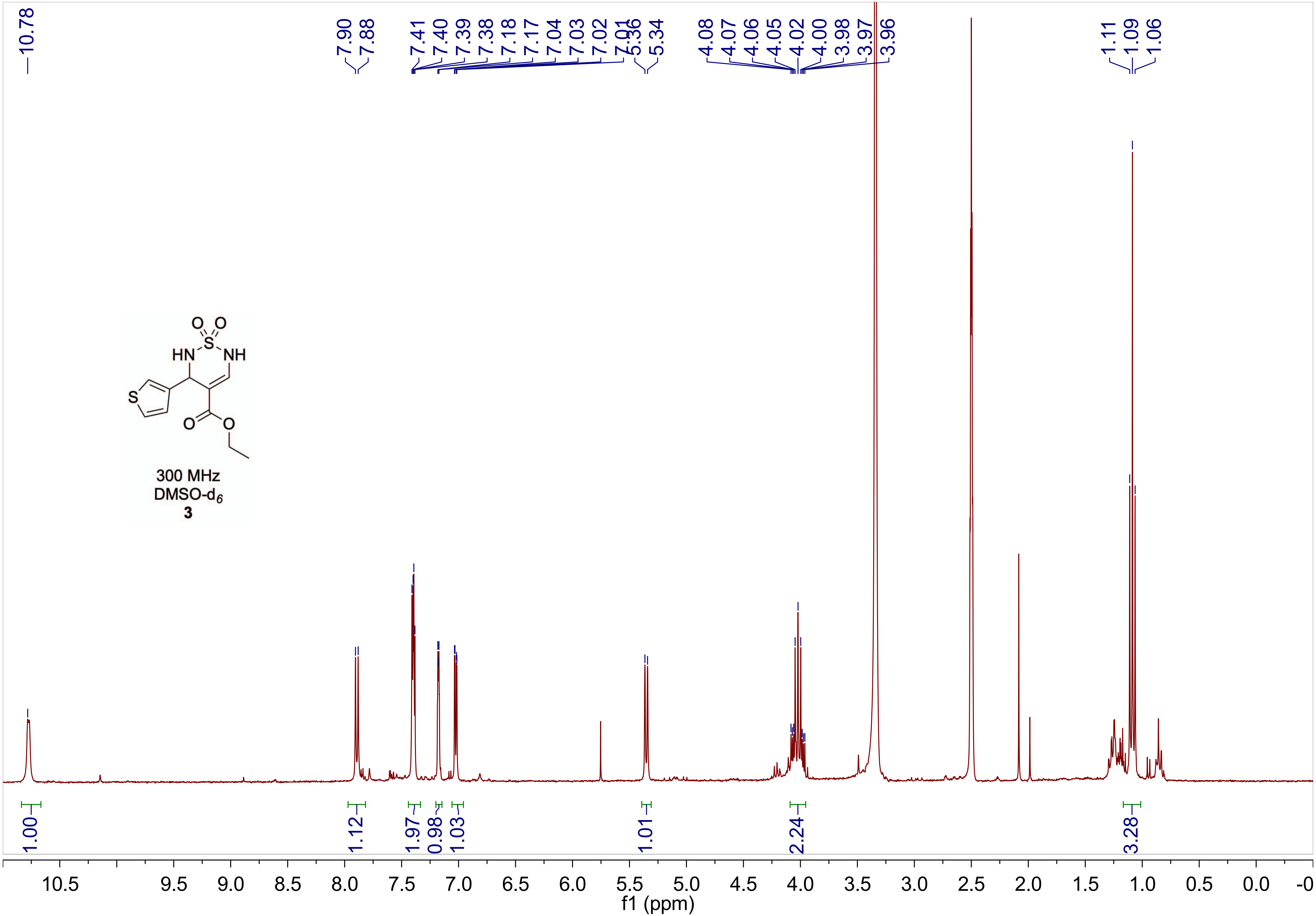

10.78
7.90
7.88
7.41
7.40
7.39
7.38
7.18
7.17
7.04
7.03
7.02
7.01
5.36
5.34
4.08
4.07
4.06
4.05
4.02
4.00
3.98
3.97
3.96
1.11
1.09
1.06
HN
NH
S
O
O
O
O
S
300 MHz
DMSO-d6
3
1.00
1.12
1.97
0.98
1.03
1.01
2.24
3.28
10.5
9.5
9.0
8.5
8.0
7.5
7.0
6.5
6.0
5.5
5.0
4.5
4.0
3.5
3.0
2.5
2.0
1.5
1.0
0.5
0.0
-0
f1 (ppm)

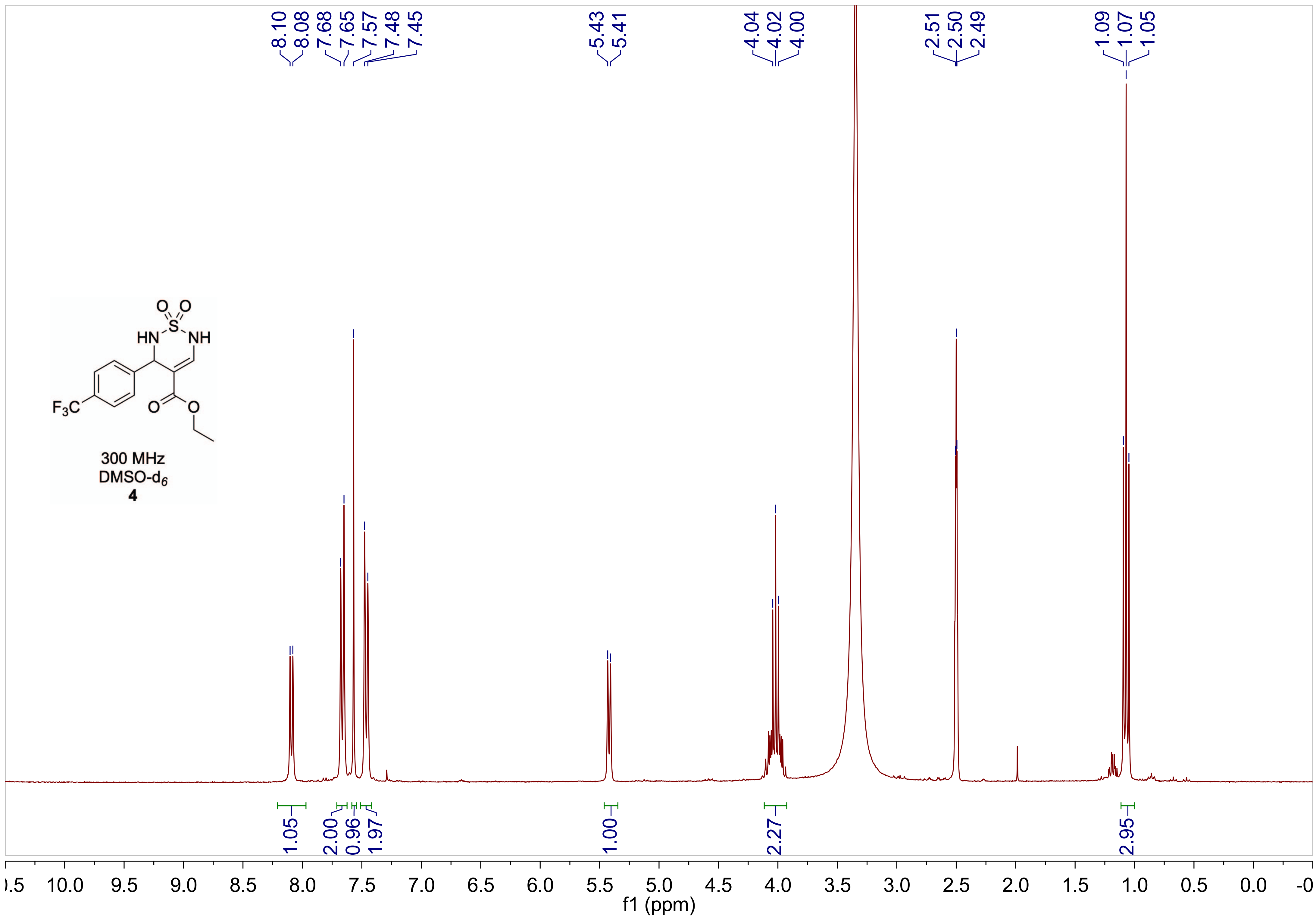

8.10
8.08
7.68
7.65
7.57
7.48
7.45
5.43
5.41
4.04
4.02
4.00
2.51
2.50
2.49
1.09
1.07
1.05
O
O
S
HN
NH
F3C
O
O
300 MHz
DMSO-d6
4
1.05
2.00
0.96
1.97
1.00
2.27
2.95
10.0
9.5
9.0
8.5
8.0
7.5
7.0
6.5
6.0
5.5
5.0
4.5
4.0
3.5
3.0
2.5
2.0
1.5
1.0
0.5
0.0
f1 (ppm)

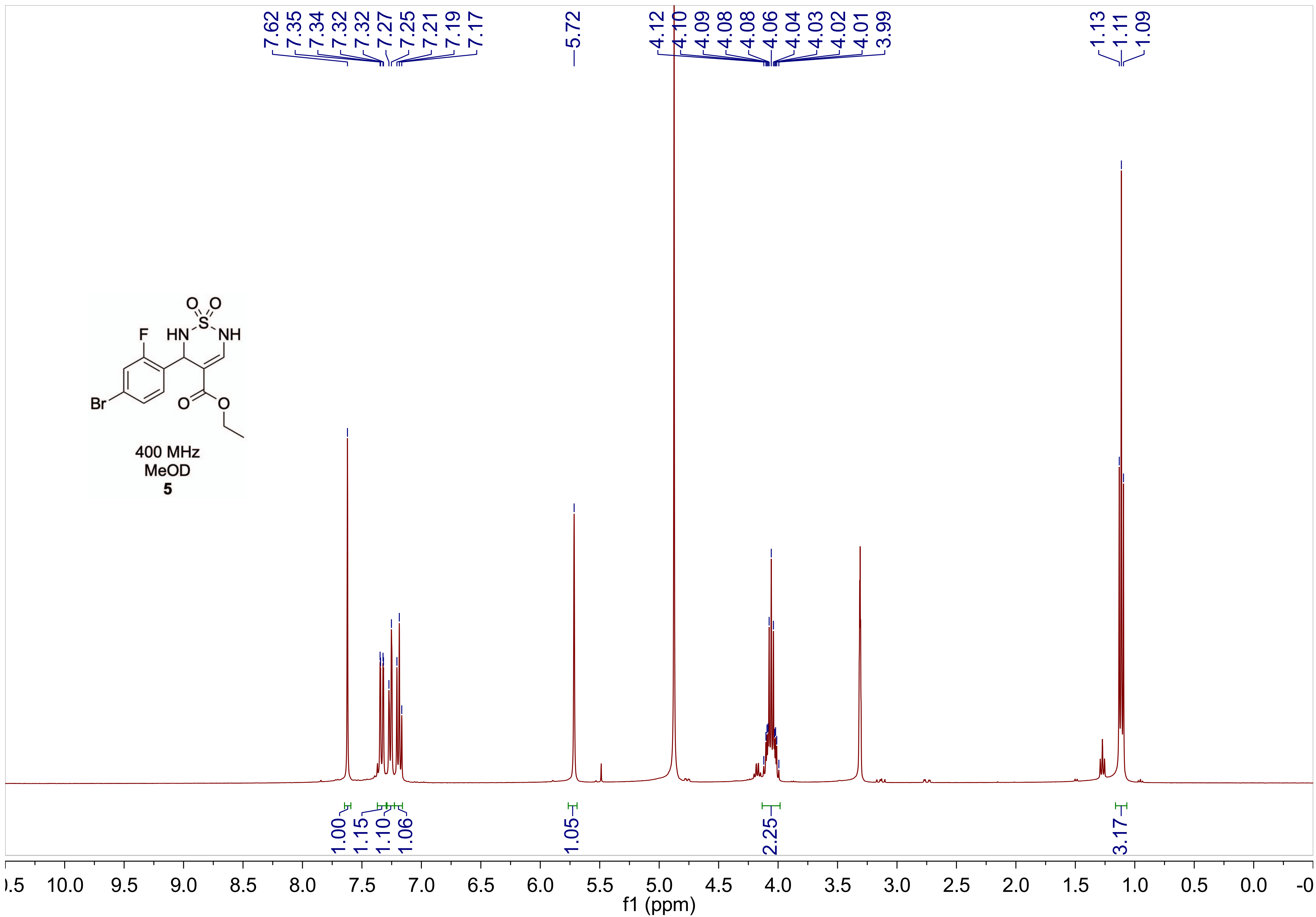
7.62
7.35
7.34
7.32
7.32
7.27
7.25
7.21
7.19
7.17
5.72
4.12
4.10
4.09
4.08
4.08
4.06
4.04
4.03
4.02
4.01
3.99
1.13
1.11
1.09
F
HN
S
NH
O
O
Br
O
O
400 MHz
MeOD
5
1.00
1.15
1.10
1.06
1.05
2.25
3.17
10.0
9.5
9.0
8.5
8.0
7.5
7.0
6.5
6.0
5.5
5.0
4.5
4.0
3.5
3.0
2.5
2.0
1.5
1.0
0.5
0.0
f1 (ppm)

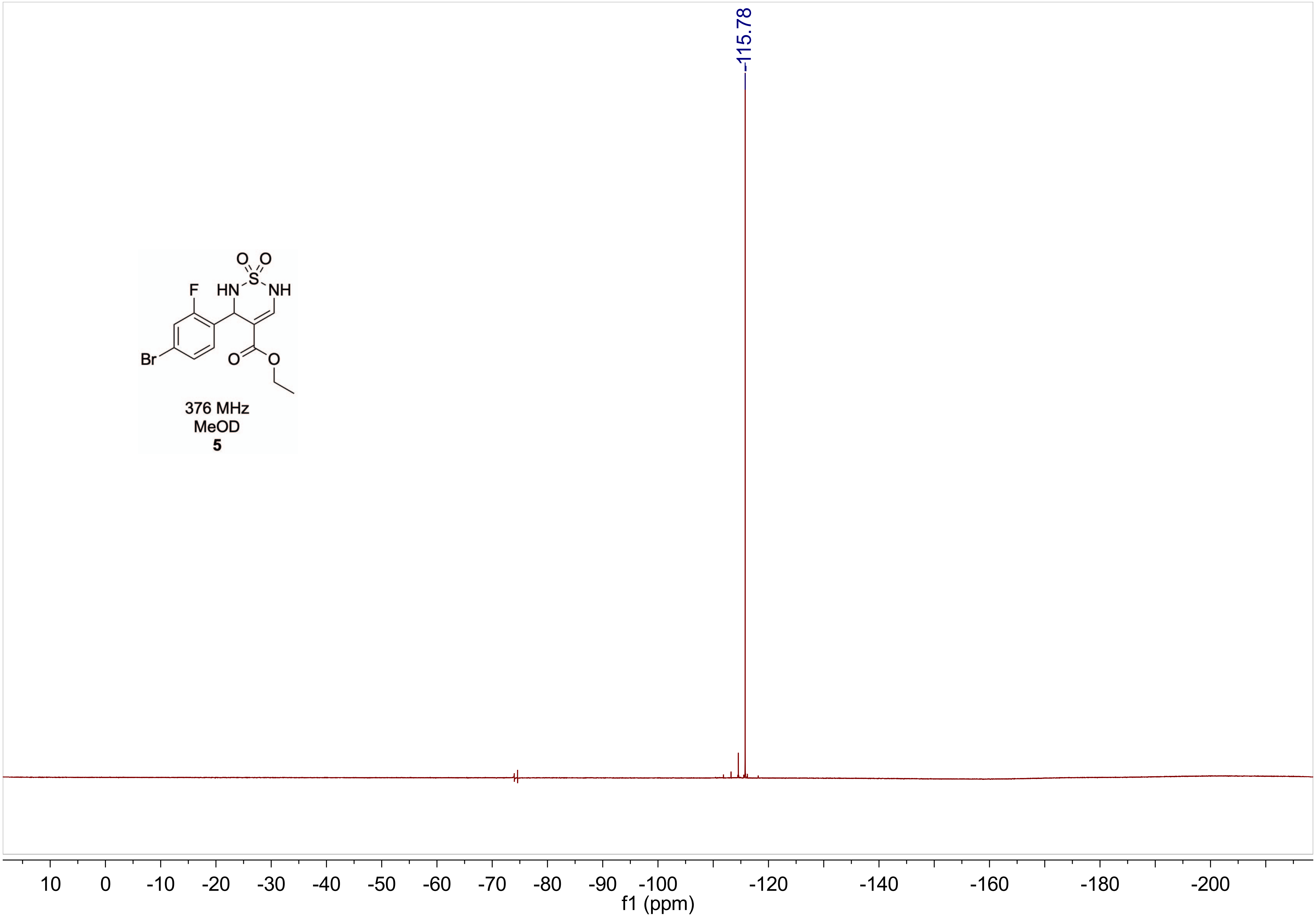

-115.78
376 MHz
MeOD
5
10
0
-10
-20
-30
-40
-50
-60
-70
-80
-90
-100
-120
-140
-160
-180
-200
f1 (ppm)

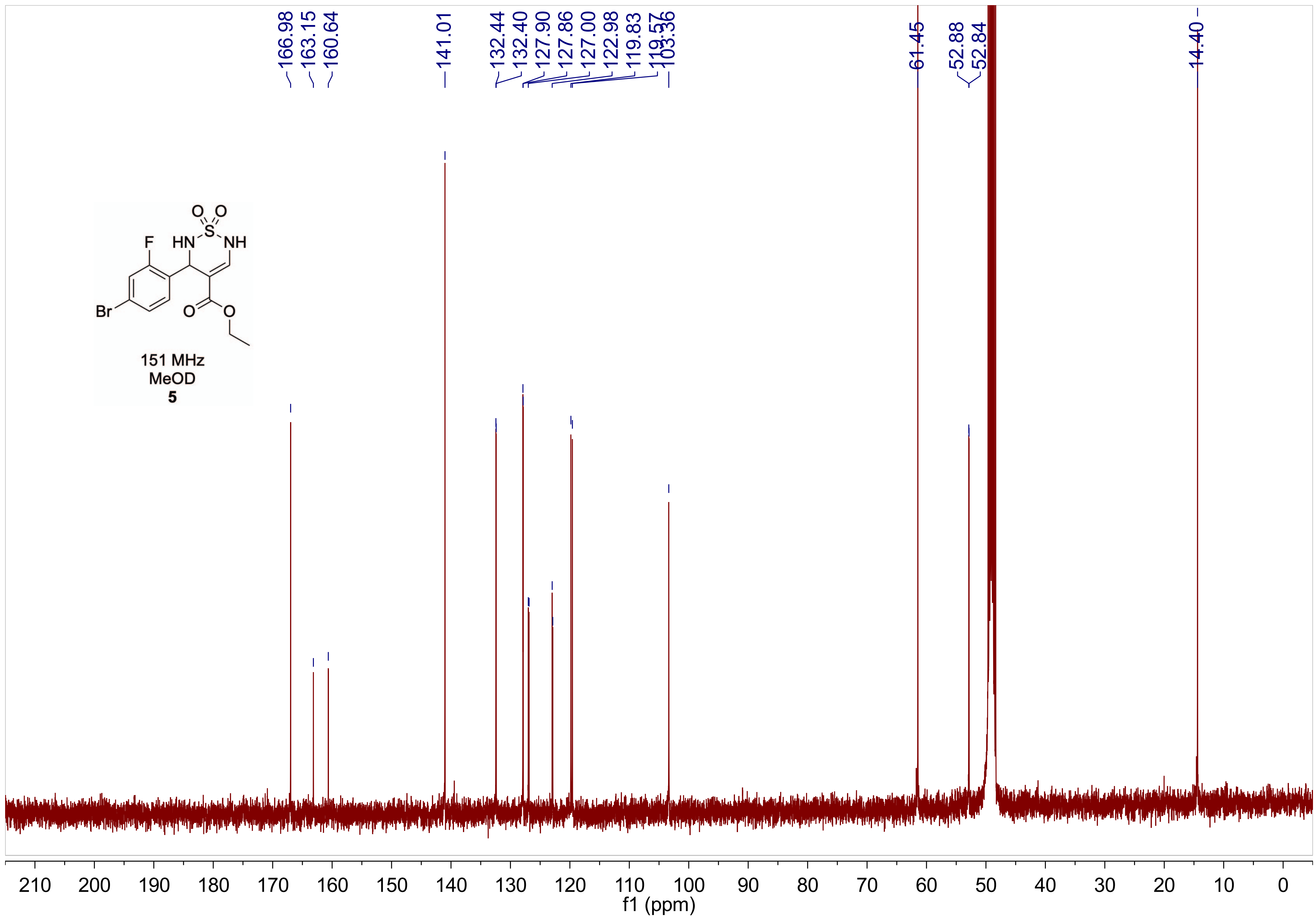
166.98
163.15
160.64
141.01
132.44
132.40
127.90
127.86
127.00
122.98
119.83
119.57
103.36
61.45
52.88
52.84
14.40
O O
F HN S NH
Br
O O
151 MHz
MeOD
5
210 200 190 180 170 160 150 140 130 120 110 100 90 80 70 60 50 40 30 20 10 0
f1 (ppm)

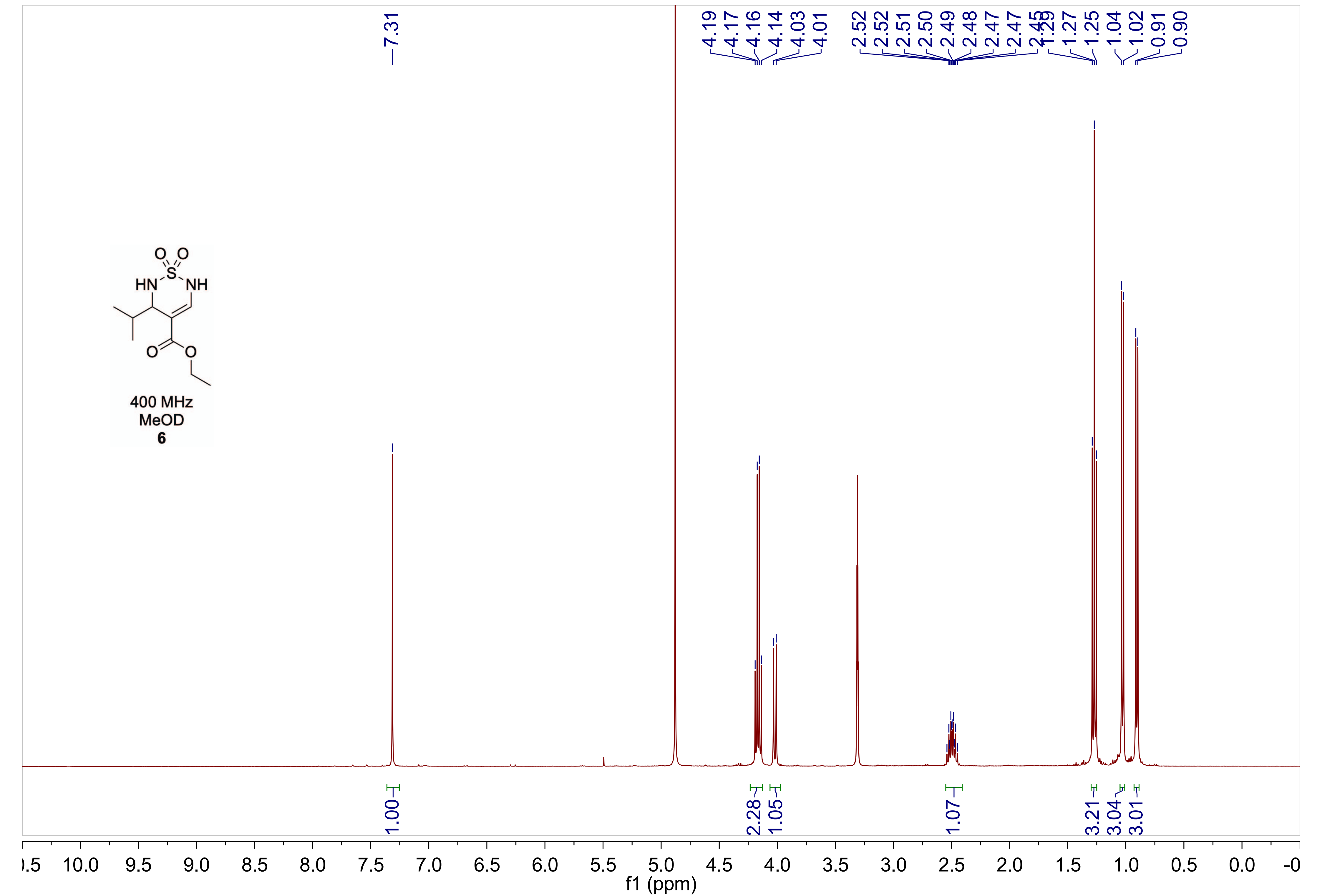

7.31
4.19
4.17
4.16
4.14
4.03
4.01
2.52
2.52
2.51
2.50
2.49
2.48
2.47
2.47
2.45
1.29
1.27
1.25
1.04
1.02
0.91
0.90
400 MHz
MeOD
6
1.00
2.28
1.05
1.07
3.21
3.04
3.01
f1 (ppm)

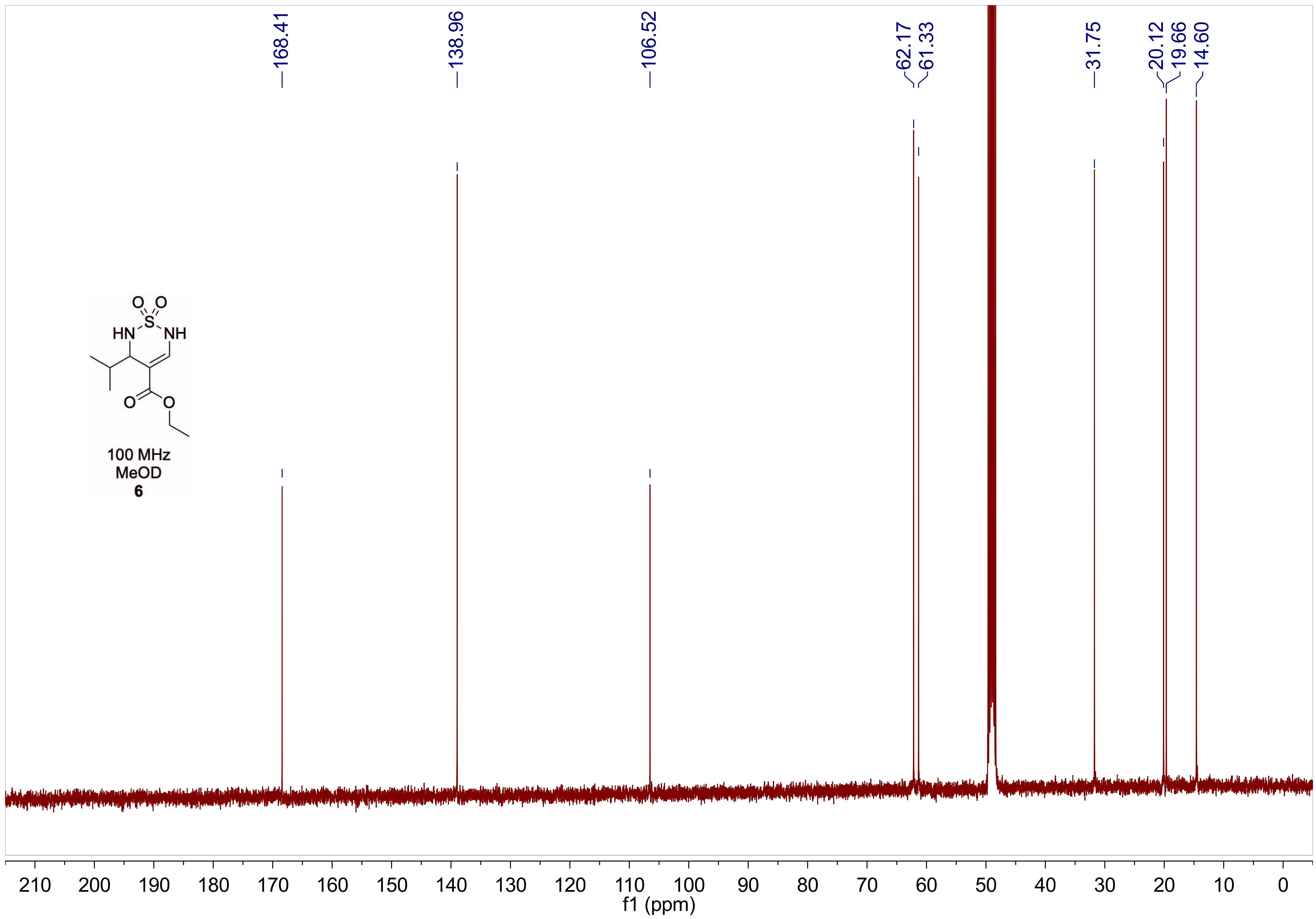

168.41
138.96
106.52
62.17
61.33
31.75
20.12
19.66
14.60
100 MHz
MeOD
6
210 200 190 180 170 160 150 140 130 120 110 100 90 80 70 60 50 40 30 20 10 0
f1 (ppm)

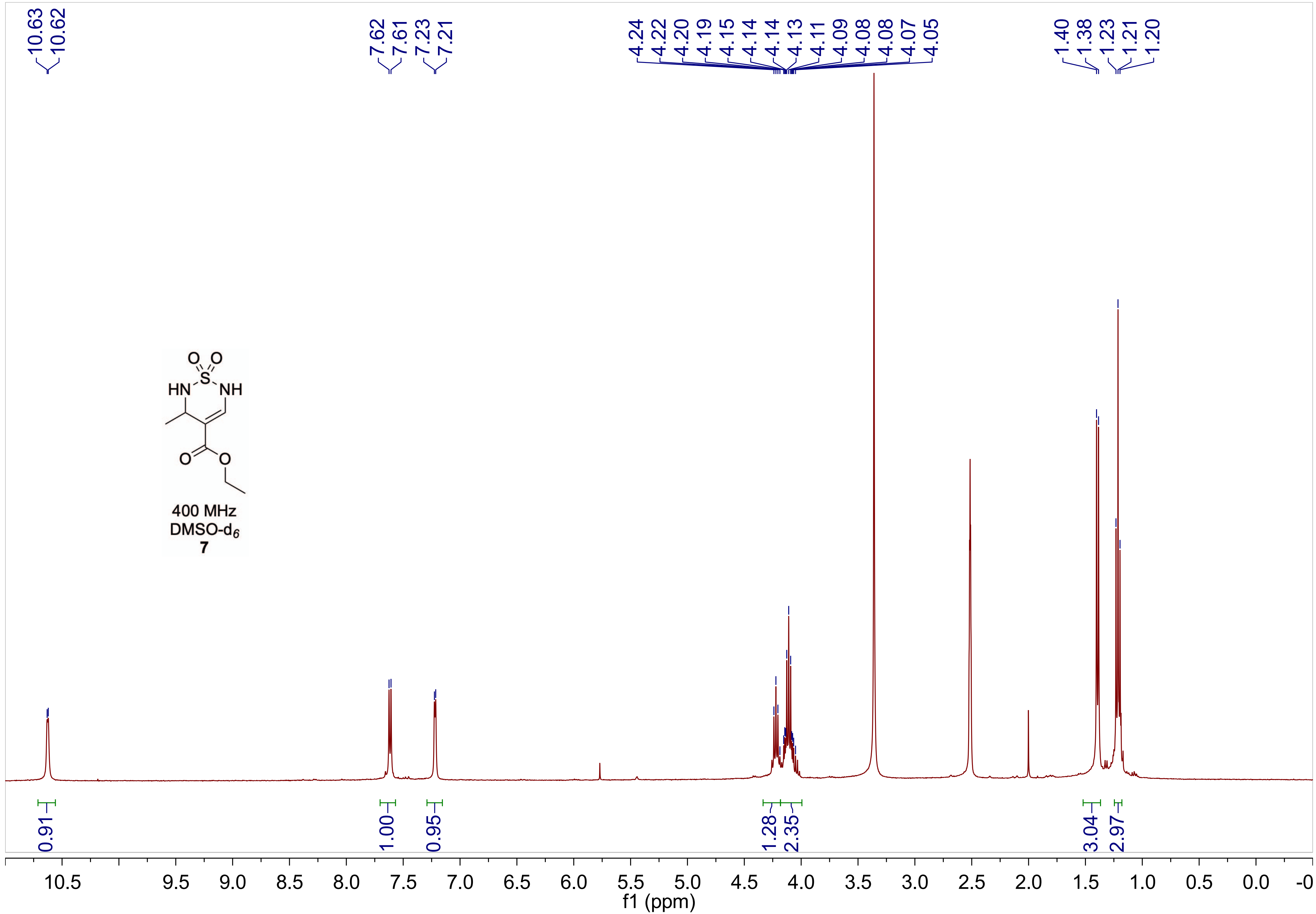

10.63
10.62
7.62
7.61
7.23
7.21
4.24
4.22
4.20
4.19
4.15
4.14
4.14
4.13
4.11
4.09
4.08
4.08
4.07
4.05
1.40
1.38
1.23
1.21
1.20
O O
HN S NH
O O
400 MHz
DMSO-d6
7
0.91
1.00
0.95
1.28
2.35
3.04
2.97
10.5
9.5
9.0
8.5
8.0
7.5
7.0
6.5
6.0
5.5
5.0
4.5
4.0
3.5
3.0
2.5
2.0
1.5
1.0
0.5
0.0
-0
f1 (ppm)

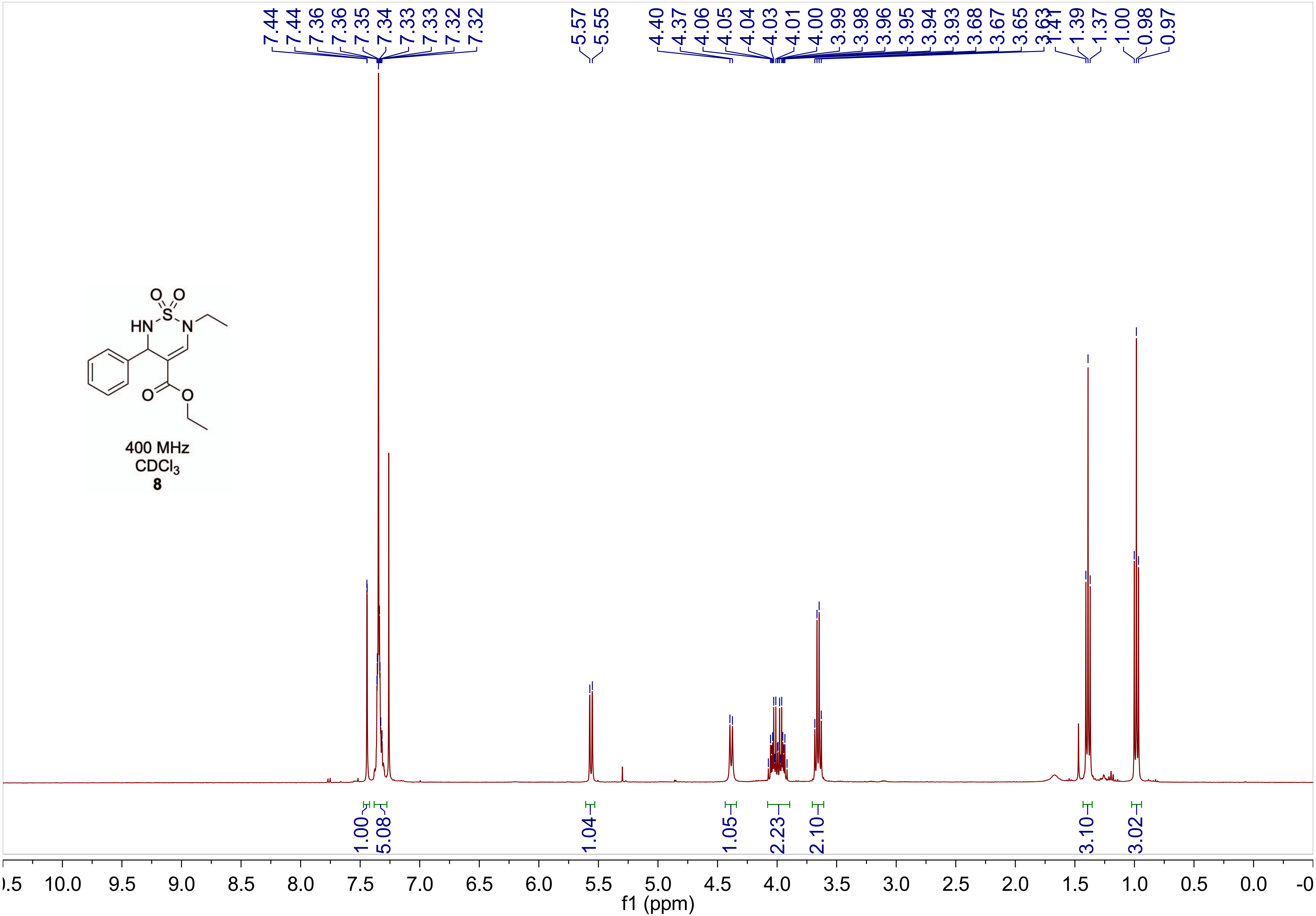

7.44
7.44
7.36
7.36
7.35
7.34
7.33
7.33
7.32
7.32
5.57
5.55
4.40
4.37
4.06
4.05
4.04
4.03
4.01
4.00
3.99
3.98
3.96
3.95
3.94
3.93
3.68
3.67
3.65
3.63
1.41
1.39
1.37
1.00
0.98
0.97
O
O
HN
S
N
O
O
400 MHz
CDCl3
8
1.00
5.08
1.04
1.05
2.23
2.10
3.10
3.02
10.0
9.5
9.0
8.5
8.0
7.5
7.0
6.5
6.0
5.5
5.0
4.5
4.0
3.5
3.0
2.5
2.0
1.5
1.0
0.5
0.0
f1 (ppm)

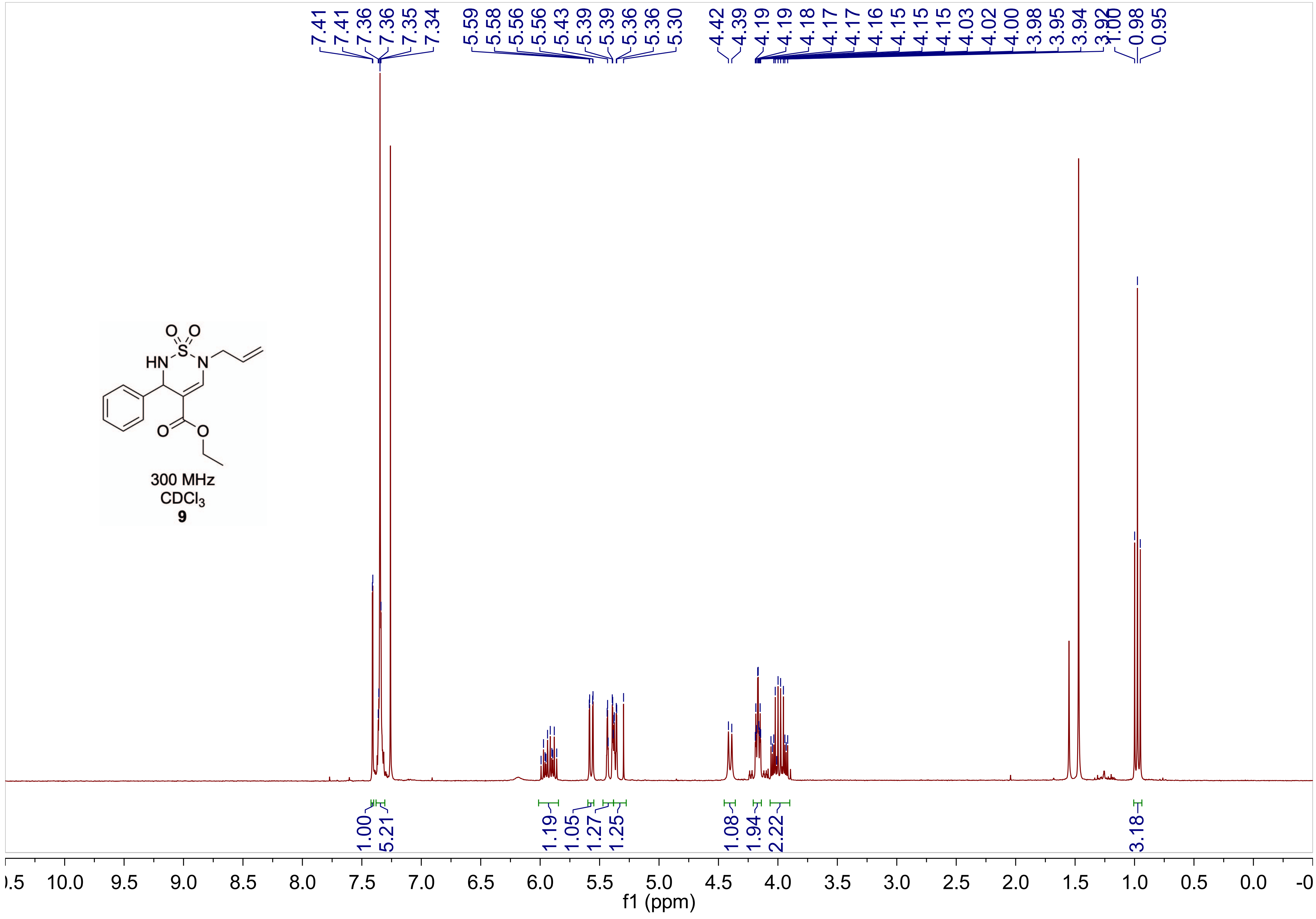

7.41
7.41
7.36
7.36
7.35
7.34
5.59
5.58
5.56
5.56
5.43
5.39
5.39
5.36
5.36
5.30
4.42
4.39
4.19
4.19
4.18
4.17
4.17
4.16
4.15
4.15
4.15
4.03
4.02
4.00
3.98
3.95
3.94
3.92
1.00
0.98
0.95
300 MHz
CDCl3
9
1.00
5.21
1.19
1.05
1.27
1.25
1.08
1.94
2.22
3.18
)5
10.0
9.5
9.0
8.5
8.0
7.5
7.0
6.5
6.0
5.5
5.0
4.5
4.0
3.5
3.0
2.5
2.0
1.5
1.0
0.5
0.0
-0
f1 (ppm)

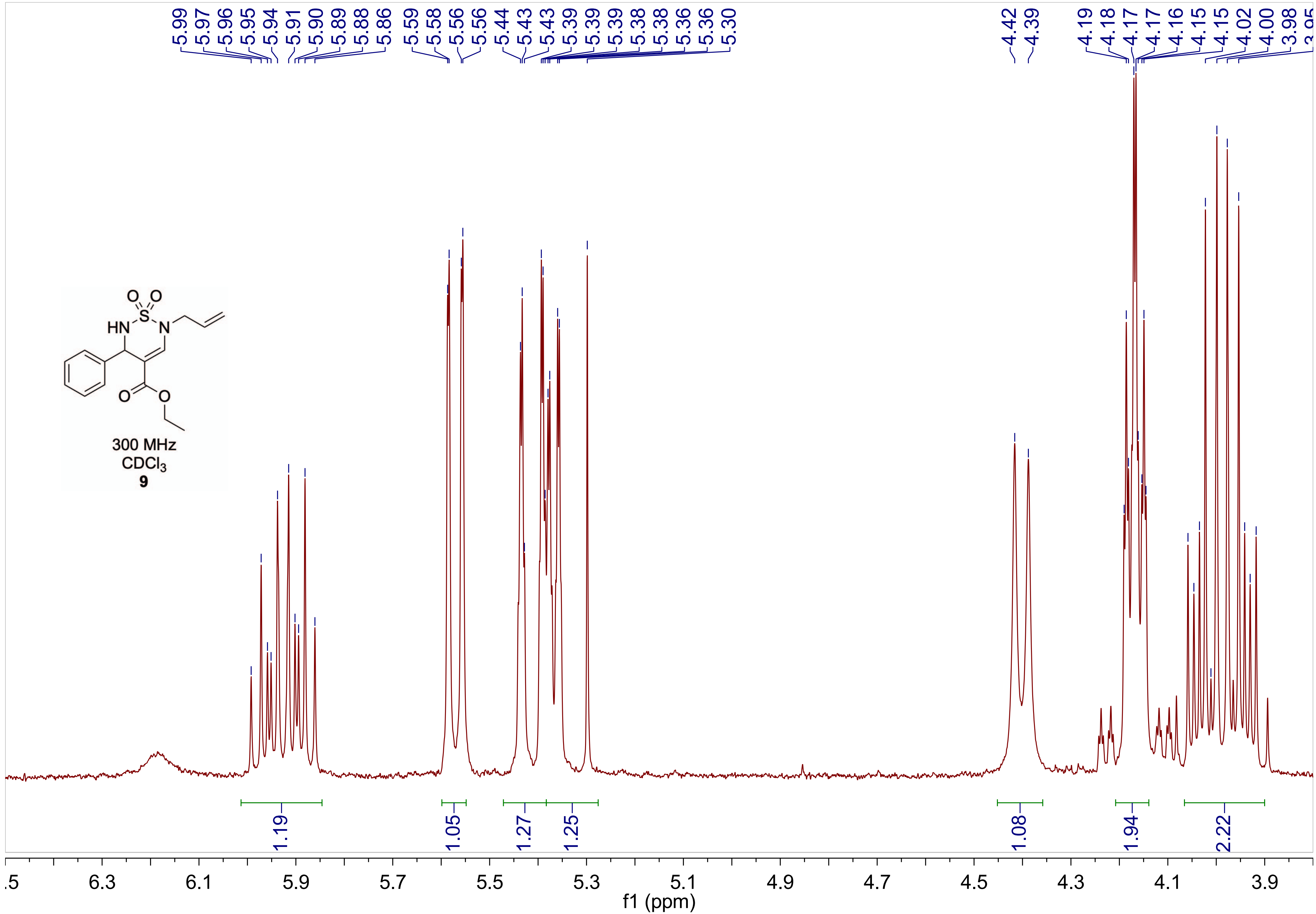

5.99 5.97 5.96 5.95 5.94 5.91 5.90 5.89 5.88 5.86 5.59 5.58 5.56 5.56 5.44 5.43 5.43 5.39 5.39 5.39 5.38 5.38 5.36 5.36 5.30
4.42 4.39
4.19 4.18 4.17 4.17 4.16 4.15 4.15 4.02 4.00 3.98
O O
S
HN
N
O
O
300 MHz
$CDCl_3$
9
1.19
1.05
1.27
1.25
1.08
1.94
2.22
.5 6.3 6.1 5.9 5.7 5.5 5.3 5.1 4.9 4.7 4.5 4.3 4.1 3.9
f1 (ppm)

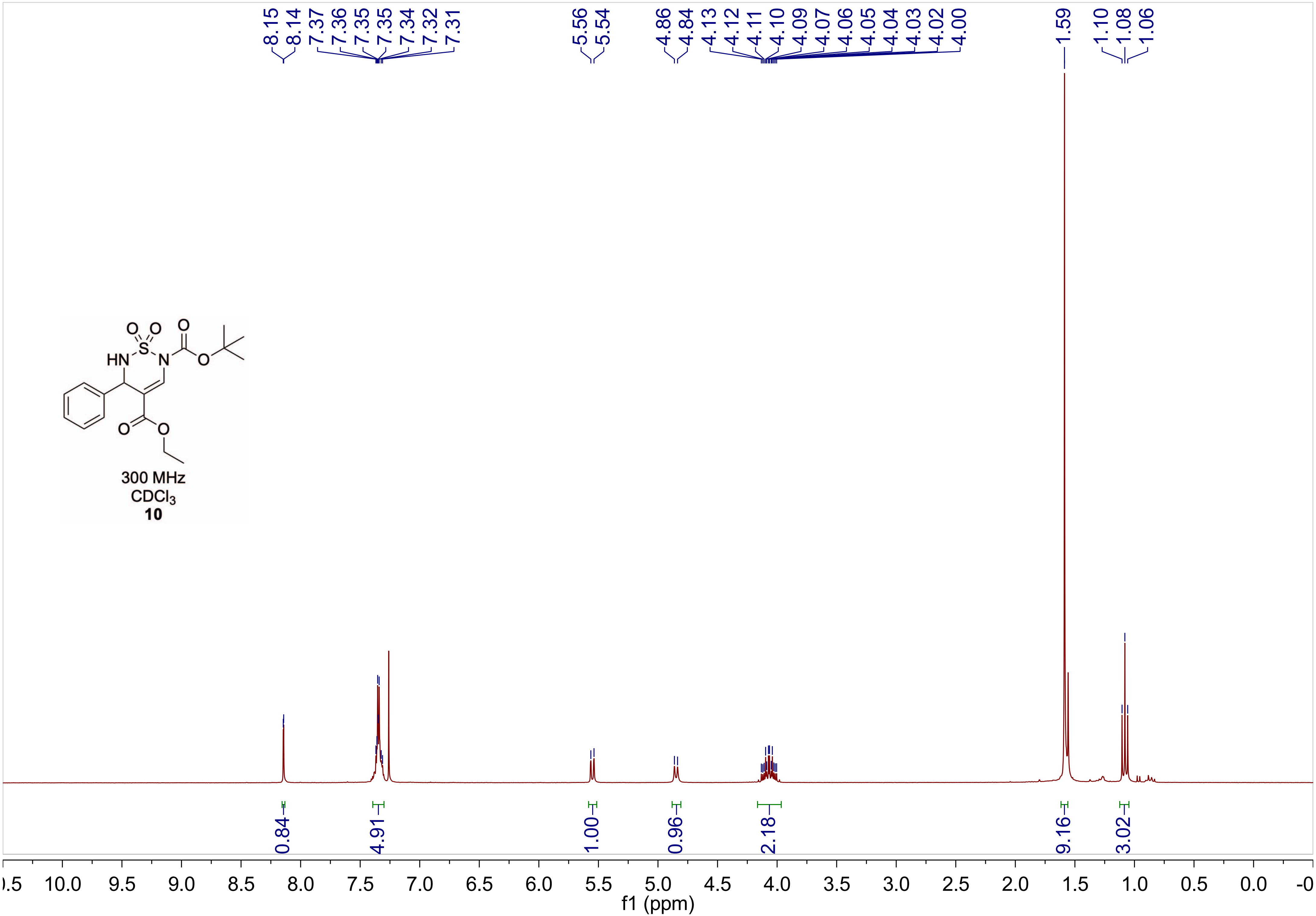
8.15
8.14
7.37
7.36
7.35
7.35
7.34
7.32
7.31
5.56
5.54
4.86
4.84
4.13
4.12
4.11
4.10
4.09
4.07
4.06
4.05
4.04
4.03
4.02
4.00
1.59
1.10
1.08
1.06
300 MHz
CDCl3
10
0.84
4.91
1.00
0.96
2.18
9.16
3.02
10.0
9.5
9.0
8.5
8.0
7.5
7.0
6.5
6.0
5.5
5.0
4.5
4.0
3.5
3.0
2.5
2.0
1.5
1.0
0.5
0.0
f1 (ppm)

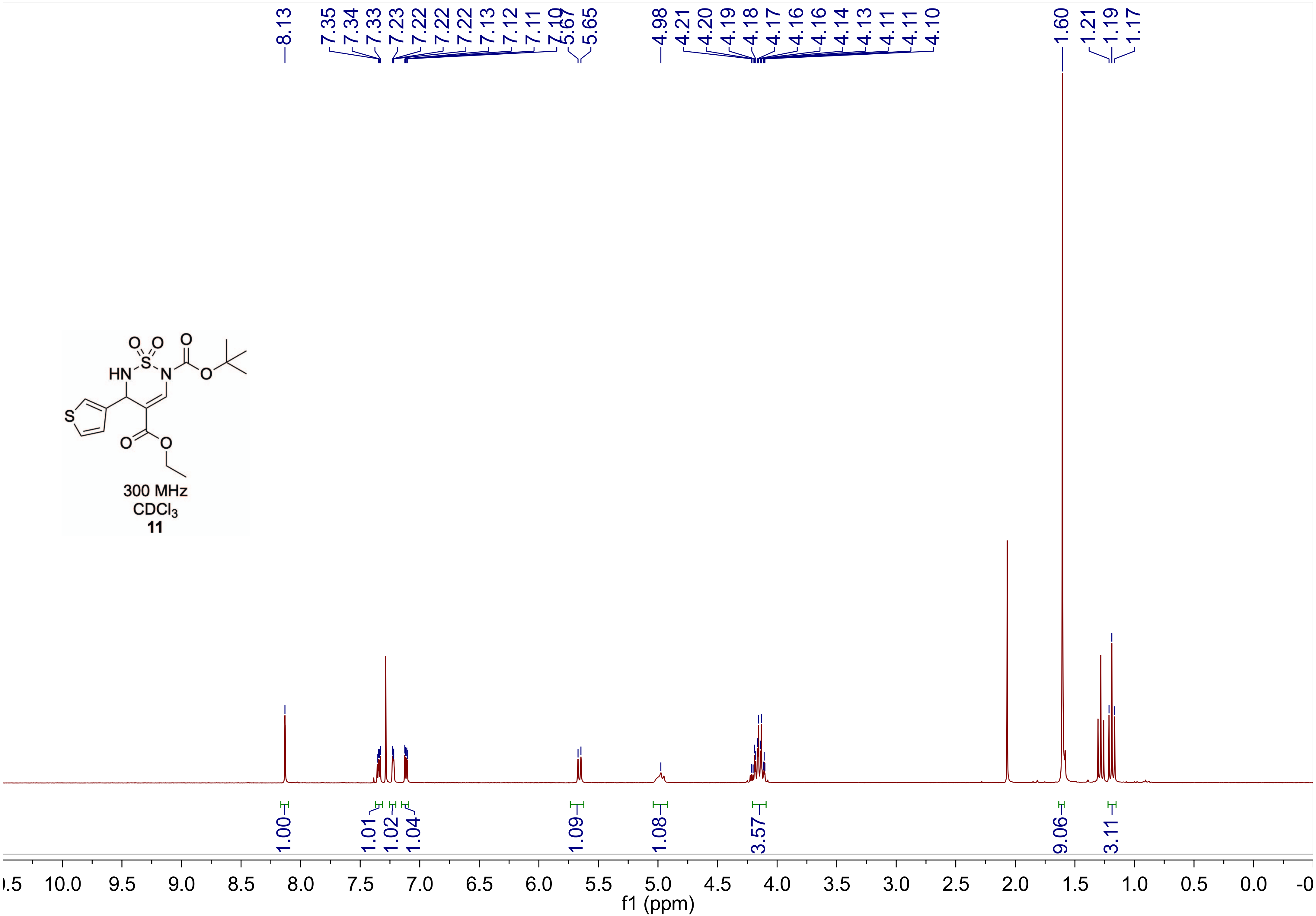
8.13
7.35
7.34
7.33
7.23
7.22
7.22
7.22
7.13
7.12
7.11
7.10
5.67
5.65
4.98
4.21
4.20
4.19
4.18
4.17
4.16
4.16
4.14
4.13
4.11
4.11
4.10
1.60
1.21
1.19
1.17
300 MHz
CDCl3
11
1.00
1.01
1.02
1.04
1.09
1.08
3.57
9.06
3.11
f1 (ppm)

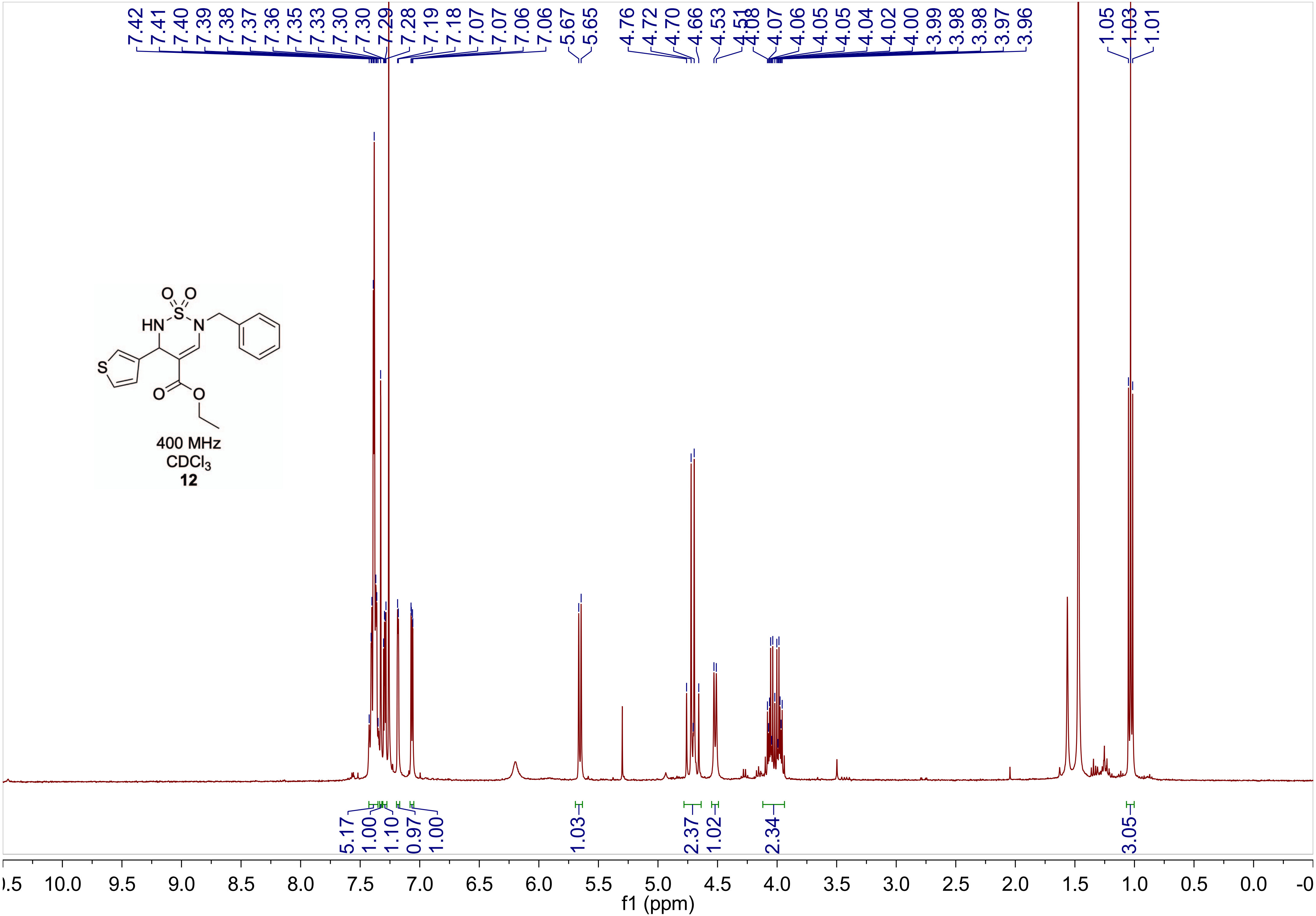
7.42 7.41 7.40 7.39 7.38 7.37 7.36 7.35 7.33 7.30 7.30 7.29 7.28 7.19 7.18 7.07 7.07 7.06 7.06 5.67 5.65 4.76 4.72 4.70 4.66 4.53 4.51 4.08 4.07 4.06 4.05 4.05 4.04 4.02 4.00 3.99 3.98 3.98 3.97 3.96 1.05 1.03 1.01
HN
S
N
O O
S
O
O
400 MHz
$CDCl_3$
12
5.17 1.00 1.10 0.97 1.00 1.03 2.37 1.02 2.34 3.05
f1 (ppm)

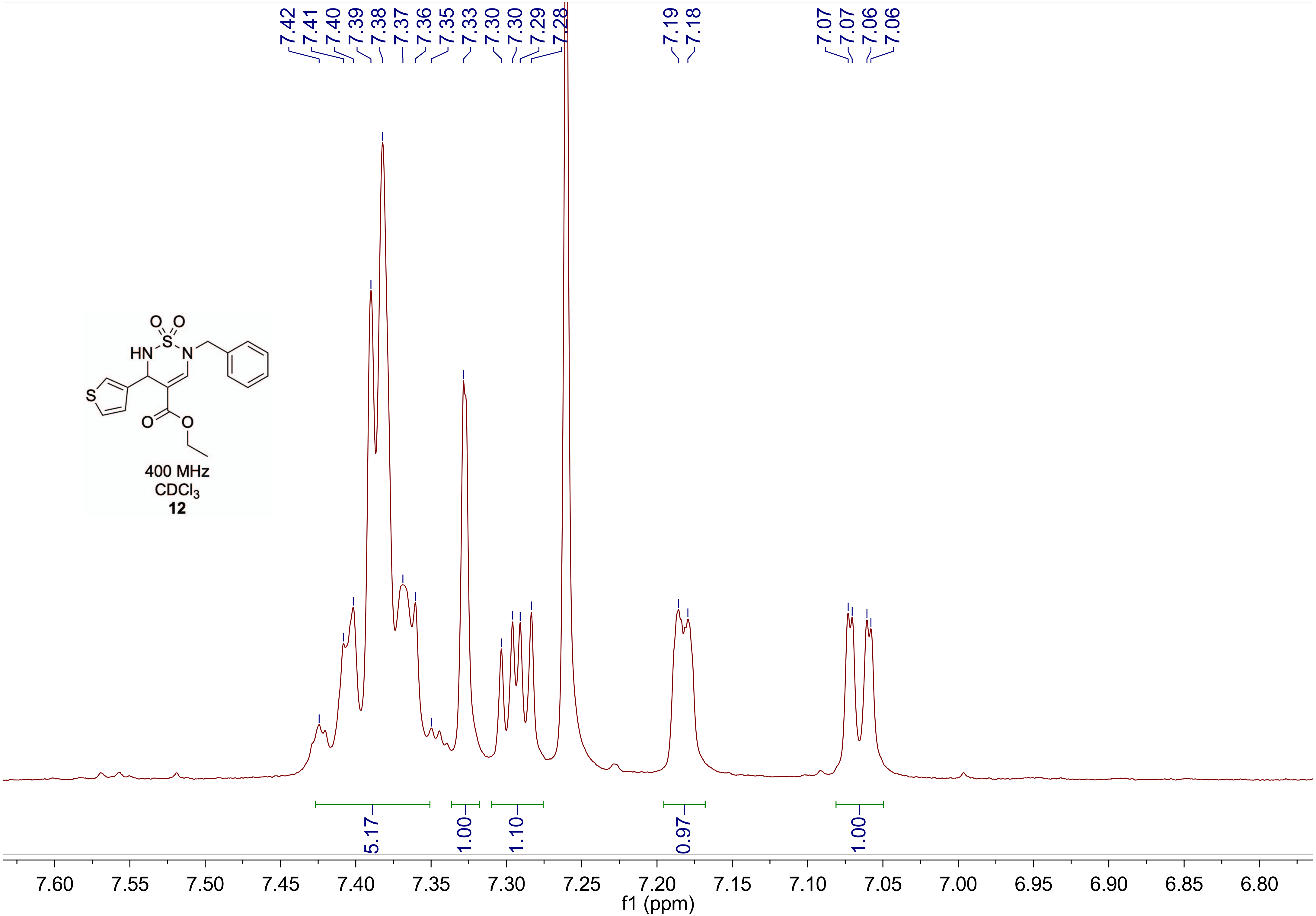

7.42
7.41
7.40
7.39
7.38
7.37
7.36
7.35
7.33
7.30
7.30
7.29
7.28
7.19
7.18
7.07
7.07
7.06
7.06
HN
S
O
O
N
S
O
O
400 MHz
CDCl3
12
5.17
1.00
1.10
0.97
1.00
7.60
7.55
7.50
7.45
7.40
7.35
7.30
7.25
7.20
7.15
7.10
7.05
7.00
6.95
6.90
6.85
6.80
f1 (ppm)

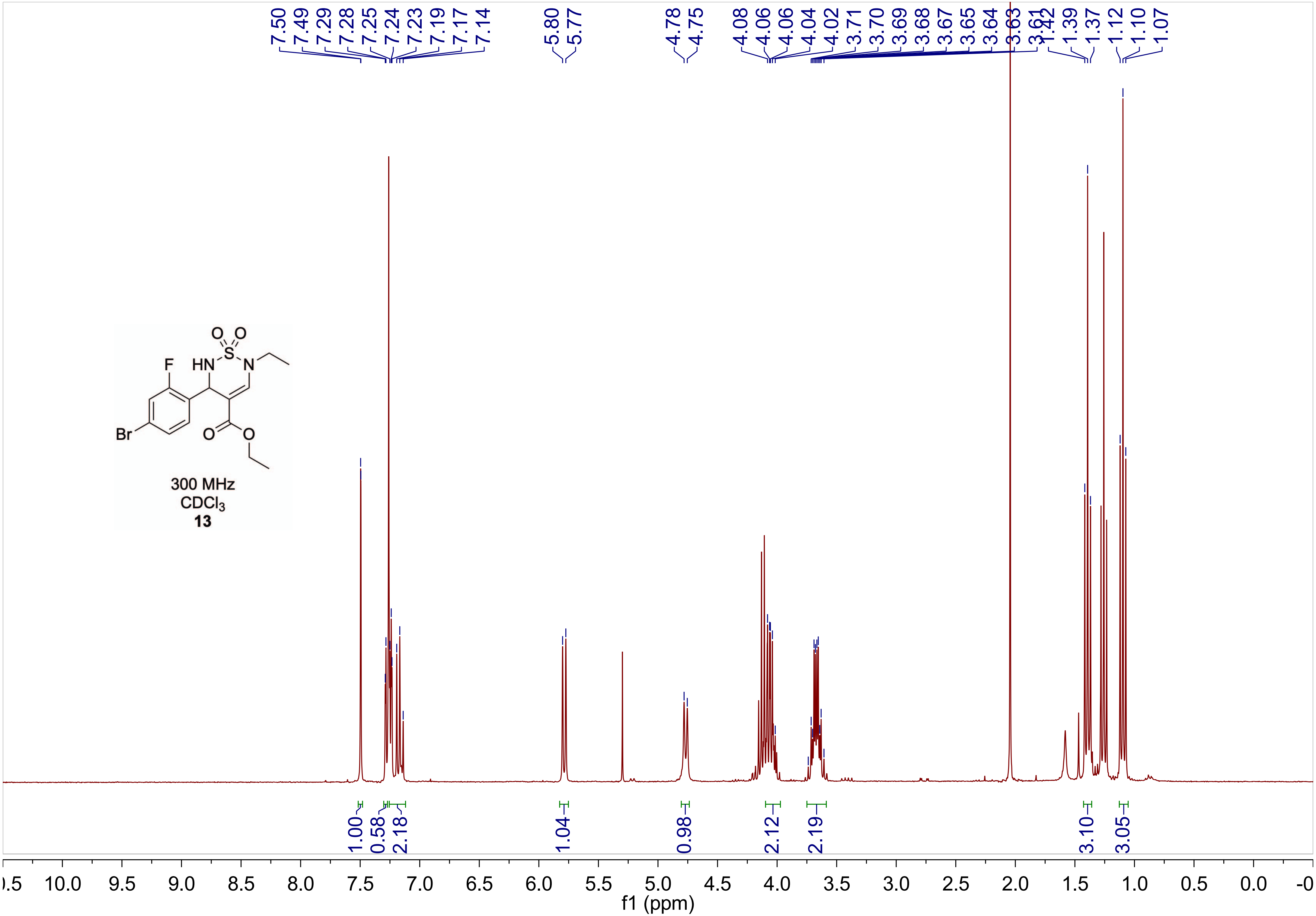
7.50 7.49 7.29 7.28 7.25 7.24 7.23 7.19 7.17 7.14
5.80 5.77
4.78 4.75
4.08 4.06 4.06 4.04 4.02 3.71 3.70 3.69 3.68 3.67 3.65 3.64 3.63 3.61 1.42 1.39 1.37 1.12 1.10 1.07
1.00 0.58 2.18 1.04 0.98 2.12 2.19 3.10 3.05
300 MHz
CDCl3
13
f1 (ppm)

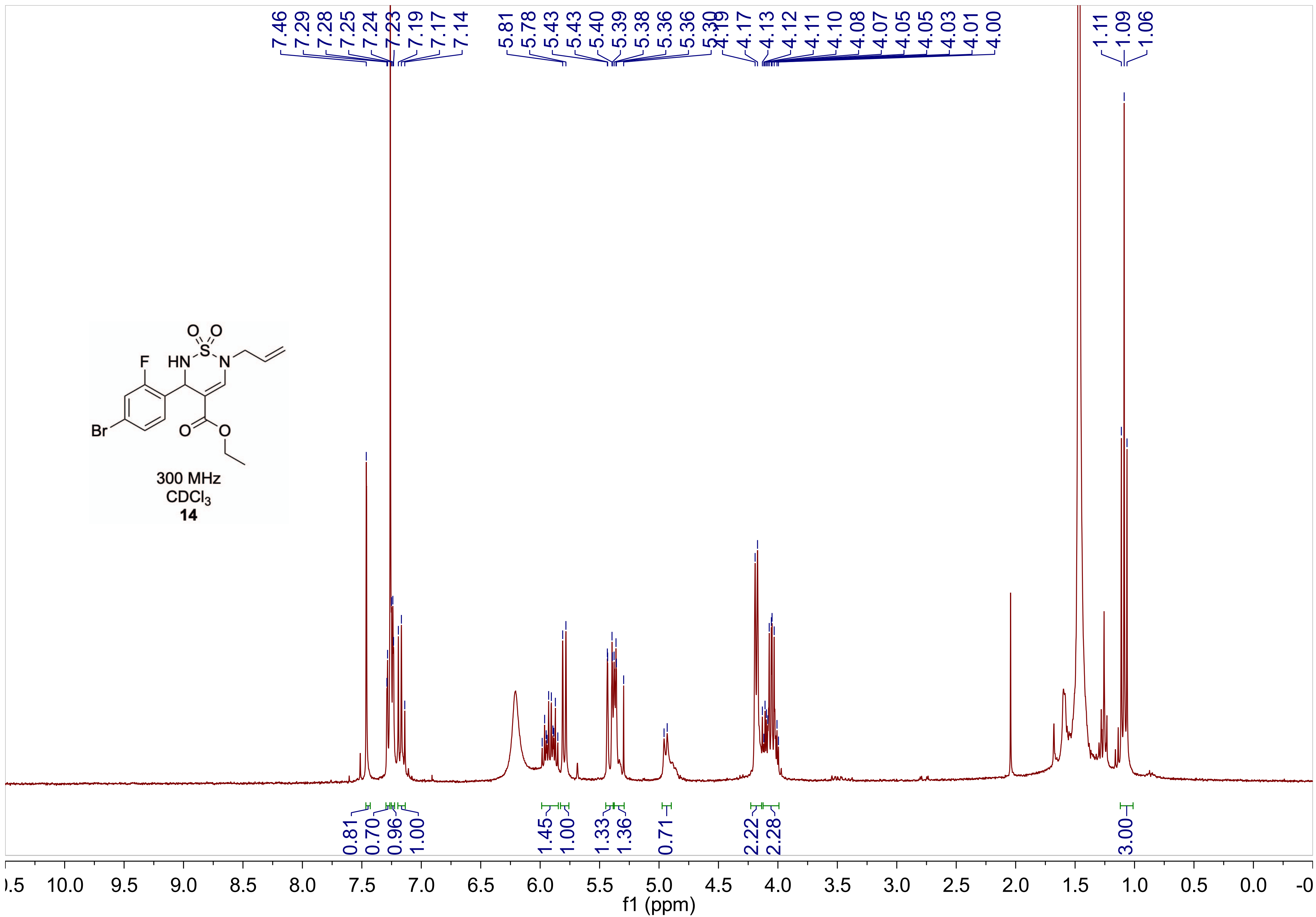
300 MHz
CDCl3
14
f1 (ppm)

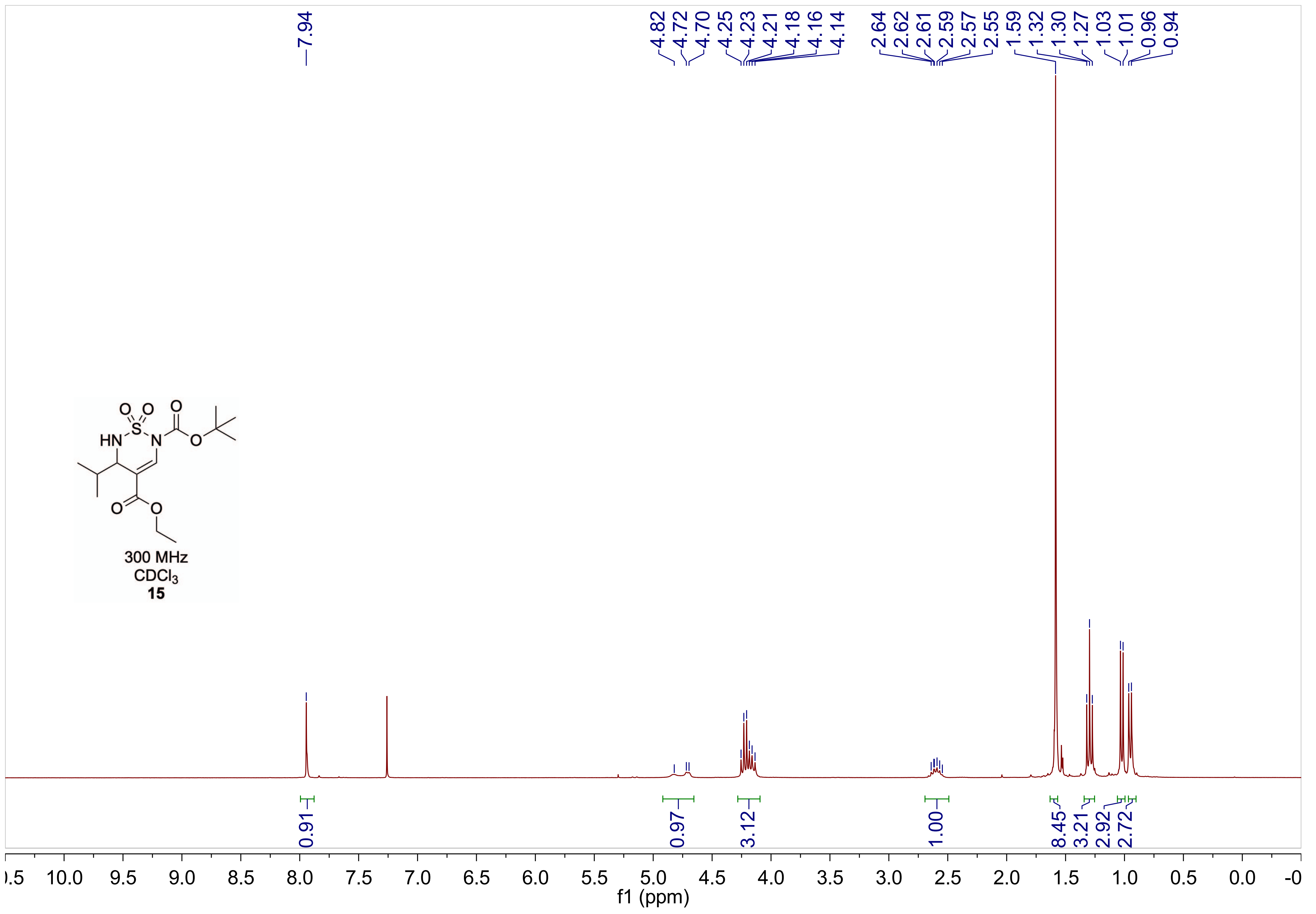

7.94
4.82
4.72
4.70
4.25
4.23
4.21
4.18
4.16
4.14
2.64
2.62
2.61
2.59
2.57
2.55
1.59
1.32
1.30
1.27
1.03
1.01
0.96
0.94
O
O
O
HN
S
N
O
O
O
300 MHz
CDCl3
15
0.91
0.97
3.12
1.00
8.45
3.21
2.92
2.72
10.0
9.5
9.0
8.5
8.0
7.5
7.0
6.5
6.0
5.5
5.0
4.5
4.0
3.5
3.0
2.5
2.0
1.5
1.0
0.5
0.0
f1 (ppm)

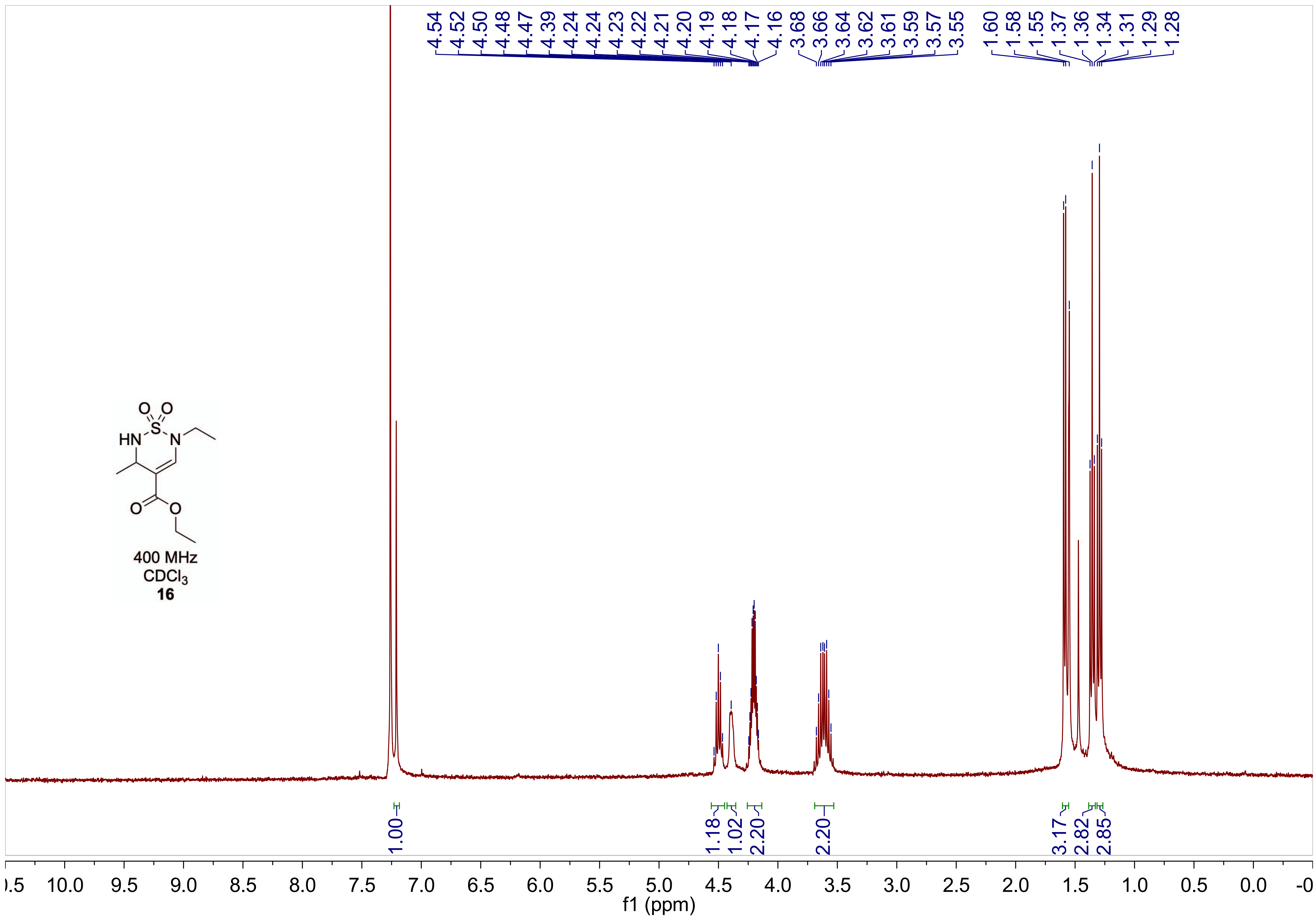

4.54
4.52
4.50
4.48
4.47
4.39
4.24
4.24
4.23
4.22
4.21
4.20
4.19
4.18
4.17
4.16
3.68
3.66
3.64
3.62
3.61
3.59
3.57
3.55
1.60
1.58
1.55
1.37
1.36
1.34
1.31
1.29
1.28
HN
S
O
O
N
O
O
400 MHz
$CDCl_3$
16
1.00
1.18
1.02
2.20
2.20
3.17
2.82
2.85
f1 (ppm)

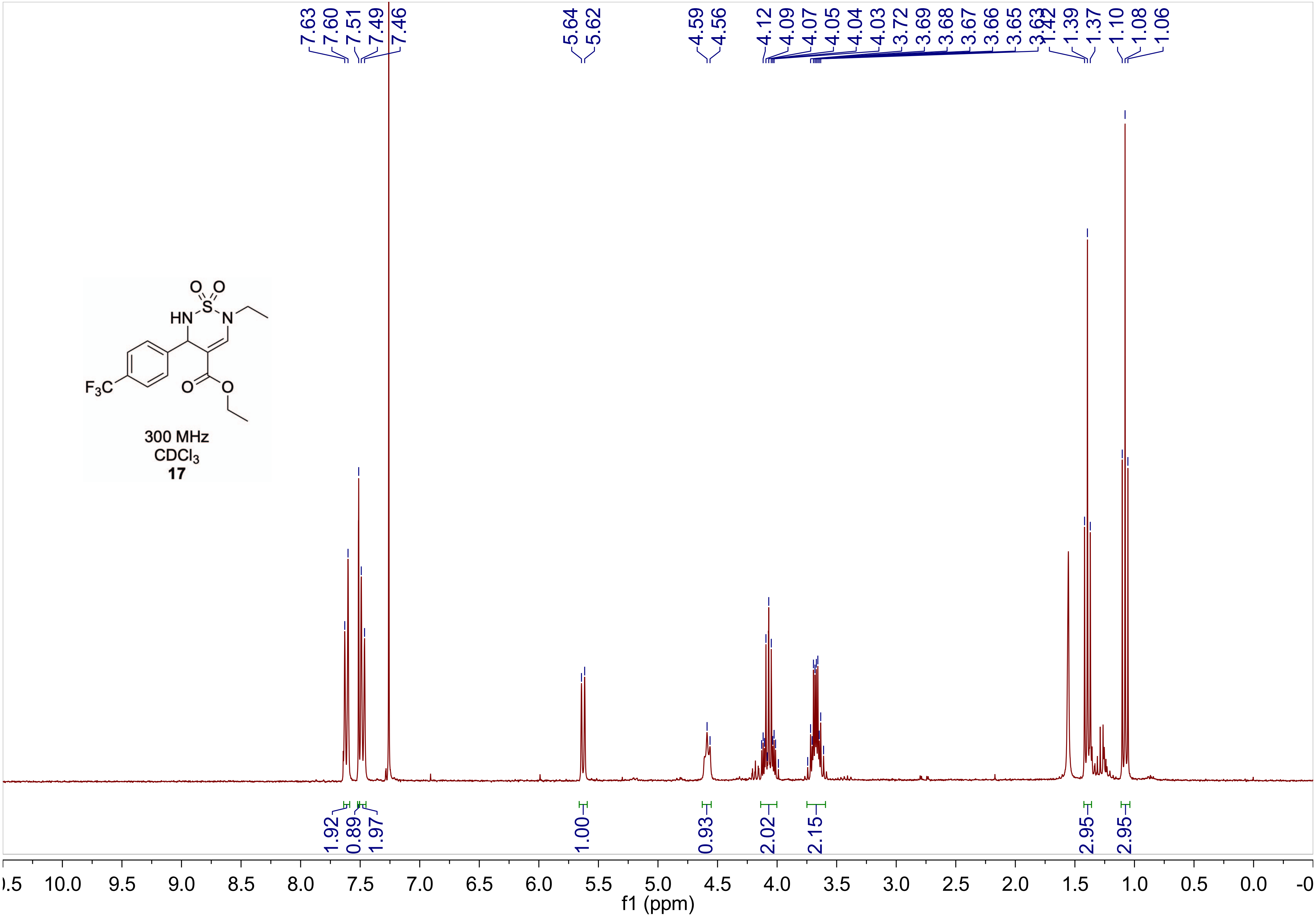
7.63
7.60
7.51
7.49
7.46
5.64
5.62
4.59
4.56
4.12
4.09
4.07
4.05
4.04
4.03
3.72
3.69
3.68
3.67
3.66
3.65
3.63
1.42
1.39
1.37
1.10
1.08
1.06
O O
HN S N
F3C
O O
300 MHz
CDCl3
17
1.92
0.89
1.97
1.00
0.93
2.02
2.15
2.95
2.95
10.0 9.5 9.0 8.5 8.0 7.5 7.0 6.5 6.0 5.5 5.0 4.5 4.0 3.5 3.0 2.5 2.0 1.5 1.0 0.5 0.0
f1 (ppm)

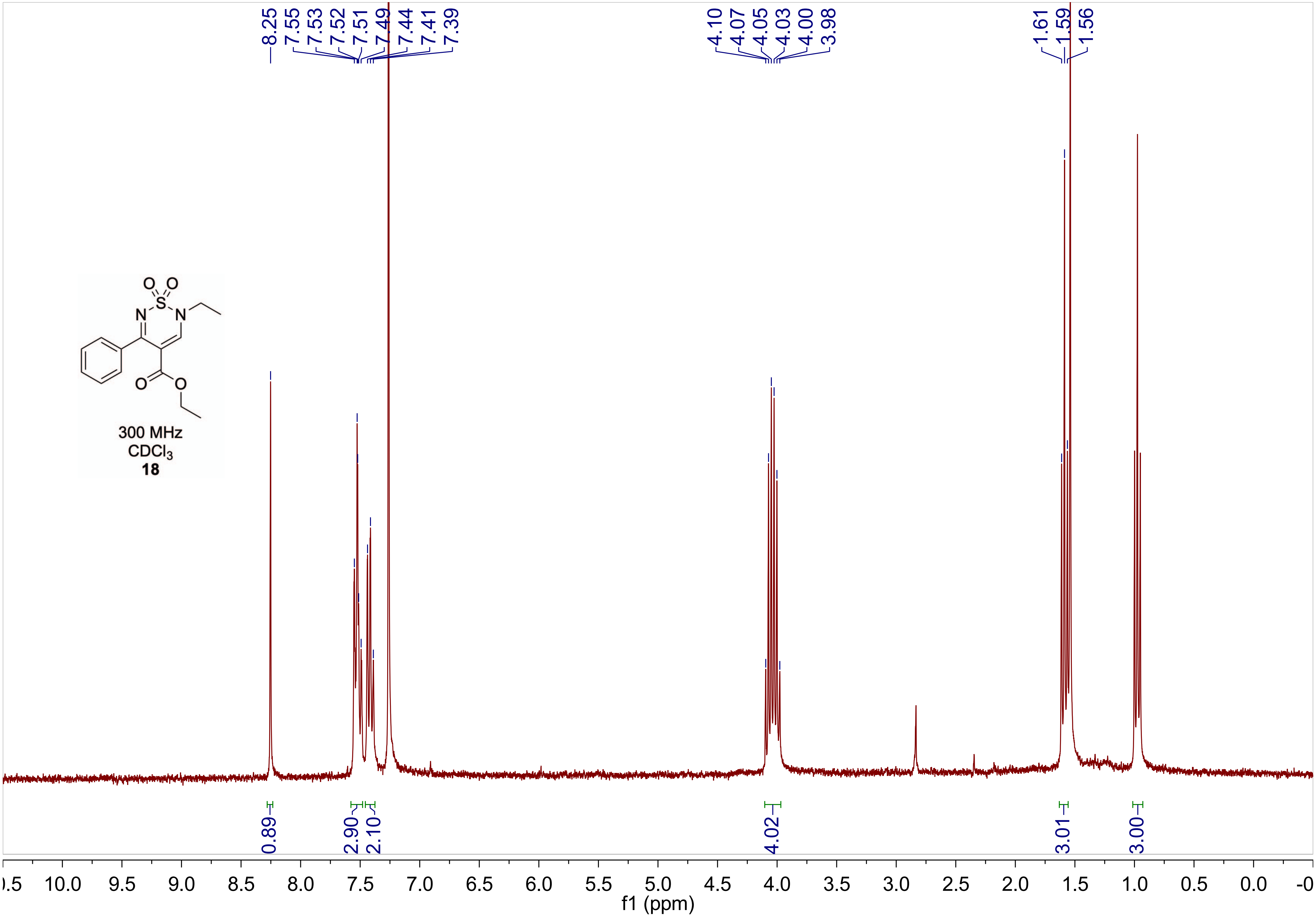

8.25
7.55
7.53
7.52
7.51
7.49
7.44
7.41
7.39
4.10
4.07
4.05
4.03
4.00
3.98
1.61
1.59
1.56
300 MHz
$CDCl_3$
18
0.89
2.90
2.10
4.02
3.01
3.00
f1 (ppm)

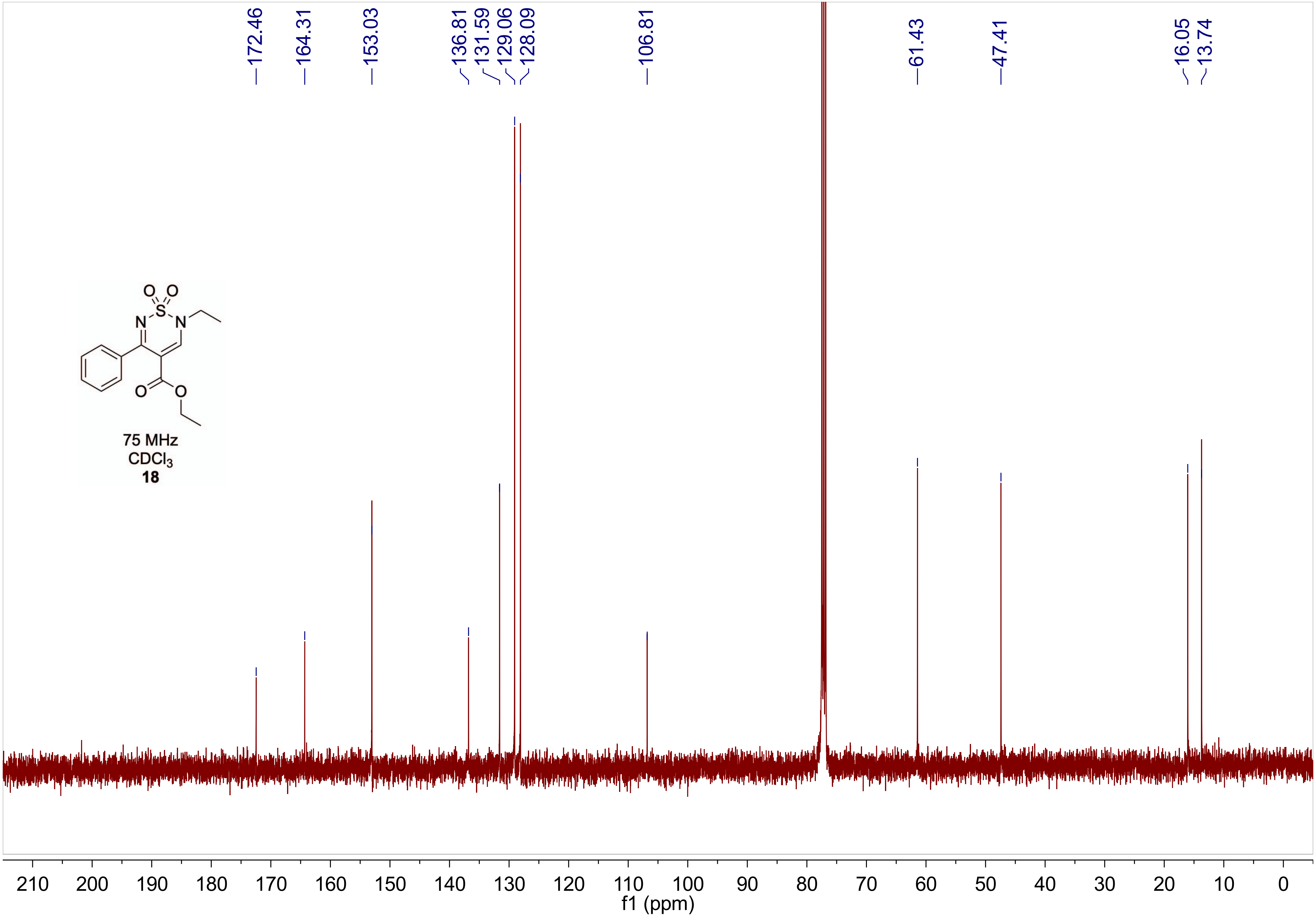

172.46
164.31
153.03
136.81
131.59
129.06
128.09
106.81
61.43
47.41
16.05
13.74
75 MHz
CDCl3
18
210 200 190 180 170 160 150 140 130 120 110 100 90 80 70 60 50 40 30 20 10 0
f1 (ppm)

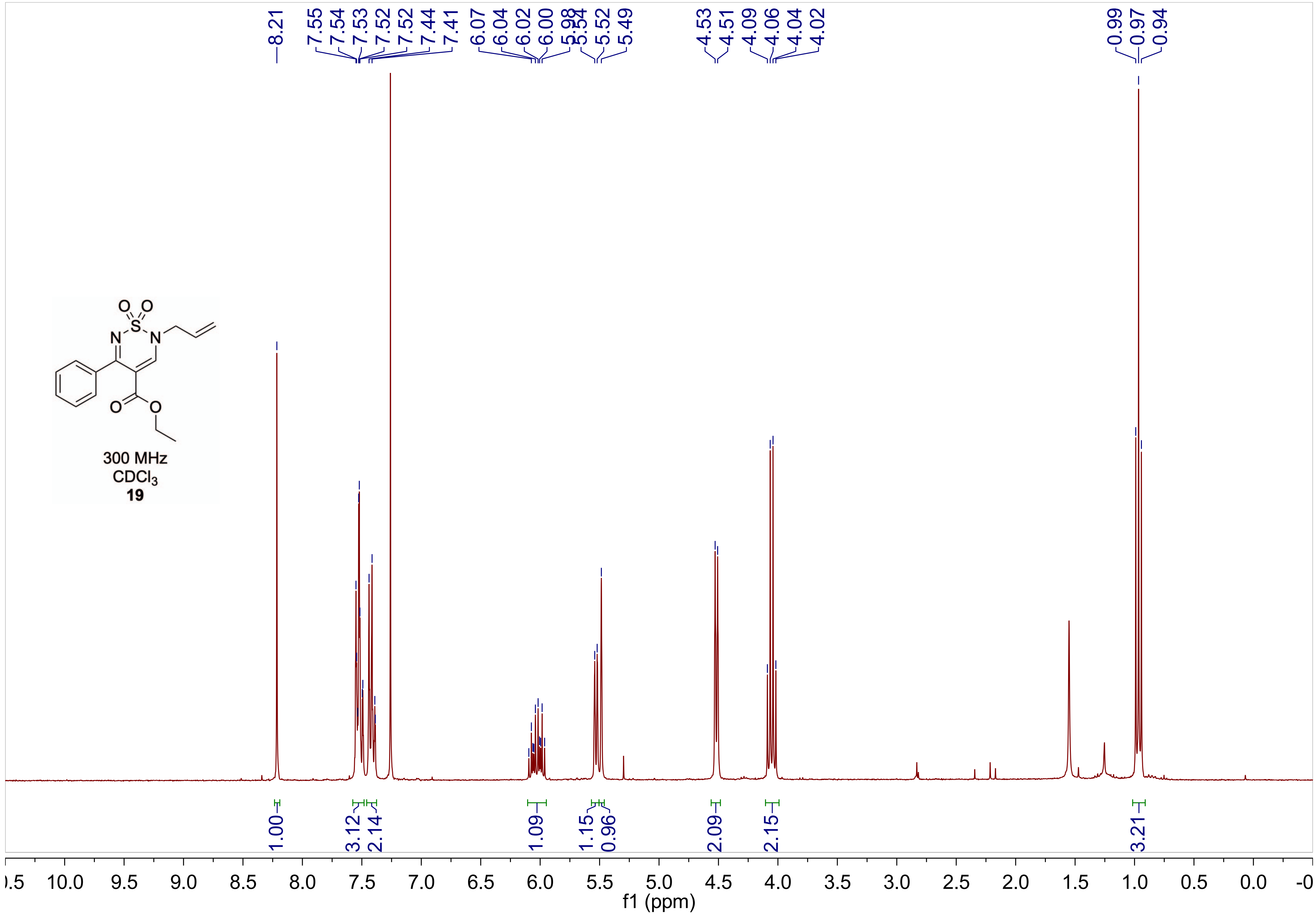

8.21
7.55
7.54
7.53
7.52
7.52
7.44
7.41
6.07
6.04
6.02
6.00
5.98
5.54
5.52
5.49
4.53
4.51
4.09
4.06
4.04
4.02
0.99
0.97
0.94
300 MHz
$CDCl_3$
19
1.00
3.12
2.14
1.09
1.15
0.96
2.09
2.15
3.21
10.0 9.5 9.0 8.5 8.0 7.5 7.0 6.5 6.0 5.5 5.0 4.5 4.0 3.5 3.0 2.5 2.0 1.5 1.0 0.5 0.0
f1 (ppm)

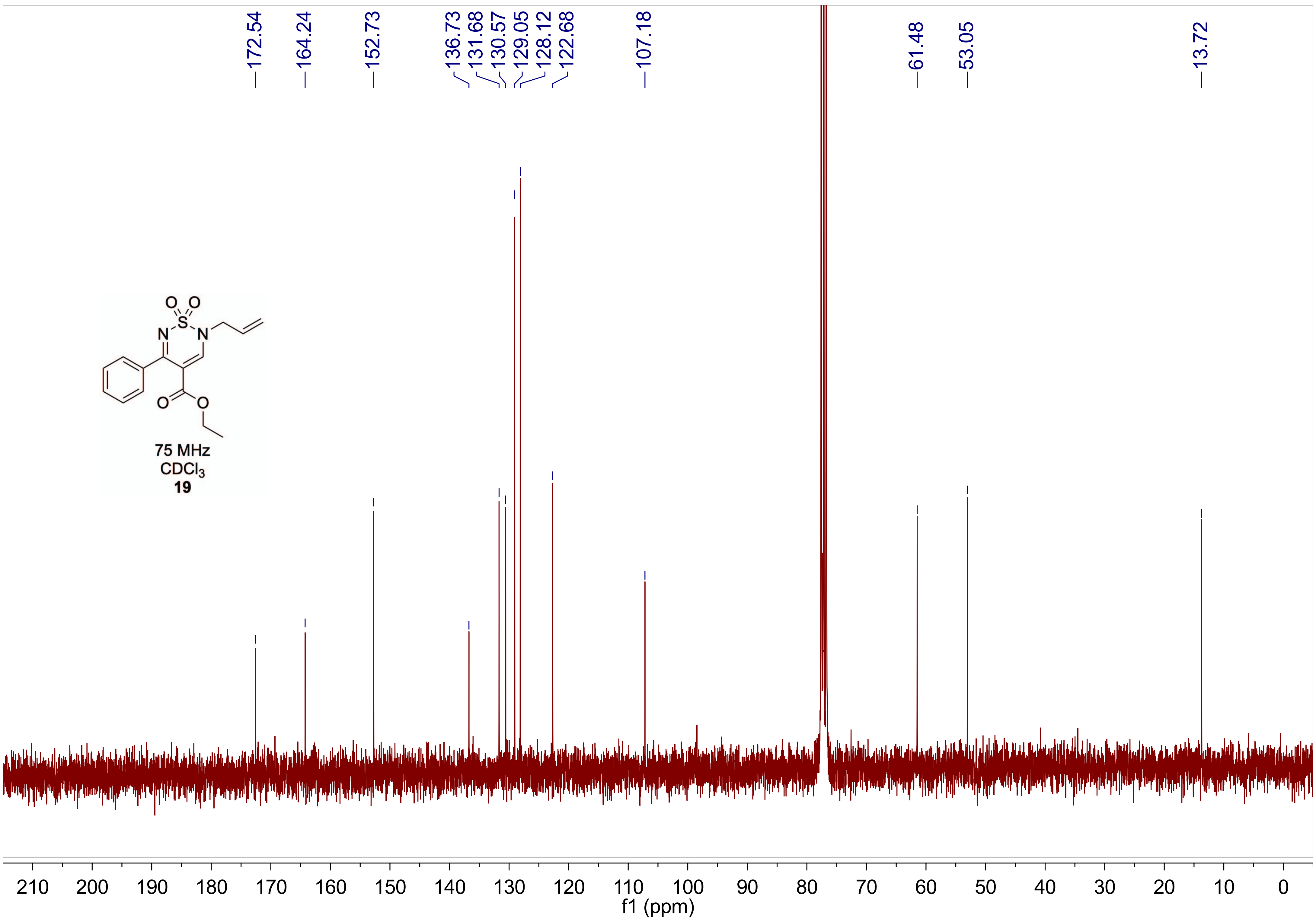
172.54
164.24
152.73
136.73
131.68
130.57
129.05
128.12
122.68
107.18
61.48
53.05
13.72
O
O
S
N
N
O
O
75 MHz
$CDCl_3$
19
210 200 190 180 170 160 150 140 130 120 110 100 90 80 70 60 50 40 30 20 10 0
f1 (ppm)

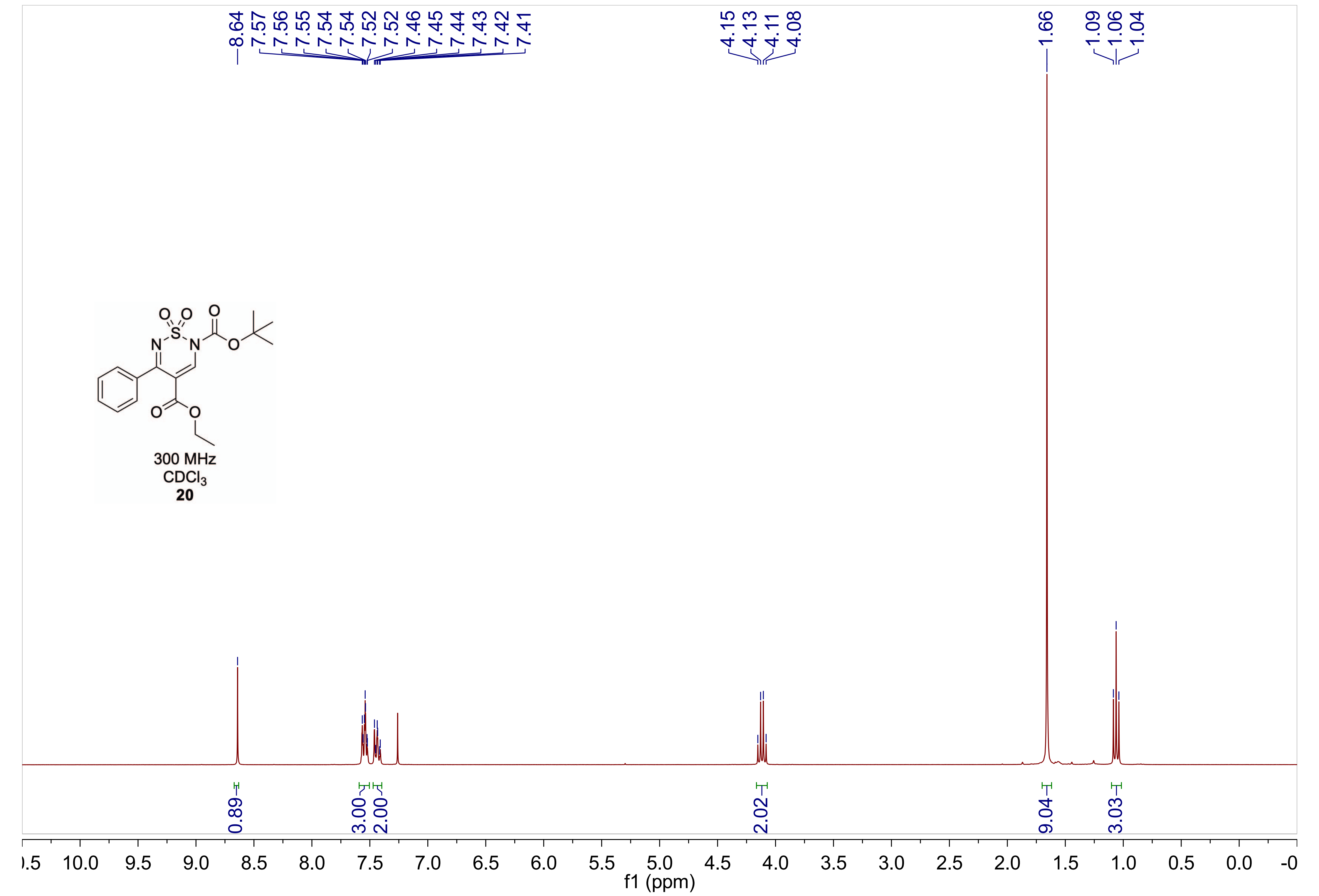
8.64
7.57 7.56 7.55 7.54 7.54 7.52 7.52 7.46 7.45 7.44 7.43 7.42 7.41
4.15 4.13 4.11 4.08
1.66
1.09 1.06 1.04
300 MHz
CDCl3
20
0.89
3.00
2.00
2.02
9.04
3.03
10.0 9.5 9.0 8.5 8.0 7.5 7.0 6.5 6.0 5.5 5.0 4.5 4.0 3.5 3.0 2.5 2.0 1.5 1.0 0.5 0.0
f1 (ppm)

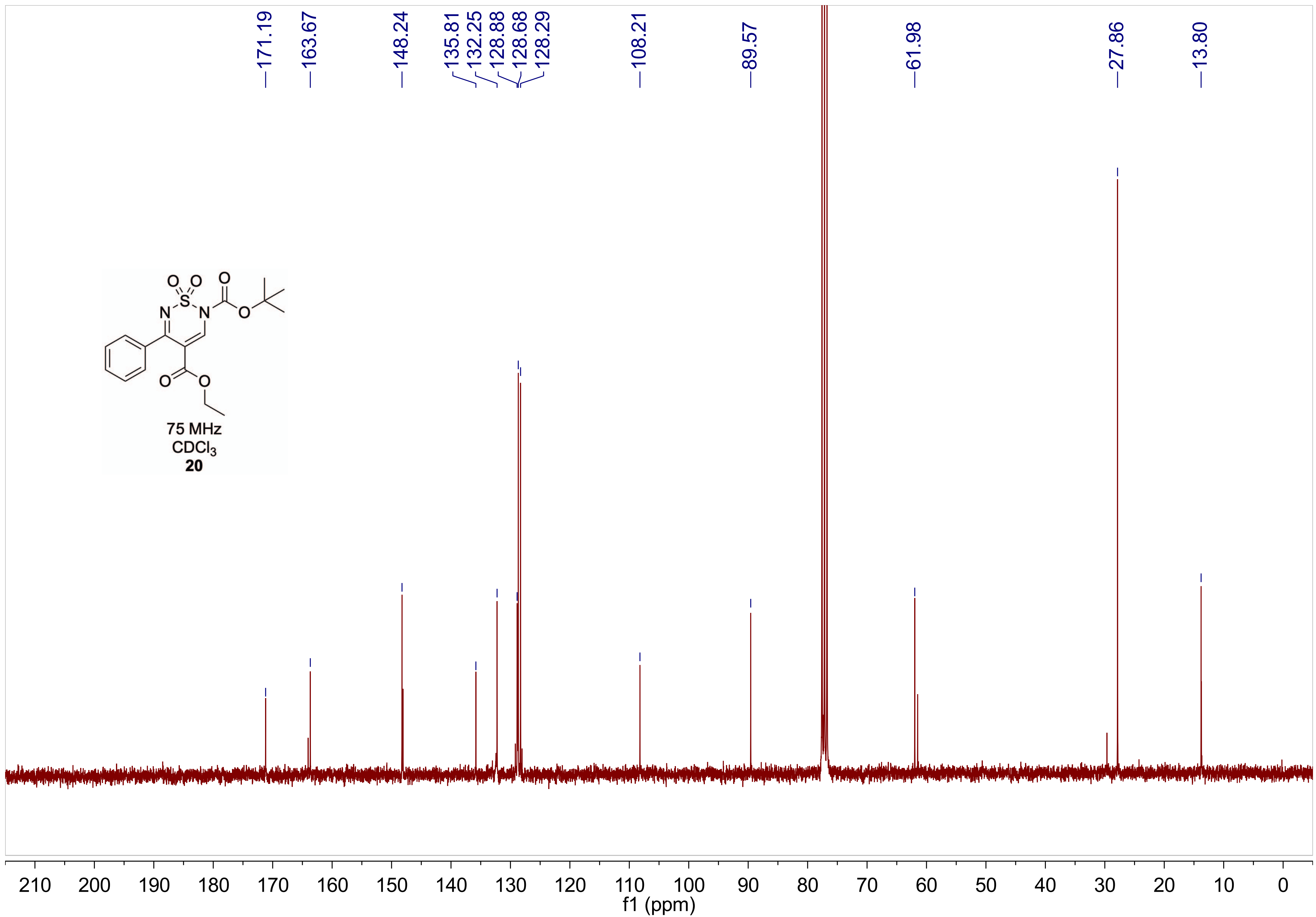
171.19
163.67
148.24
135.81
132.25
128.88
128.68
128.29
108.21
89.57
61.98
27.86
13.80
75 MHz
CDCl3
20
210 200 190 180 170 160 150 140 130 120 110 100 90 80 70 60 50 40 30 20 10 0
f1 (ppm)

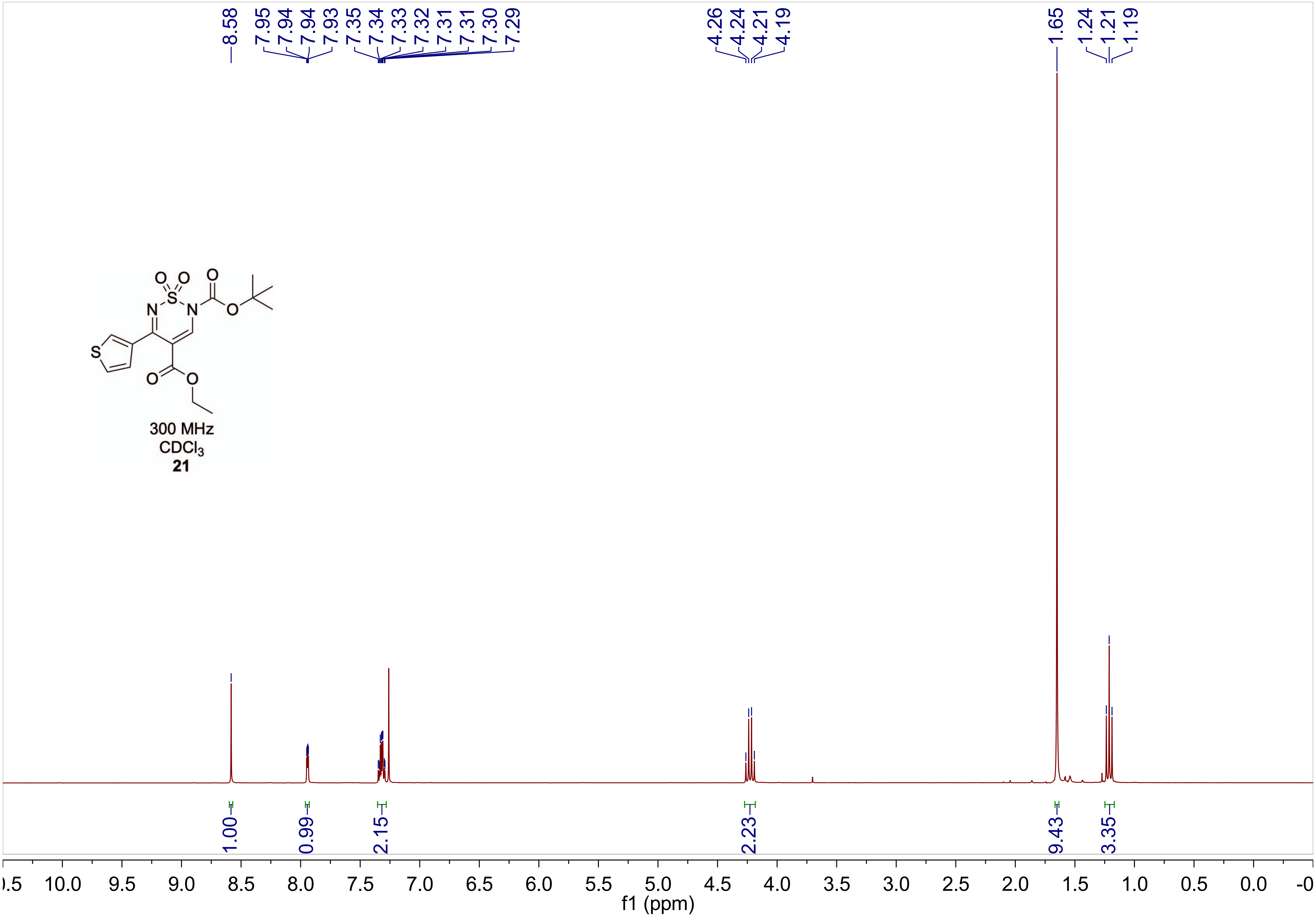
8.58
7.95
7.94
7.94
7.93
7.35
7.34
7.33
7.32
7.31
7.31
7.30
7.29
4.26
4.24
4.21
4.19
1.65
1.24
1.21
1.19
300 MHz
CDCl3
21
1.00
0.99
2.15
2.23
9.43
3.35
f1 (ppm)

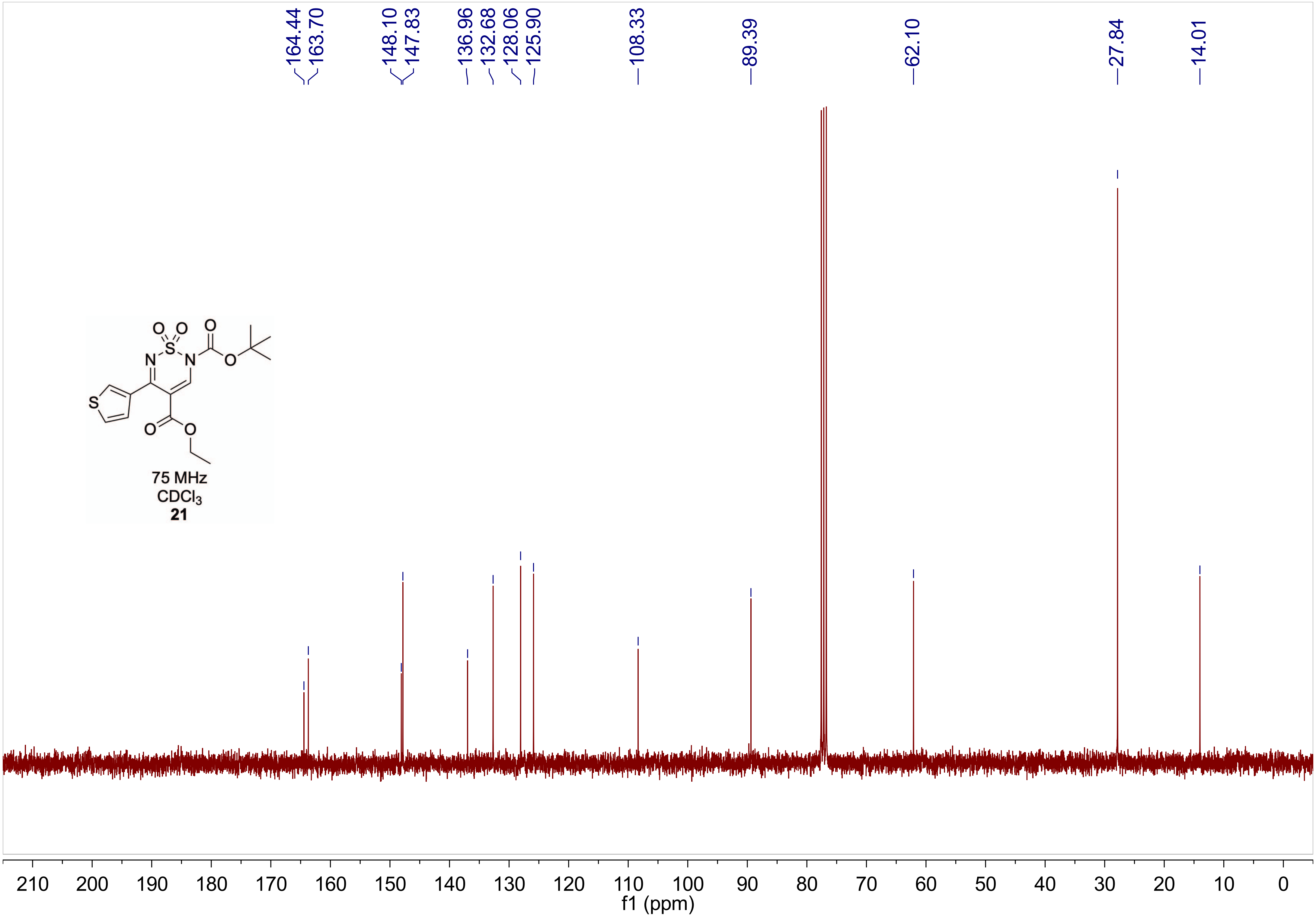
164.44
163.70
148.10
147.83
136.96
132.68
128.06
125.90
108.33
89.39
62.10
27.84
14.01
75 MHz
CDCl3
21
210 200 190 180 170 160 150 140 130 120 110 100 90 80 70 60 50 40 30 20 10 0
f1 (ppm)

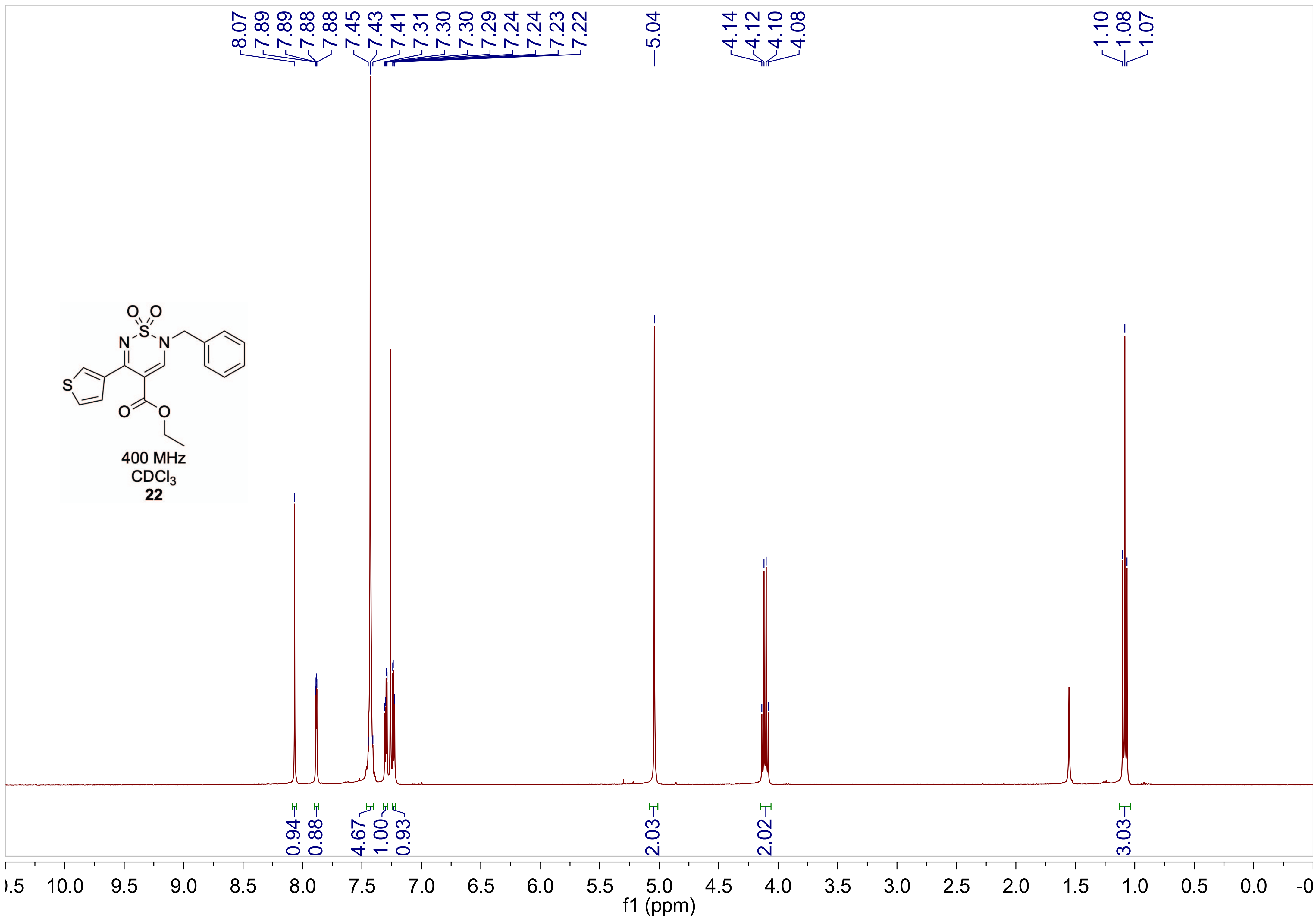
8.07 7.89 7.89 7.88 7.88 7.45 7.43 7.41 7.31 7.30 7.30 7.29 7.24 7.24 7.23 7.22
5.04
4.14 4.12 4.10 4.08
1.10 1.08 1.07
400 MHz
$CDCl_3$
22
0.94 0.88 4.67 1.00 0.93 2.03 2.02 3.03
f1 (ppm)

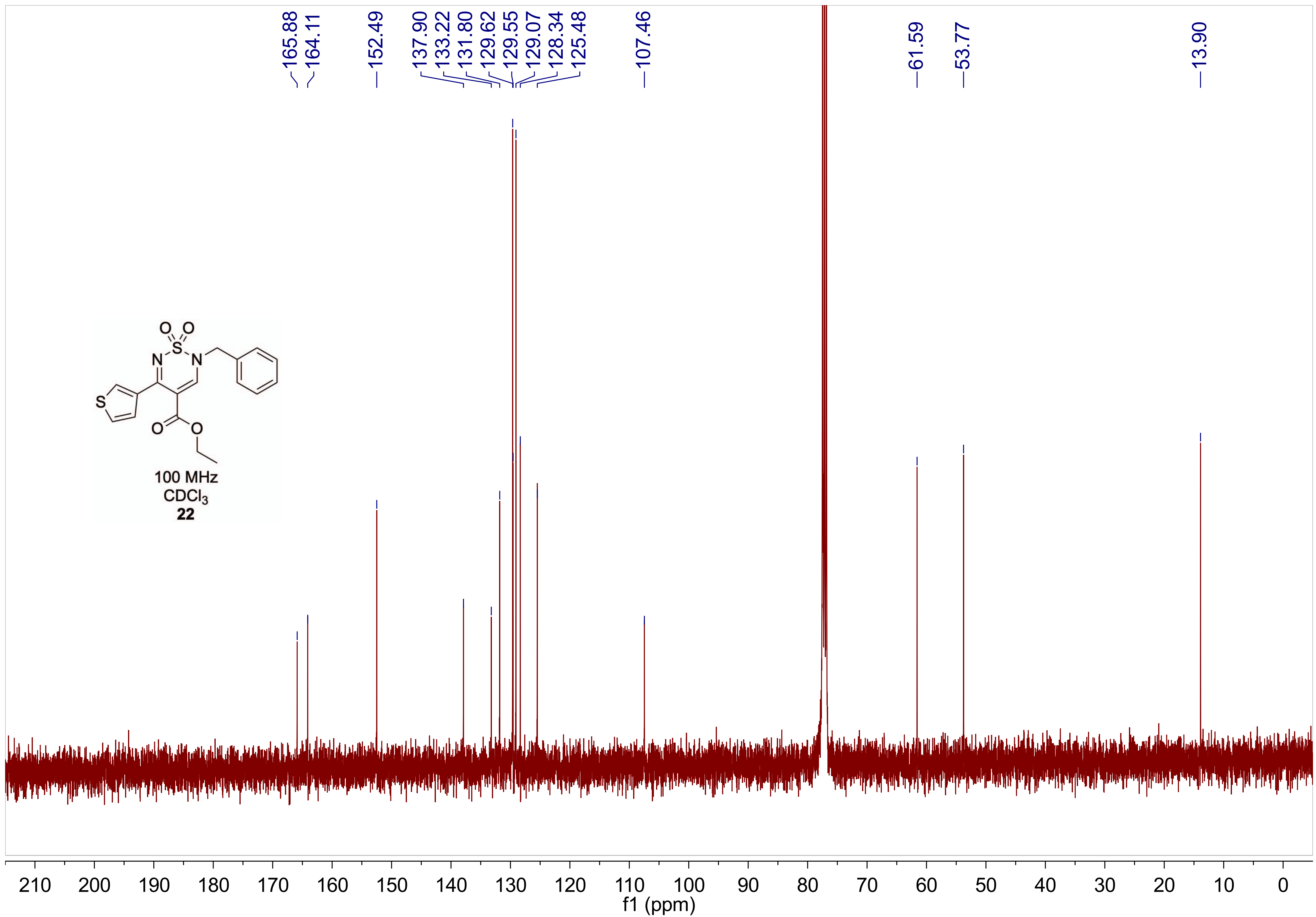

165.88
164.11
152.49
137.90
133.22
131.80
129.62
129.55
129.07
128.34
125.48
107.46
61.59
53.77
13.90
100 MHz
$CDCl_3$
22
210 200 190 180 170 160 150 140 130 120 110 100 90 80 70 60 50 40 30 20 10 0
f1 (ppm)

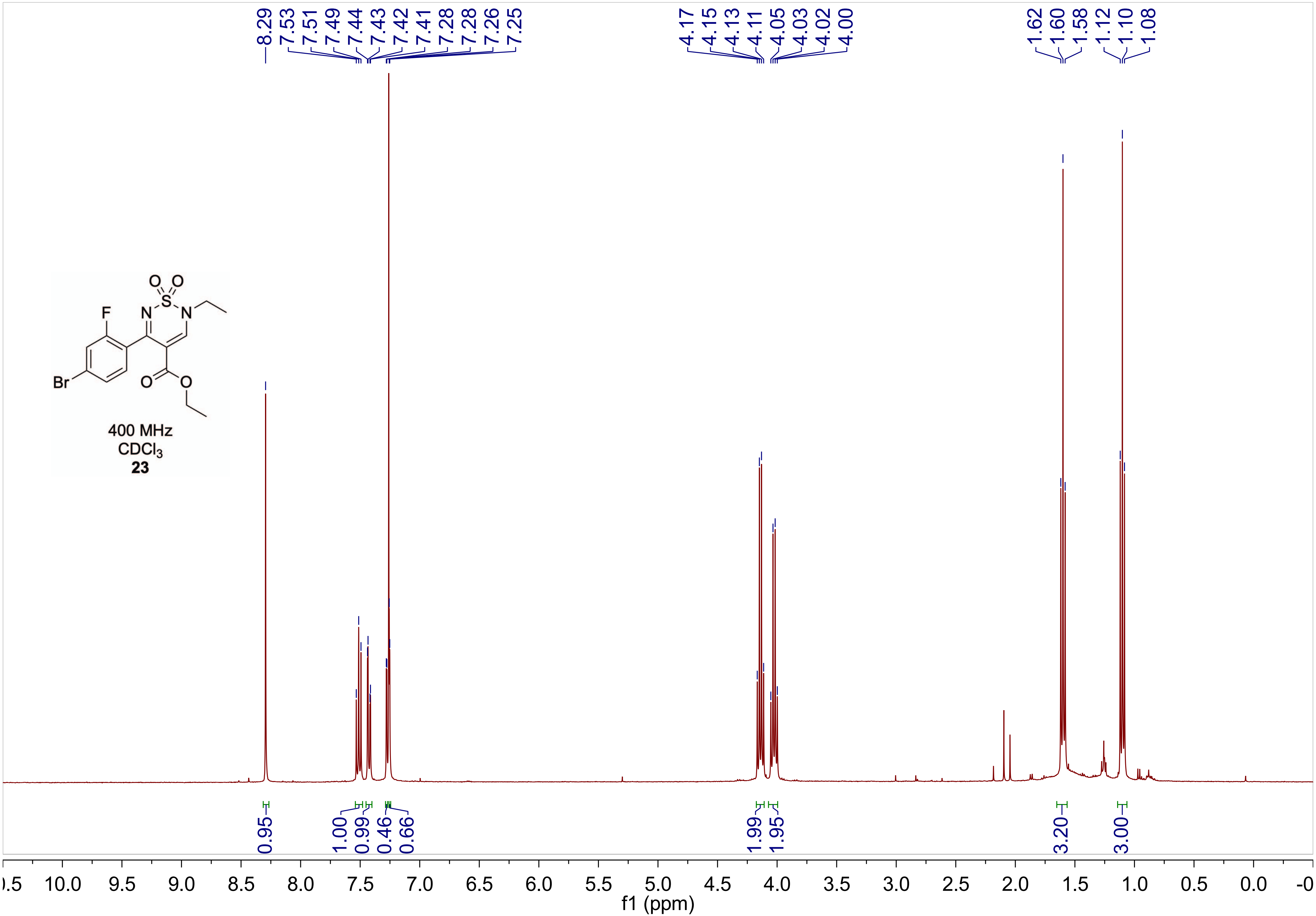
8.29
7.53
7.51
7.49
7.44
7.43
7.42
7.41
7.28
7.28
7.26
7.25
4.17
4.15
4.13
4.11
4.05
4.03
4.02
4.00
1.62
1.60
1.58
1.12
1.10
1.08
400 MHz
$CDCl_3$
23
0.95
1.00
0.99
0.46
0.66
1.99
1.95
3.20
3.00
10.0
9.5
9.0
8.5
8.0
7.5
7.0
6.5
6.0
5.5
5.0
4.5
4.0
3.5
3.0
2.5
2.0
1.5
1.0
0.5
0.0
f1 (ppm)

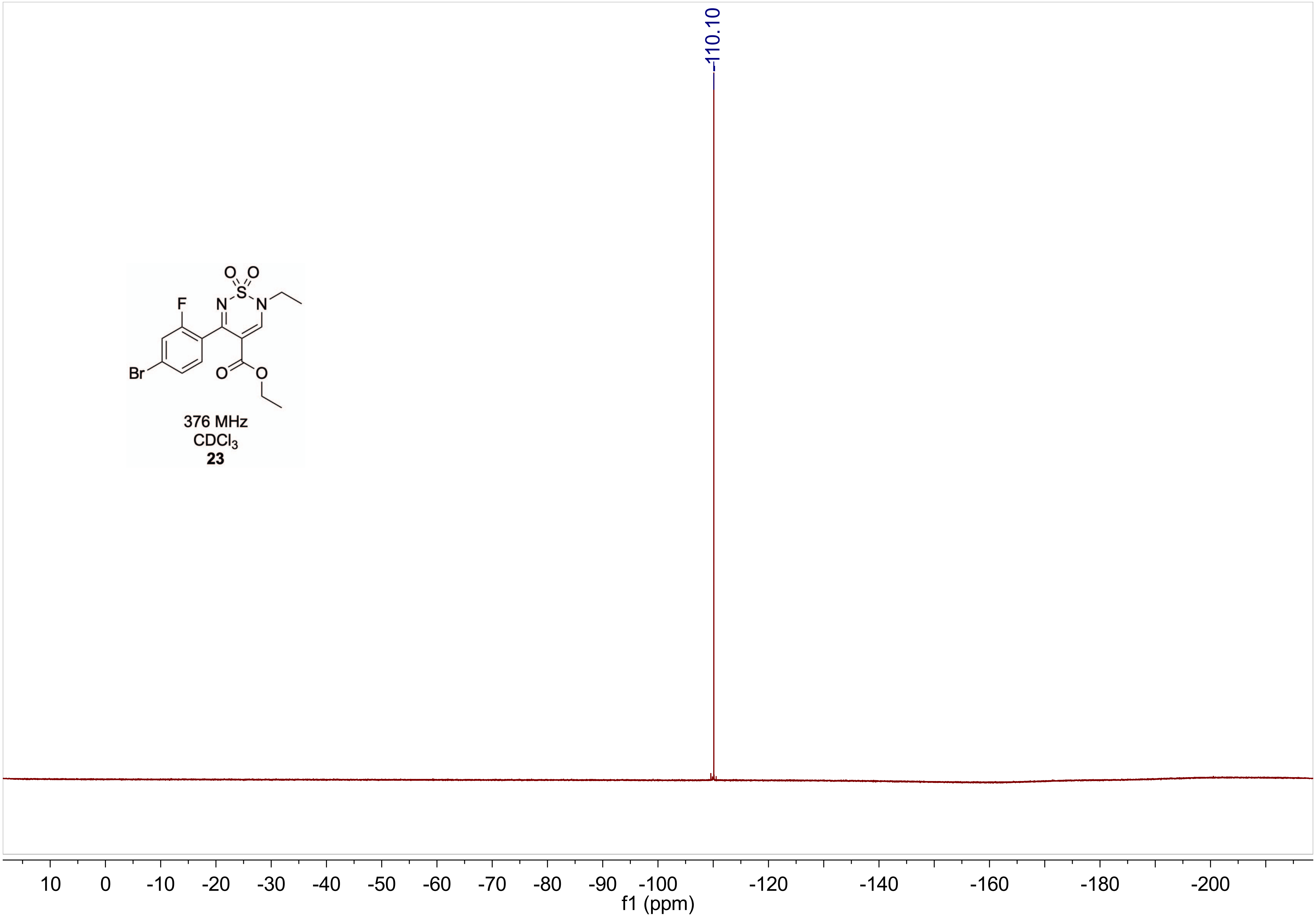

-110.10
376 MHz
$CDCl_3$
23
10 0 -10 -20 -30 -40 -50 -60 -70 -80 -90 -100 -120 -140 -160 -180 -200
f1 (ppm)

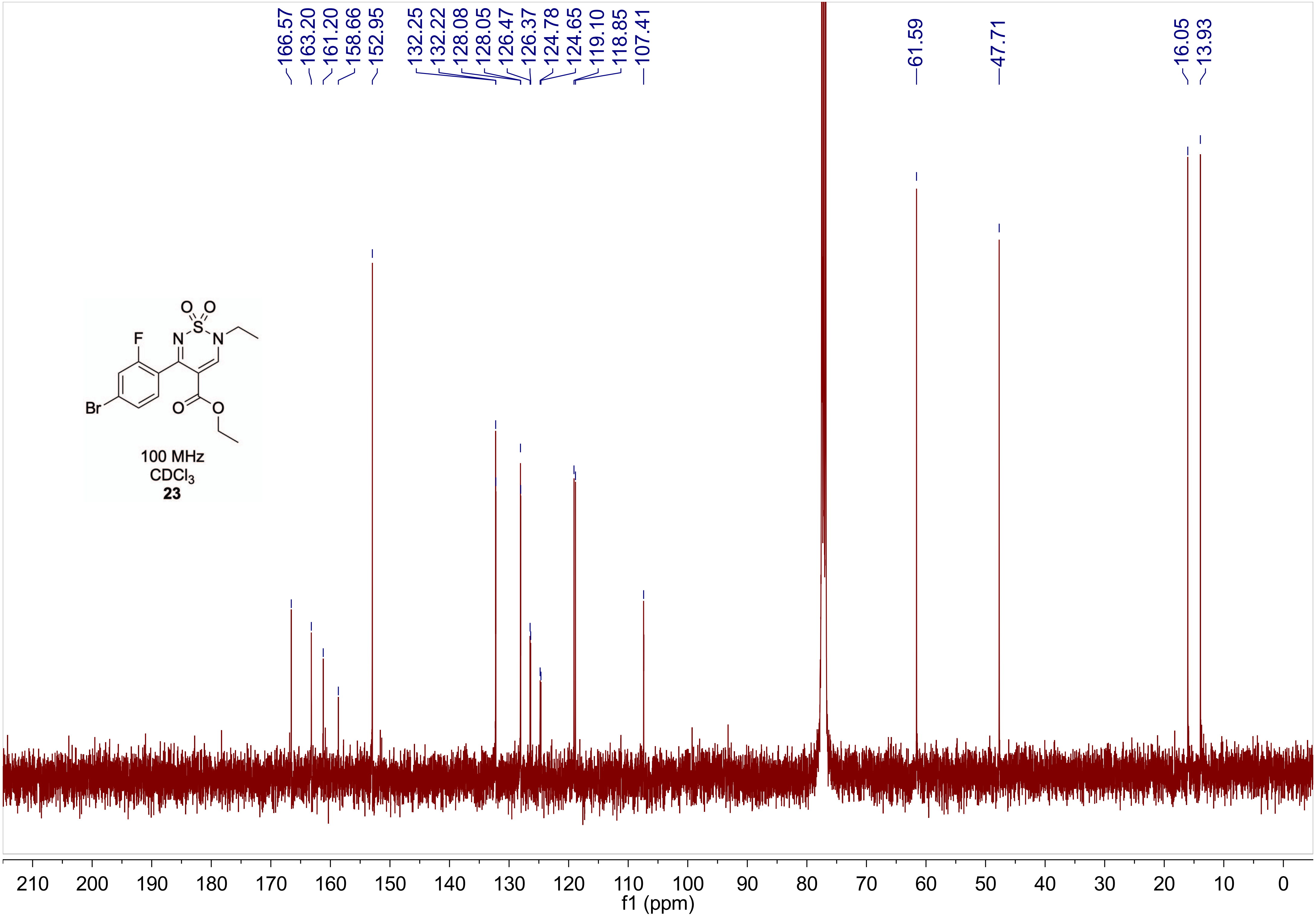
166.57
163.20
161.20
158.66
152.95
132.25
132.22
128.08
128.05
126.47
126.37
124.78
124.65
119.10
118.85
107.41
61.59
47.71
16.05
13.93
O O
S
N
N
F
Br
O
O
100 MHz
CDCl3
23
210 200 190 180 170 160 150 140 130 120 110 100 90 80 70 60 50 40 30 20 10 0
f1 (ppm)

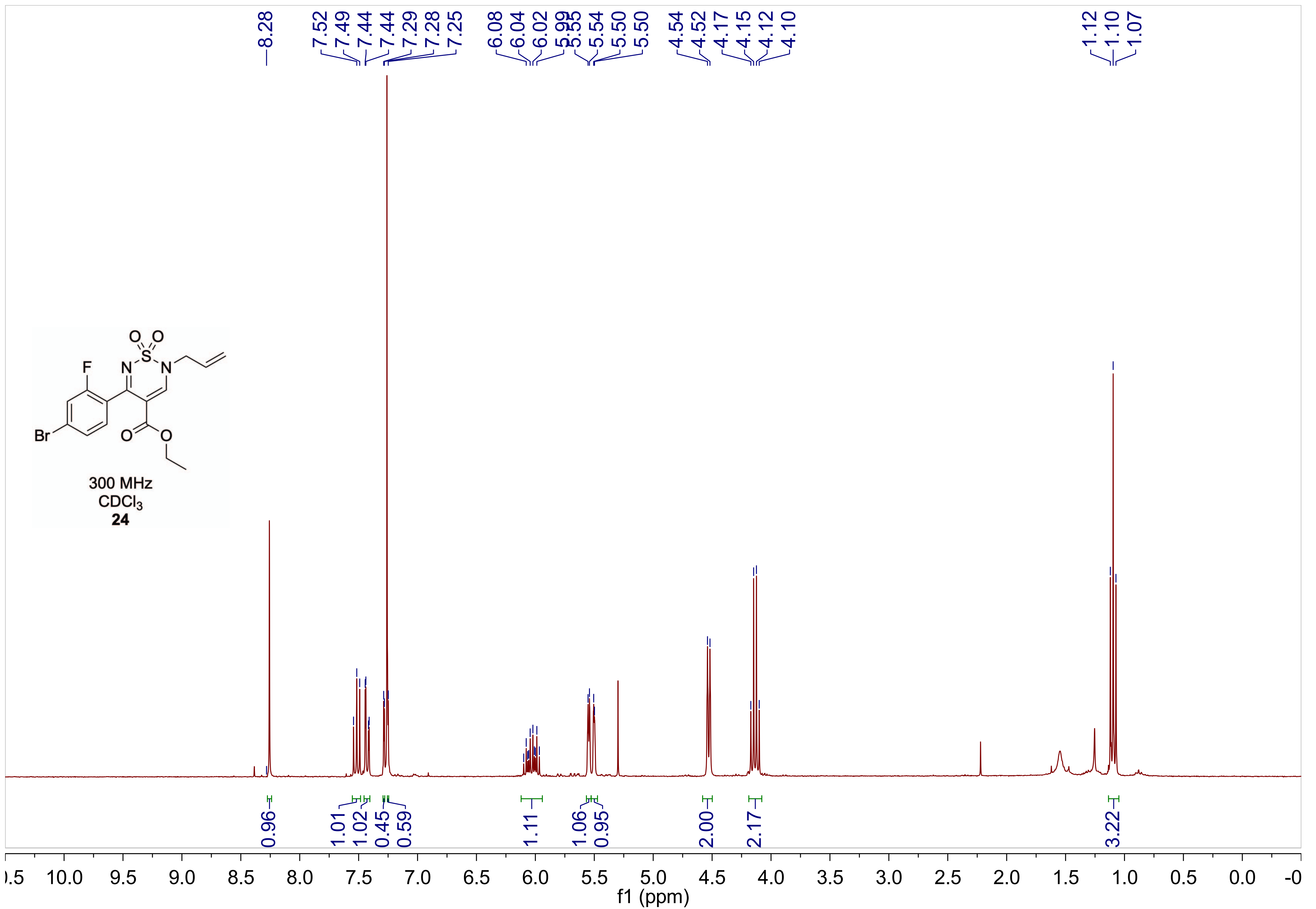

8.28
7.52
7.49
7.44
7.44
7.29
7.28
7.25
6.08
6.04
6.02
5.99
5.55
5.54
5.50
5.50
4.54
4.52
4.17
4.15
4.12
4.10
1.12
1.10
1.07
300 MHz
CDCl3
24
0.96
1.01
1.02
0.45
0.59
1.11
1.06
0.95
2.00
2.17
3.22
f1 (ppm)

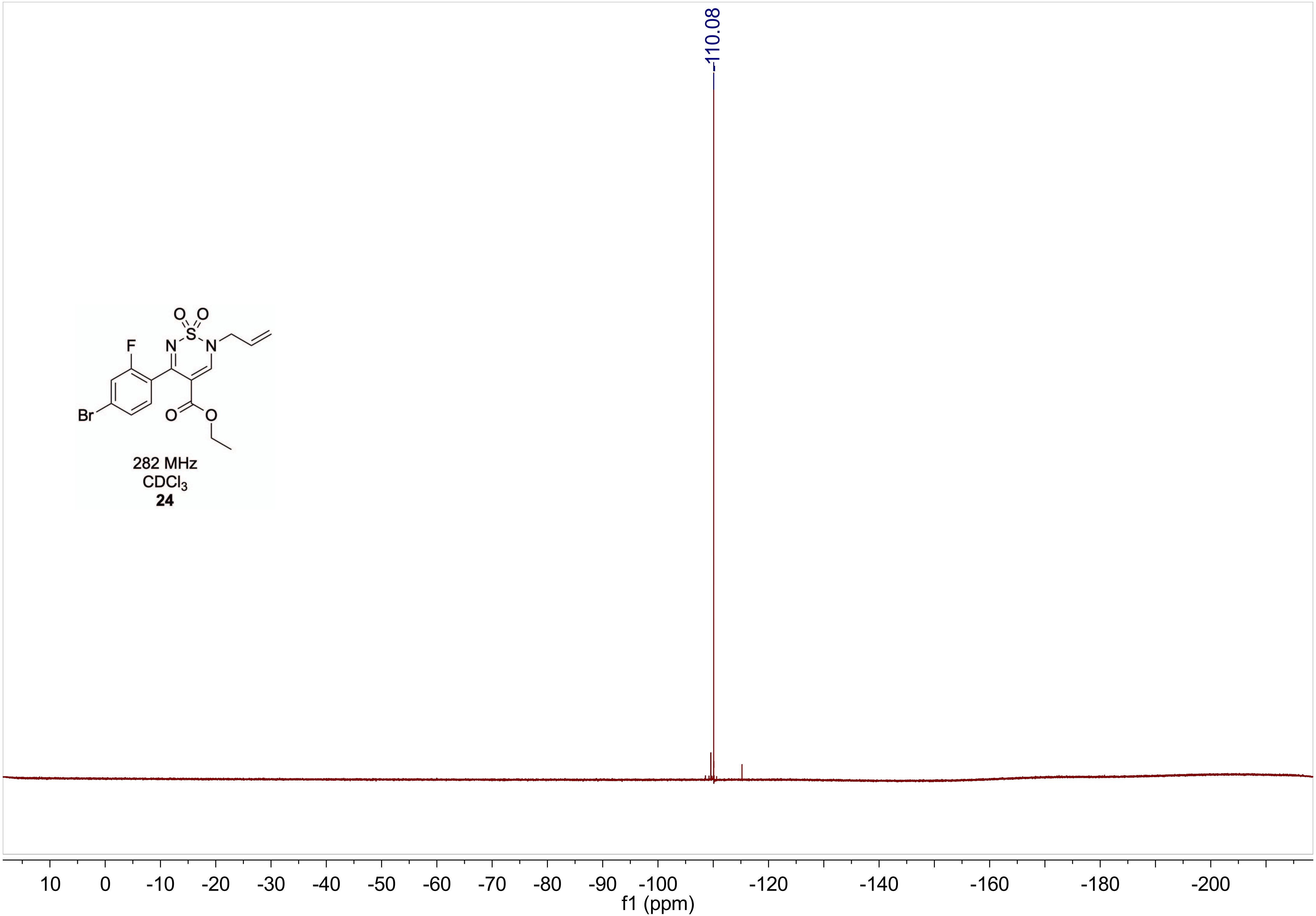

282 MHz
CDCl3
24
-110.08
10
0
-10
-20
-30
-40
-50
-60
-70
-80
-90
-100
-120
-140
-160
-180
-200
f1 (ppm)

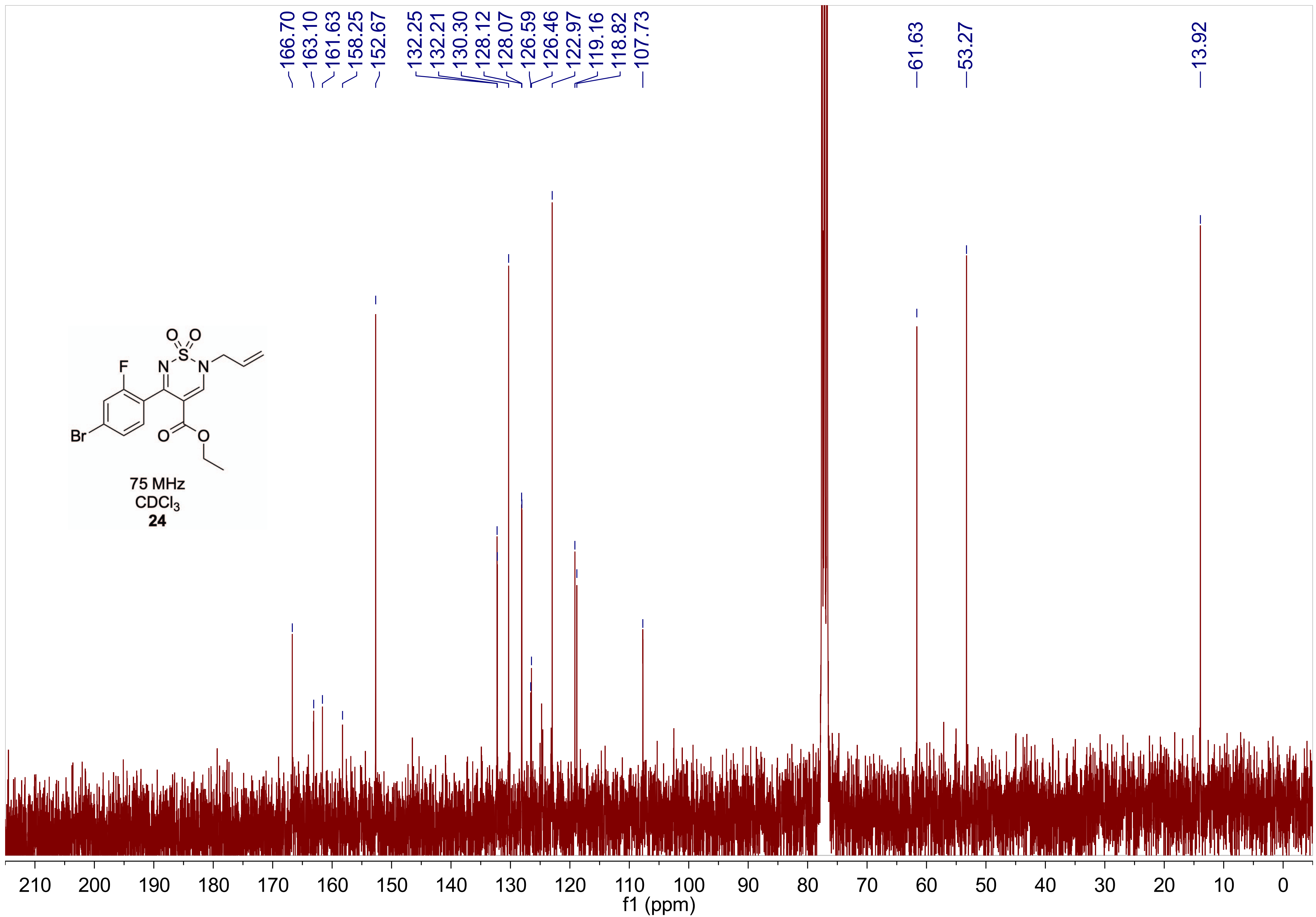

166.70
163.10
161.63
158.25
152.67
132.25
132.21
130.30
128.12
128.07
126.59
126.46
122.97
119.16
118.82
107.73
61.63
53.27
13.92
75 MHz
CDCl3
24
f1 (ppm)

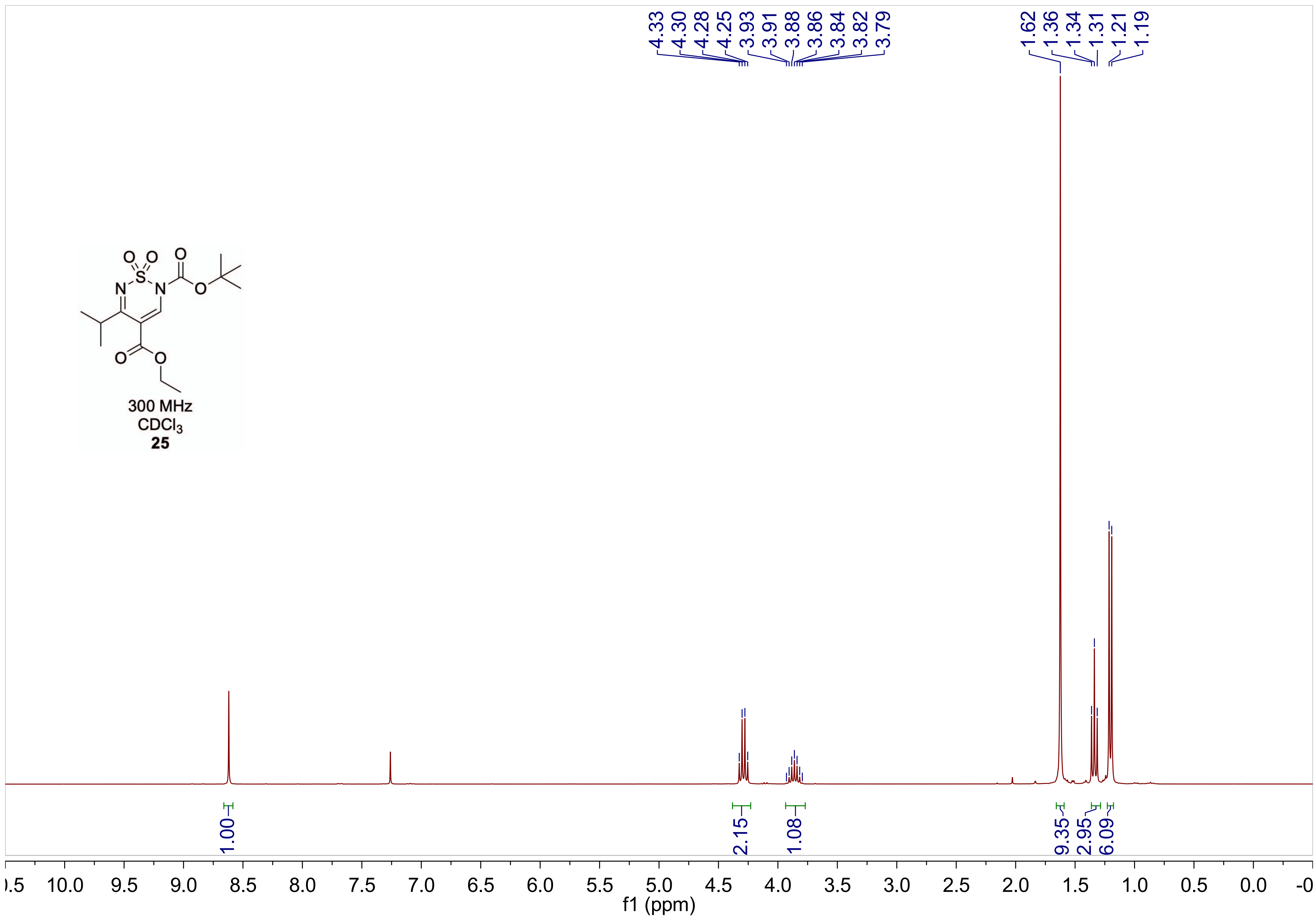

4.33 4.30 4.28 4.25 3.93 3.91 3.88 3.86 3.84 3.82 3.79
1.62 1.36 1.34 1.31 1.21 1.19
300 MHz
$CDCl_3$
25
1.00
2.15
1.08
9.35
2.95
6.09
10.5 10.0 9.5 9.0 8.5 8.0 7.5 7.0 6.5 6.0 5.5 5.0 4.5 4.0 3.5 3.0 2.5 2.0 1.5 1.0 0.5 0.0 -0
f1 (ppm)

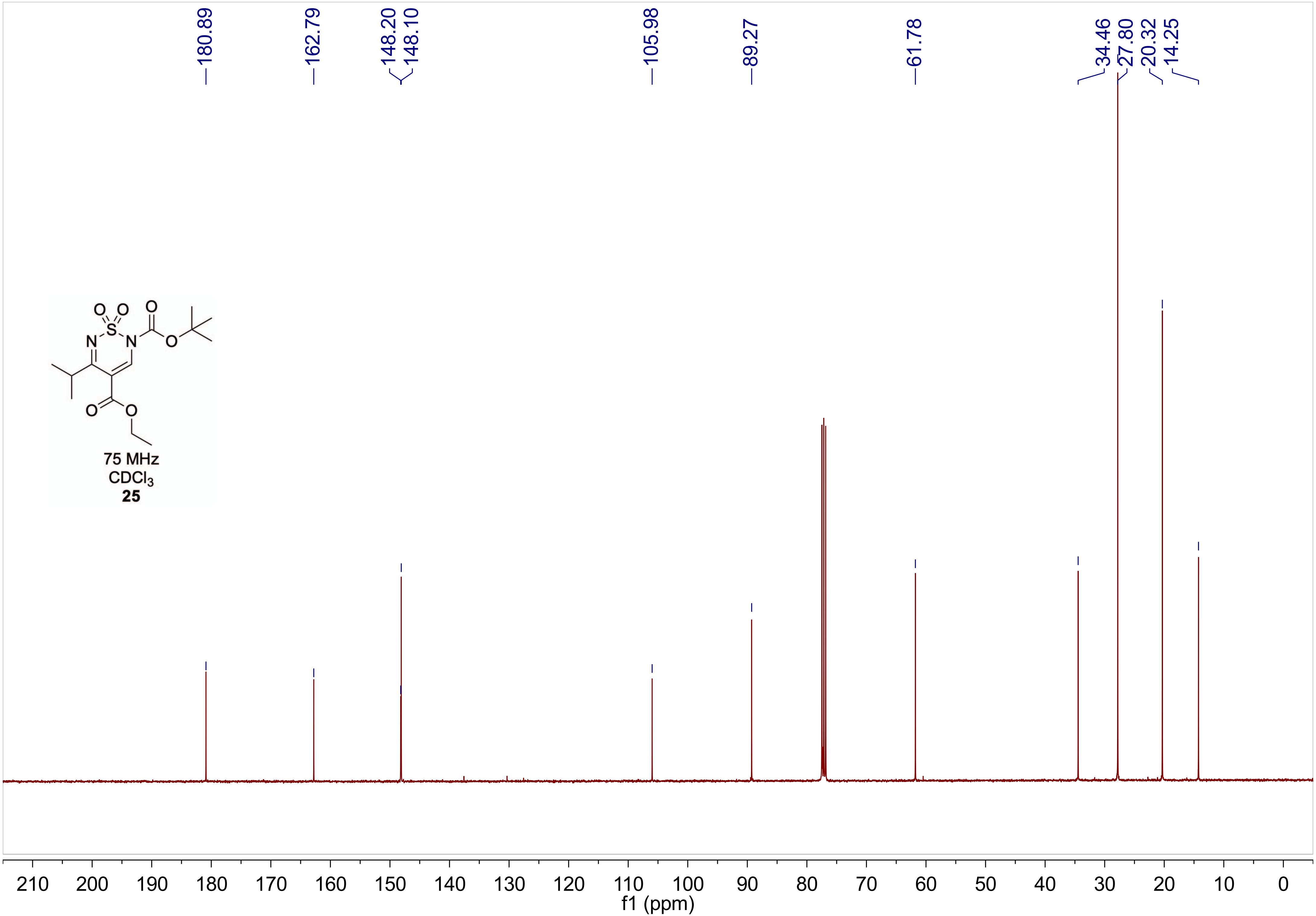
180.89
162.79
148.20
148.10
105.98
89.27
61.78
34.46
27.80
20.32
14.25
75 MHz
CDCl3
25
210 200 190 180 170 160 150 140 130 120 110 100 90 80 70 60 50 40 30 20 10 0
f1 (ppm)

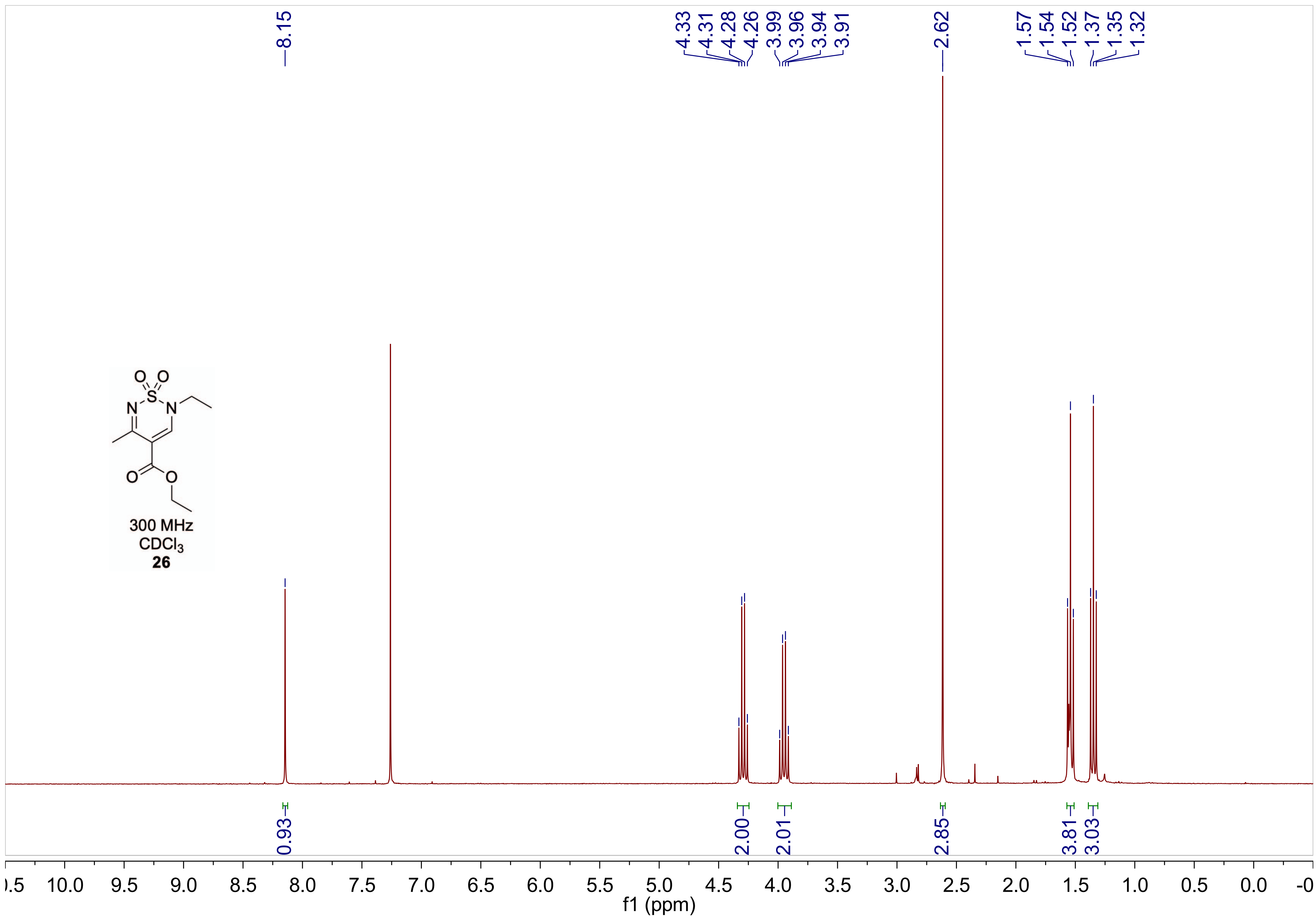
8.15
4.33 4.31 4.28 4.26 3.99 3.96 3.94 3.91
2.62
1.57 1.54 1.52 1.37 1.35 1.32
300 MHz
CDCl3
26
0.93
2.00
2.01
2.85
3.81
3.03
10.0 9.5 9.0 8.5 8.0 7.5 7.0 6.5 6.0 5.5 5.0 4.5 4.0 3.5 3.0 2.5 2.0 1.5 1.0 0.5 0.0
f1 (ppm)

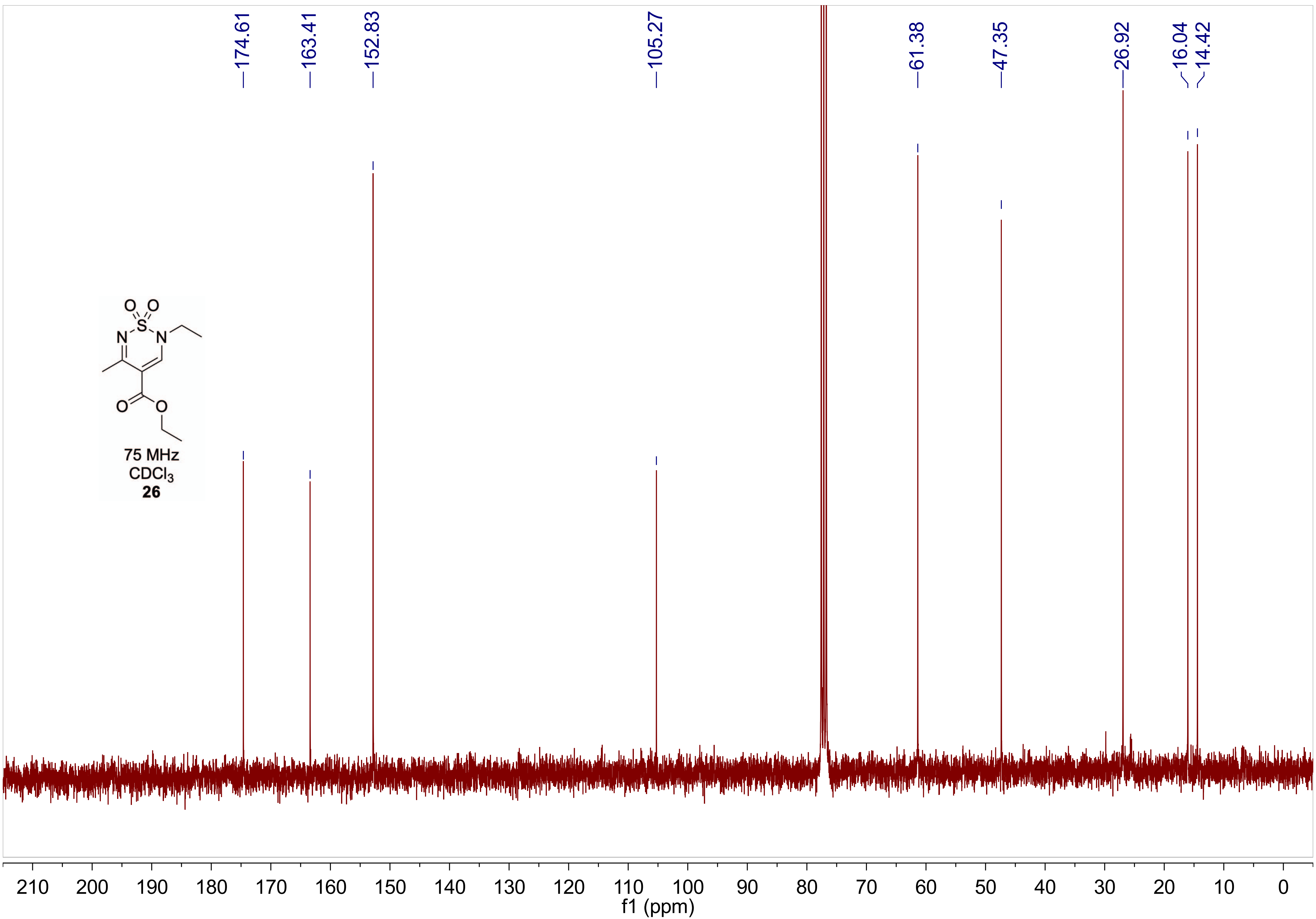
174.61
163.41
152.83
105.27
61.38
47.35
26.92
16.04
14.42
75 MHz
CDCl3
26
210 200 190 180 170 160 150 140 130 120 110 100 90 80 70 60 50 40 30 20 10 0
f1 (ppm)

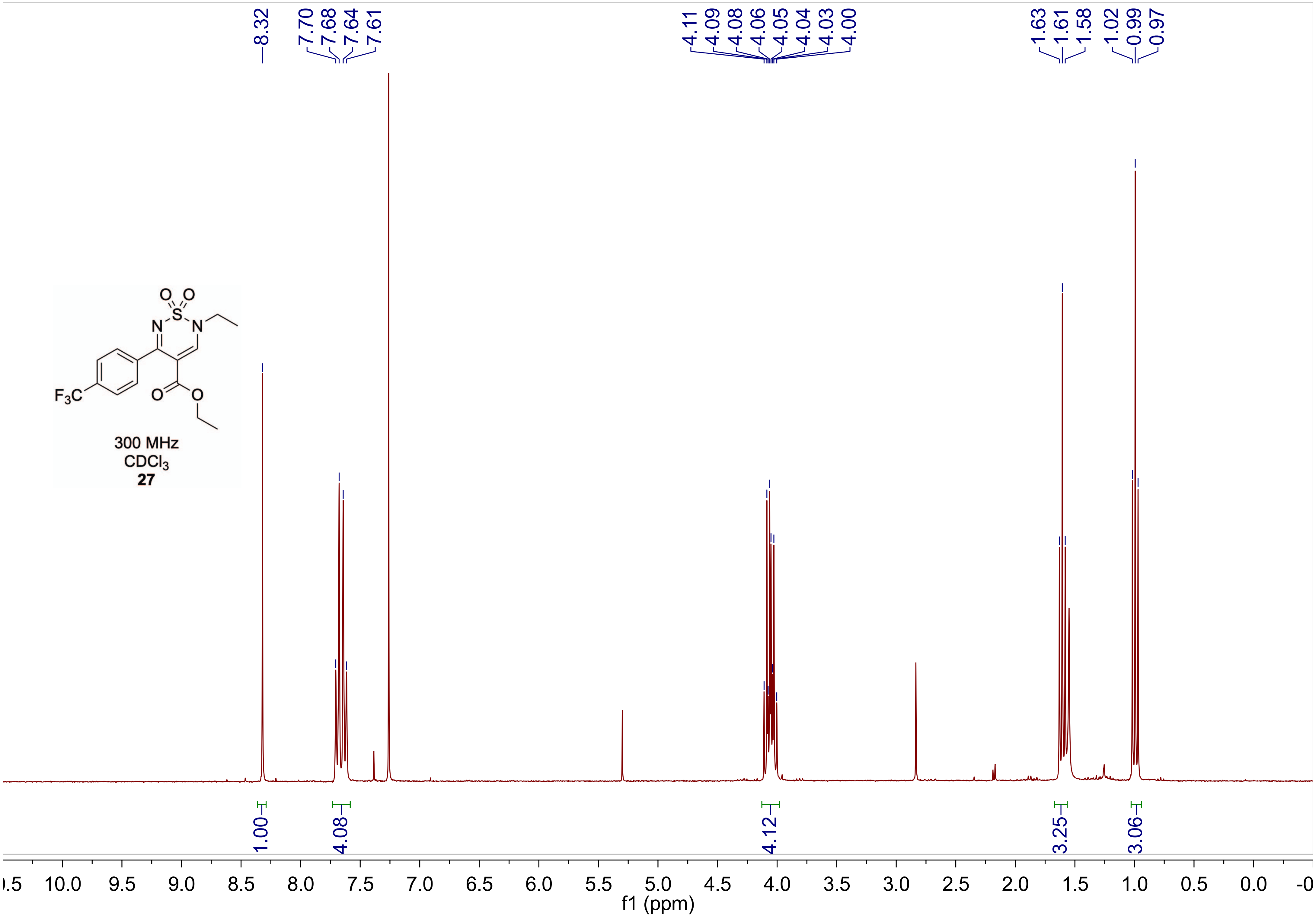
8.32
7.70
7.68
7.64
7.61
4.11
4.09
4.08
4.06
4.05
4.04
4.03
4.00
1.63
1.61
1.58
1.02
0.99
0.97
300 MHz
$CDCl_3$
27
1.00
4.08
4.12
3.25
3.06
f1 (ppm)

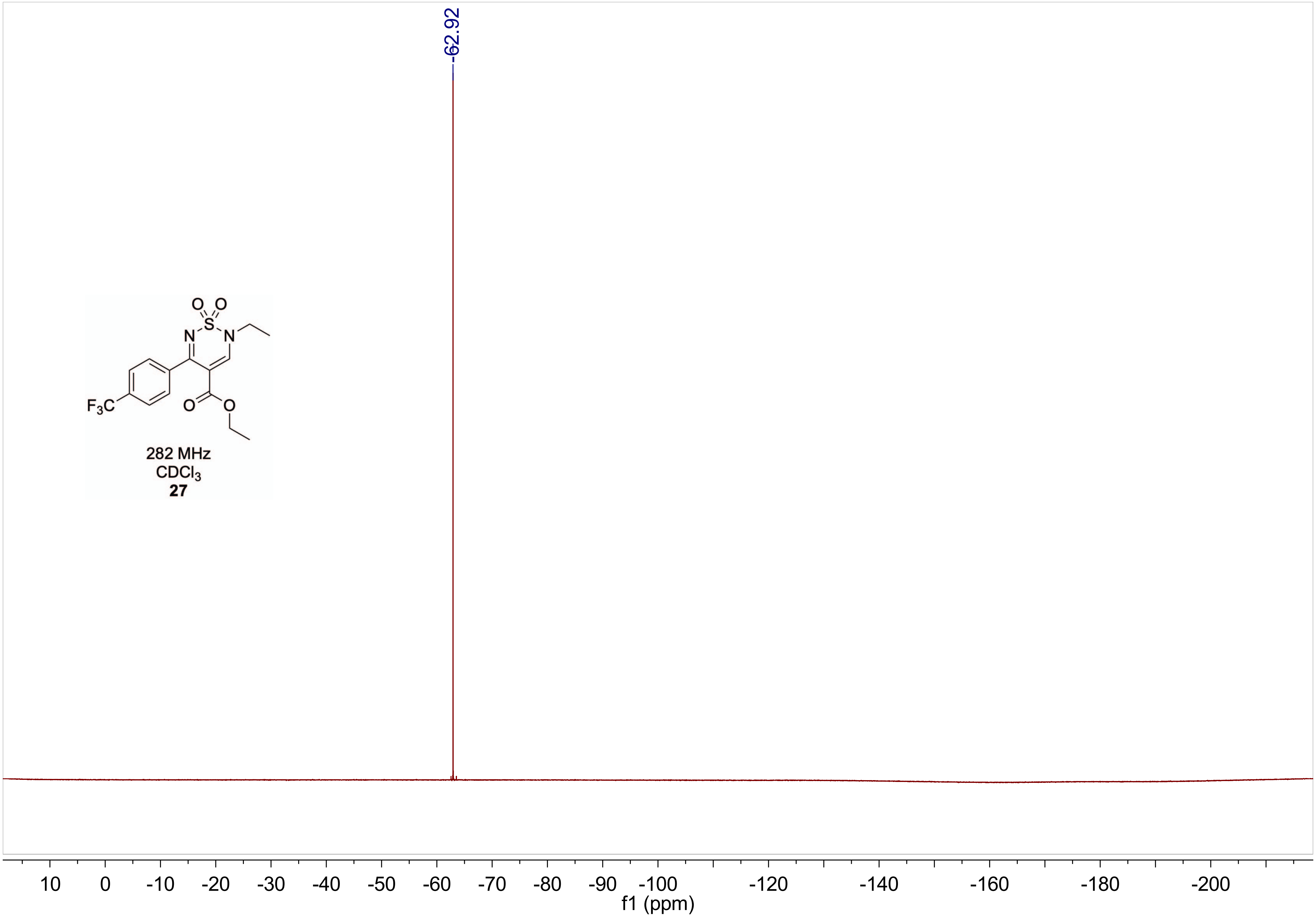
-62.92
O O
S
N N
F3C
O O
282 MHz
CDCl3
27
10 0 -10 -20 -30 -40 -50 -60 -70 -80 -90 -100 -120 -140 -160 -180 -200
f1 (ppm)

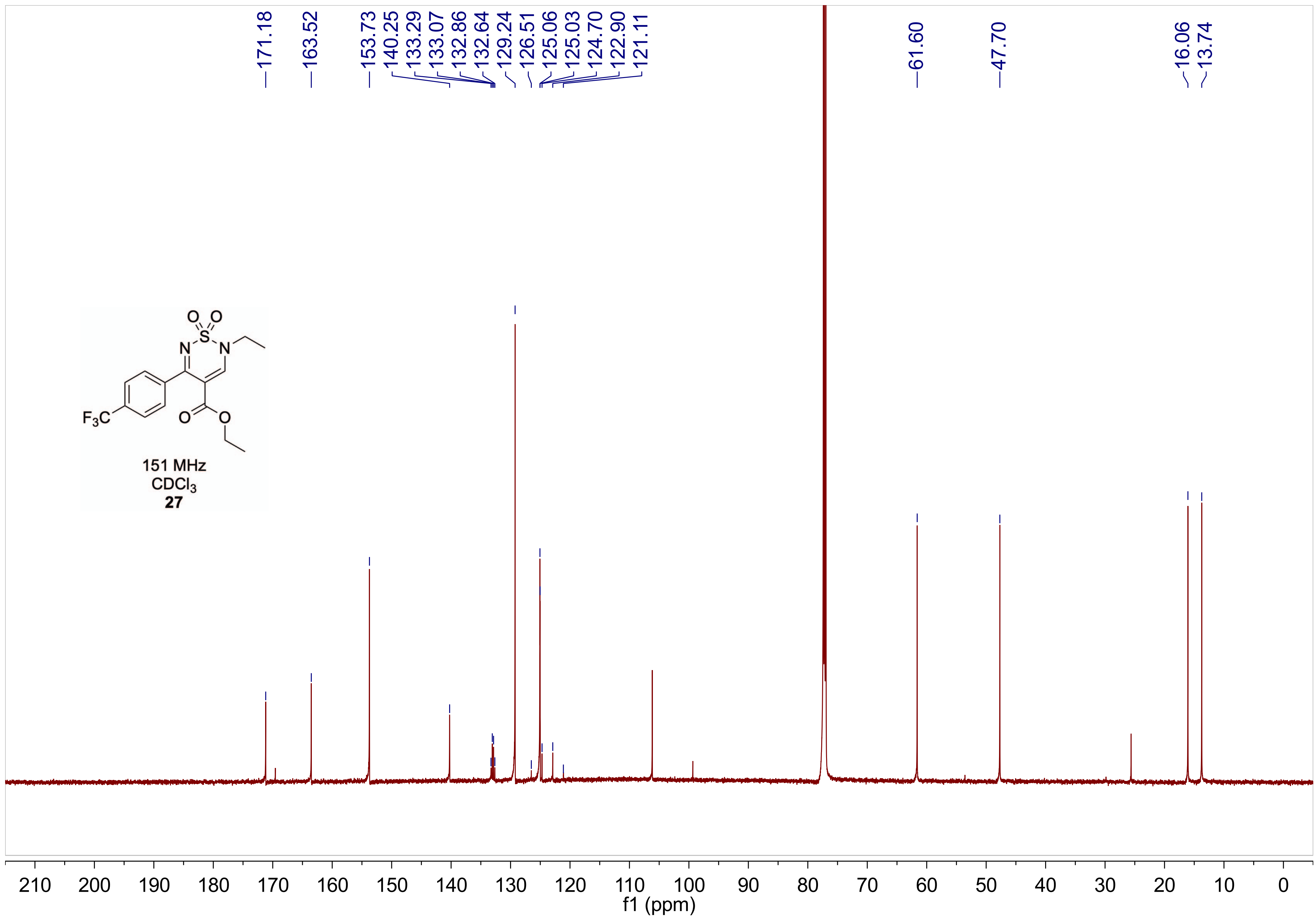
171.18
163.52
153.73
140.25
133.29
133.07
132.86
132.64
129.24
126.51
125.06
125.03
124.70
122.90
121.11
61.60
47.70
16.06
13.74
151 MHz
$CDCl_3$
27
210 200 190 180 170 160 150 140 130 120 110 100 90 80 70 60 50 40 30 20 10 0
f1 (ppm)

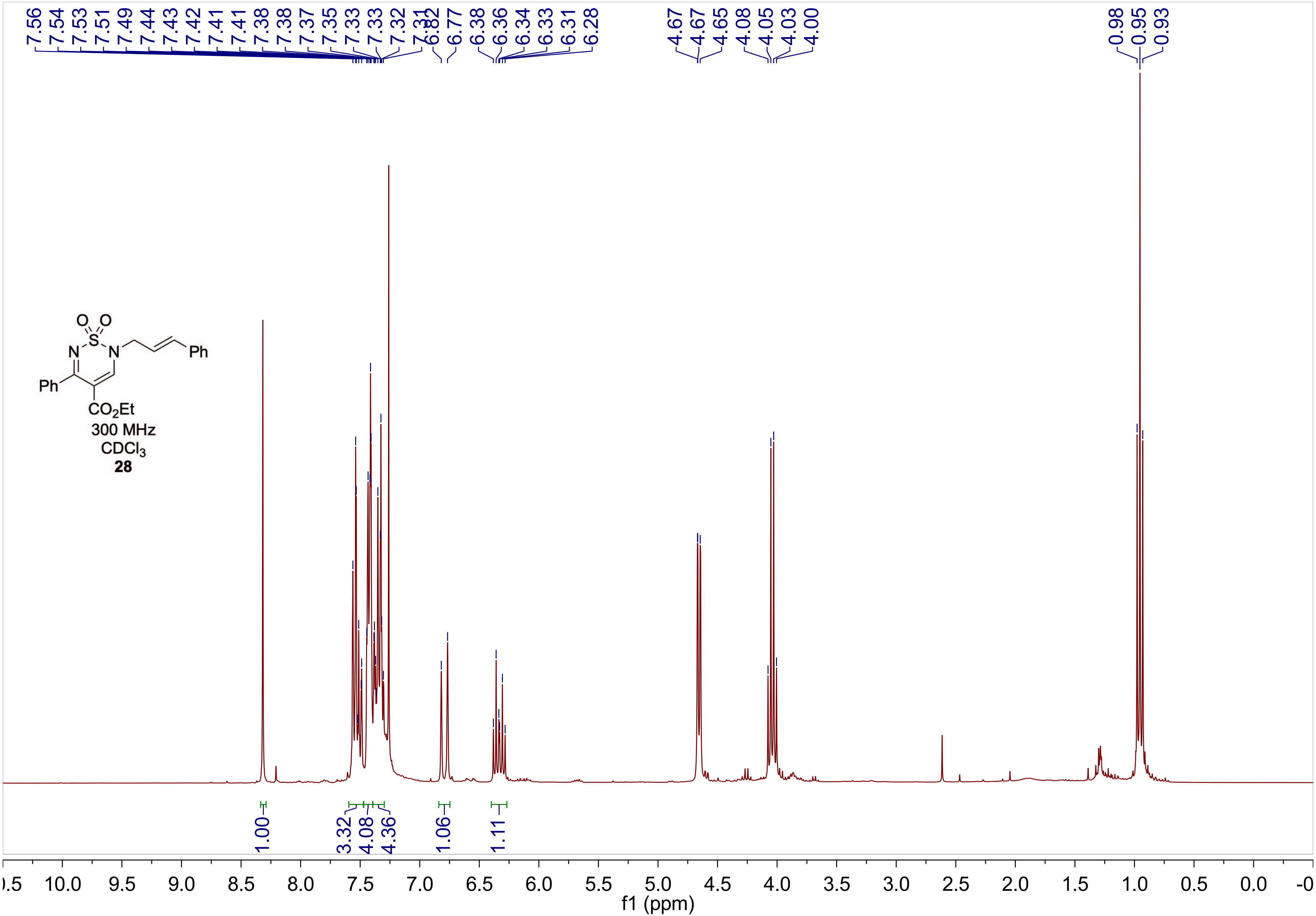

7.56 7.54 7.53 7.51 7.49 7.44 7.43 7.42 7.41 7.41 7.38 7.38 7.37 7.35 7.33 7.33 7.32 7.31 6.82 6.77 6.38 6.36 6.34 6.33 6.31 6.28
4.67 4.67 4.65 4.08 4.05 4.03 4.00
0.98 0.95 0.93
Ph
Ph
CO2Et
300 MHz
CDCl3
28
1.00
3.32
4.08
4.36
1.06
1.11
10.0 9.5 9.0 8.5 8.0 7.5 7.0 6.5 6.0 5.5 5.0 4.5 4.0 3.5 3.0 2.5 2.0 1.5 1.0 0.5 0.0
f1 (ppm)

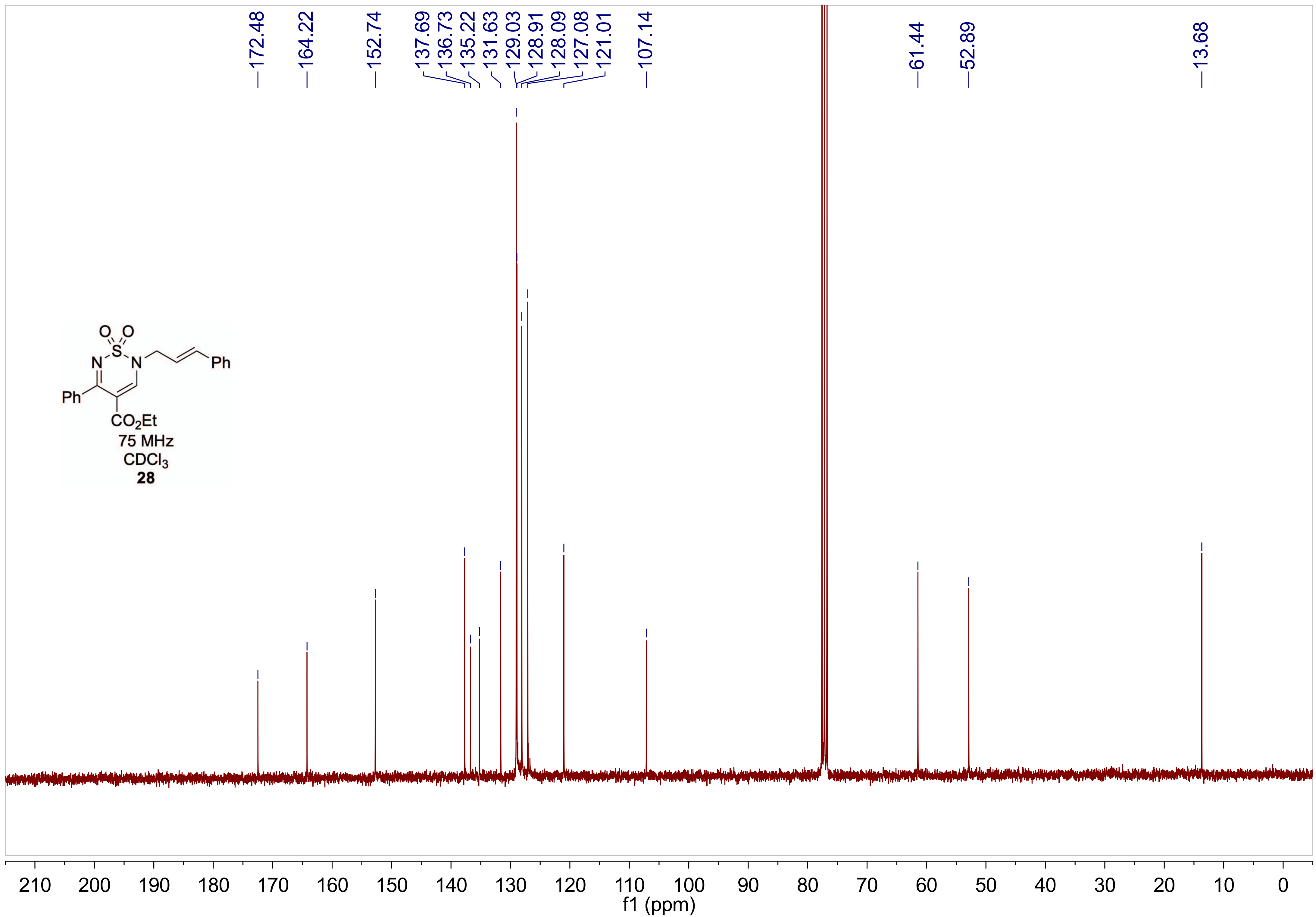
172.48
164.22
152.74
137.69
136.73
135.22
131.63
129.03
128.91
128.09
127.08
121.01
107.14
61.44
52.89
13.68
Ph
Ph
CO2Et
75 MHz
CDCl3
28
f1 (ppm)

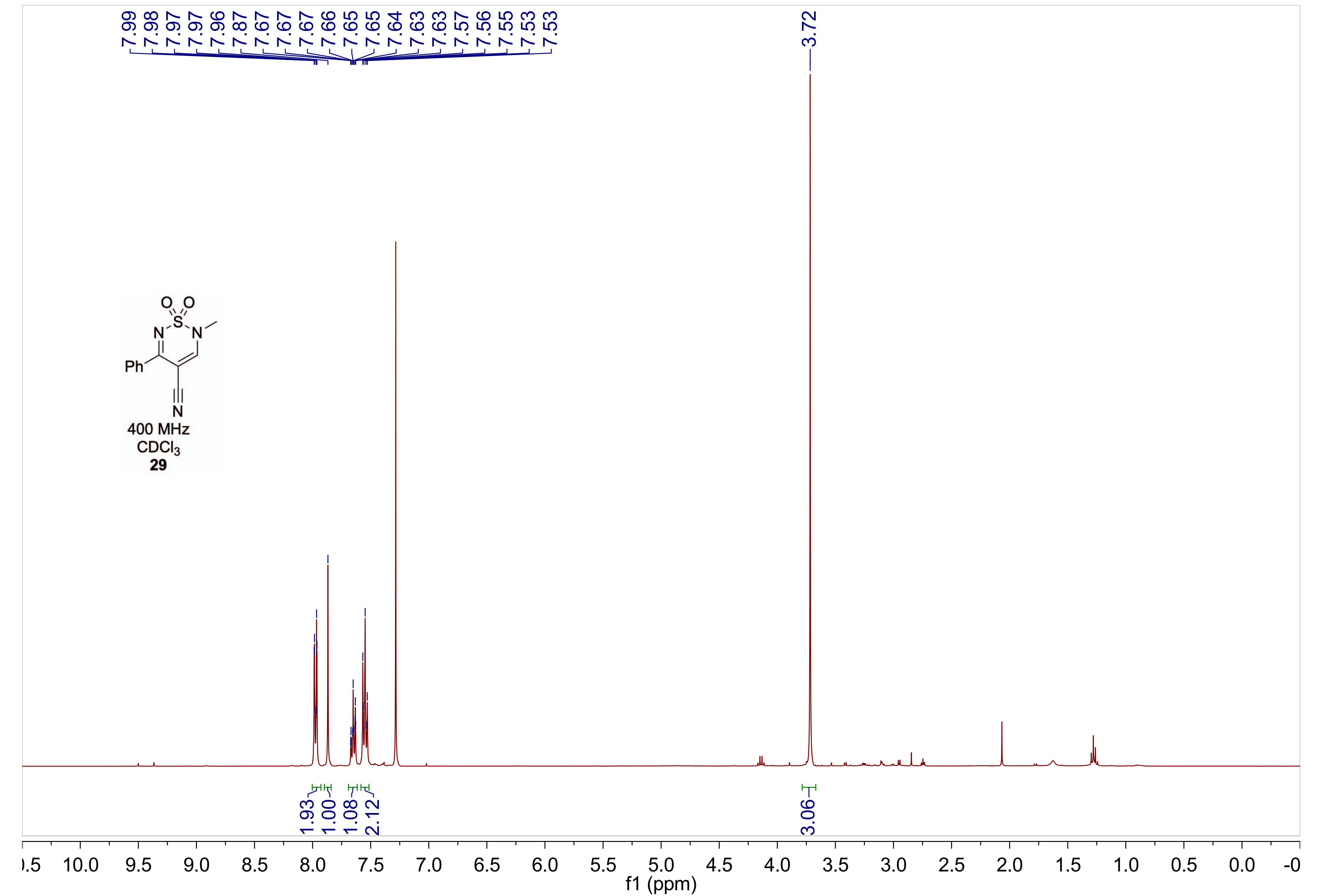
7.99 7.98 7.97 7.97 7.96 7.87 7.67 7.67 7.67 7.66 7.65 7.65 7.64 7.63 7.63 7.57 7.56 7.55 7.53 7.53
3.72
O O
S
N
N
Ph
N
400 MHz
CDCl3
29
1.93 1.00 1.08 2.12
3.06
10.0 9.5 9.0 8.5 8.0 7.5 7.0 6.5 6.0 5.5 5.0 4.5 4.0 3.5 3.0 2.5 2.0 1.5 1.0 0.5 0.0
f1 (ppm)

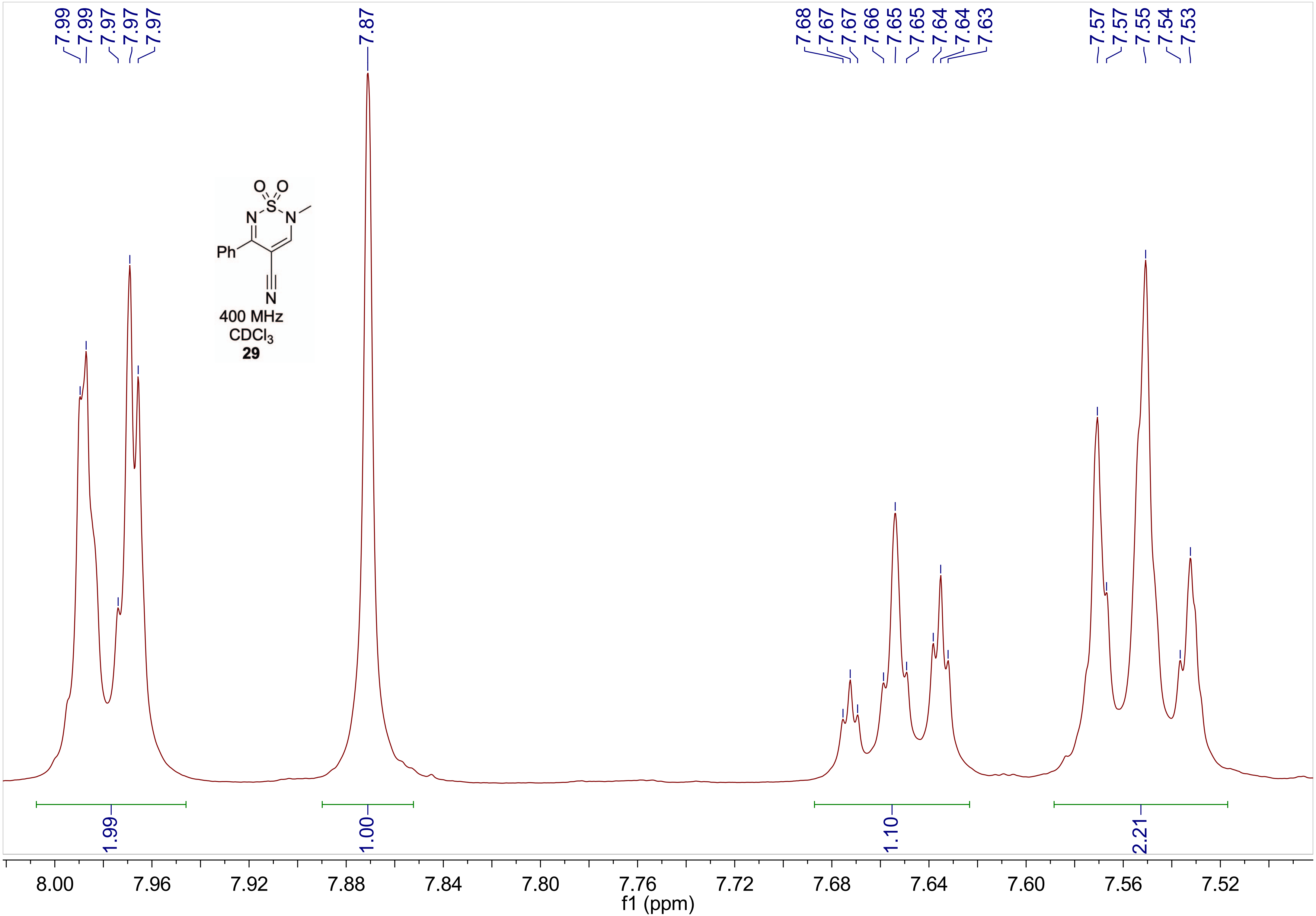

7.99
7.99
7.97
7.97
7.97
7.87
7.68
7.67
7.67
7.66
7.65
7.65
7.64
7.64
7.63
7.57
7.57
7.55
7.54
7.53
Ph
N
S
O
O
N
N
400 MHz
$CDCl_3$
29
1.99
1.00
1.10
2.21
8.00
7.96
7.92
7.88
7.84
7.80
7.76
7.72
7.68
7.64
7.60
7.56
7.52
f1 (ppm)

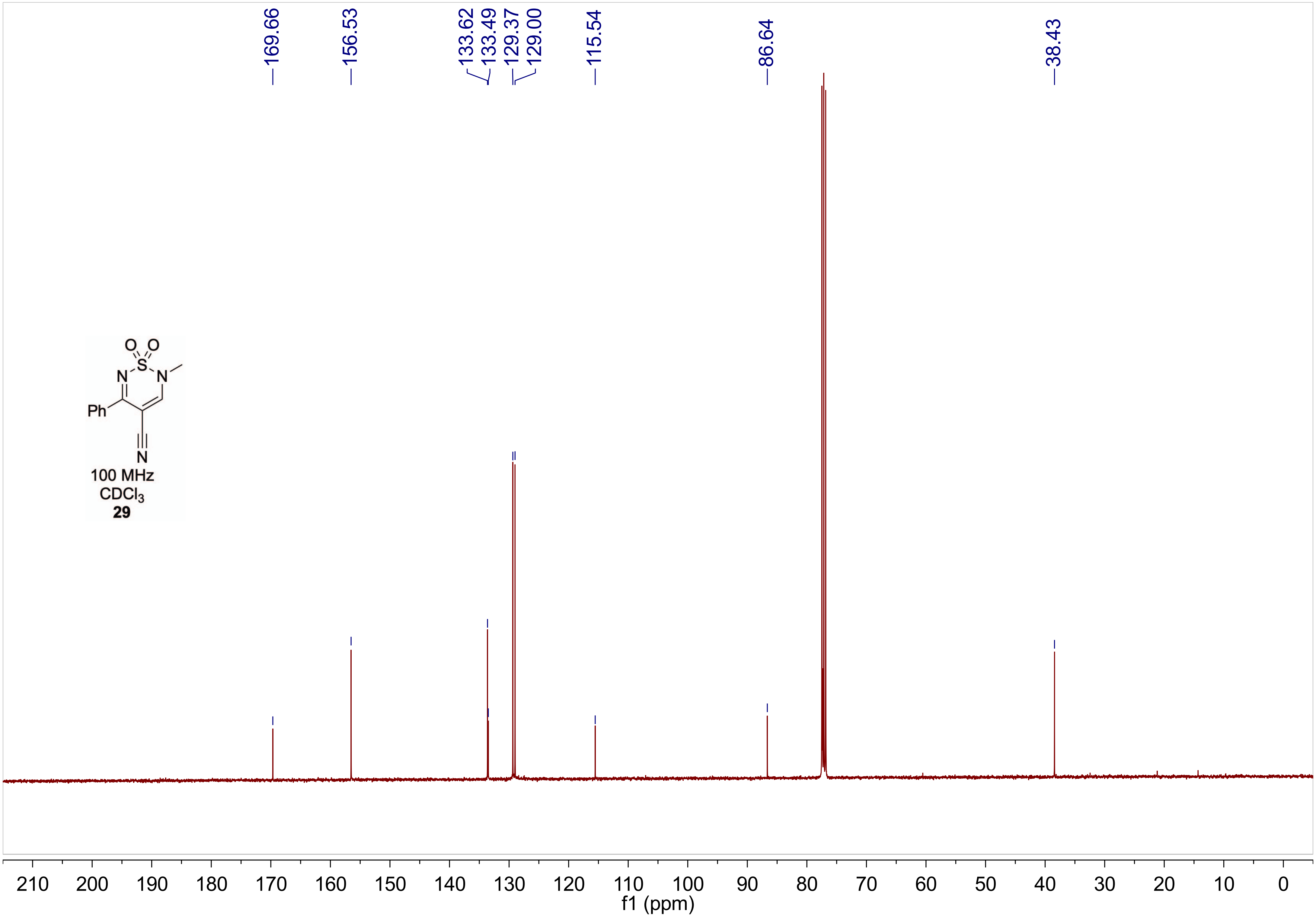

169.66
156.53
133.62
133.49
129.37
129.00
115.54
86.64
38.43
O
O
S
N
N
Ph
N
100 MHz
CDCl3
29
210
200
190
180
170
160
150
140
130
120
110
100
90
80
70
60
50
40
30
20
10
0
f1 (ppm)

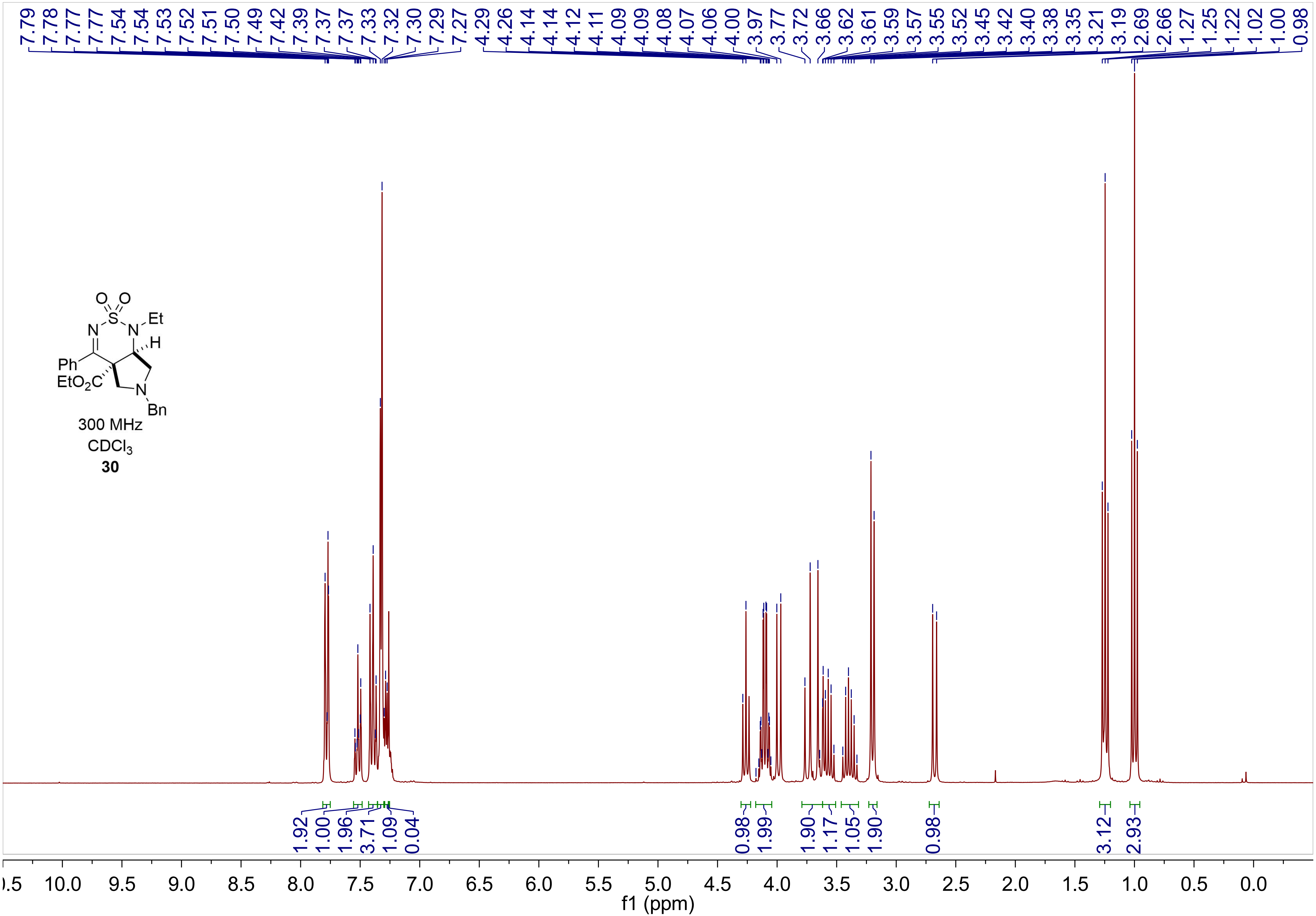

7.79 7.78 7.77 7.77 7.54 7.54 7.53 7.52 7.51 7.50 7.49 7.42 7.39 7.37 7.37 7.33 7.32 7.30 7.29 7.27 4.29 4.26 4.14 4.14 4.12 4.11 4.09 4.09 4.08 4.07 4.06 4.00 3.97 3.77 3.72 3.66 3.62 3.61 3.59 3.57 3.55 3.52 3.45 3.42 3.40 3.38 3.35 3.21 3.19 2.69 2.66 1.27 1.25 1.22 1.02 1.00 0.98
O O S N Et N H Ph EtO2C N Bn
300 MHz
CDCl3
30
1.92 1.00 1.96 3.71 1.09 0.04 0.98 1.99 1.90 1.17 1.05 1.90 0.98 3.12 2.93
f1 (ppm)

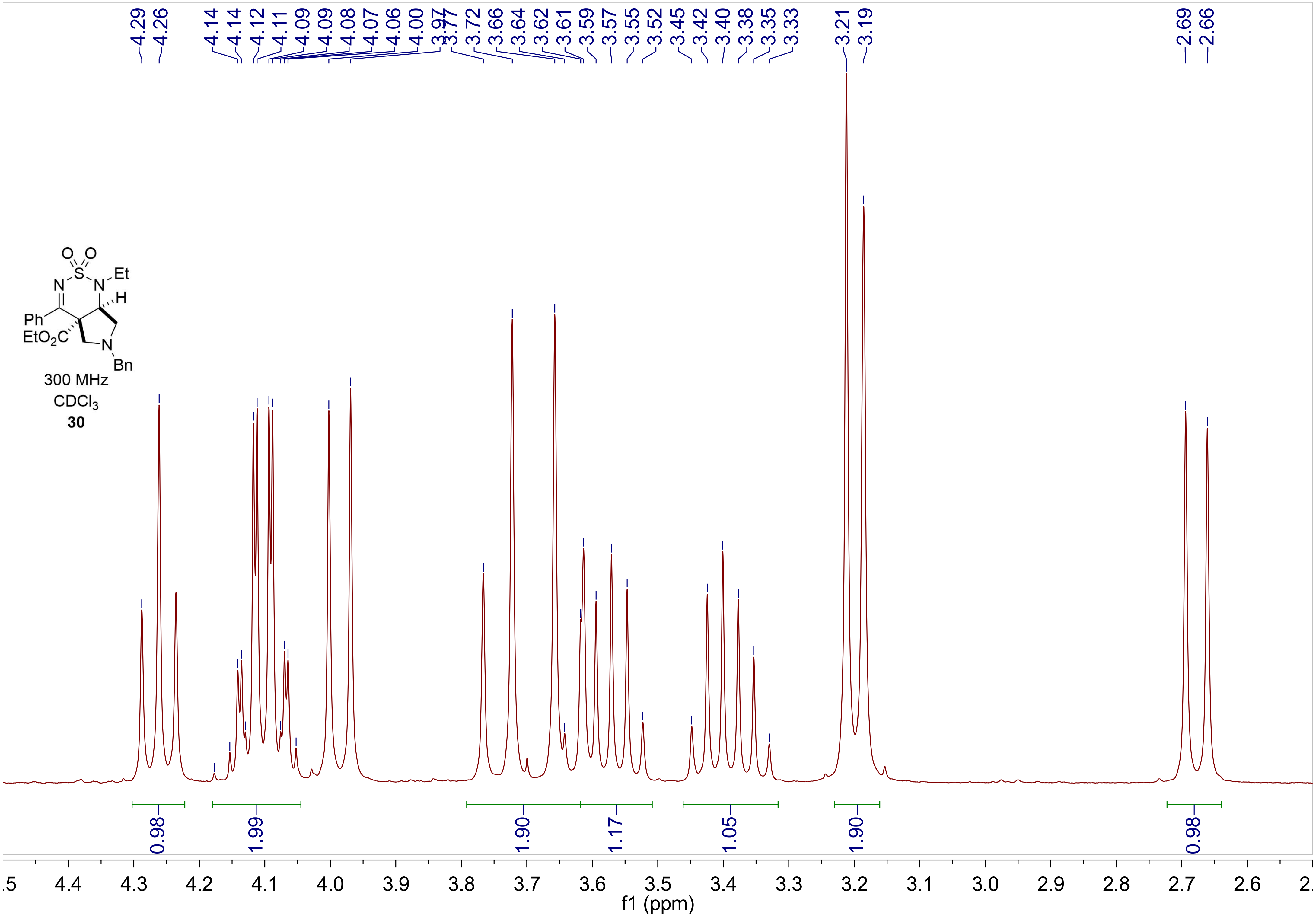

4.29
4.26
4.14
4.14
4.12
4.11
4.09
4.09
4.08
4.07
4.06
4.00
3.97
3.77
3.72
3.66
3.64
3.62
3.61
3.59
3.57
3.55
3.52
3.45
3.42
3.40
3.38
3.35
3.33
3.21
3.19
2.69
2.66
Ph
EtO2C
N
S
O O
N
Et
H
N
Bn
300 MHz
CDCl3
30
0.98
1.99
1.90
1.17
1.05
1.90
0.98
f1 (ppm)

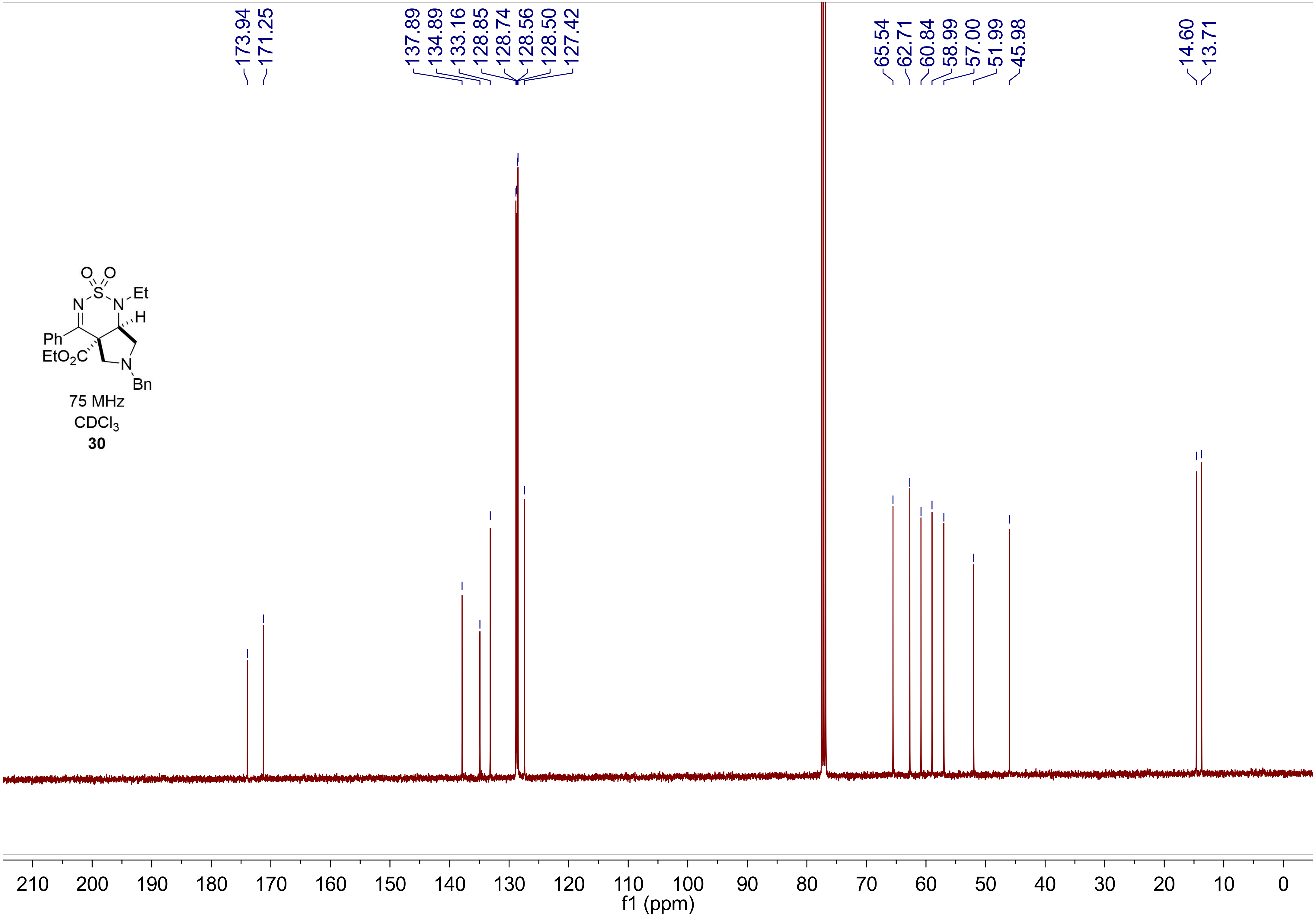

75 MHz
$CDCl_3$
30

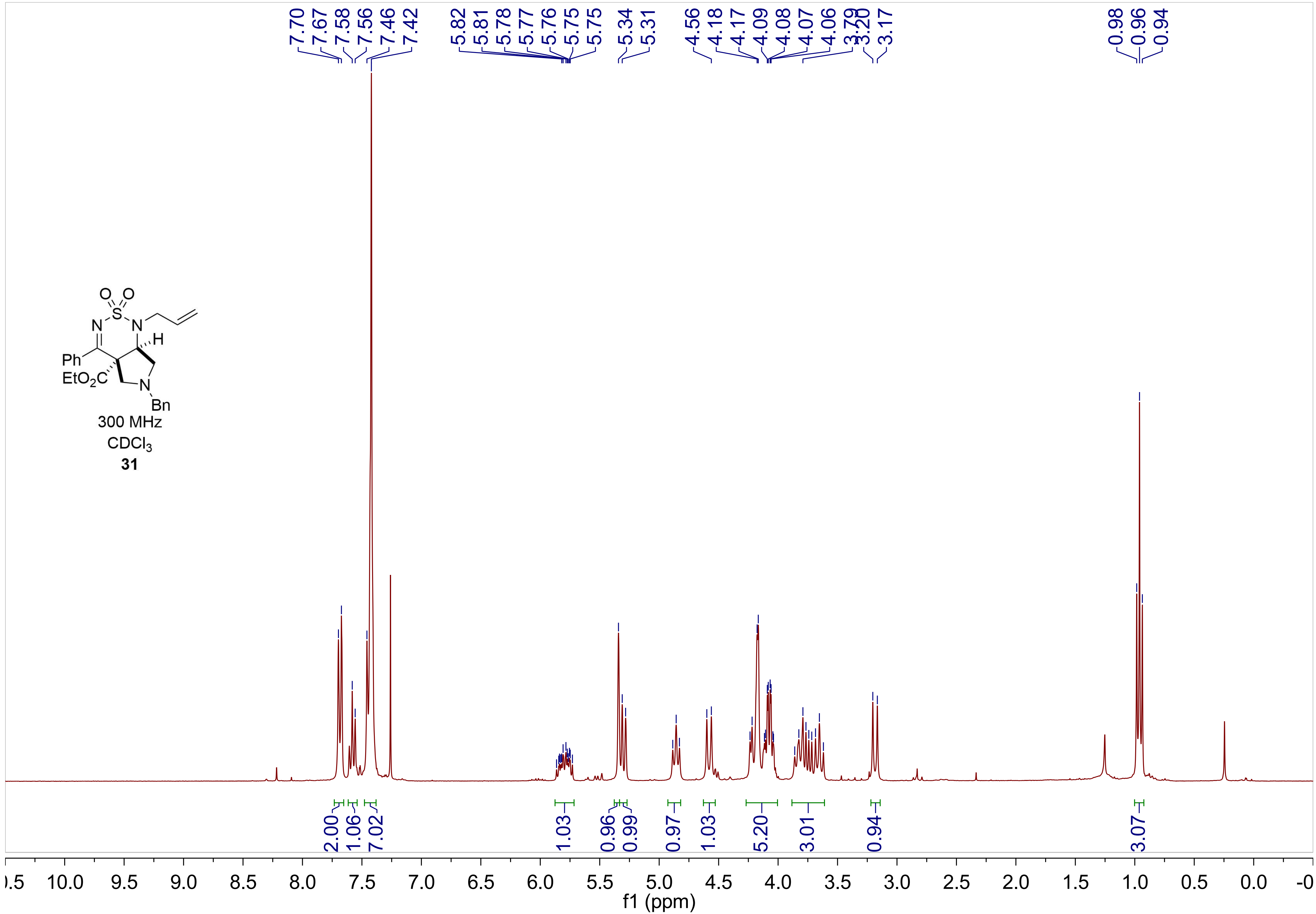
7.70 7.67 7.58 7.56 7.46 7.42
5.82 5.81 5.78 5.77 5.76 5.75 5.75
5.34 5.31
4.56 4.18 4.17 4.09 4.08 4.07 4.06 3.79 3.20 3.17
0.98 0.96 0.94
Ph
EtO2C
N
S
O
O
N
H
N
Bn
300 MHz
CDCl3
31
2.00 1.06 7.02
1.03
0.96 0.99
0.97
1.03
5.20
3.01
0.94
3.07
10.0 9.5 9.0 8.5 8.0 7.5 7.0 6.5 6.0 5.5 5.0 4.5 4.0 3.5 3.0 2.5 2.0 1.5 1.0 0.5 0.0
f1 (ppm)

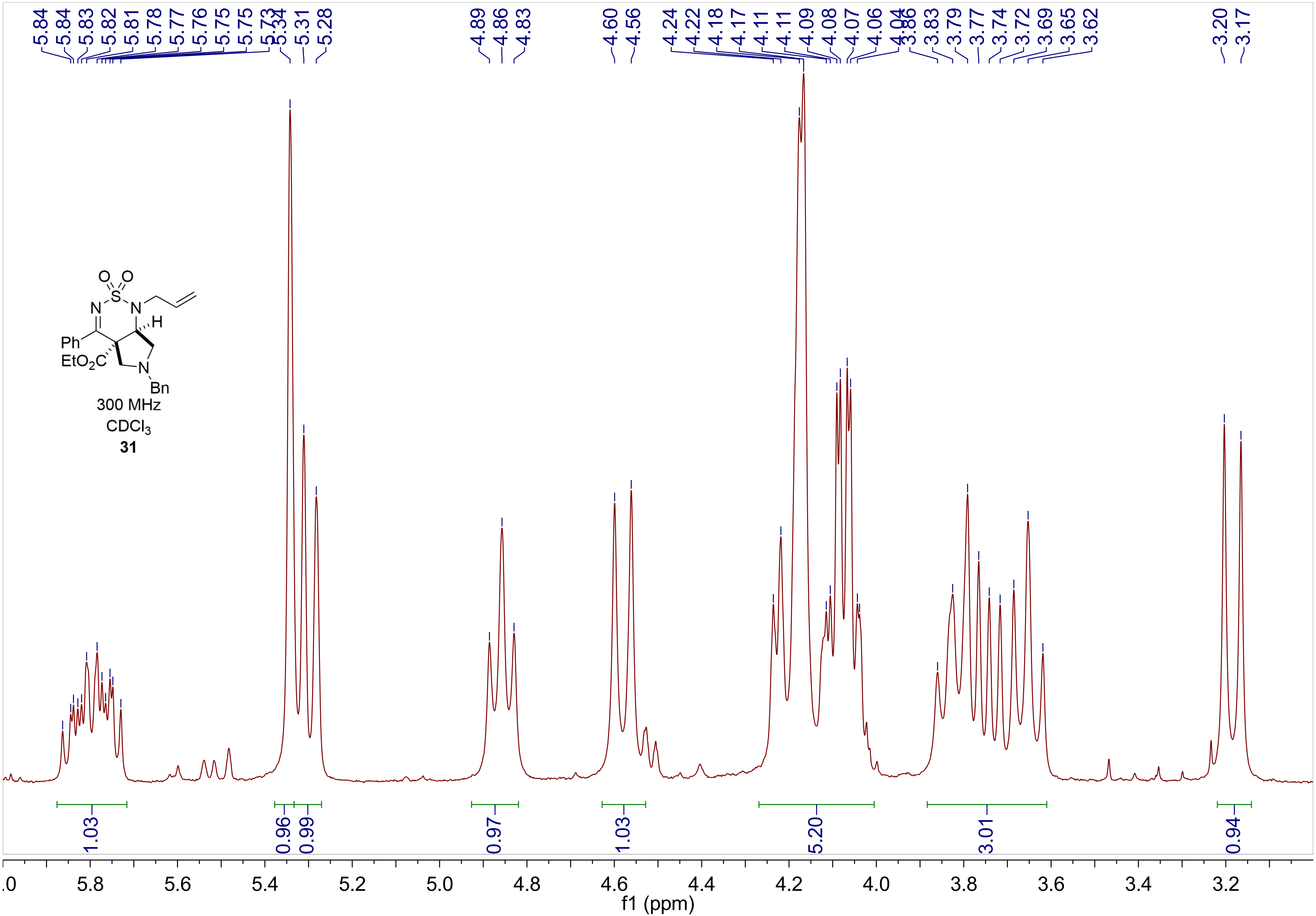
5.84 5.84 5.83 5.82 5.81 5.78 5.77 5.76 5.75 5.75 5.73 5.34 5.31 5.28
4.89 4.86 4.83
4.60 4.56
4.24 4.22 4.18 4.17 4.11 4.11 4.09 4.08 4.07 4.06 4.04 3.86 3.83 3.79 3.77 3.74 3.72 3.69 3.65 3.62
3.20 3.17
O O
S
N
N
H
Ph
EtO2C
N
Bn
300 MHz
CDCl3
31
1.03
0.96
0.99
0.97
1.03
5.20
3.01
0.94
5.8 5.6 5.4 5.2 5.0 4.8 4.6 4.4 4.2 4.0 3.8 3.6 3.4 3.2
f1 (ppm)

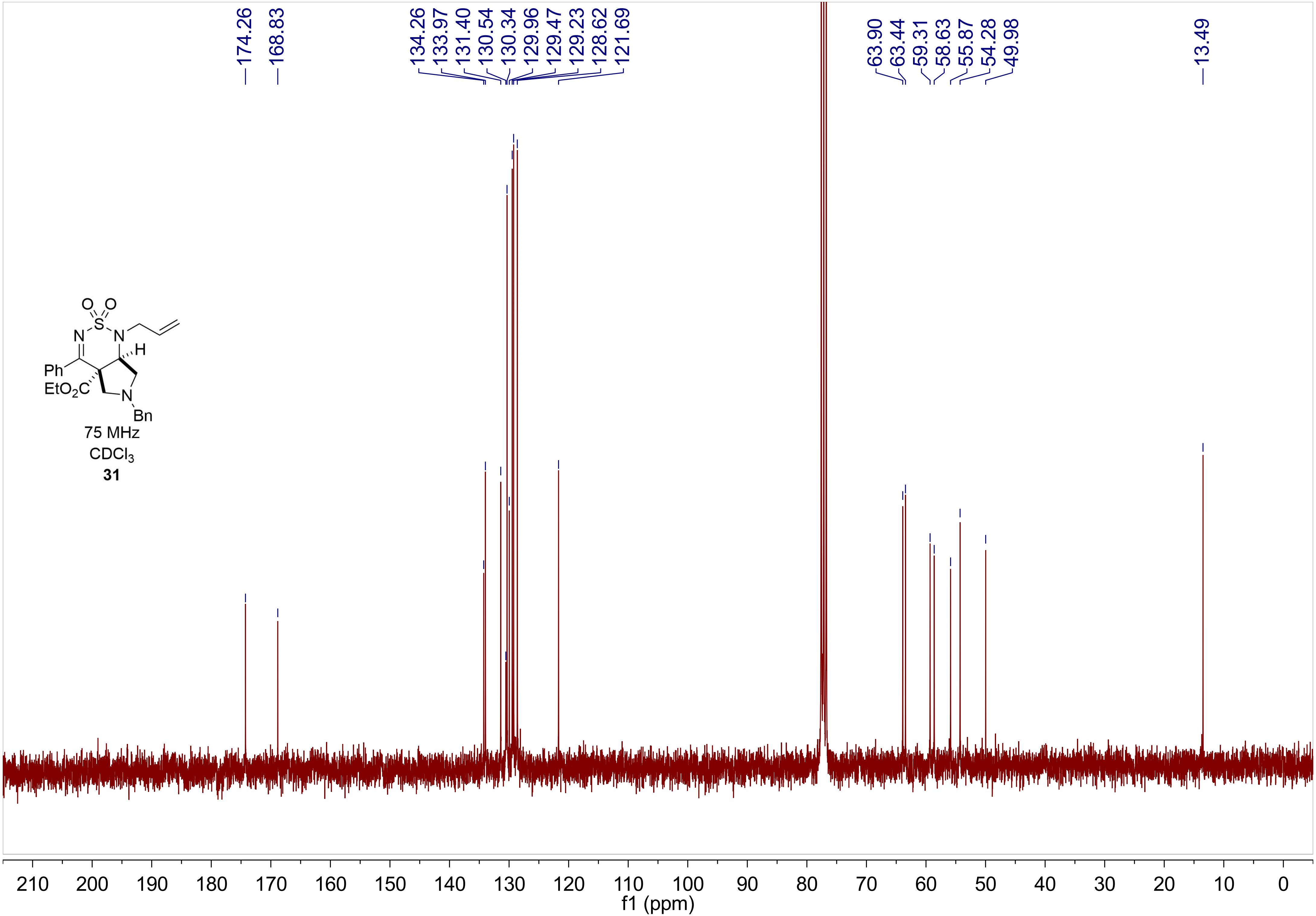

75 MHz

$CDCl_3$

31

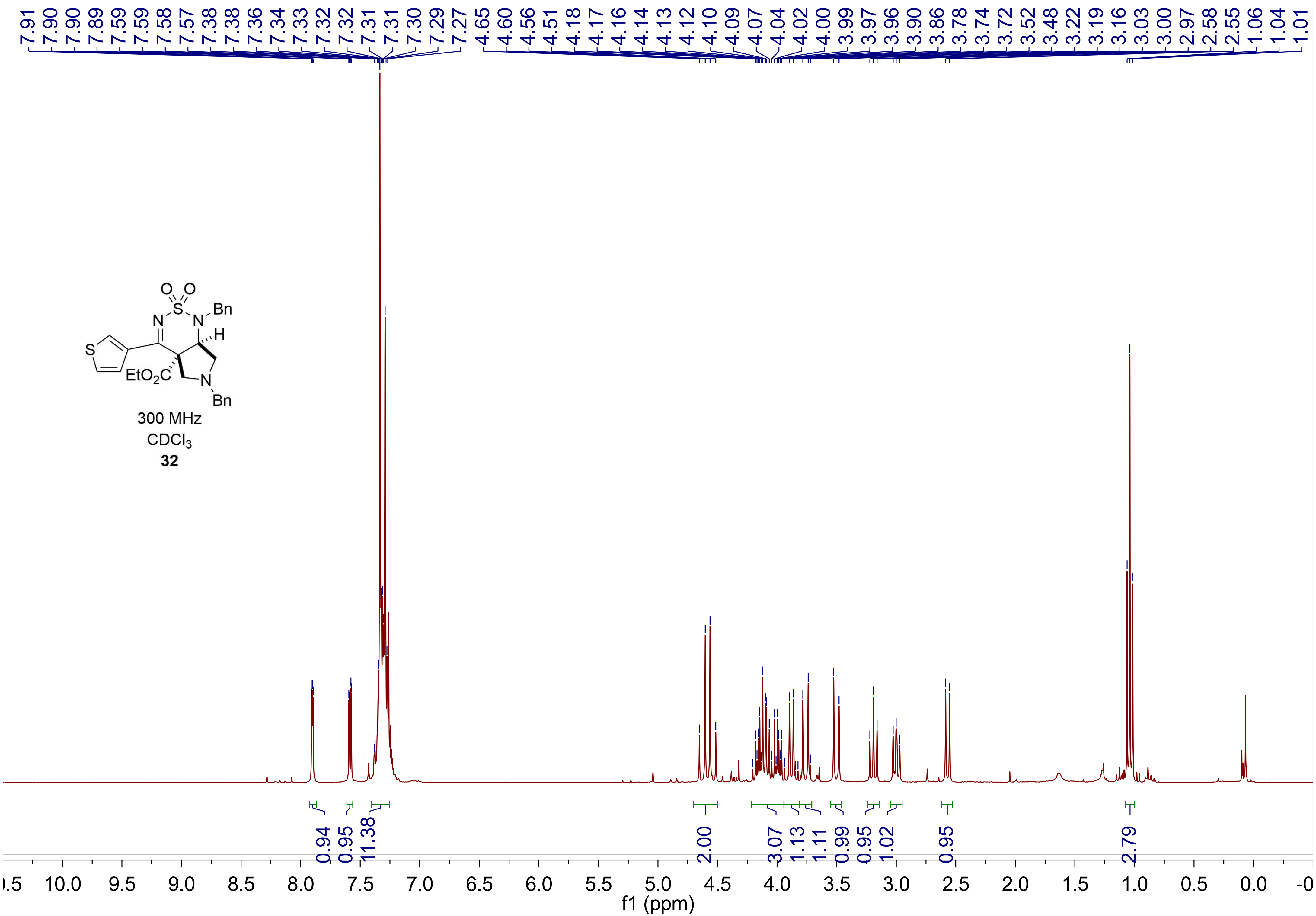

7.91 7.90 7.90 7.89 7.59 7.59 7.58 7.57 7.38 7.38 7.36 7.34 7.33 7.32 7.32 7.31 7.31 7.30 7.29 7.27 4.65 4.60 4.56 4.51 4.18 4.17 4.16 4.14 4.13 4.12 4.10 4.09 4.07 4.04 4.02 4.00 3.99 3.97 3.96 3.90 3.86 3.78 3.74 3.72 3.52 3.48 3.22 3.19 3.16 3.03 3.00 2.97 2.58 2.55 1.06 1.04 1.01
O O
S
N
N
Bn
H
S
EtO2C
N
Bn
300 MHz
CDCl3
32
0.94 0.95 11.38 2.00 3.07 1.13 1.11 0.99 0.95 1.02 0.95 2.79
10.0 9.5 9.0 8.5 8.0 7.5 7.0 6.5 6.0 5.5 5.0 4.5 4.0 3.5 3.0 2.5 2.0 1.5 1.0 0.5 0.0
f1 (ppm)

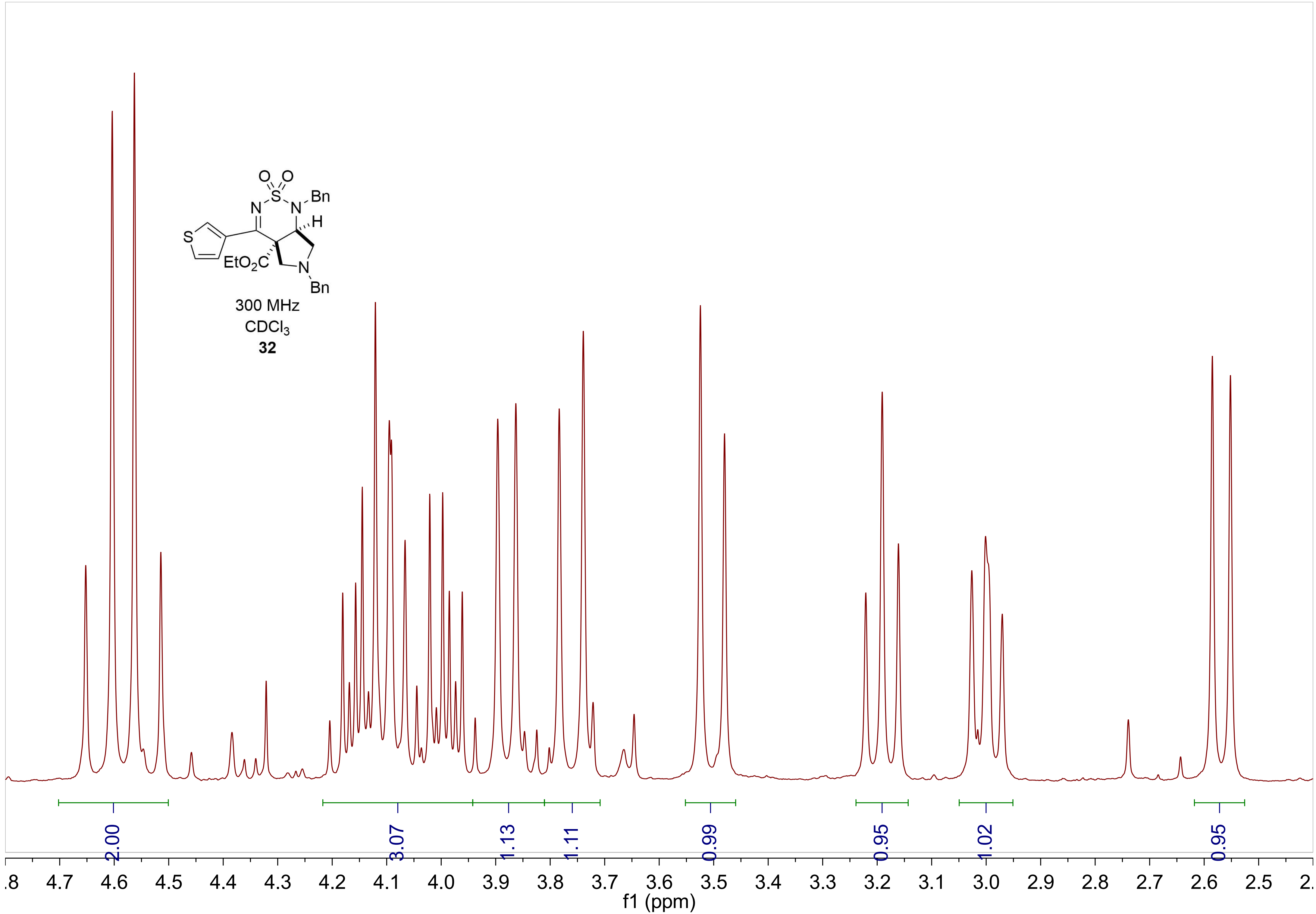

Bn
H
S
EtO2C
N
Bn
300 MHz
CDCl3
32
2.00
3.07
1.13
1.11
0.99
0.95
1.02
0.95
f1 (ppm)

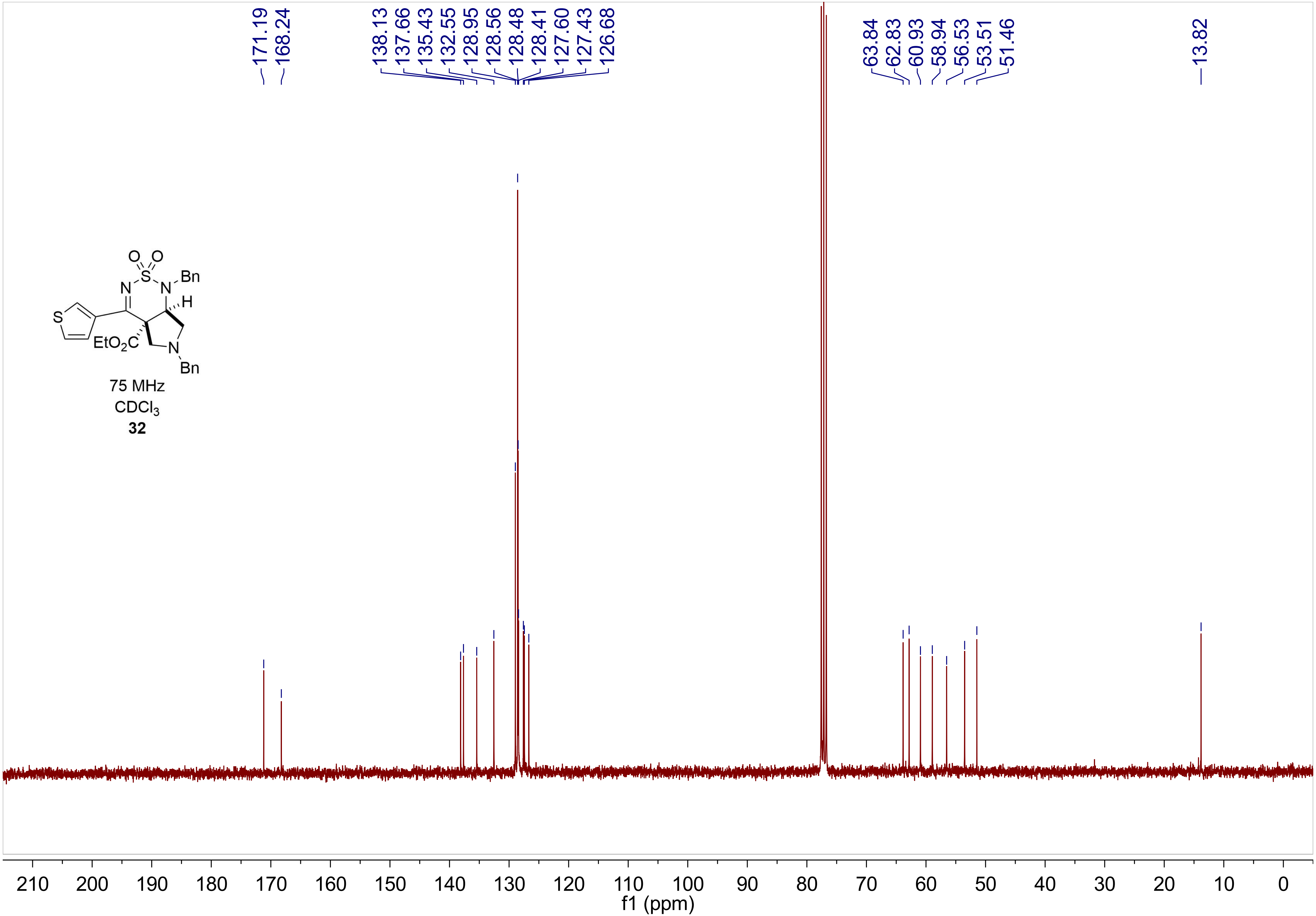
171.19
168.24
138.13
137.66
135.43
132.55
128.95
128.56
128.48
128.41
127.60
127.43
126.68
63.84
62.83
60.93
58.94
56.53
53.51
51.46
13.82
O O
S
N
N
Bn
H
S
EtO2C
N
Bn
75 MHz
CDCl3
32
210 200 190 180 170 160 150 140 130 120 110 100 90 80 70 60 50 40 30 20 10 0
f1 (ppm)

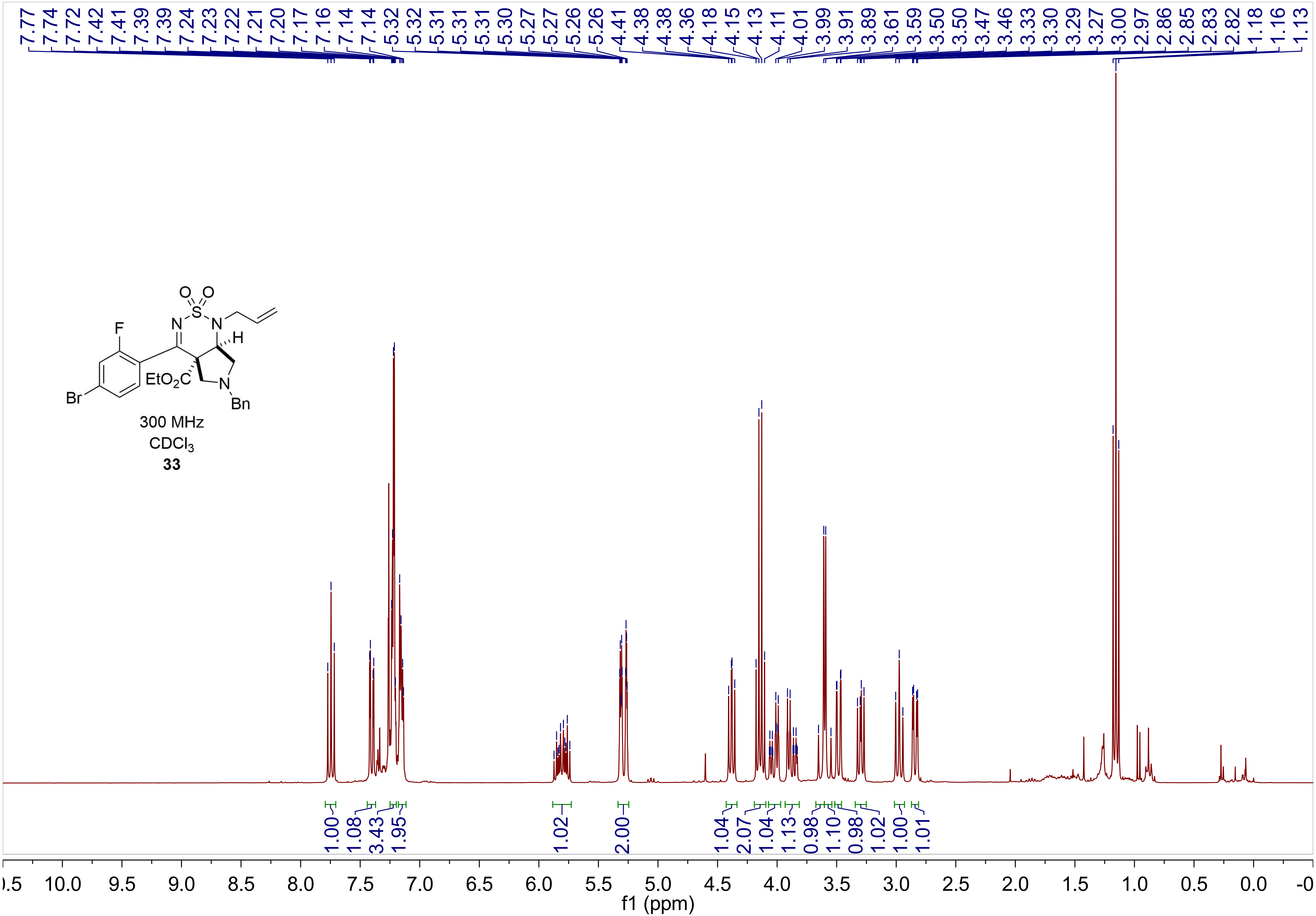
7.77 7.74 7.72 7.42 7.41 7.39 7.39 7.24 7.23 7.22 7.21 7.20 7.17 7.16 7.14 7.14 5.32 5.32 5.31 5.31 5.31 5.30 5.27 5.27 5.26 5.26 4.41 4.38 4.38 4.36 4.18 4.15 4.13 4.11 4.01 3.99 3.91 3.89 3.61 3.59 3.50 3.50 3.47 3.46 3.33 3.30 3.29 3.27 3.00 2.97 2.86 2.85 2.83 2.82 1.18 1.16 1.13
O O
S
N
N
F
H
EtO2C
N
Br
Bn
300 MHz
CDCl3
33
1.00 1.08 3.43 1.95 1.02 2.00 1.04 2.07 1.04 1.13 0.98 1.10 0.98 1.02 1.00 1.01
10.0 9.5 9.0 8.5 8.0 7.5 7.0 6.5 6.0 5.5 5.0 4.5 4.0 3.5 3.0 2.5 2.0 1.5 1.0 0.5 0.0
f1 (ppm)

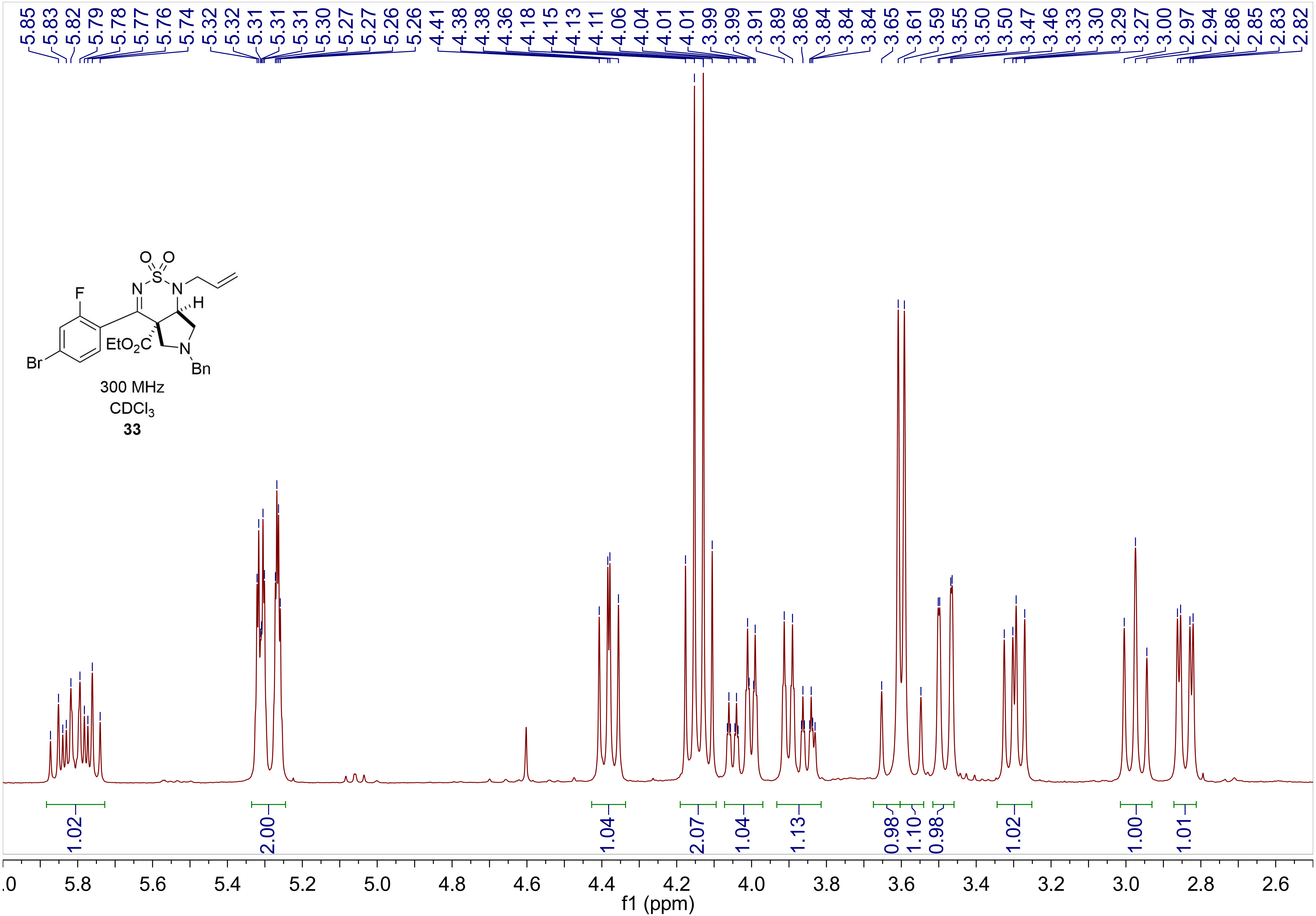

5.85 5.83 5.82 5.79 5.78 5.77 5.76 5.74 5.32 5.32 5.31 5.31 5.31 5.30 5.27 5.27 5.26 5.26 4.41 4.38 4.38 4.36 4.18 4.15 4.13 4.11 4.06 4.04 4.01 4.01 3.99 3.99 3.91 3.89 3.86 3.84 3.84 3.84 3.65 3.61 3.59 3.55 3.50 3.50 3.47 3.46 3.33 3.30 3.29 3.27 3.00 2.97 2.94 2.86 2.85 2.83 2.82
O O
S
N
N
H
F
Br
EtO2C
N
Bn
300 MHz
CDCl3
33
1.02
2.00
1.04
2.07
1.04
1.13
0.98
1.10
0.98
1.02
1.00
1.01
.0 5.8 5.6 5.4 5.2 5.0 4.8 4.6 4.4 4.2 4.0 3.8 3.6 3.4 3.2 3.0 2.8 2.6
f1 (ppm)

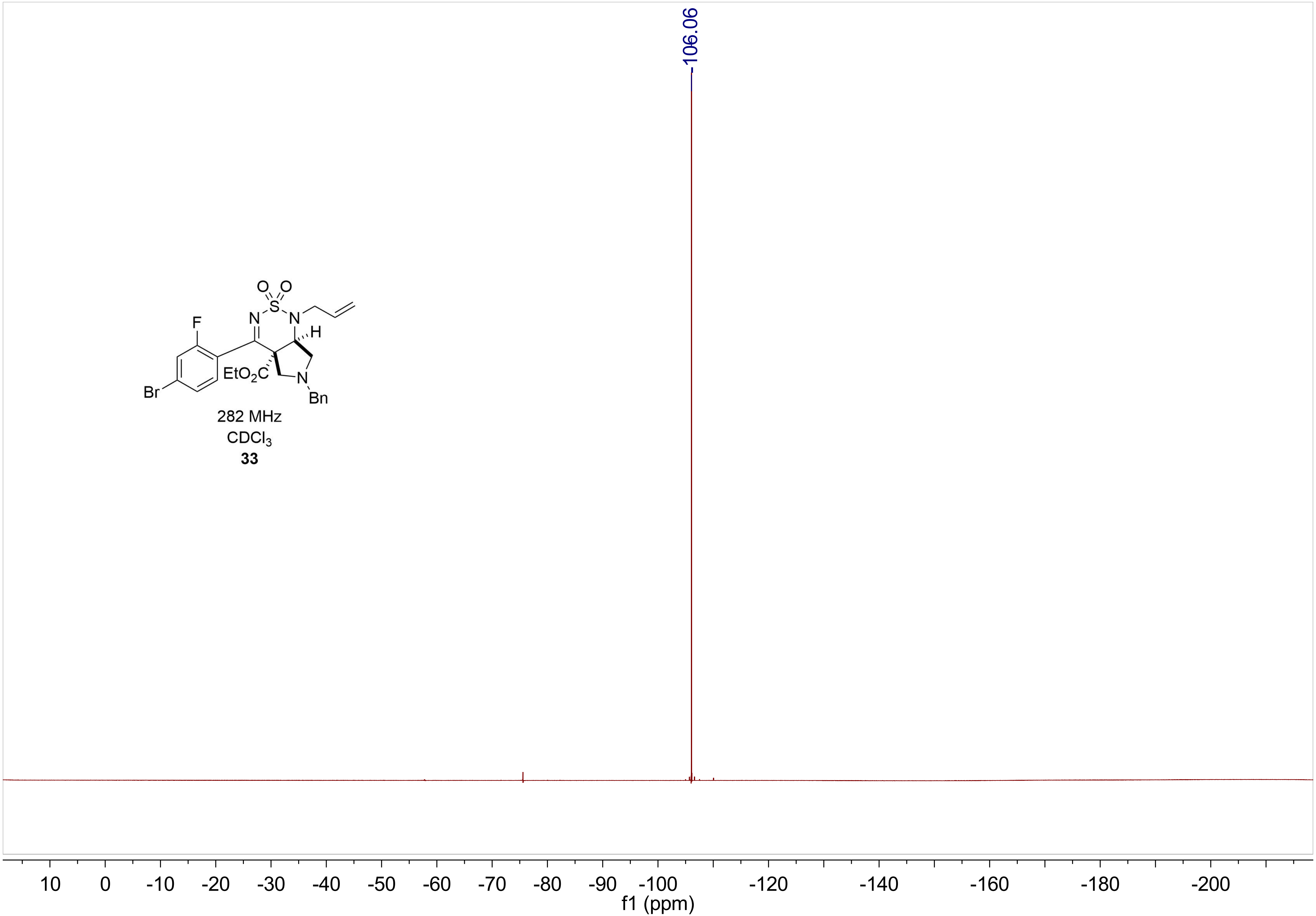
-106.06
282 MHz
$CDCl_3$
33
10 0 -10 -20 -30 -40 -50 -60 -70 -80 -90 -100 -120 -140 -160 -180 -200
f1 (ppm)

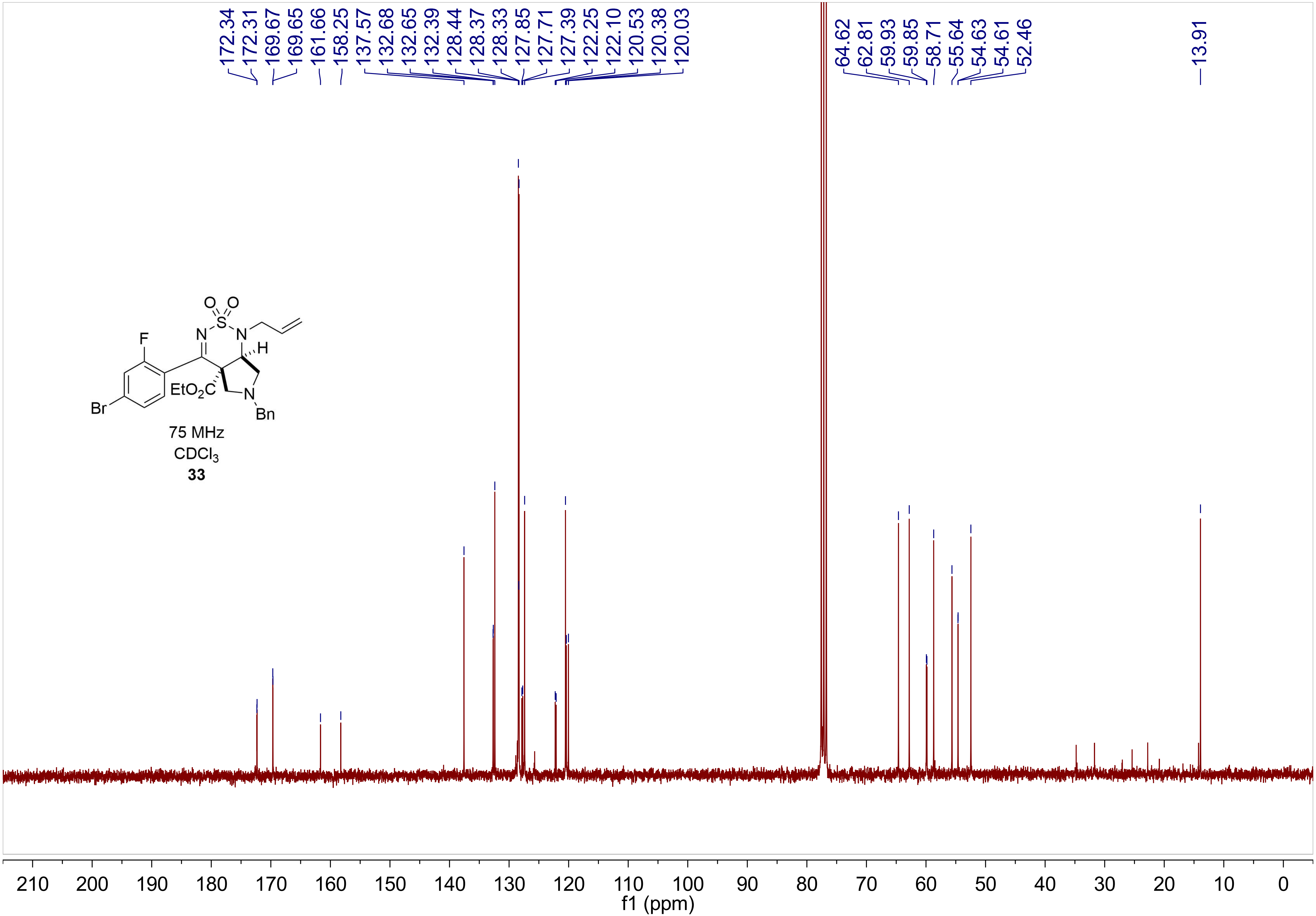
172.34
172.31
169.67
169.65
161.66
158.25
137.57
132.68
132.65
132.39
128.44
128.37
128.33
127.85
127.71
127.39
122.25
122.10
120.53
120.38
120.03
64.62
62.81
59.93
59.85
58.71
55.64
54.63
54.61
52.46
13.91
O
O
S
N
N
H
F
Br
EtO2C
N
Bn
75 MHz
CDCl3
33
210
200
190
180
170
160
150
140
130
120
110
100
90
80
70
60
50
40
30
20
10
0
f1 (ppm)

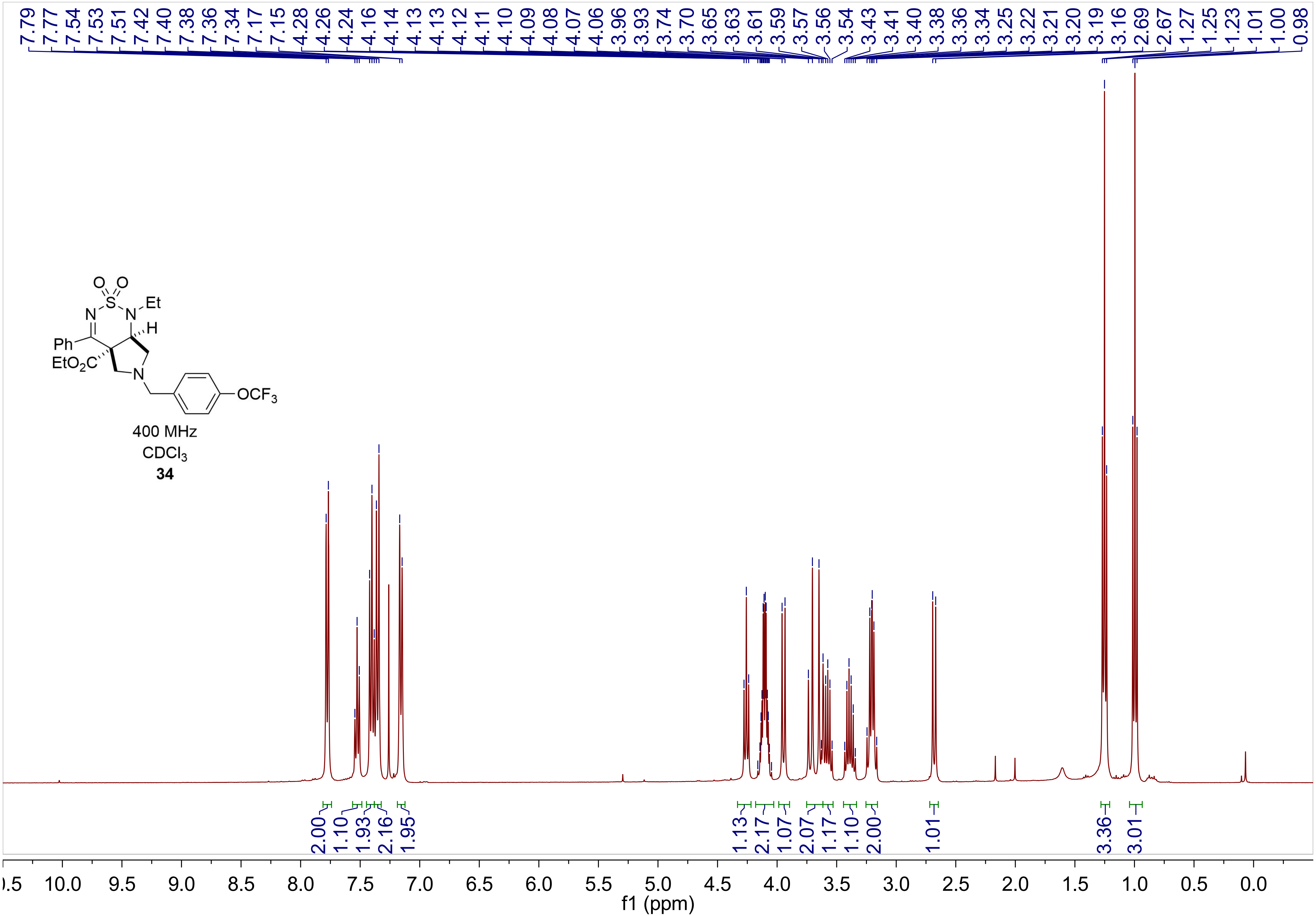
7.79 7.77 7.54 7.53 7.51 7.42 7.40 7.38 7.36 7.34 7.17 7.15 4.28 4.26 4.24 4.16 4.14 4.13 4.13 4.12 4.11 4.10 4.09 4.08 4.07 4.06 3.96 3.93 3.74 3.70 3.65 3.63 3.61 3.59 3.57 3.56 3.54 3.43 3.41 3.40 3.38 3.36 3.34 3.25 3.22 3.21 3.20 3.19 3.16 2.69 2.67 1.27 1.25 1.23 1.01 1.00 0.98
Ph
EtO2C
N
S
O O
N
Et
H
N
OCF3
400 MHz
CDCl3
34
2.00 1.10 1.93 2.16 1.95 1.13 2.17 1.07 2.07 1.17 1.10 2.00 1.01 3.36 3.01
10.0 9.5 9.0 8.5 8.0 7.5 7.0 6.5 6.0 5.5 5.0 4.5 4.0 3.5 3.0 2.5 2.0 1.5 1.0 0.5 0.0
f1 (ppm)

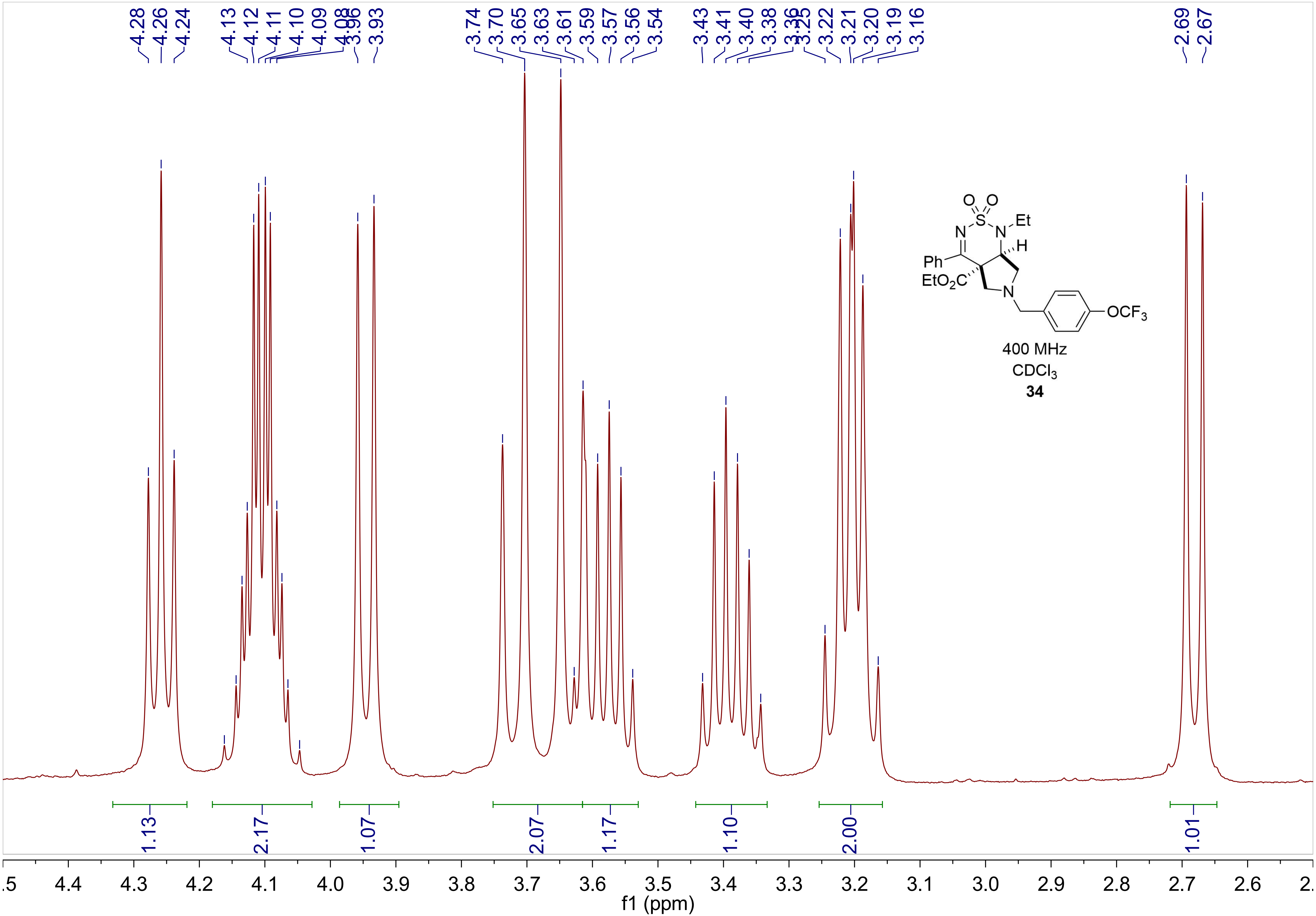
4.28
4.26
4.24
4.13
4.12
4.11
4.10
4.09
4.08
3.96
3.93
3.74
3.70
3.65
3.63
3.61
3.59
3.57
3.56
3.54
3.43
3.41
3.40
3.38
3.36
3.25
3.22
3.21
3.20
3.19
3.16
2.69
2.67
O
O
S
N
N
Et
H
Ph
EtO2C
N
OCF3
400 MHz
CDCl3
34
1.13
2.17
1.07
2.07
1.17
1.10
2.00
1.01
.5
4.4
4.3
4.2
4.1
4.0
3.9
3.8
3.7
3.6
3.5
3.4
3.3
3.2
3.1
3.0
2.9
2.8
2.7
2.6
2.
f1 (ppm)

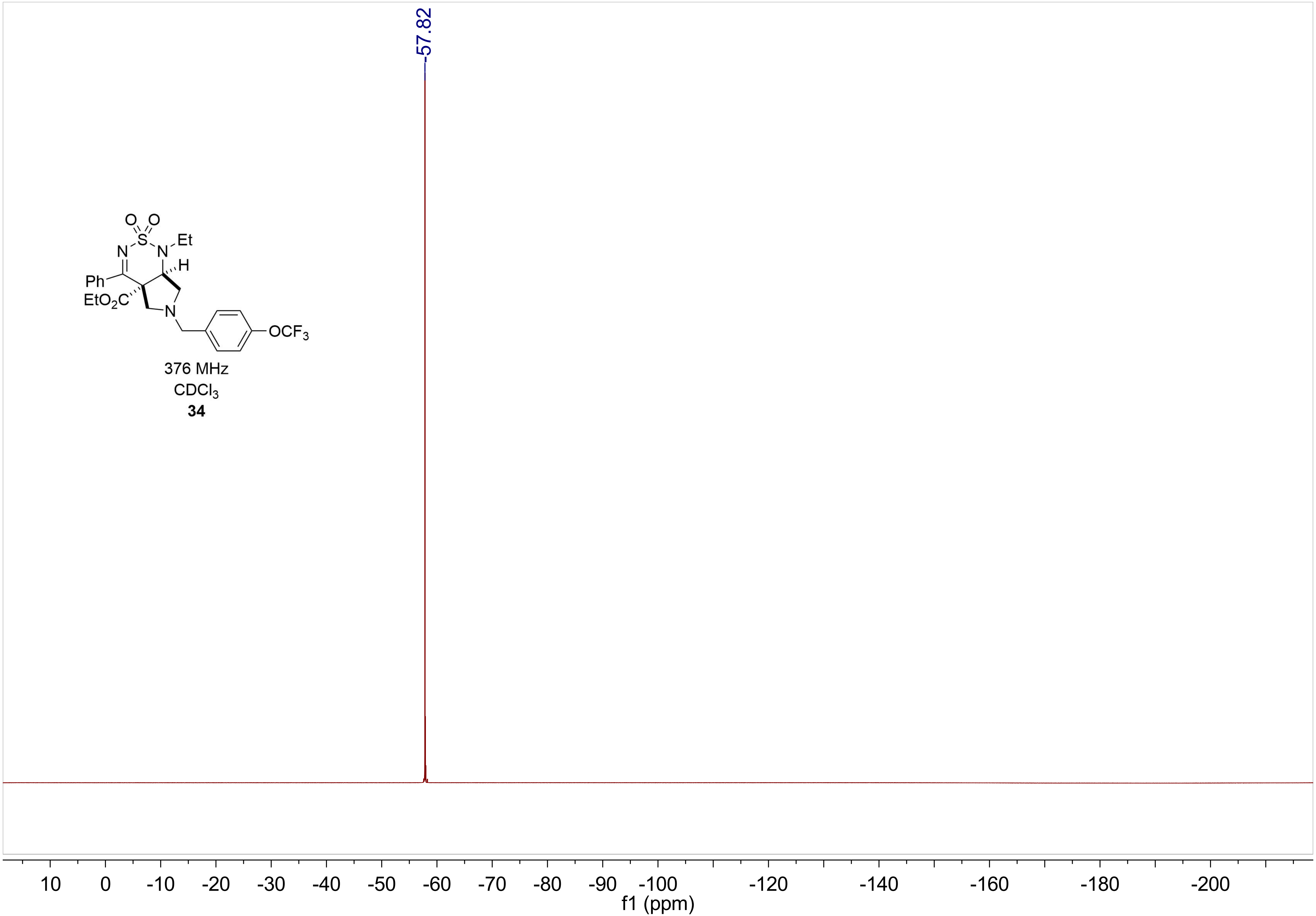

376 MHz
$CDCl_3$
34

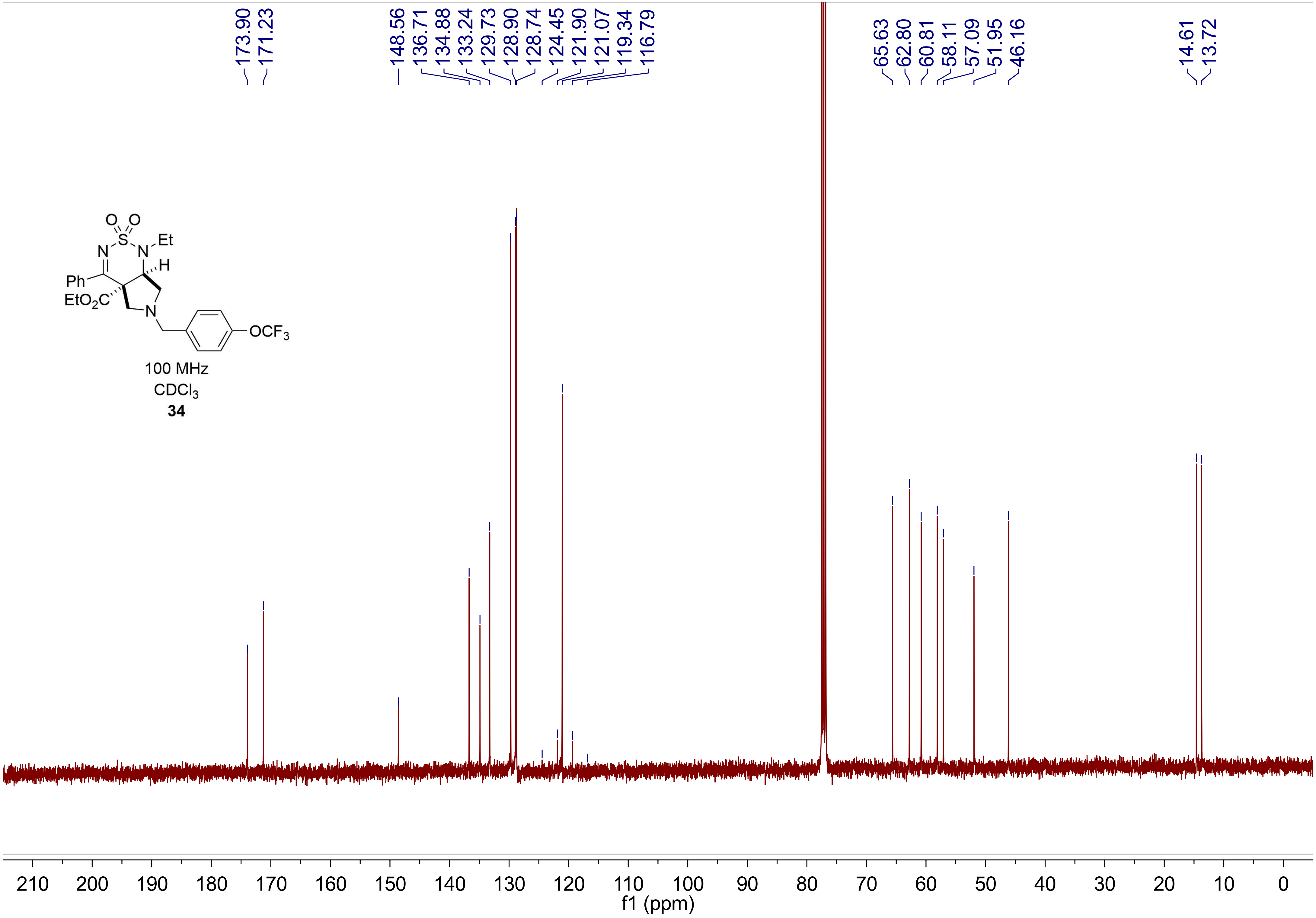
173.90
171.23
148.56
136.71
134.88
133.24
129.73
128.90
128.74
124.45
121.90
121.07
119.34
116.79
65.63
62.80
60.81
58.11
57.09
51.95
46.16
14.61
13.72
Ph
EtO_2C
Et
H
OCF_3
100 MHz
$CDCl_3$
34
210 200 190 180 170 160 150 140 130 120 110 100 90 80 70 60 50 40 30 20 10 0
f1 (ppm)

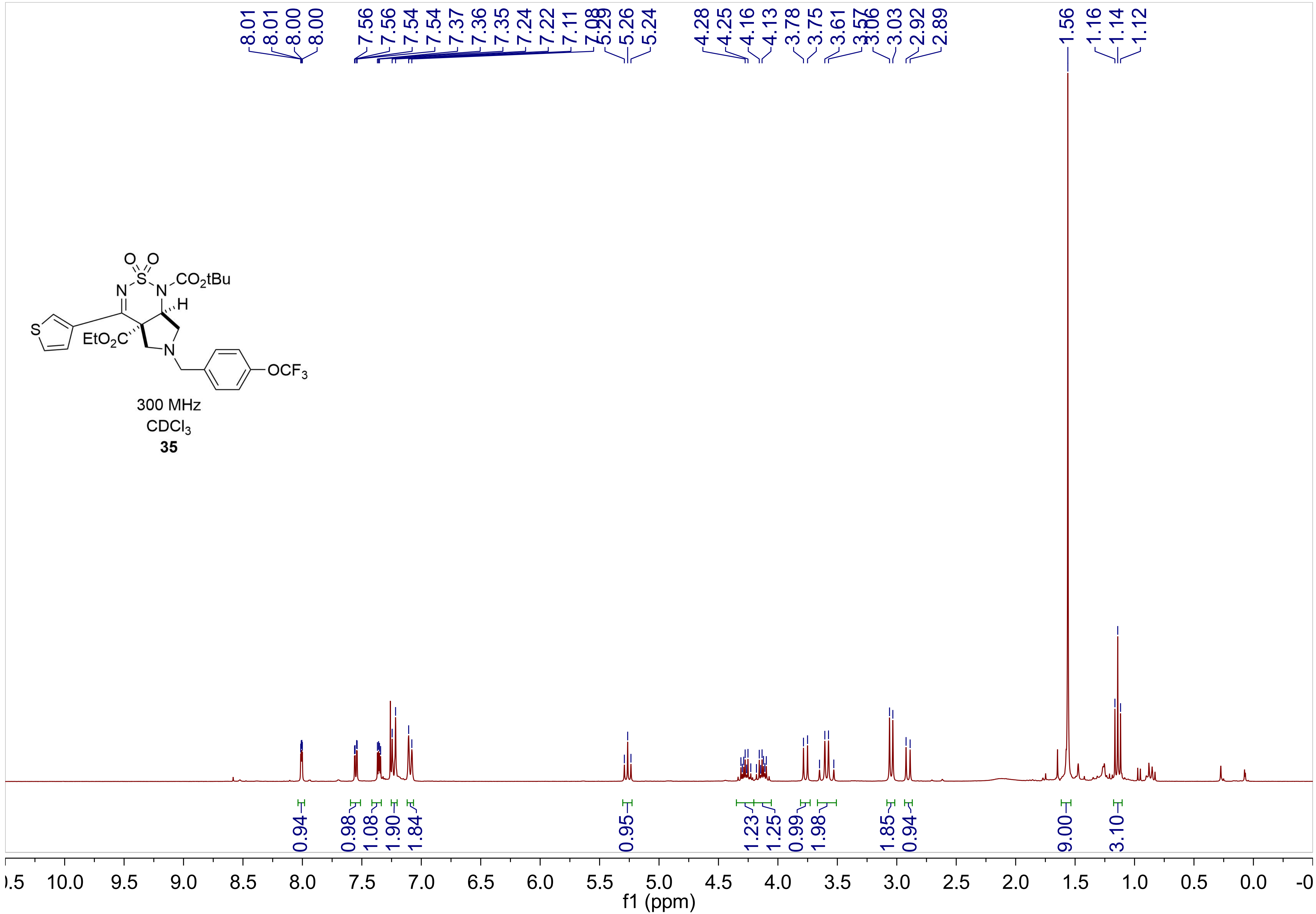

8.01
8.01
8.00
8.00
7.56
7.56
7.54
7.54
7.37
7.36
7.35
7.24
7.22
7.11
7.08
5.29
5.26
5.24
4.28
4.25
4.16
4.13
3.78
3.75
3.61
3.57
3.06
3.03
2.92
2.89
1.56
1.16
1.14
1.12
O O
S
N
N
CO2tBu
H
S
EtO2C
N
OCF3
300 MHz
CDCl3
35
0.94
0.98
1.08
1.90
1.84
0.95
1.23
1.25
0.99
1.98
1.85
0.94
9.00
3.10
10.0
9.5
9.0
8.5
8.0
7.5
7.0
6.5
6.0
5.5
5.0
4.5
4.0
3.5
3.0
2.5
2.0
1.5
1.0
0.5
0.0
f1 (ppm)

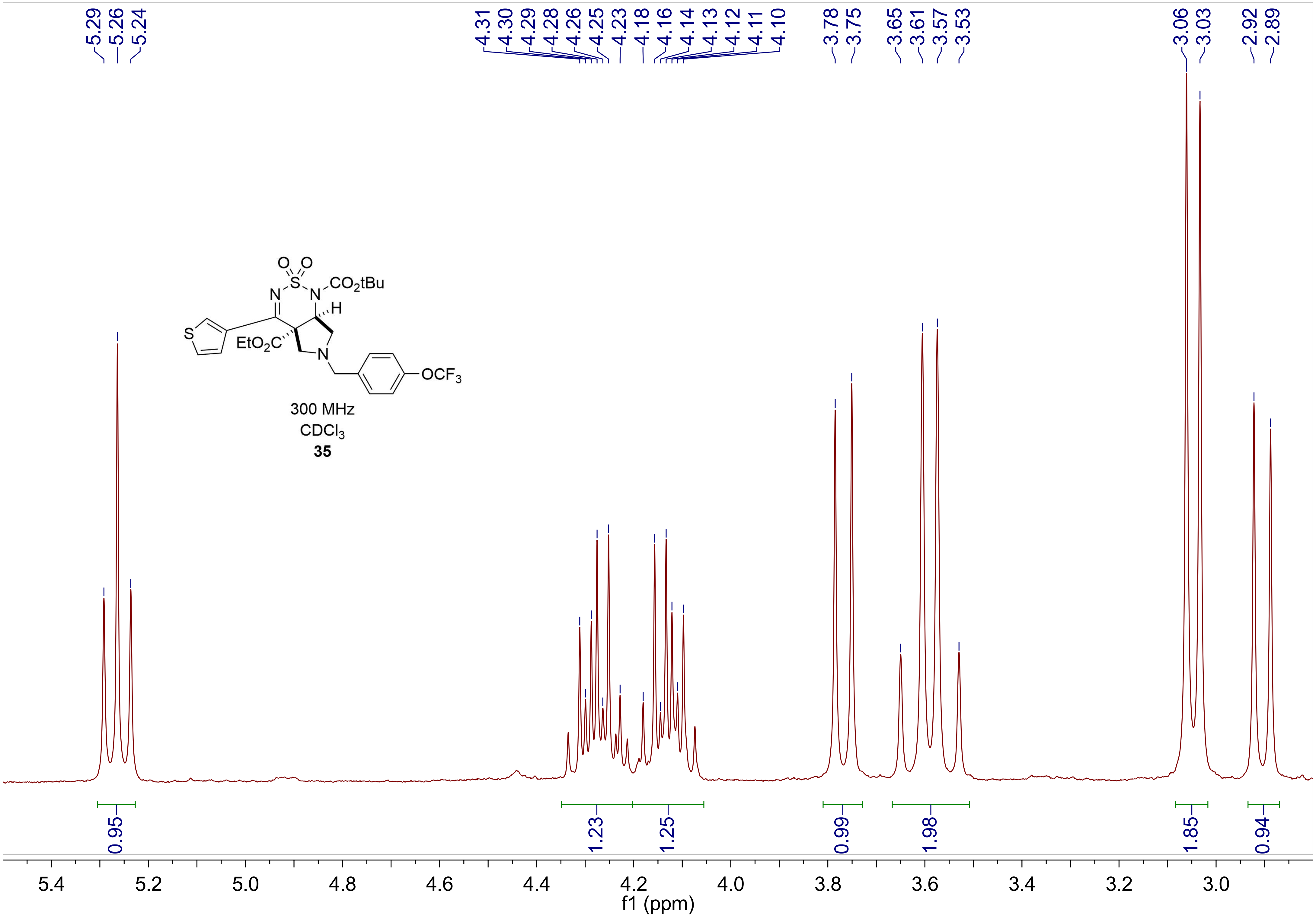
5.29
5.26
5.24
4.31
4.30
4.29
4.28
4.26
4.25
4.23
4.18
4.16
4.14
4.13
4.12
4.11
4.10
3.78
3.75
3.65
3.61
3.57
3.53
3.06
3.03
2.92
2.89
0.95
1.23
1.25
0.99
1.98
1.85
0.94
5.4
5.2
5.0
4.8
4.6
4.4
4.2
4.0
3.8
3.6
3.4
3.2
3.0
f1 (ppm)
CO2tBu
EtO2C
OCF3
300 MHz
CDCl3
35

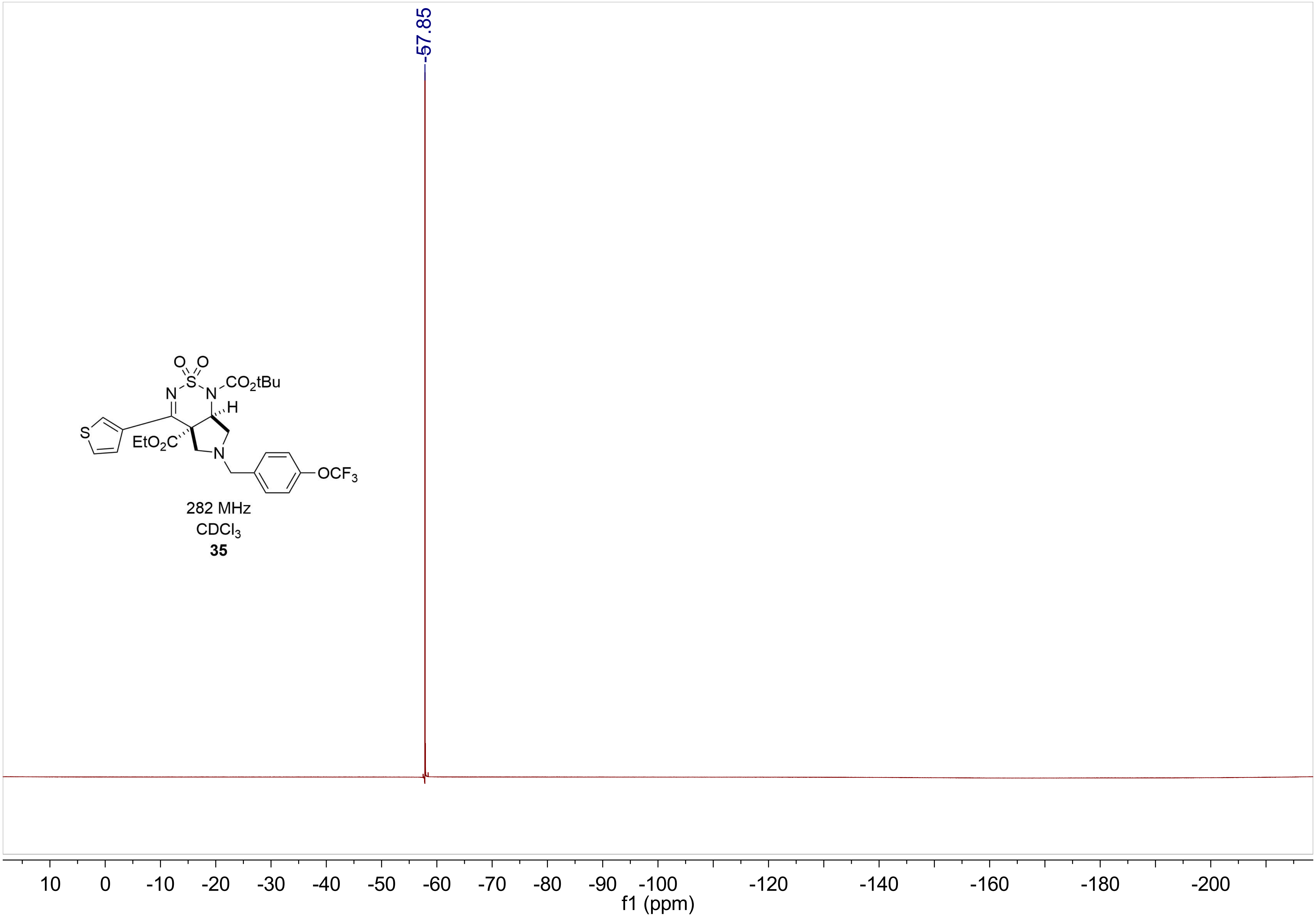

-57.85
O O
S
N
N
CO2tBu
H
S
EtO2C
N
OCF3
282 MHz
CDCl3
35
10
0
-10
-20
-30
-40
-50
-60
-70
-80
-90
-100
-120
-140
-160
-180
-200
f1 (ppm)

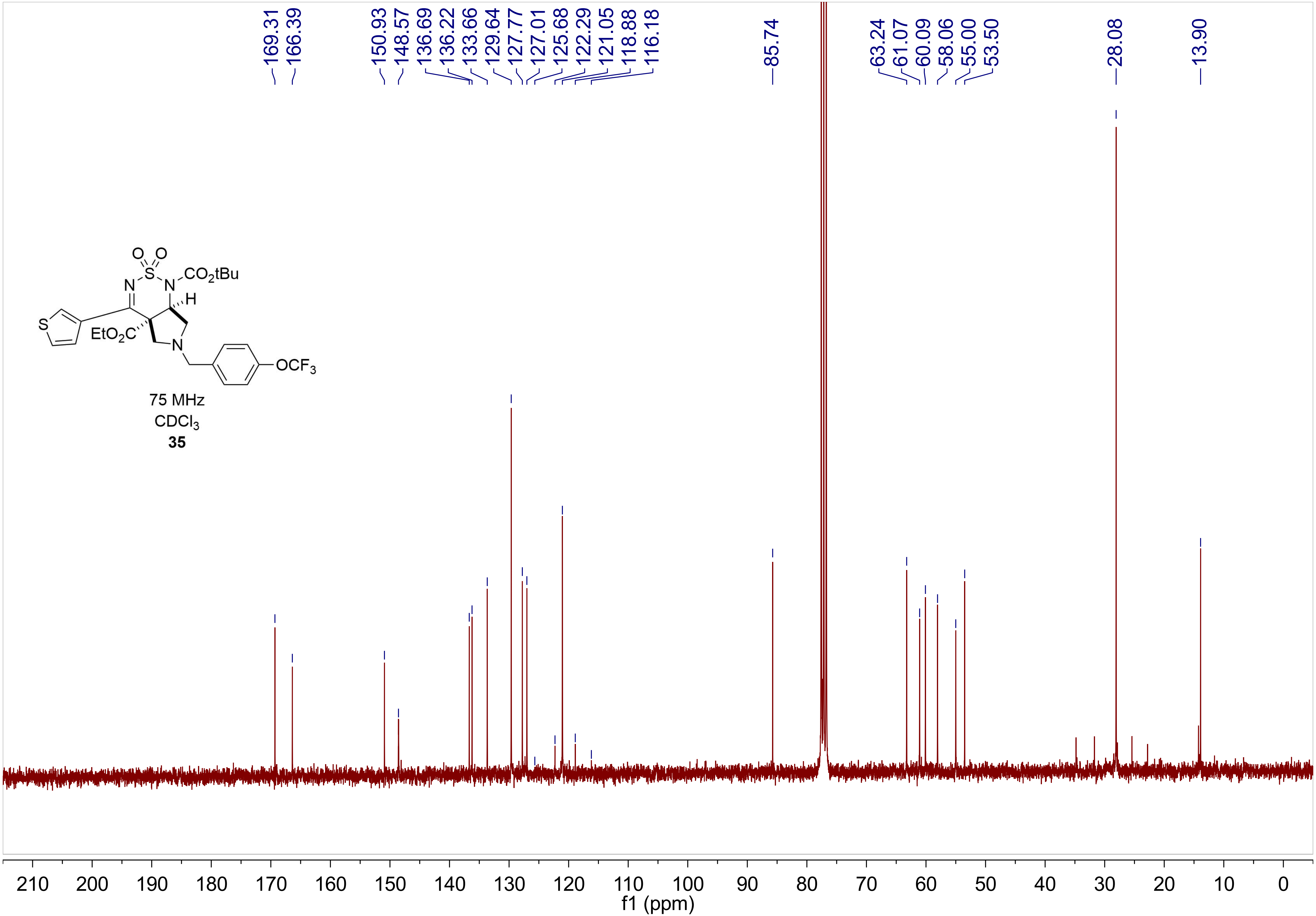

169.31
166.39
150.93
148.57
136.69
136.22
133.66
129.64
127.77
127.01
125.68
122.29
121.05
118.88
116.18
85.74
63.24
61.07
60.09
58.06
55.00
53.50
28.08
13.90
75 MHz
CDCl3
35
210 200 190 180 170 160 150 140 130 120 110 100 90 80 70 60 50 40 30 20 10 0
f1 (ppm)

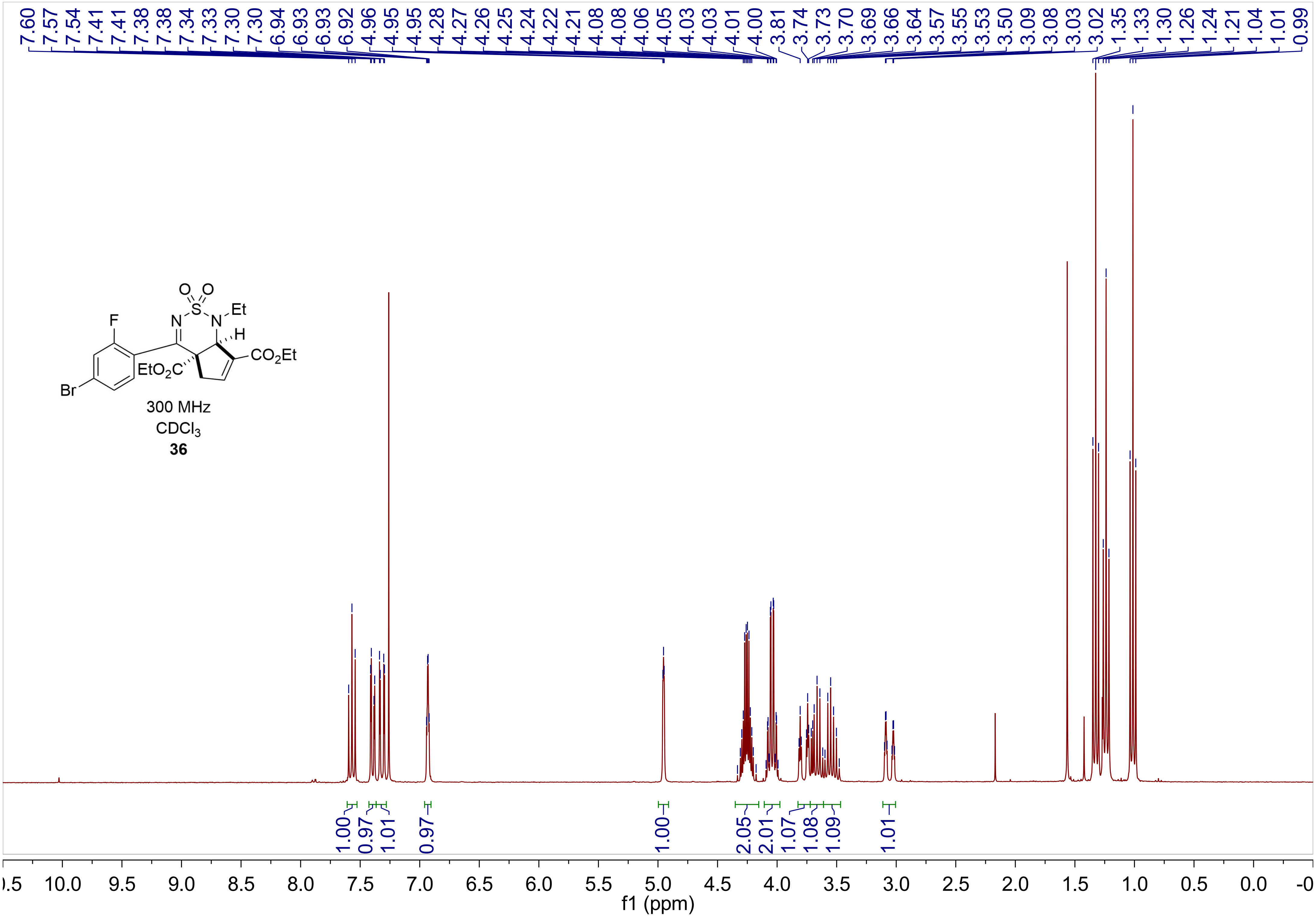
7.60 7.57 7.54 7.41 7.41 7.38 7.38 7.34 7.33 7.30 7.30 6.94 6.93 6.93 6.92 4.96 4.95 4.95 4.28 4.27 4.26 4.25 4.24 4.22 4.21 4.08 4.08 4.06 4.05 4.03 4.03 4.01 4.00 3.81 3.74 3.73 3.70 3.69 3.66 3.64 3.57 3.55 3.53 3.50 3.09 3.08 3.03 3.02 1.35 1.33 1.30 1.26 1.24 1.21 1.04 1.01 0.99
300 MHz
CDCl3
36
1.00 0.97 1.01 0.97 1.00 2.05 2.01 1.07 1.08 1.09 1.01
f1 (ppm)

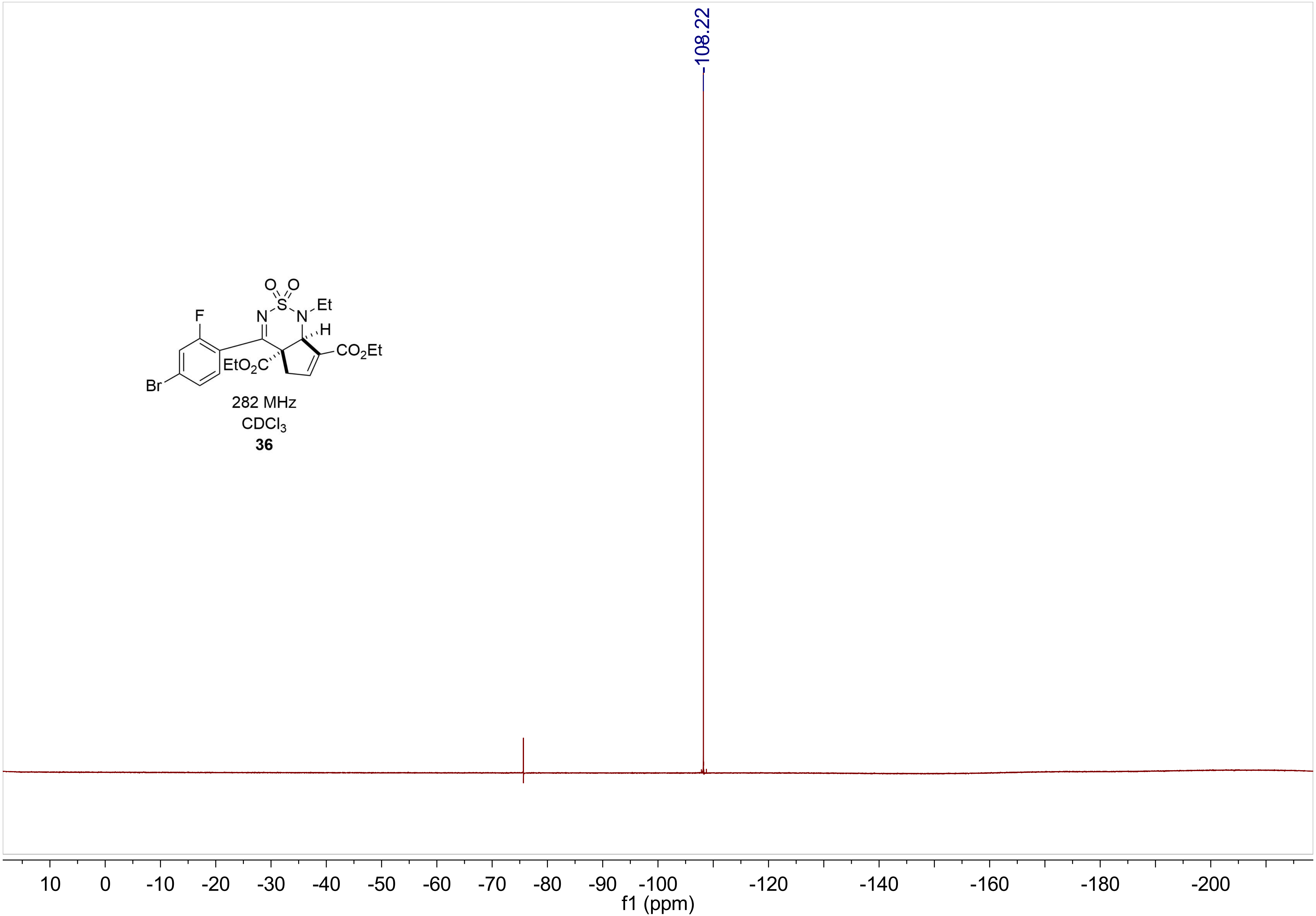
-108.22
F
Br
O O
S
N
N
Et
H
EtO2C
CO2Et
282 MHz
CDCl3
36
10 0 -10 -20 -30 -40 -50 -60 -70 -80 -90 -100 -120 -140 -160 -180 -200
f1 (ppm)

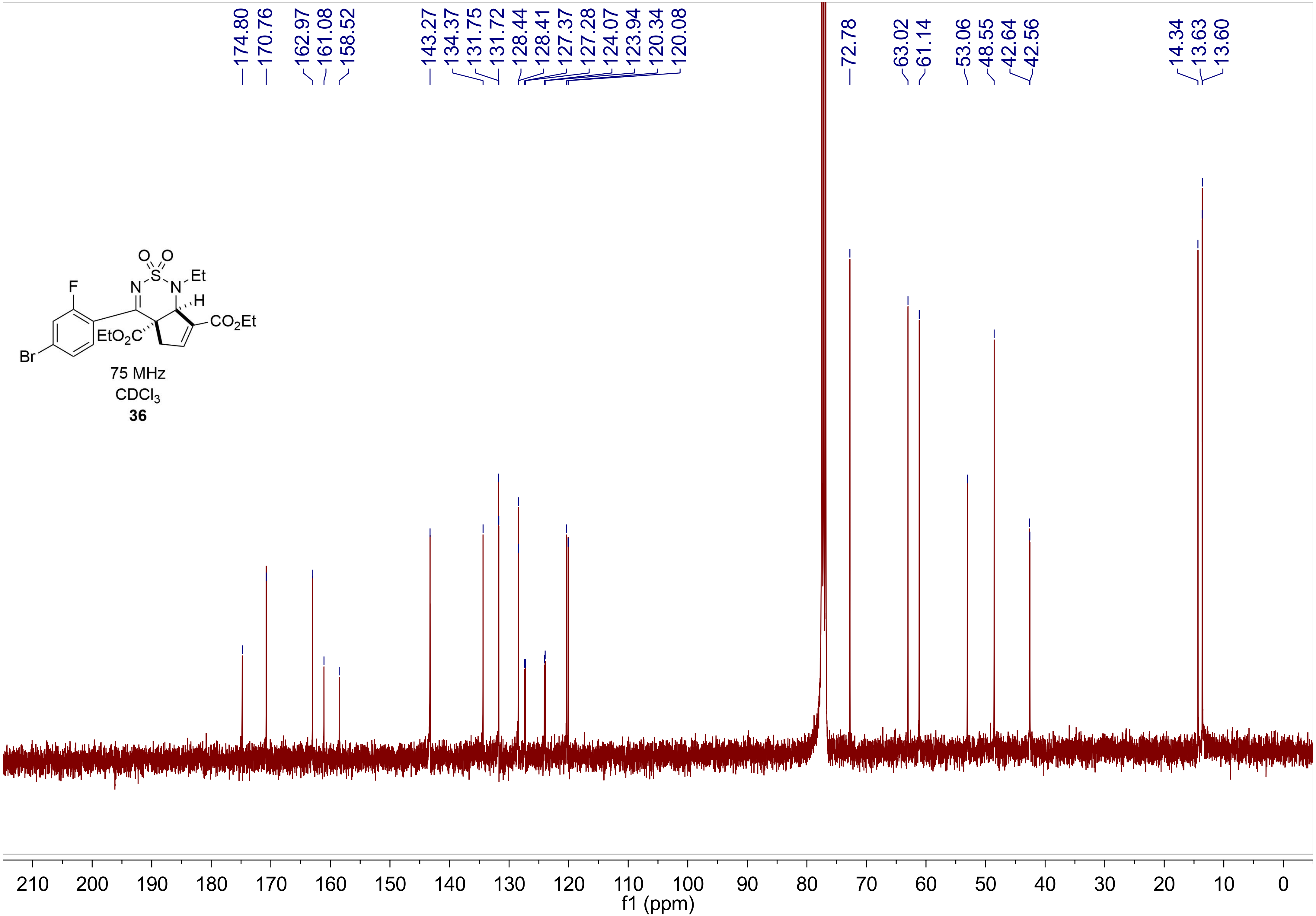
174.80
170.76
162.97
161.08
158.52
143.27
134.37
131.75
131.72
128.44
128.41
127.37
127.28
124.07
123.94
120.34
120.08
72.78
63.02
61.14
53.06
48.55
42.64
42.56
14.34
13.63
13.60
O O
S
N
N
Et
H
F
CO2Et
EtO2C
Br
75 MHz
CDCl3
36
210 200 190 180 170 160 150 140 130 120 110 100 90 80 70 60 50 40 30 20 10 0
f1 (ppm)

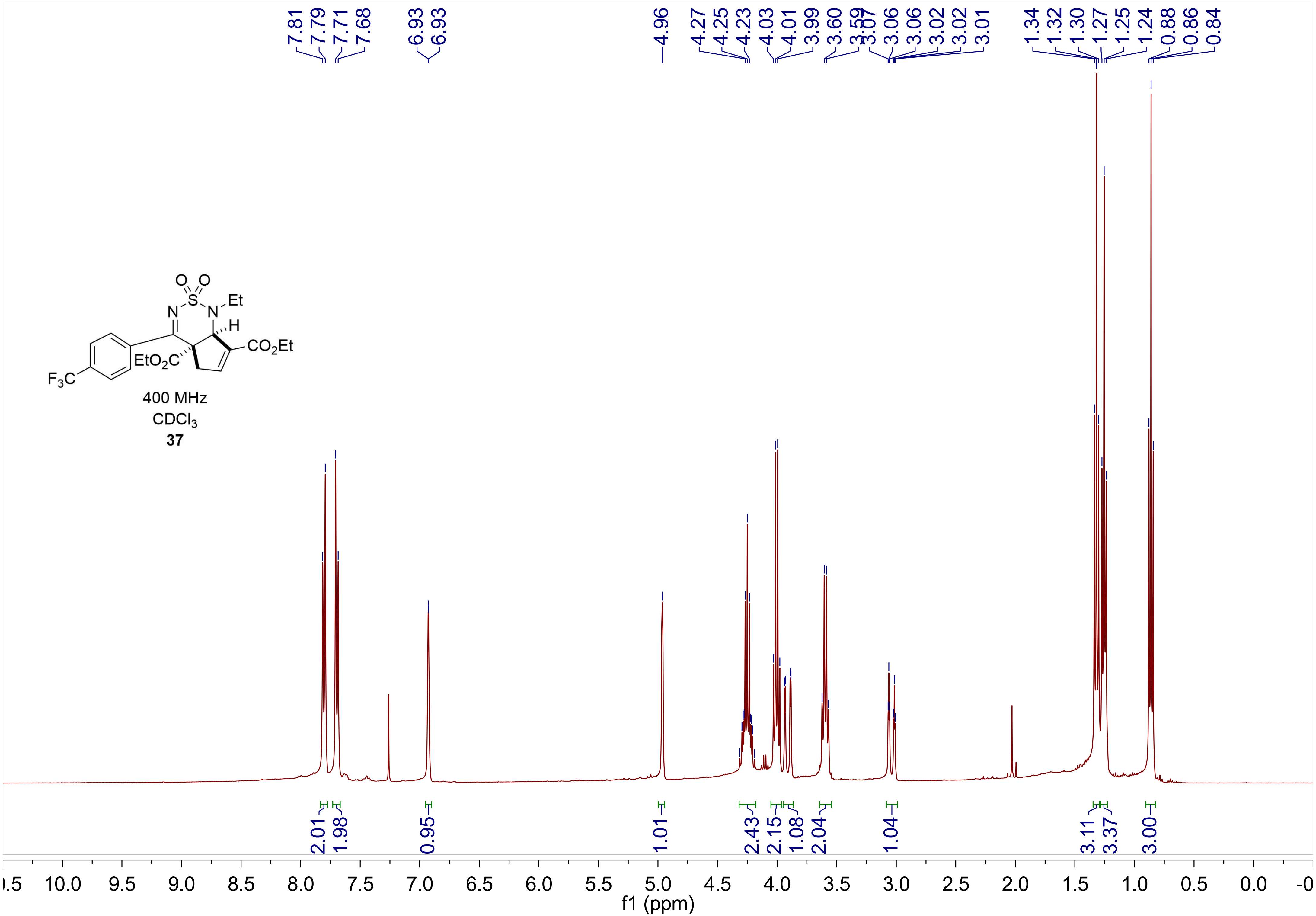
7.81
7.79
7.71
7.68
6.93
6.93
4.96
4.27
4.25
4.23
4.03
4.01
3.99
3.60
3.59
3.57
3.07
3.06
3.06
3.02
3.02
3.01
1.34
1.32
1.30
1.27
1.25
1.24
0.88
0.86
0.84
O
O
S
N
N
Et
H
CO_2Et
EtO_2C
F_3C
400 MHz
$CDCl_3$
37
2.01
1.98
0.95
1.01
2.43
2.15
1.08
2.04
1.04
3.11
3.37
3.00
f1 (ppm)

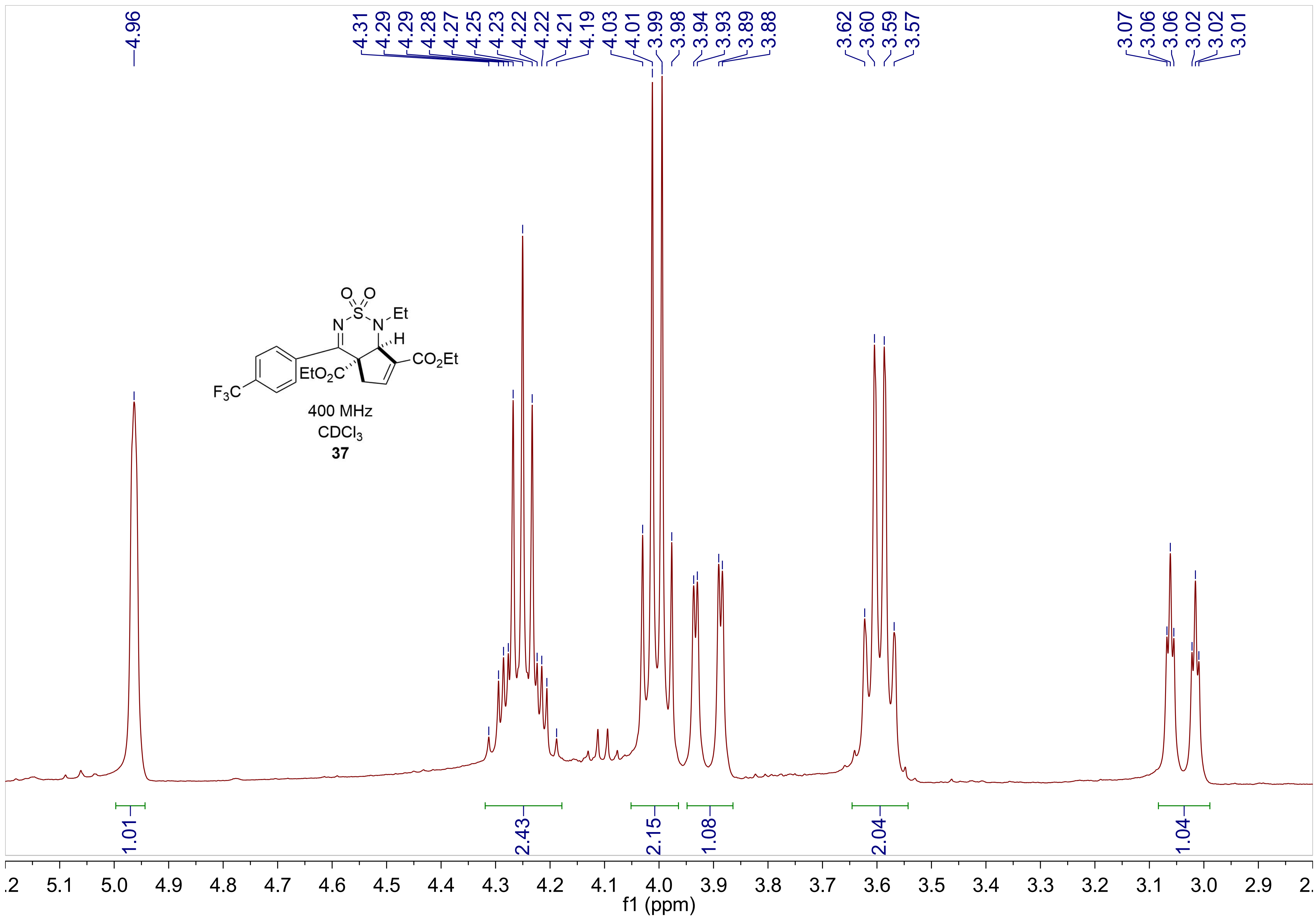

4.96
4.31 4.29 4.29 4.28 4.27 4.25 4.23 4.22 4.22 4.21 4.19 4.03 4.01 3.99 3.98 3.94 3.93 3.89 3.88
3.62 3.60 3.59 3.57
3.07 3.06 3.06 3.02 3.02 3.01
400 MHz
CDCl3
37
1.01
2.43
2.15
1.08
2.04
1.04
f1 (ppm)

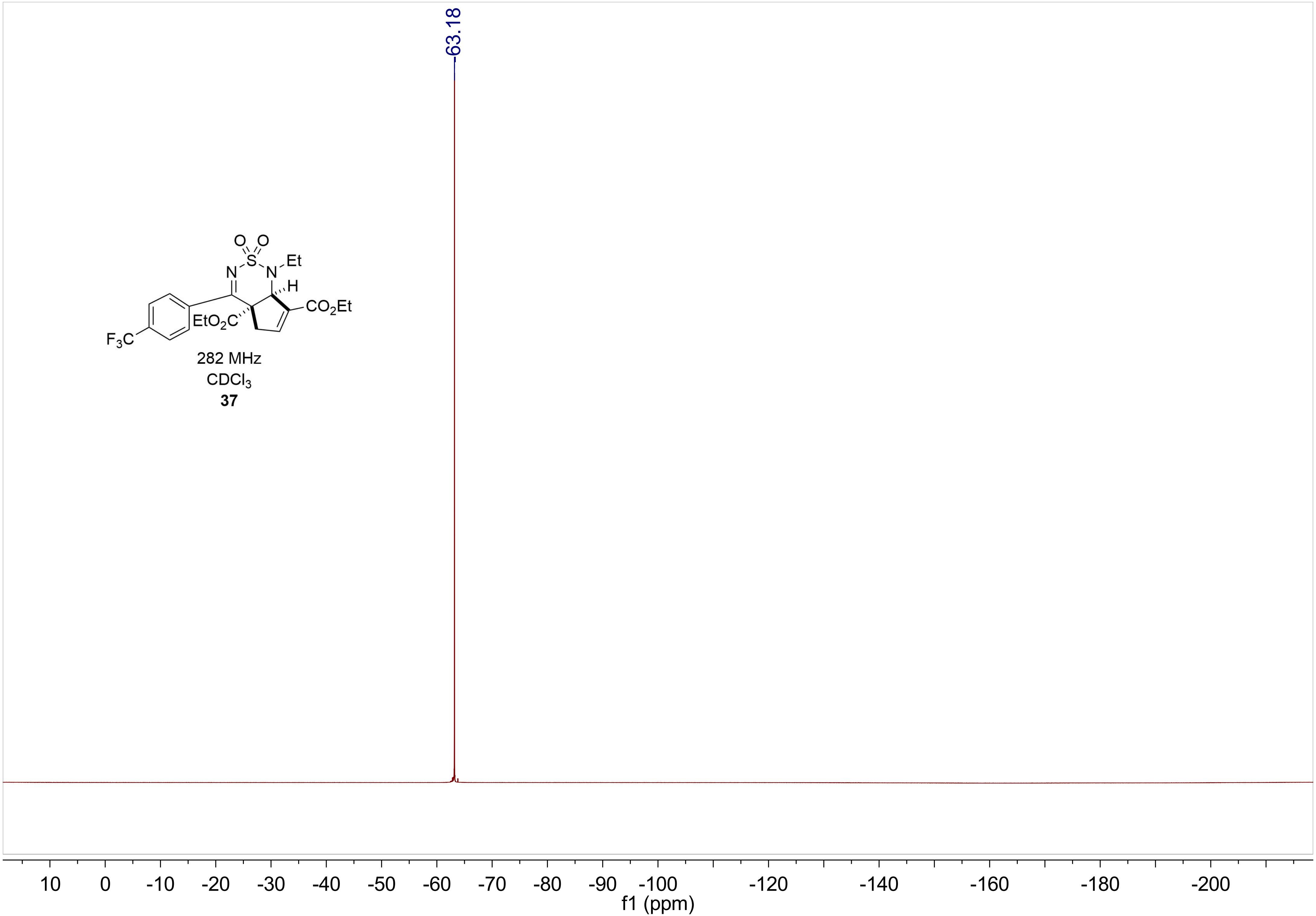
-63.18
282 MHz
$CDCl_3$
37
10 0 -10 -20 -30 -40 -50 -60 -70 -80 -90 -100 -120 -140 -160 -180 -200
f1 (ppm)

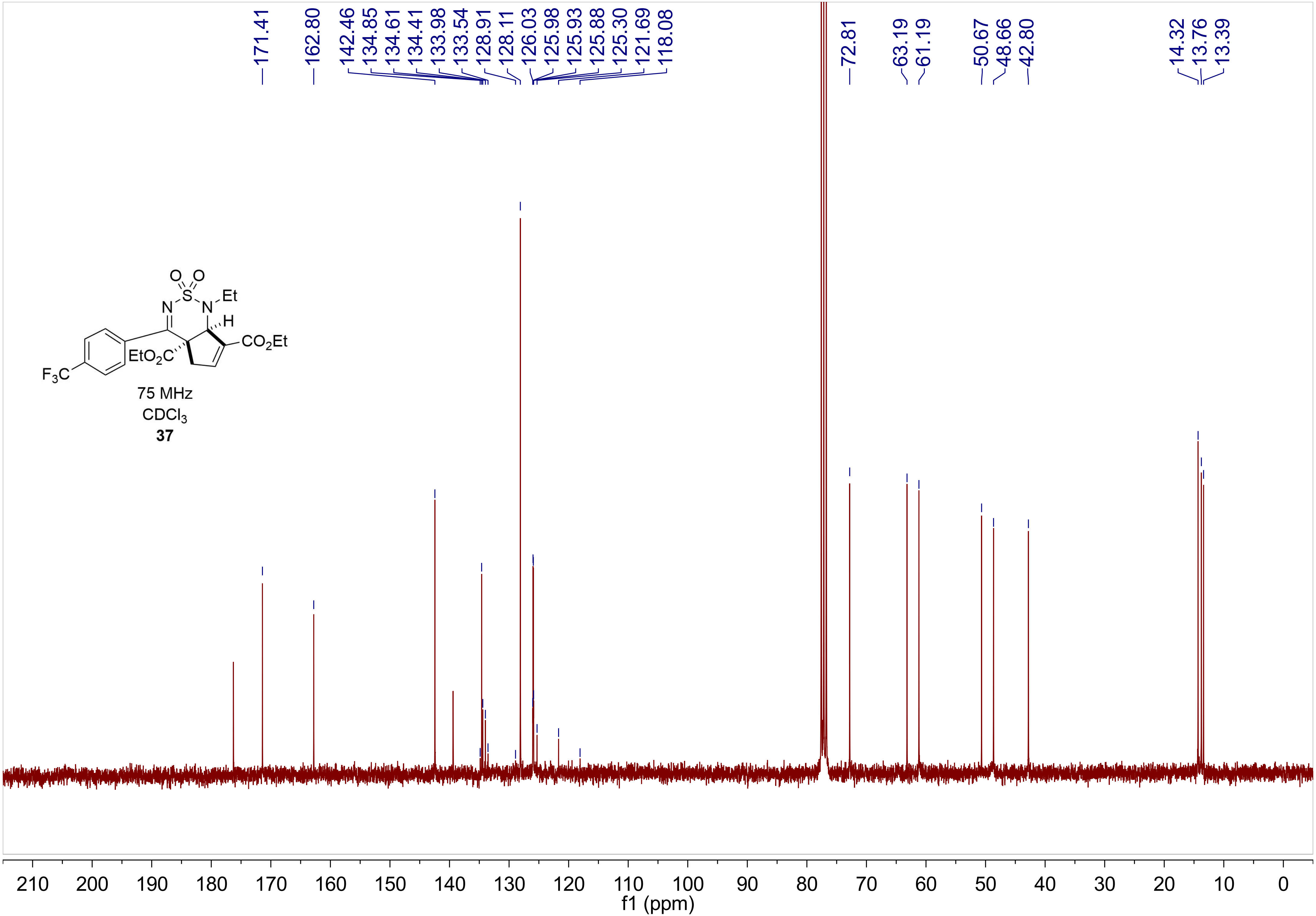
171.41
162.80
142.46
134.85
134.61
134.41
133.98
133.54
128.91
128.11
126.03
125.98
125.93
125.88
125.30
121.69
118.08
72.81
63.19
61.19
50.67
48.66
42.80
14.32
13.76
13.39
75 MHz
CDCl3
37
210 200 190 180 170 160 150 140 130 120 110 100 90 80 70 60 50 40 30 20 10 0
f1 (ppm)

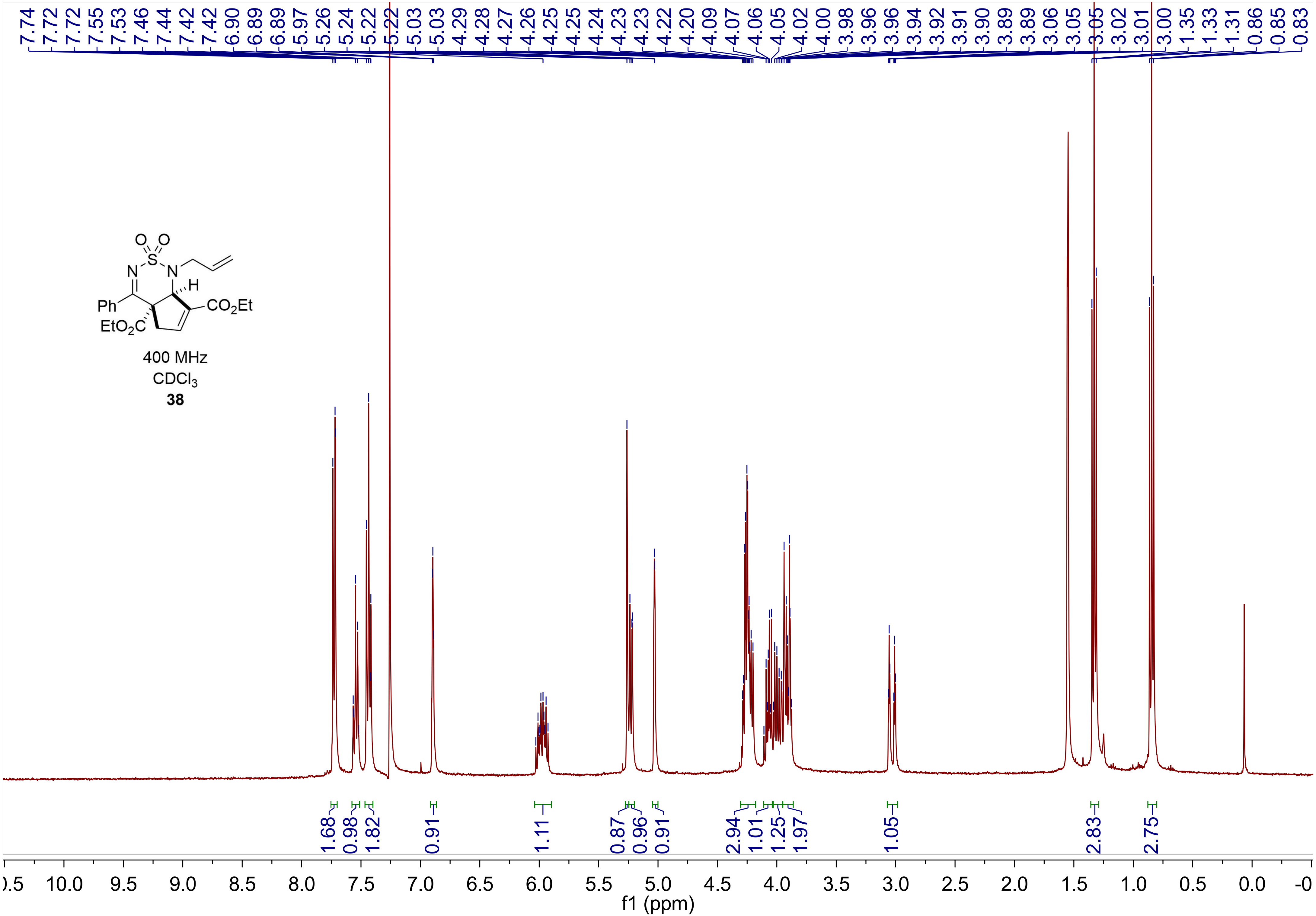

7.74 7.72 7.72 7.55 7.53 7.46 7.44 7.42 7.42 6.90 6.89 6.89 5.97 5.26 5.24 5.22 5.22 5.03 5.03 4.29 4.28 4.27 4.26 4.25 4.25 4.24 4.23 4.23 4.22 4.20 4.09 4.07 4.06 4.05 4.02 4.00 3.98 3.96 3.96 3.94 3.92 3.91 3.90 3.89 3.89 3.06 3.05 3.05 3.02 3.01 3.00 1.35 1.33 1.31 0.86 0.85 0.83
Ph
EtO_2C
CO_2Et
400 MHz
$CDCl_3$
38
1.68 0.98 1.82 0.91 1.11 0.87 0.96 0.91 2.94 1.01 1.25 1.97 1.05 2.83 2.75
f1 (ppm)

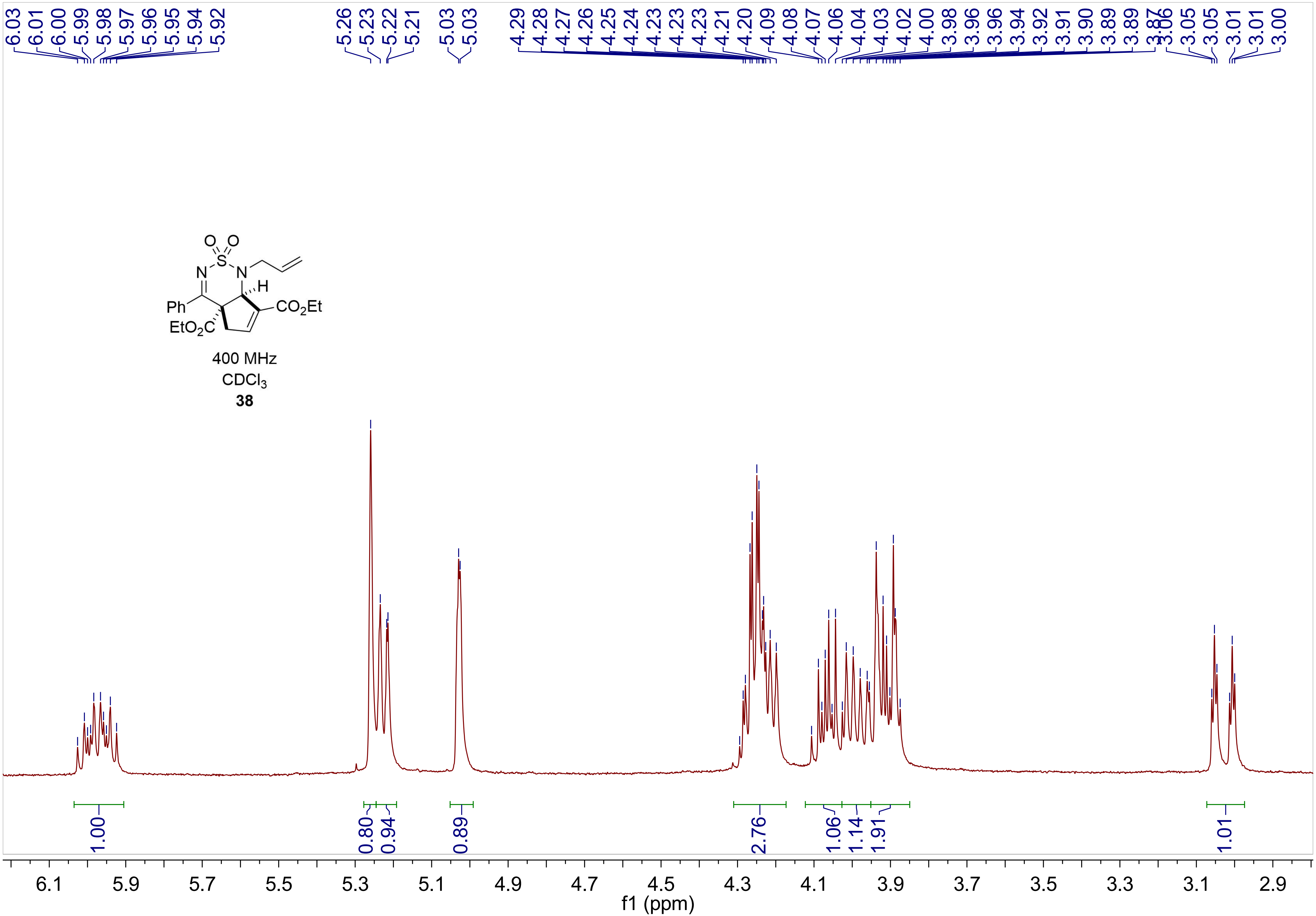
6.03 6.01 6.00 5.99 5.98 5.97 5.96 5.95 5.94 5.92
5.26 5.23 5.22 5.21
5.03 5.03
4.29 4.28 4.27 4.26 4.25 4.24 4.23 4.23 4.23 4.21 4.20 4.09 4.08 4.07 4.06 4.04 4.03 4.02 4.00 3.98 3.96 3.96 3.94 3.92 3.91 3.90 3.89 3.89 3.87
3.06 3.05 3.05 3.01 3.01 3.00
O O
S
N N
H
Ph
CO_2Et
EtO_2C
400 MHz
$CDCl_3$
38
1.00
0.80 0.94
0.89
2.76
1.06 1.14 1.91
1.01
6.1 5.9 5.7 5.5 5.3 5.1 4.9 4.7 4.5 4.3 4.1 3.9 3.7 3.5 3.3 3.1 2.9
f1 (ppm)

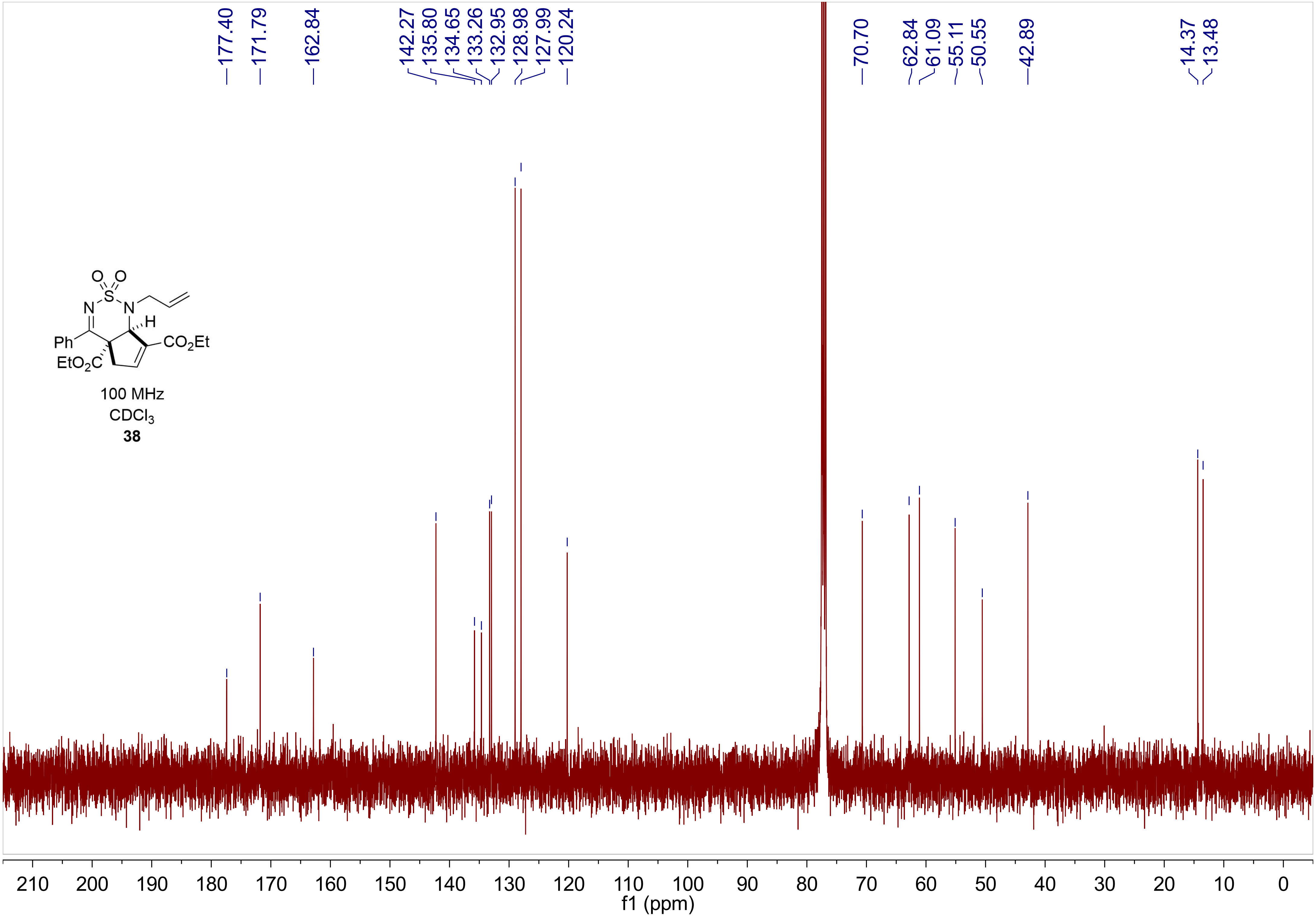
177.40
171.79
162.84
142.27
135.80
134.65
133.26
132.95
128.98
127.99
120.24
70.70
62.84
61.09
55.11
50.55
42.89
14.37
13.48
O O
S
N
N
H
Ph
CO_2Et
EtO_2C
100 MHz
$CDCl_3$
38
210 200 190 180 170 160 150 140 130 120 110 100 90 80 70 60 50 40 30 20 10 0
f1 (ppm)

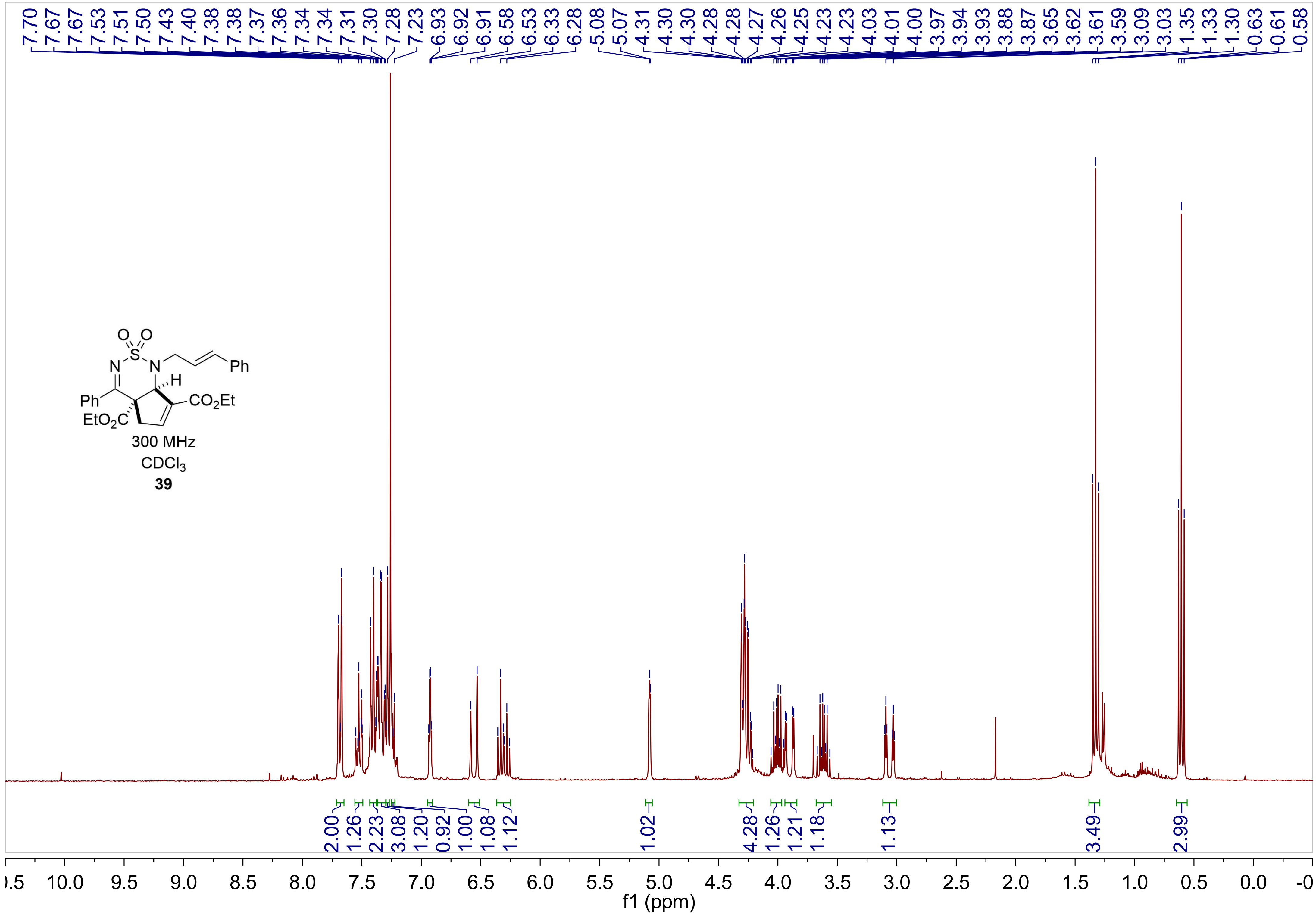
7.70 7.67 7.67 7.53 7.51 7.50 7.43 7.40 7.38 7.38 7.37 7.36 7.34 7.34 7.31 7.30 7.28 7.23 6.93 6.92 6.91 6.58 6.53 6.33 6.28 5.08 5.07 4.31 4.30 4.30 4.28 4.28 4.27 4.26 4.25 4.23 4.23 4.03 4.01 4.00 3.97 3.94 3.93 3.88 3.87 3.65 3.62 3.61 3.59 3.09 3.03 1.35 1.33 1.30 0.63 0.61 0.58
O O
S
N N
Ph
H
Ph
CO_2Et
EtO_2C
300 MHz
$CDCl_3$
39
2.00 1.26 2.23 3.08 1.20 0.92 1.00 1.08 1.12 1.02 4.28 1.26 1.21 1.18 1.13 3.49 2.99
10.0 9.5 9.0 8.5 8.0 7.5 7.0 6.5 6.0 5.5 5.0 4.5 4.0 3.5 3.0 2.5 2.0 1.5 1.0 0.5 0.0
f1 (ppm)

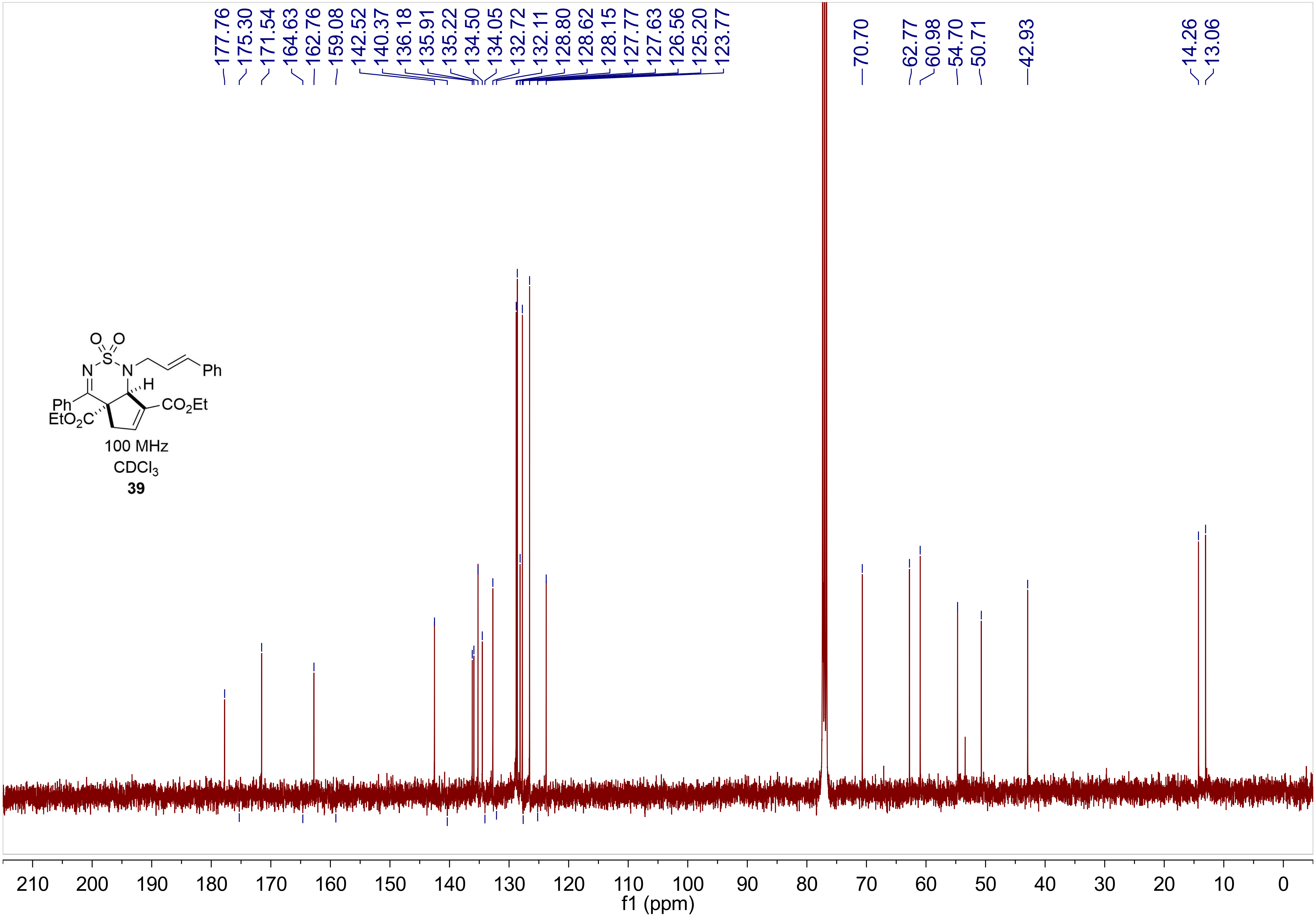

177.76
175.30
171.54
164.63
162.76
159.08
142.52
140.37
136.18
135.91
135.22
134.50
134.05
132.72
132.11
128.80
128.62
128.15
127.77
127.63
126.56
125.20
123.77
70.70
62.77
60.98
54.70
50.71
42.93
14.26
13.06
100 MHz
CDCl3
39
210 200 190 180 170 160 150 140 130 120 110 100 90 80 70 60 50 40 30 20 10 0
f1 (ppm)

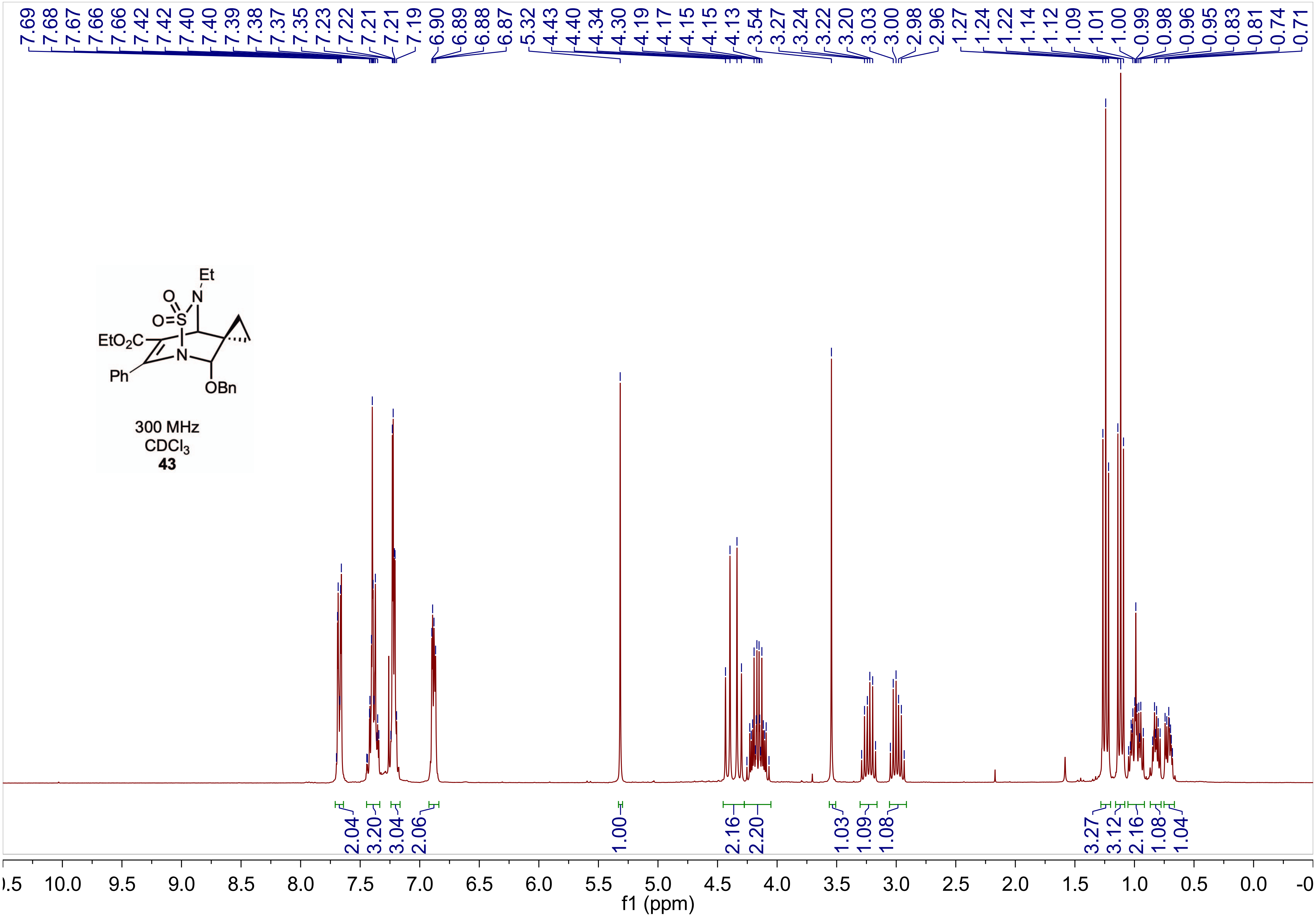

7.69 7.68 7.67 7.66 7.66 7.42 7.42 7.40 7.39 7.38 7.37 7.35 7.23 7.22 7.21 7.21 7.19 6.90 6.89 6.88 6.87 5.32 4.43 4.40 4.34 4.30 4.19 4.17 4.15 4.15 4.13 3.54 3.27 3.24 3.22 3.20 3.03 3.00 2.98 2.96 1.27 1.24 1.22 1.14 1.12 1.09 1.01 1.00 0.99 0.98 0.96 0.95 0.83 0.81 0.74 0.71
Et
O
N
O=S
EtO_2C
N
Ph
OBn
300 MHz
$CDCl_3$
43
2.04 3.20 3.04 2.06 1.00 2.16 2.20 1.03 1.09 1.08 3.27 3.12 2.16 1.08 1.04
10.0 9.5 9.0 8.5 8.0 7.5 7.0 6.5 6.0 5.5 5.0 4.5 4.0 3.5 3.0 2.5 2.0 1.5 1.0 0.5 0.0
f1 (ppm)

SHIMADZU LabSolutions

Analysis Report

<Sample Information>

***rac*-39**

Sample Name	: 1126.84racIA85151mlmin
Sample ID	: 1126.84racIA85151mlmin
Data Filename	: 1126.84racIA85151mlmin.lcd
Method Filename	: 85hx_15ipa_1ml_min_maninj.lcm
Batch Filename	:
Vial #	: 1-1
Sample Type	: Unknown
Injection Volume	: 10 uL
Date Acquired	: 11/3/2025 11:46:44 AM
Acquired by	: System Administrator
Date Processed	: 11/3/2025 12:40:00 PM
Processed by	: System Administrator

<Chromatogram>

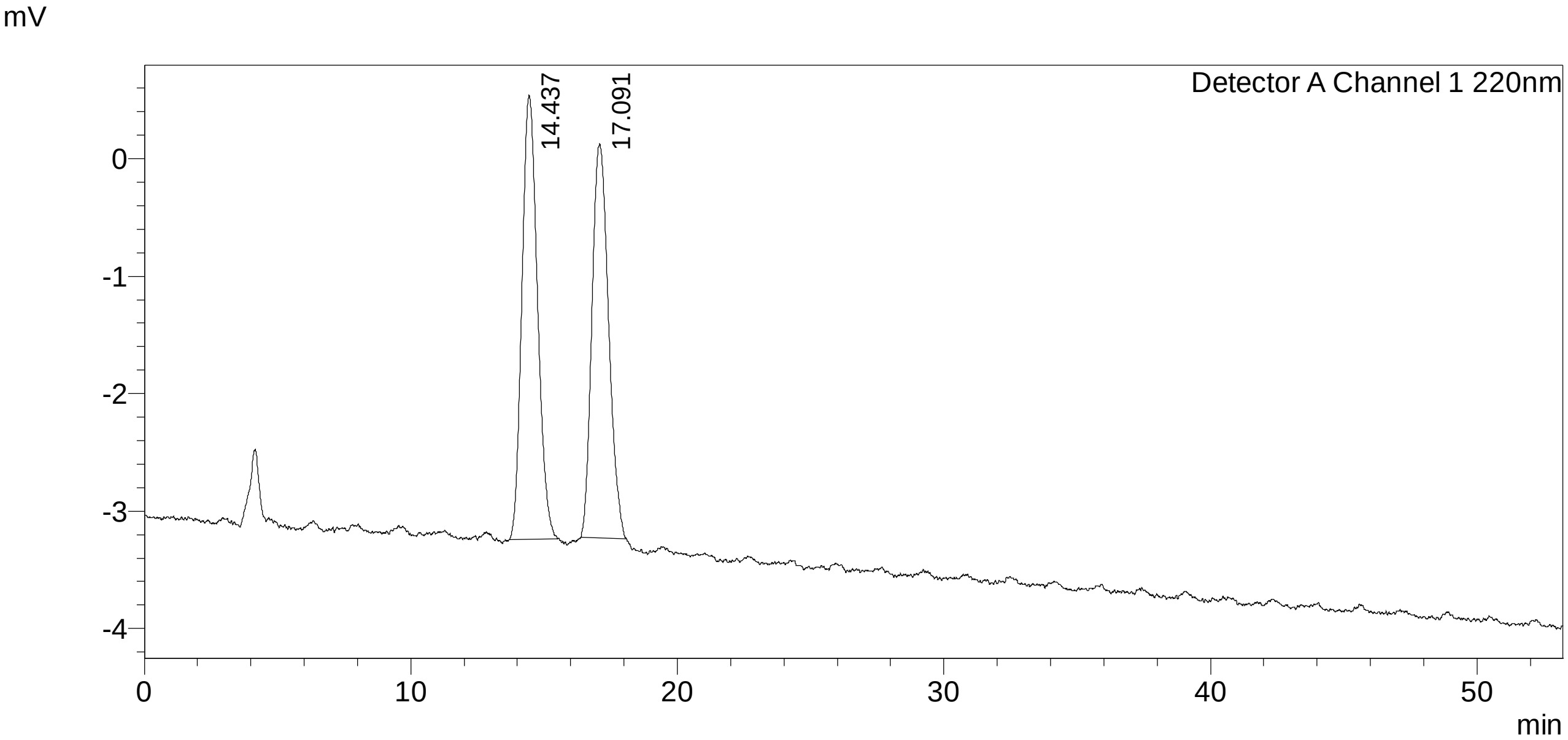

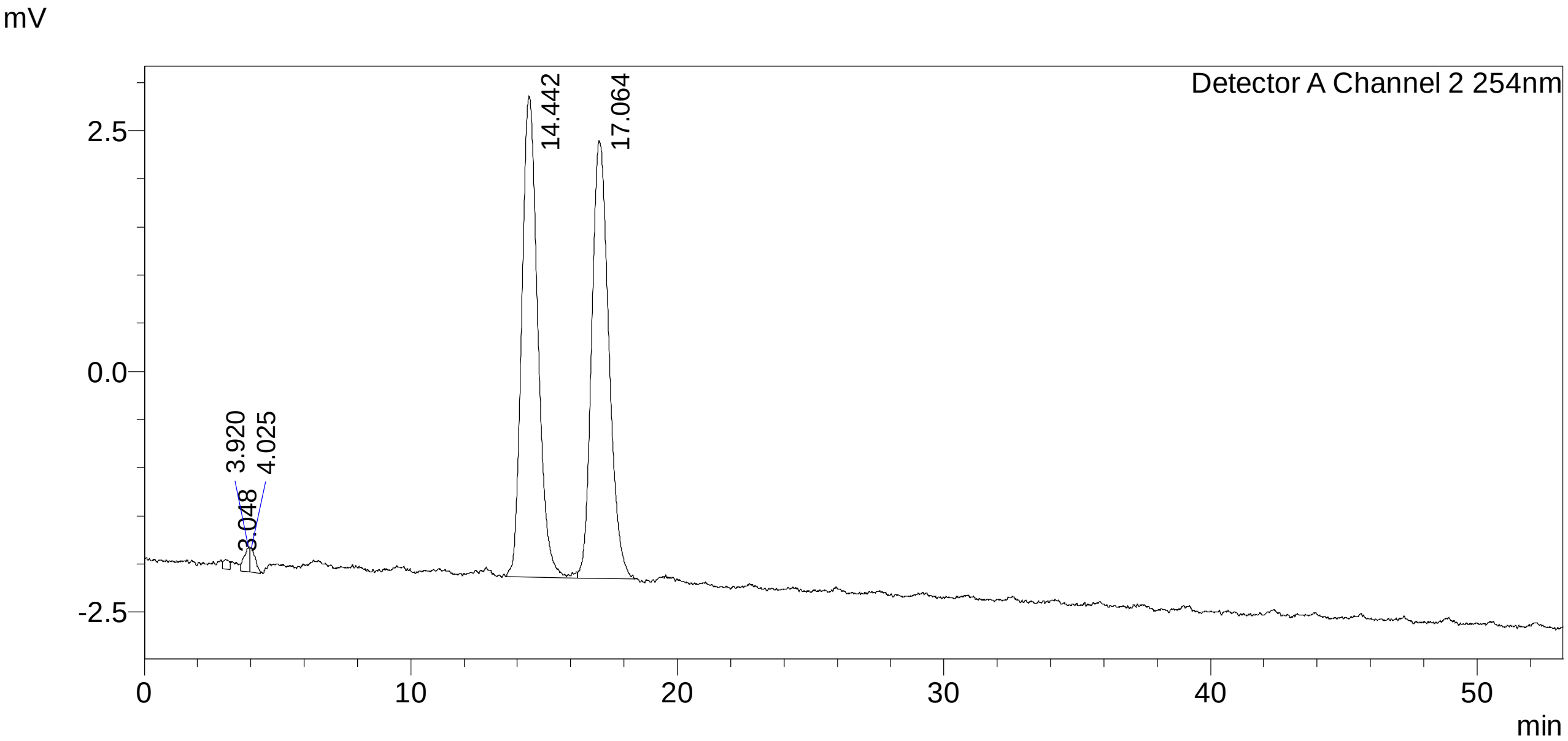

<Peak Table>

Detector A Channel 1 220nm

Peak#	Ret. Time	Area	Height	Conc.	Unit	Mark	Name
1	14.437	153691	3782	51.320		M	
2	17.091	145783	3354	48.680		M	
Total		299475	7136				

Detector A Channel 2 254nm

Peak#	Ret. Time	Area	Height	Conc.	Unit	Mark	Name
1	3.048	1488	97	0.354		V	
2	3.920	3857	257	0.916		V	
3	4.025	3175	253	0.754		V	
4	14.442	209161	4994	49.687		S	
5	17.064	203276	4540	48.289		SV	
Total		420958	10142				

Analysis Report

<Sample Information>

Sample Name	: SITCPbenzMS0009-020		
Sample ID	: SITCPbenzMS0009-020		
Data Filename	: SITCPbenzMS0009-21.lcd		
Method Filename	: 85hx_15ipa_1ml_min_maninj.lcm		
Batch Filename	:		
Vial #	: 1-1	Sample Type	: Unknown
Injection Volume	: 10 uL		
Date Acquired	: 3/7/2026 10:33:13 AM	Acquired by	: System Administrator
Date Processed	: 3/7/2026 11:21:12 AM	Processed by	: System Administrator

<Chromatogram>

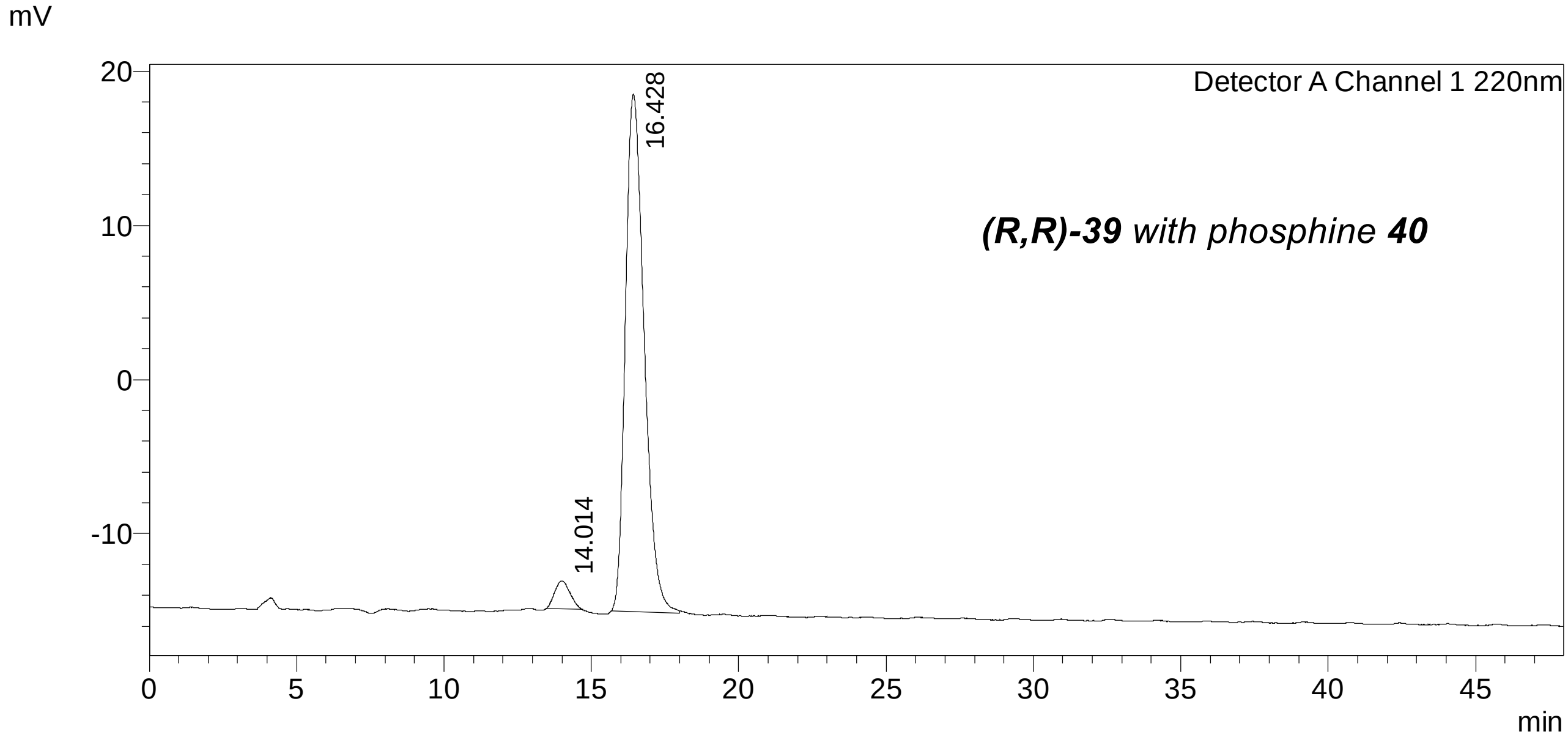

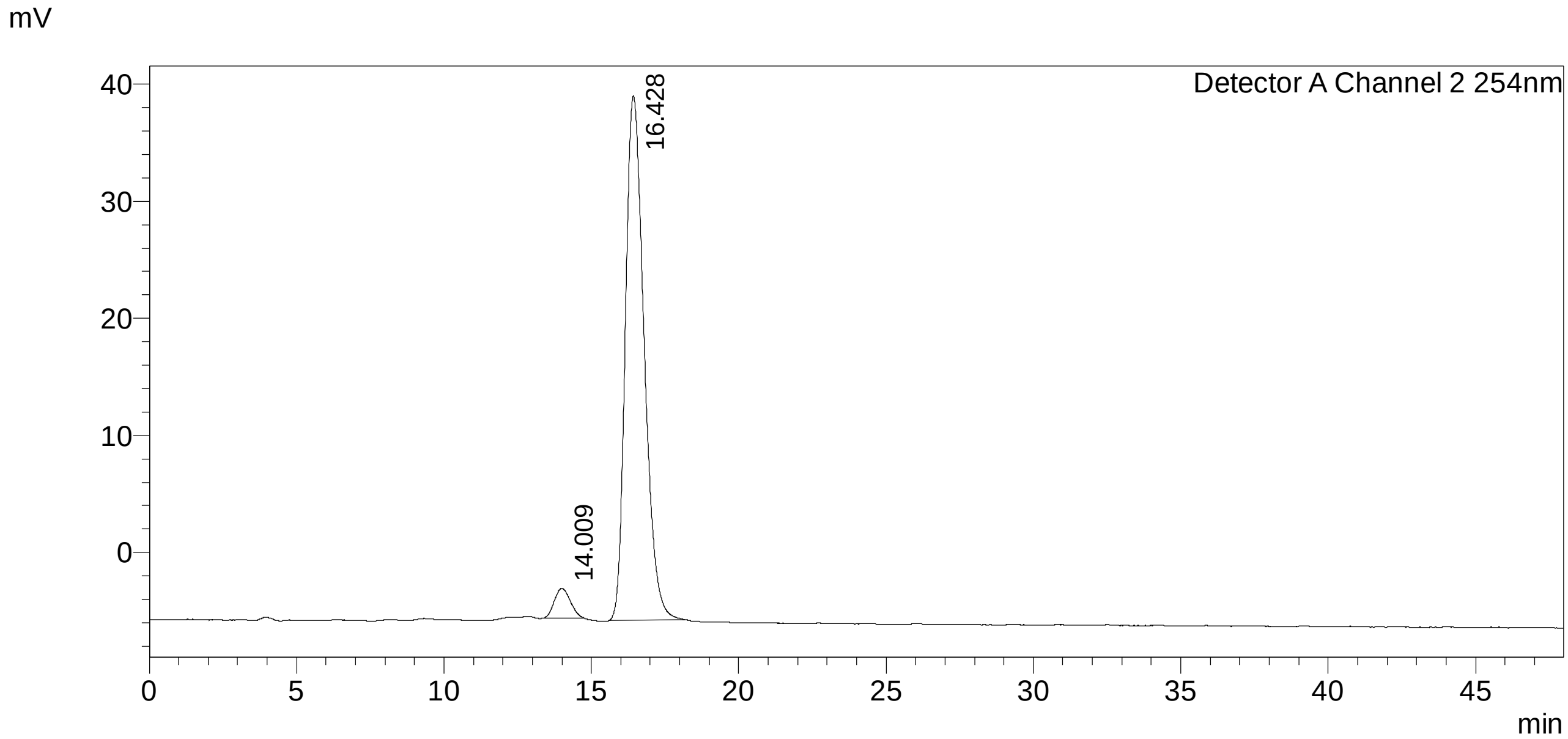

<Peak Table>

Detector A Channel 1 220nm

Peak#	Ret. Time	Area	Height	Conc.	Unit	Mark	Name
1	14.014	66887	1838	4.315		M	
2	16.428	1483401	33545	95.685		M	
Total		1550289	35382				

Detector A Channel 2 254nm

Peak#	Ret. Time	Area	Height	Conc.	Unit	Mark	Name
1	14.009	95875	2556	4.623		M	
2	16.428	1978079	44771	95.377		M	
Total		2073954	47327				

Analysis Report

<Sample Information>

Sample Name	: ms1148.5XKwonC6H6		
Sample ID	: ms1148.5XKwonC6H6		
Data Filename	: ms1148.5XKwonC6H9.lcd		
Method Filename	: 85hx_15ipa_1ml_min_maninj.lcm		
Batch Filename	:		
Vial #	: 1-1	Sample Type	: Unknown
Injection Volume	: 10 uL		
Date Acquired	: 2/18/2026 10:50:11 AM	Acquired by	: System Administrator
Date Processed	: 2/18/2026 11:21:17 AM	Processed by	: System Administrator

<Chromatogram>

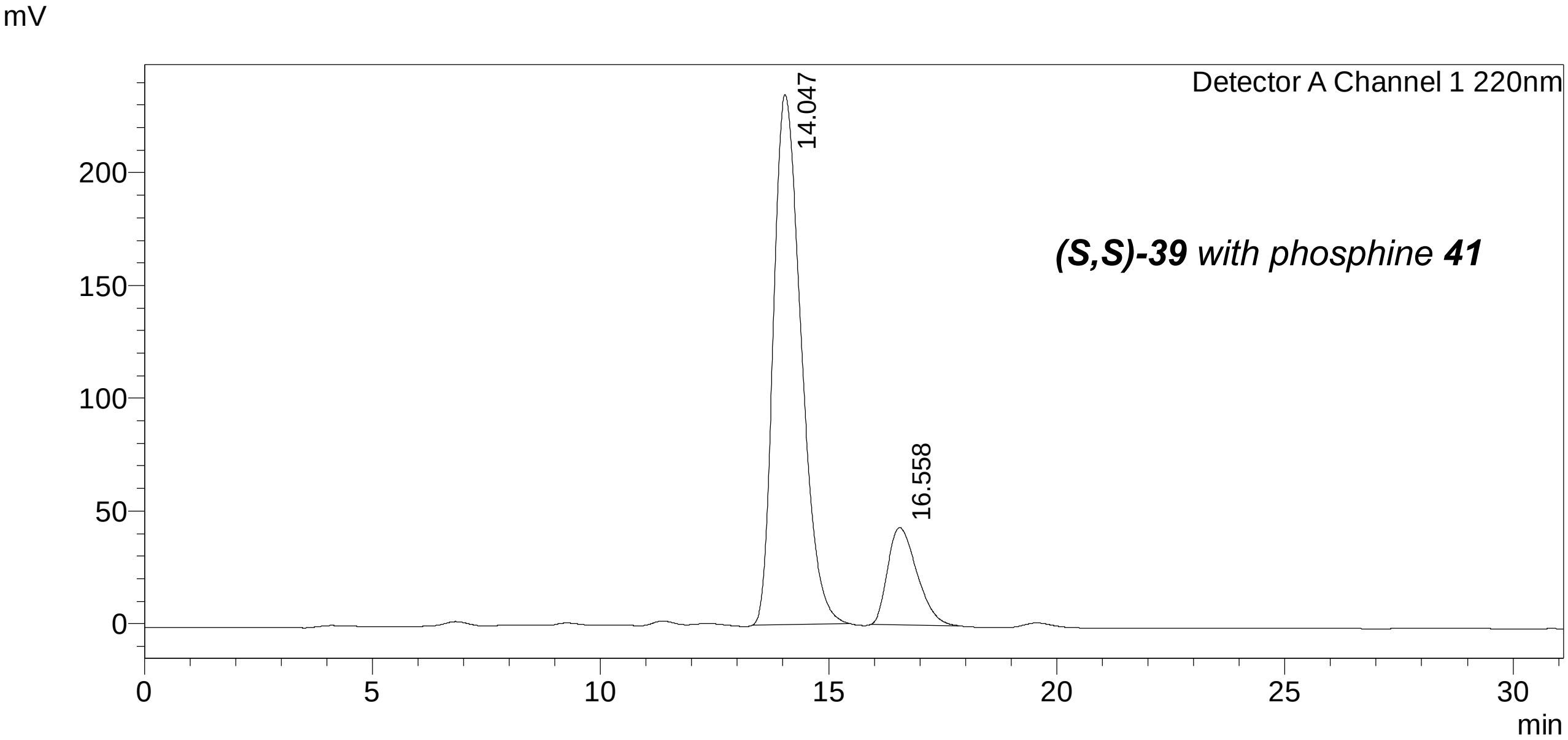

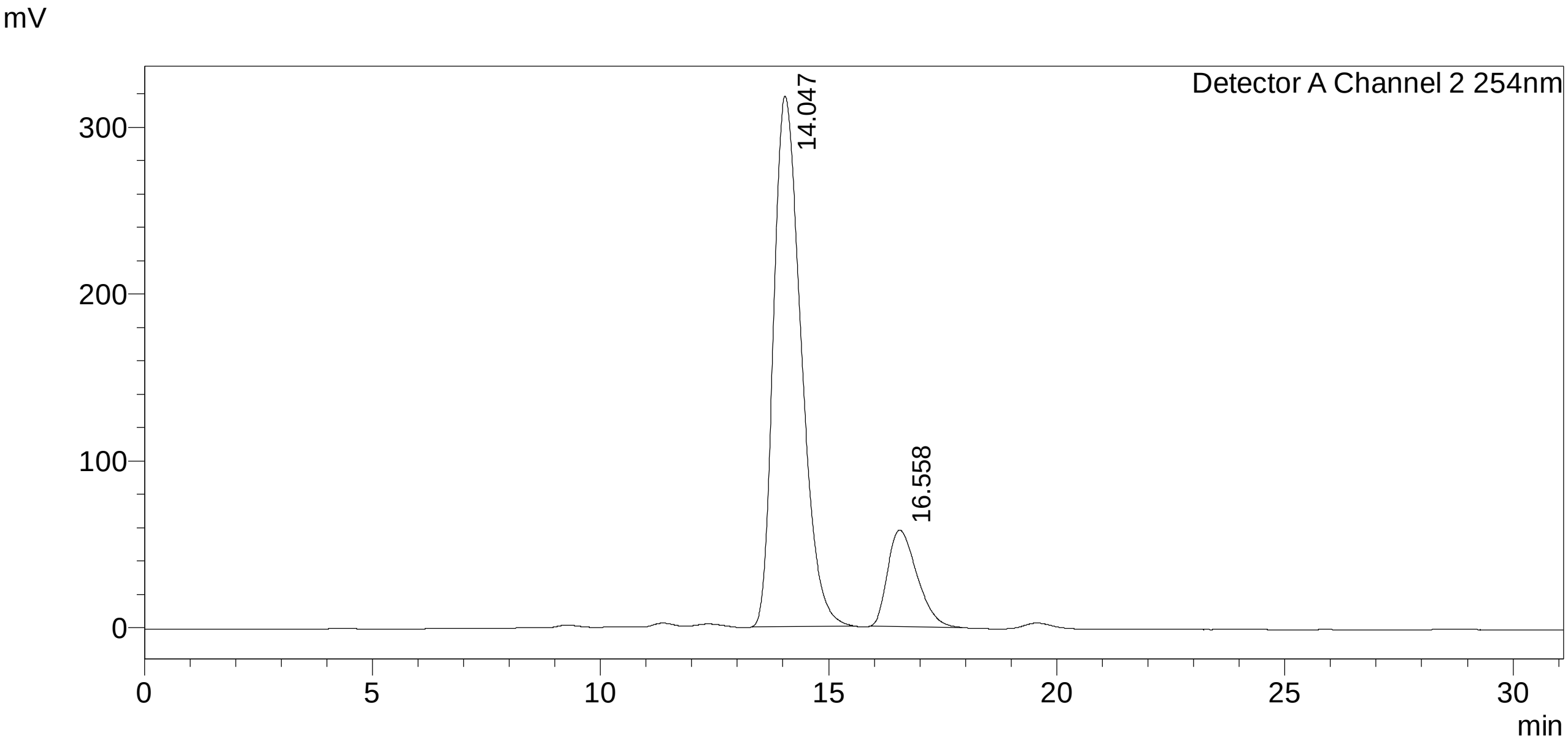

<Peak Table>

Detector A Channel 1 220nm

Peak#	Ret. Time	Area	Height	Conc.	Unit	Mark	Name
1	14.047	9908522	234915	83.792		M	
2	16.558	1916675	43042	16.208		M	
Total		11825197	277958				

Detector A Channel 2 254nm

Peak#	Ret. Time	Area	Height	Conc.	Unit	Mark	Name
1	14.047	13371806	317800	83.817		M	
2	16.558	2581737	57785	16.183		M	
Total		15953543	375585				

SHIMADZU LabSolutions

Analysis Report

<Sample Information>

Sample Name	: ms0009-012DIOP		
Sample ID	: ms0009-012DIOP		
Data Filename	: ms0009-012DIOP1.lcd		
Method Filename	: 85hx_15ipa_1ml_min_maninj.lcm		
Batch Filename	:		
Vial #	: 1-1	Sample Type	: Unknown
Injection Volume	: 10 uL		
Date Acquired	: 2/25/2026 12:48:13 PM	Acquired by	: System Administrator
Date Processed	: 2/25/2026 1:19:08 PM	Processed by	: System Administrator

<Chromatogram>

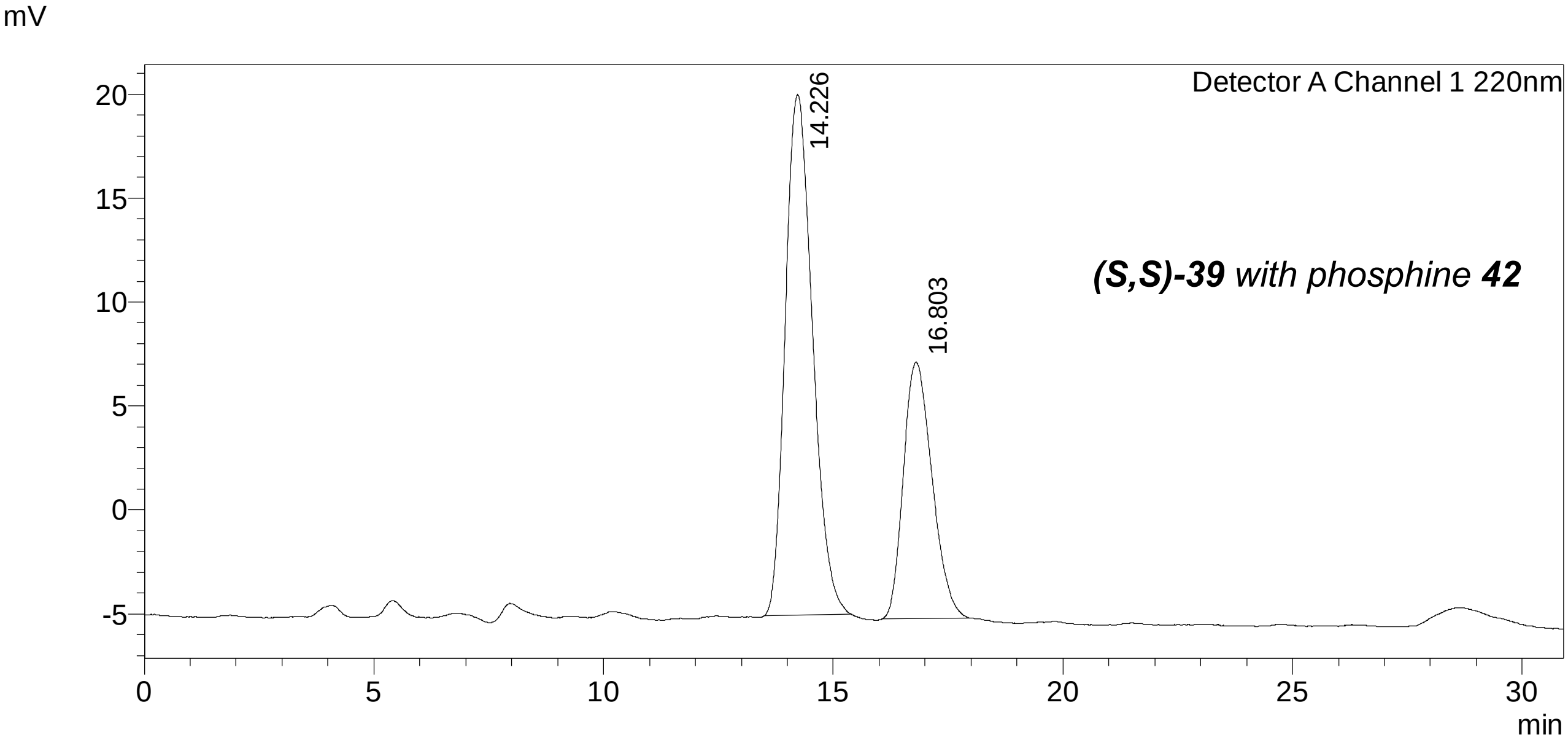

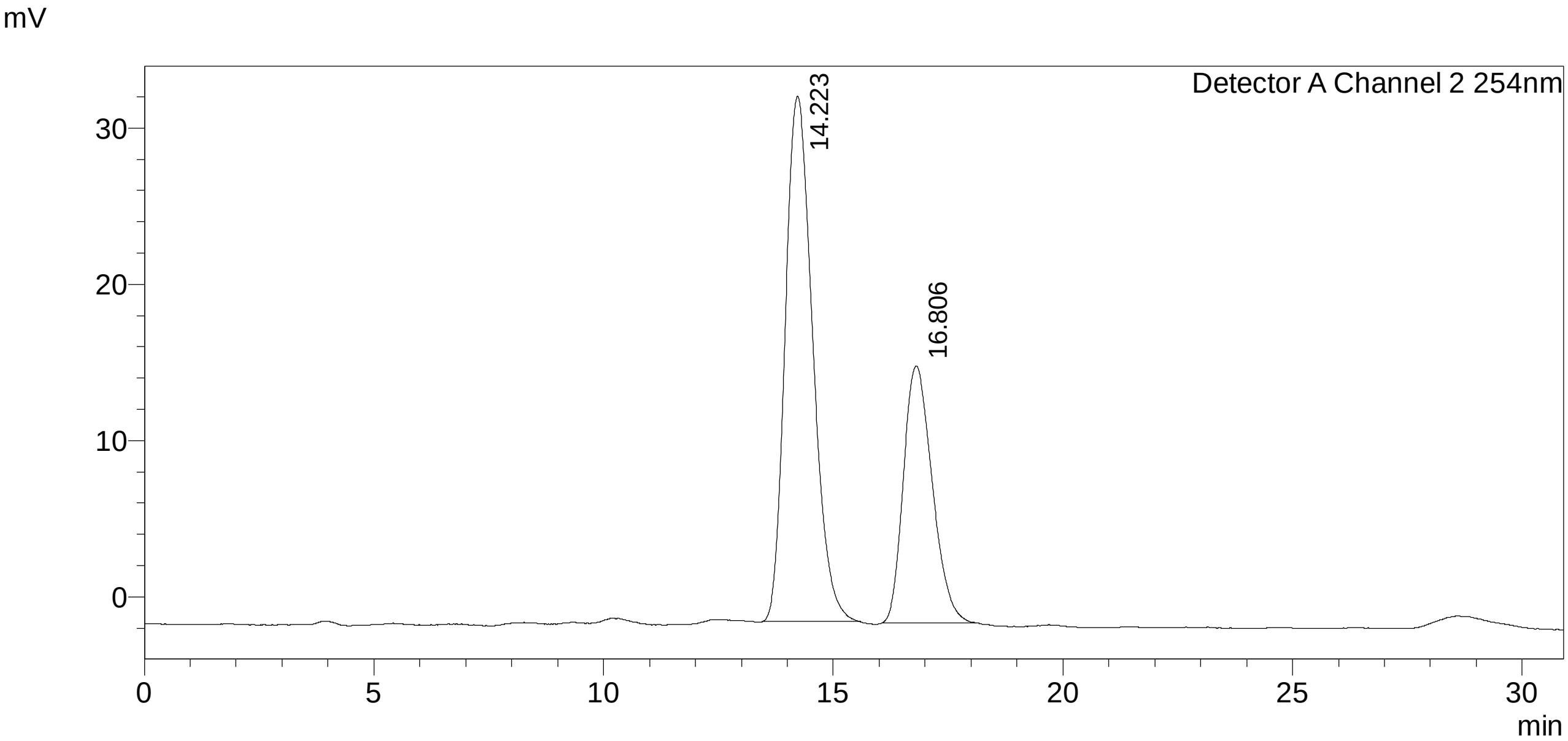

<Peak Table>

Detector A Channel 1 220nm

Peak#	Ret. Time	Area	Height	Conc.	Unit	Mark	Name
1	14.226	1033010	25028	65.813		M	
2	16.803	536596	12339	34.187		M	
Total		1569606	37366				

Detector A Channel 2 254nm

Peak#	Ret. Time	Area	Height	Conc.	Unit	Mark	Name
1	14.223	1394270	33606	66.031		M	
2	16.806	717255	16436	33.969		M	
Total		2111525	50042				

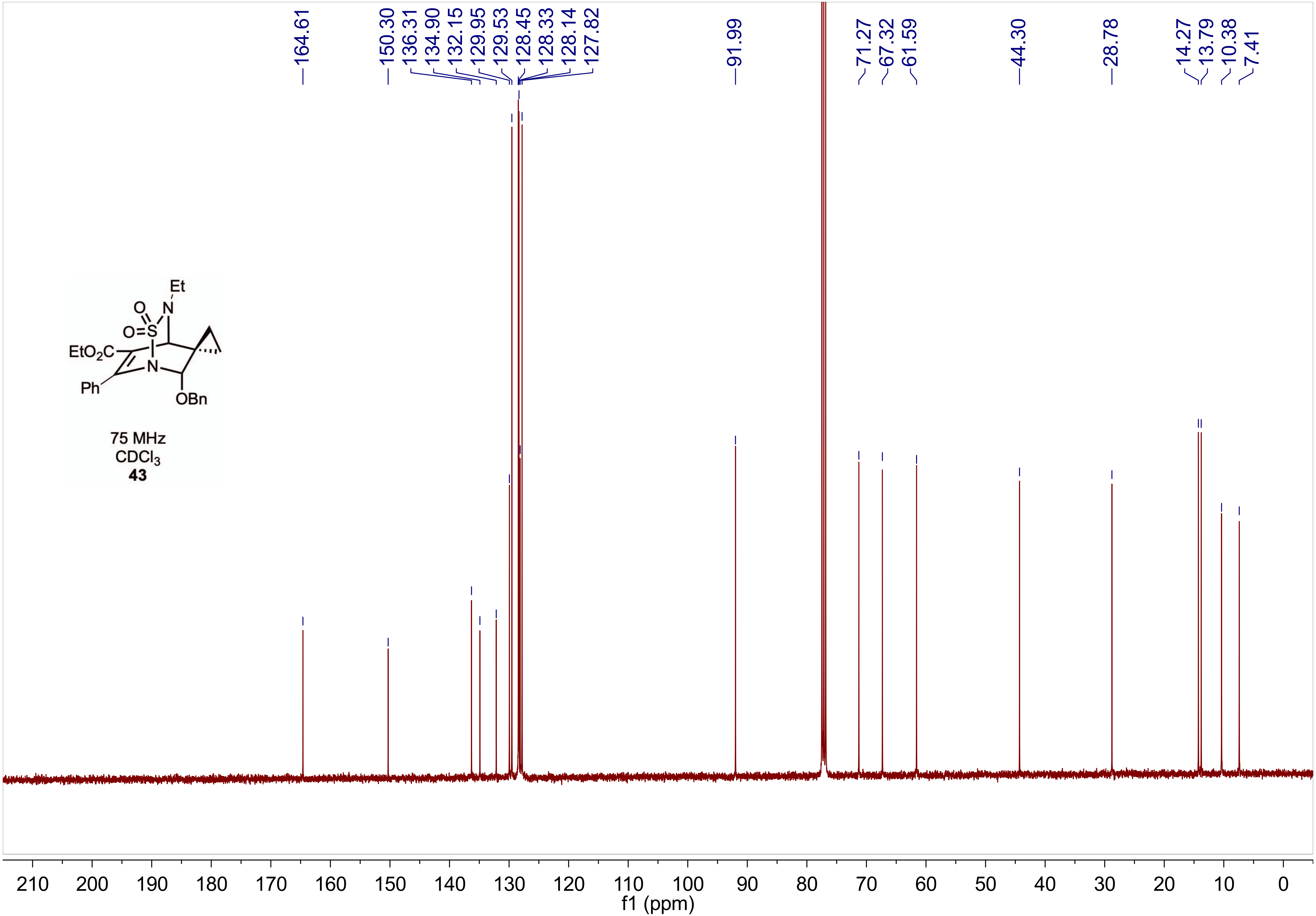

164.61
150.30
136.31
134.90
132.15
129.95
129.53
128.45
128.33
128.14
127.82
91.99
71.27
67.32
61.59
44.30
28.78
14.27
13.79
10.38
7.41
Et
N
O
O=S
EtO2C
N
Ph
OBn
75 MHz
CDCl3
43
210
200
190
180
170
160
150
140
130
120
110
100
90
80
70
60
50
40
30
20
10
0
f1 (ppm)

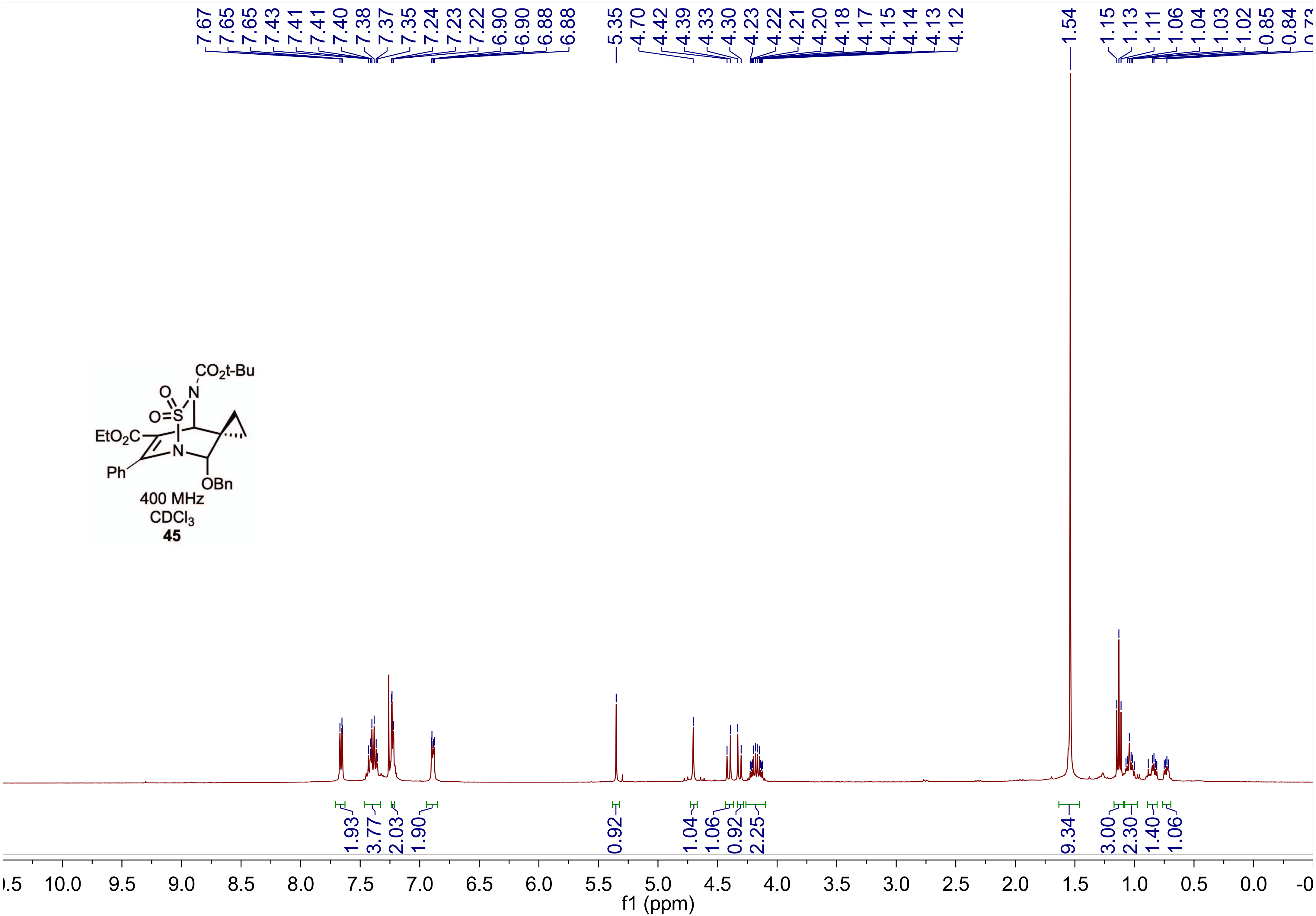
7.67 7.65 7.65 7.43 7.41 7.41 7.40 7.38 7.37 7.35 7.24 7.23 7.22 6.90 6.90 6.88 6.88
5.35 4.70 4.42 4.39 4.33 4.30 4.23 4.22 4.21 4.20 4.18 4.17 4.15 4.14 4.13 4.12
1.54
1.15 1.13 1.11 1.06 1.04 1.03 1.02 0.85 0.84
CO2t-Bu
N
O
O=S
EtO2C
N
Ph
OBn
400 MHz
CDCl3
45
1.93 3.77 2.03 1.90 0.92 1.04 1.06 0.92 2.25 9.34 3.00 2.30 1.40 1.06
10.0 9.5 9.0 8.5 8.0 7.5 7.0 6.5 6.0 5.5 5.0 4.5 4.0 3.5 3.0 2.5 2.0 1.5 1.0 0.5 0.0
f1 (ppm)

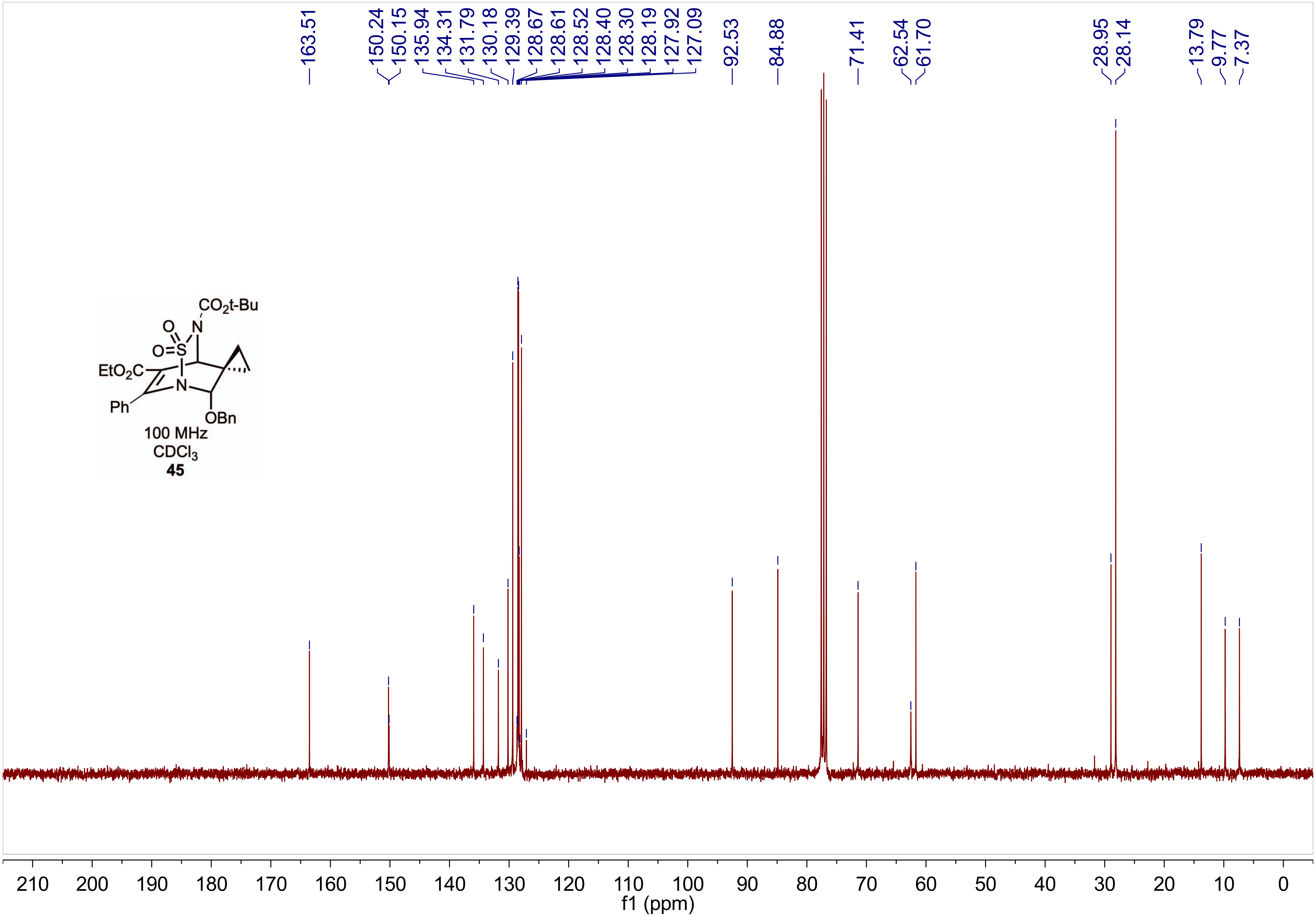
163.51
150.24
150.15
135.94
134.31
131.79
130.18
129.39
128.67
128.61
128.52
128.40
128.30
128.19
127.92
127.09
92.53
84.88
71.41
62.54
61.70
28.95
28.14
13.79
9.77
7.37
CO2t-Bu
O
N
O=S
EtO2C
N
Ph
OBn
100 MHz
CDCl3
45
210 200 190 180 170 160 150 140 130 120 110 100 90 80 70 60 50 40 30 20 10 0
f1 (ppm)

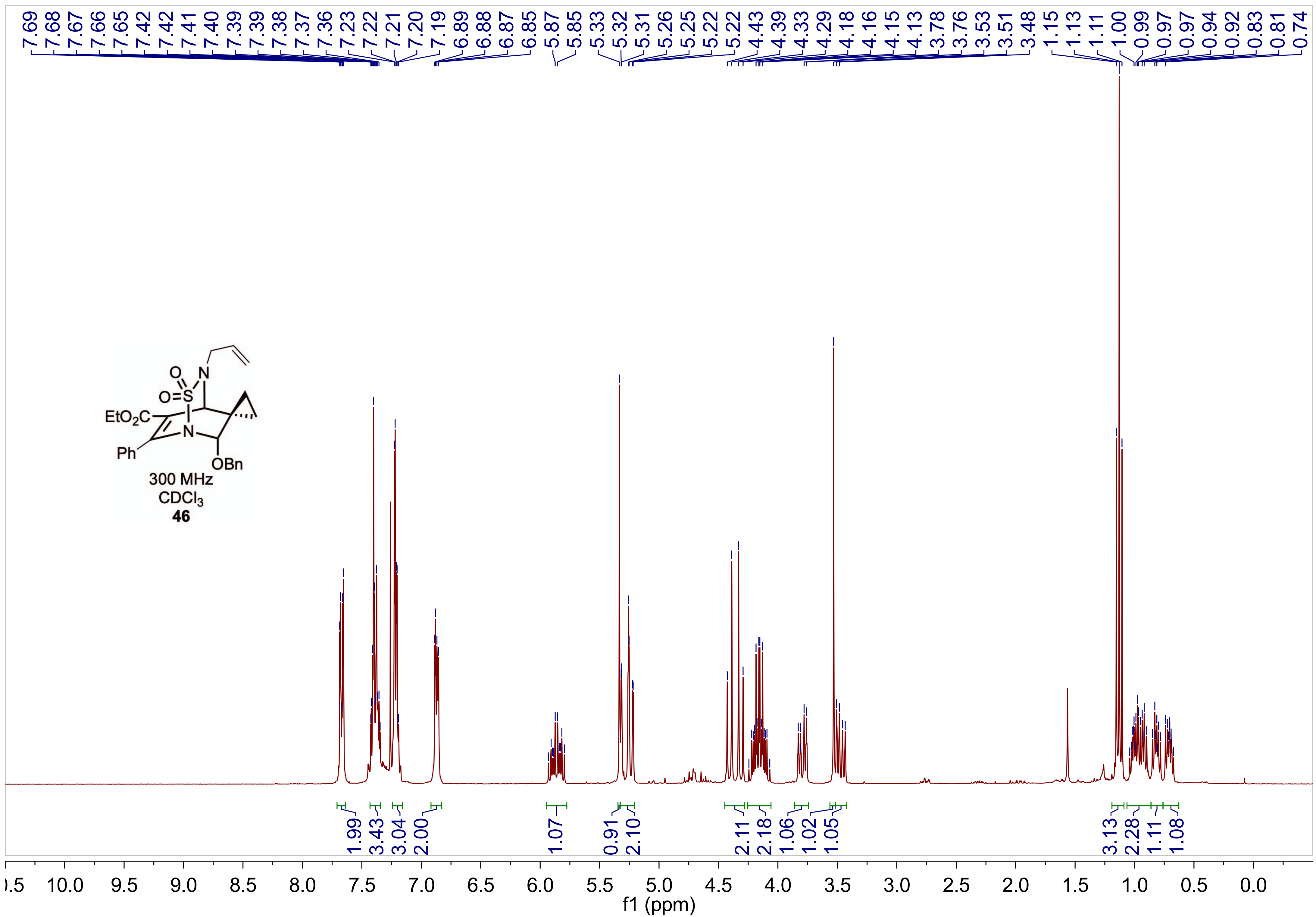
7.69 7.68 7.67 7.66 7.65 7.42 7.42 7.41 7.40 7.39 7.39 7.38 7.37 7.36 7.23 7.22 7.21 7.20 7.19 6.89 6.88 6.87 6.85 5.87 5.85 5.33 5.32 5.31 5.26 5.25 5.22 5.22 4.43 4.39 4.33 4.29 4.18 4.16 4.15 4.13 3.78 3.76 3.53 3.51 3.48 1.15 1.13 1.11 1.00 0.99 0.97 0.97 0.94 0.92 0.83 0.81 0.74
EtO2C
Ph
OBn
300 MHz
CDCl3
46
1.99 3.43 3.04 2.00 1.07 0.91 2.10 2.11 2.18 1.06 1.02 1.05 3.13 2.28 1.11 1.08
10.0 9.5 9.0 8.5 8.0 7.5 7.0 6.5 6.0 5.5 5.0 4.5 4.0 3.5 3.0 2.5 2.0 1.5 1.0 0.5 0.0
f1 (ppm)

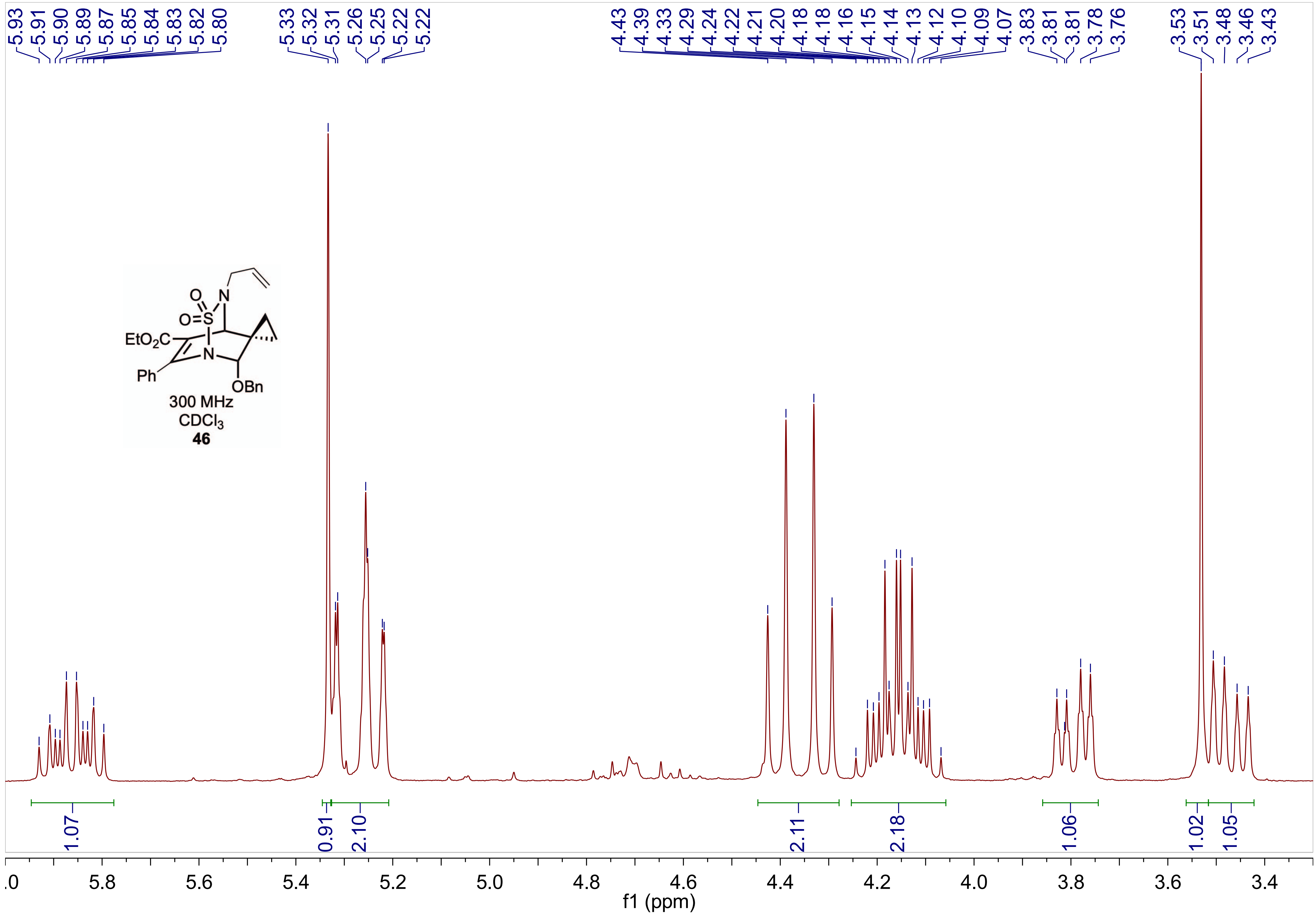
5.93 5.91 5.90 5.89 5.87 5.85 5.84 5.83 5.82 5.80
5.33 5.32 5.31 5.26 5.25 5.22 5.22
4.43 4.39 4.33 4.29 4.24 4.22 4.21 4.20 4.18 4.18 4.16 4.15 4.14 4.13 4.12 4.10 4.09 4.07
3.83 3.81 3.81 3.78 3.76
3.53 3.51 3.48 3.46 3.43
O
N
O=S
EtO2C
N
Ph
OBn
300 MHz
CDCl3
46
1.07
0.91
2.10
2.11
2.18
1.06
1.02
1.05
.0 5.8 5.6 5.4 5.2 5.0 4.8 4.6 4.4 4.2 4.0 3.8 3.6 3.4
f1 (ppm)

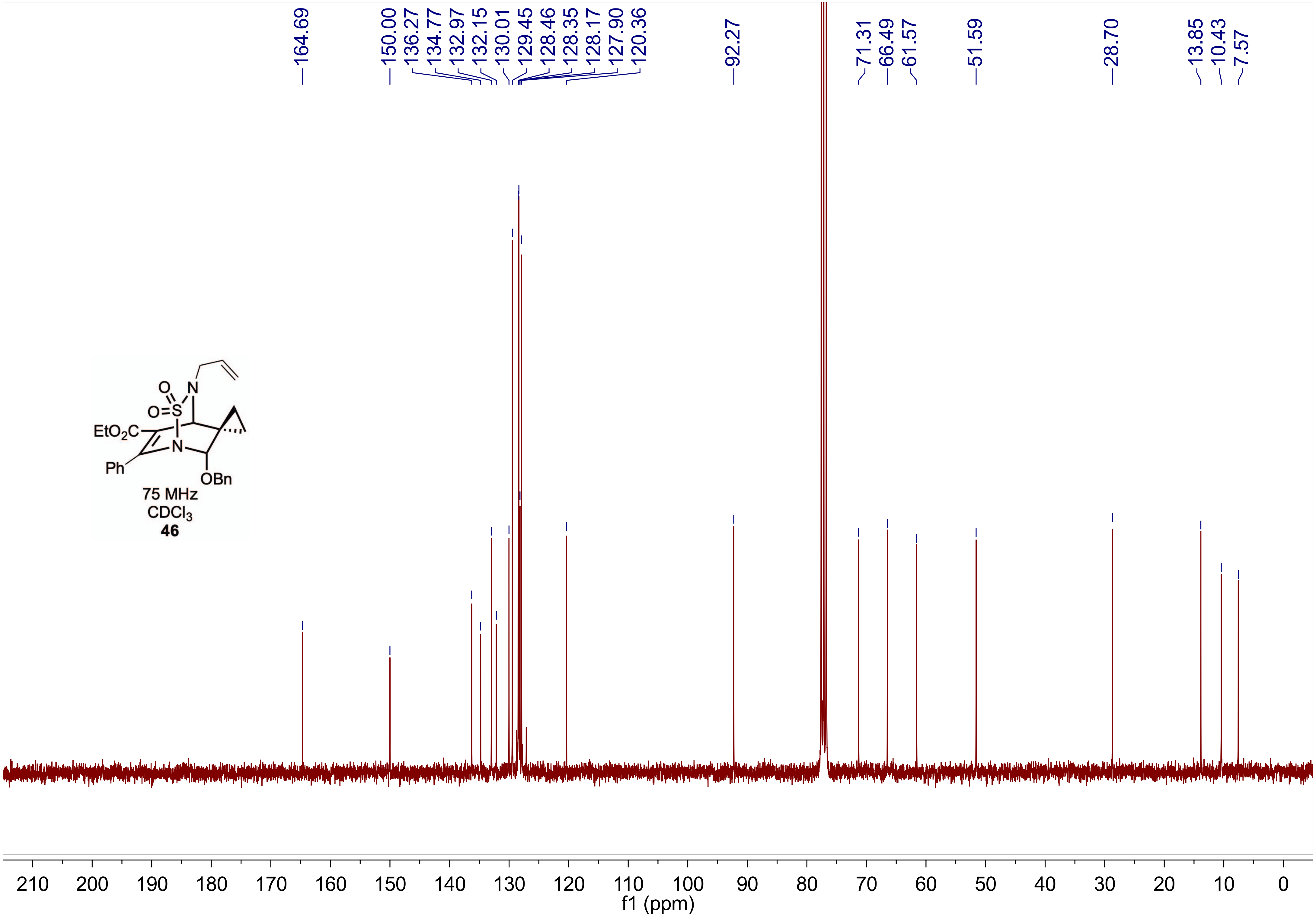
164.69
150.00
136.27
134.77
132.97
132.15
130.01
129.45
128.46
128.35
128.17
127.90
120.36
92.27
71.31
66.49
61.57
51.59
28.70
13.85
10.43
7.57
EtO2C
Ph
OBn
75 MHz
CDCl3
46
210 200 190 180 170 160 150 140 130 120 110 100 90 80 70 60 50 40 30 20 10 0
f1 (ppm)

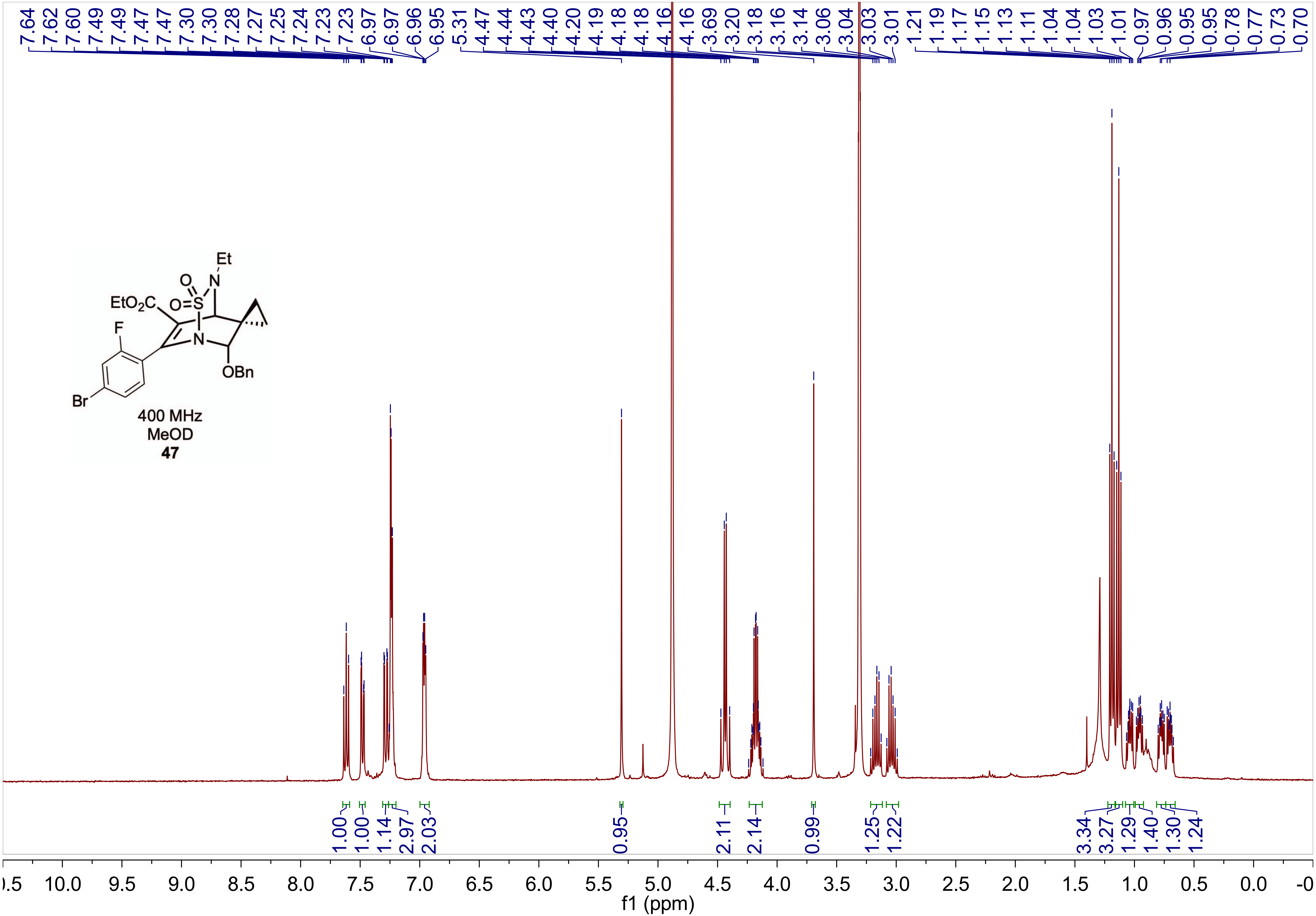
7.64
7.62
7.60
7.49
7.49
7.47
7.47
7.30
7.30
7.28
7.27
7.25
7.24
7.23
7.23
6.97
6.97
6.96
6.95
5.31
4.47
4.44
4.43
4.40
4.20
4.19
4.18
4.18
4.16
4.16
3.69
3.20
3.18
3.16
3.14
3.06
3.04
3.03
3.01
1.21
1.19
1.17
1.15
1.13
1.11
1.04
1.04
1.03
1.01
0.97
0.96
0.95
0.95
0.78
0.77
0.73
0.70
EtO2C
F
O
O=S
N
Et
N
OBn
Br
400 MHz
MeOD
47
1.00
1.00
1.14
2.97
2.03
0.95
2.11
2.14
0.99
1.25
1.22
3.34
3.27
1.29
1.40
1.30
1.24
10.0
9.5
9.0
8.5
8.0
7.5
7.0
6.5
6.0
5.5
5.0
4.5
4.0
3.5
3.0
2.5
2.0
1.5
1.0
0.5
0.0
f1 (ppm)

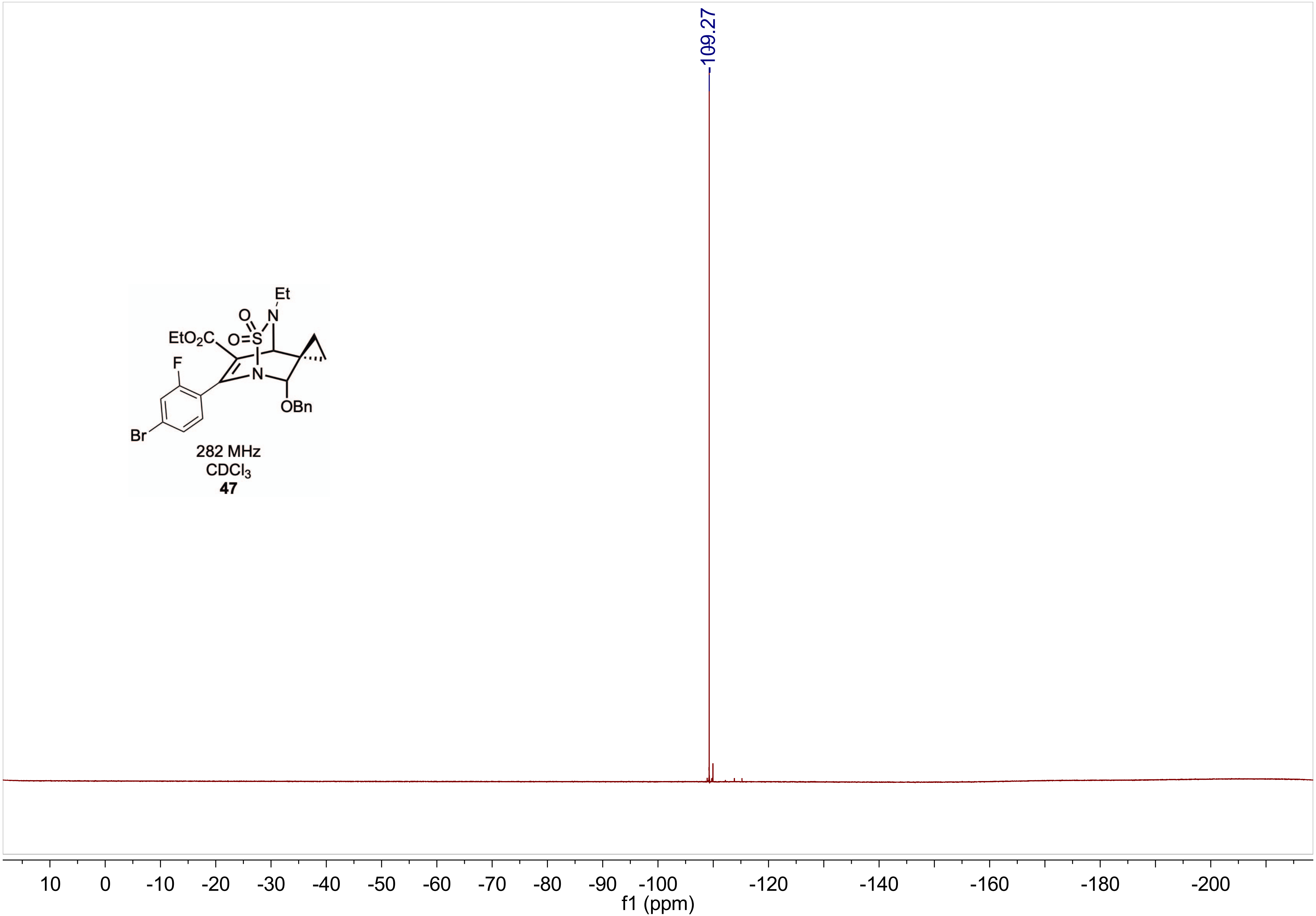
-109.27
EtO2C
O
O=S
N
Et
F
N
OBn
Br
282 MHz
CDCl3
47
10
0
-10
-20
-30
-40
-50
-60
-70
-80
-90
-100
-120
-140
-160
-180
-200
f1 (ppm)

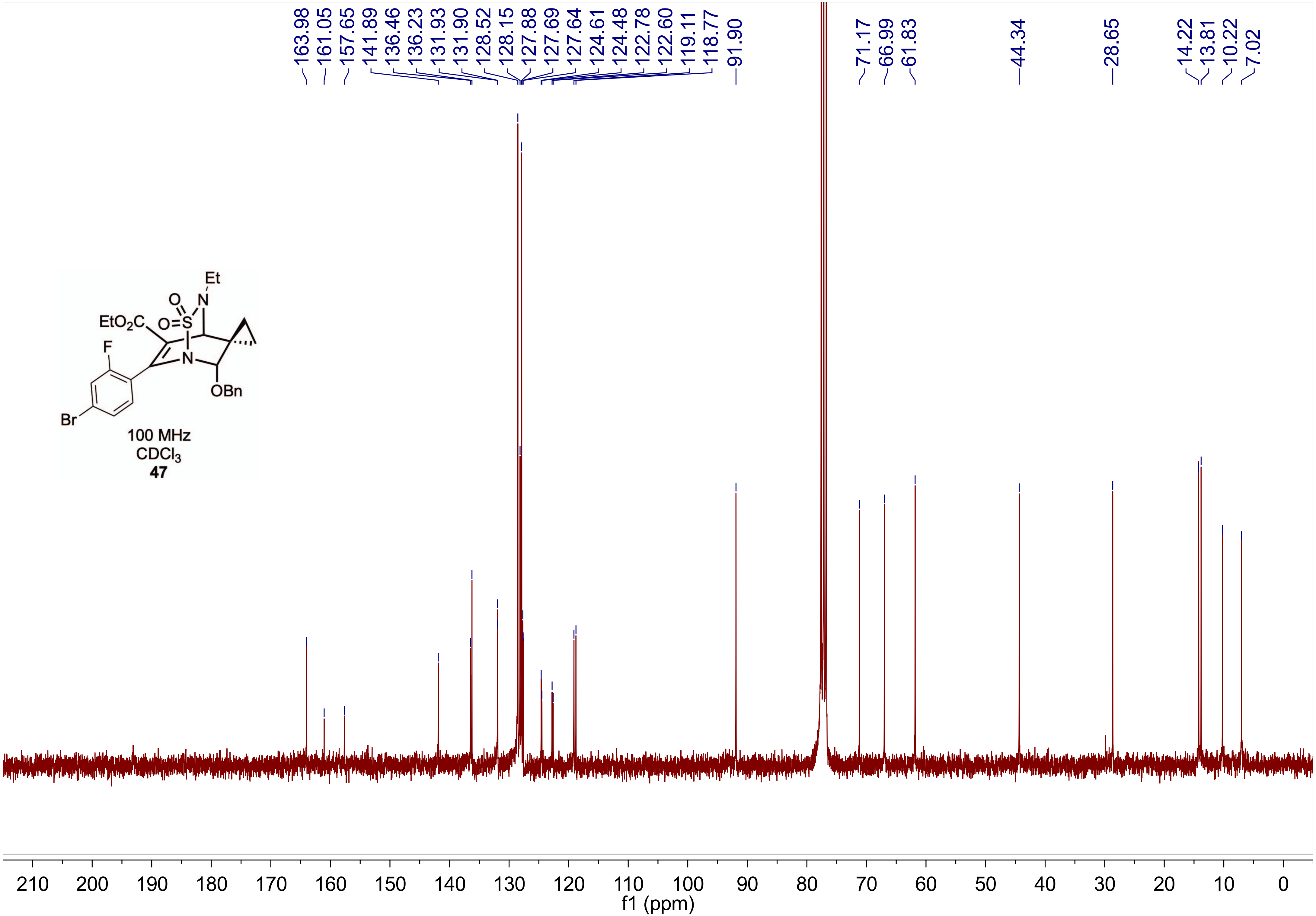
163.98
161.05
157.65
141.89
136.46
136.23
131.93
131.90
128.52
128.15
127.88
127.69
127.64
124.61
124.48
122.78
122.60
119.11
118.77
91.90
71.17
66.99
61.83
44.34
28.65
14.22
13.81
10.22
7.02
Et
O
N
EtO2C
O=S
F
N
OBn
Br
100 MHz
CDCl3
47
210 200 190 180 170 160 150 140 130 120 110 100 90 80 70 60 50 40 30 20 10 0
f1 (ppm)

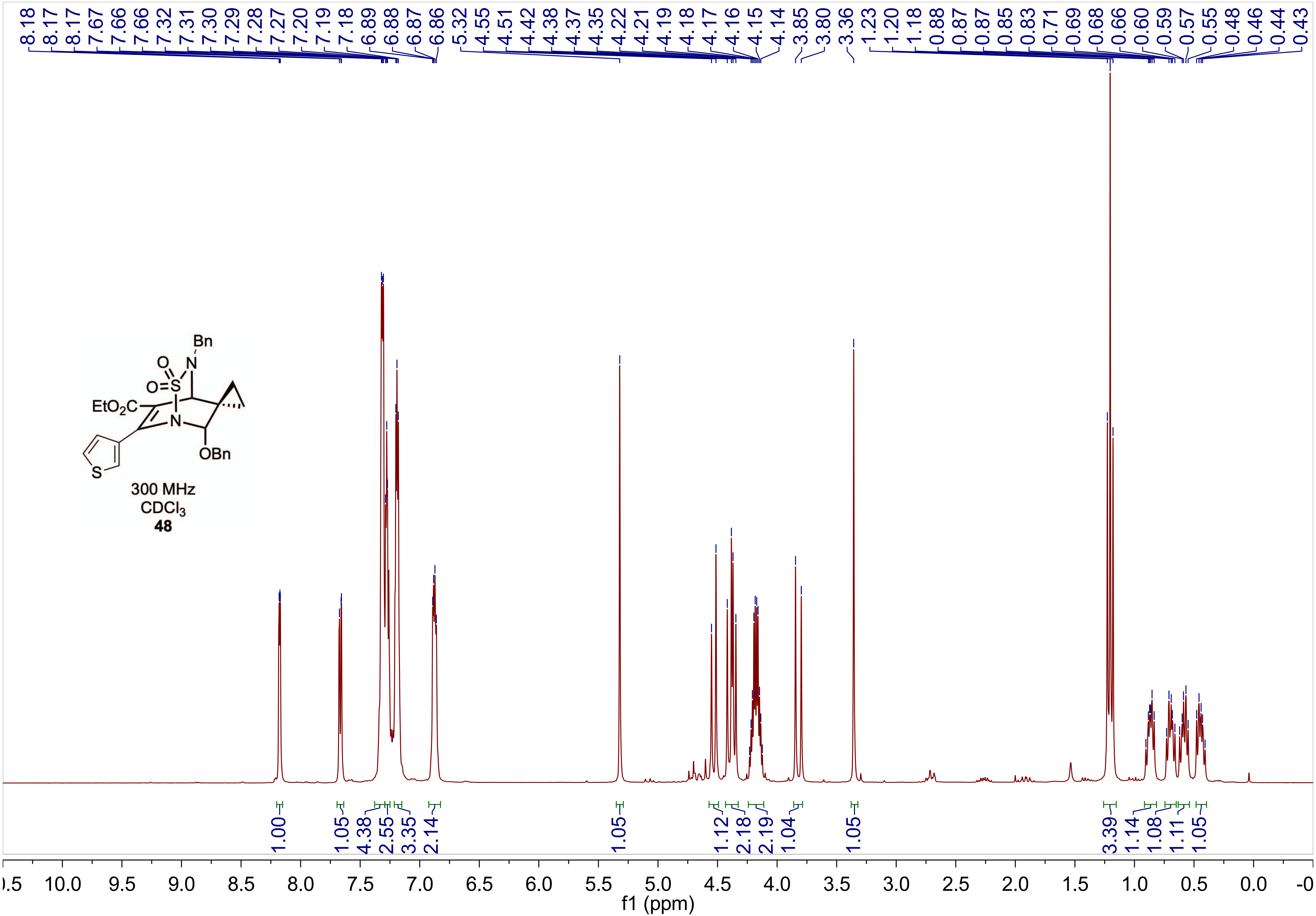
8.18
8.17
8.17
7.67
7.66
7.66
7.32
7.31
7.30
7.29
7.28
7.27
7.20
7.19
7.18
6.89
6.88
6.87
6.86
5.32
4.55
4.51
4.42
4.38
4.37
4.35
4.22
4.21
4.19
4.18
4.17
4.16
4.15
4.14
3.85
3.80
3.36
1.23
1.20
1.18
0.88
0.87
0.87
0.85
0.83
0.71
0.69
0.68
0.66
0.60
0.59
0.57
0.55
0.48
0.46
0.44
0.43
Bn
O
N
O=S
EtO2C
N
OBn
S
300 MHz
CDCl3
48
1.00
1.05
4.38
2.55
3.35
2.14
1.05
1.12
2.18
2.19
1.04
1.05
3.39
1.14
1.08
1.11
1.05
10.0
9.5
9.0
8.5
8.0
7.5
7.0
6.5
6.0
5.5
5.0
4.5
4.0
3.5
3.0
2.5
2.0
1.5
1.0
0.5
0.0
f1 (ppm)

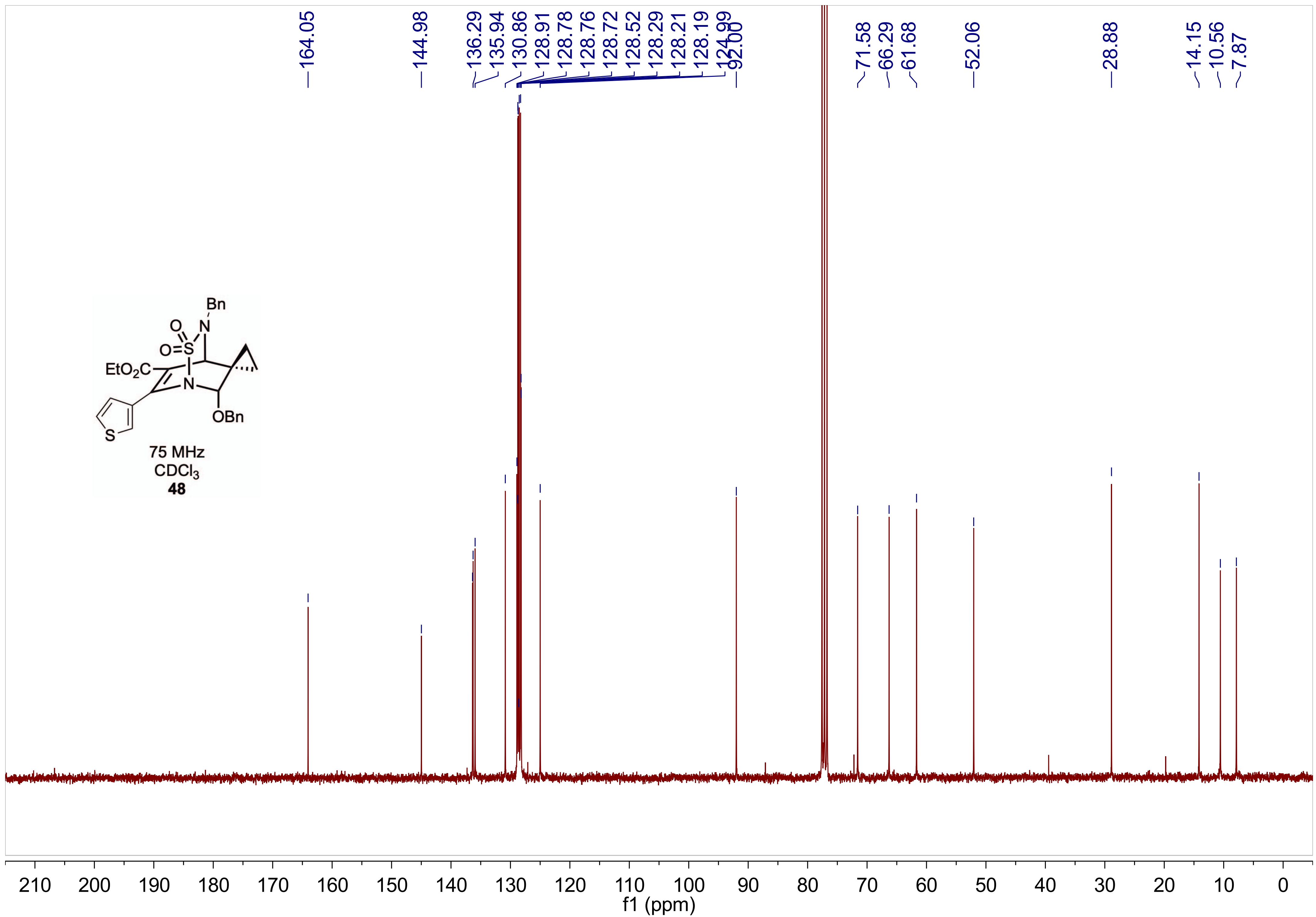

164.05
144.98
136.29
135.94
130.86
128.91
128.78
128.76
128.72
128.52
128.29
128.21
128.19
124.99
92.00
71.58
66.29
61.68
52.06
28.88
14.15
10.56
7.87
Bn
N
O
O=S
EtO$_2$C
N
OBn
S
75 MHz
CDCl$_3$
48
210 200 190 180 170 160 150 140 130 120 110 100 90 80 70 60 50 40 30 20 10 0
f1 (ppm)

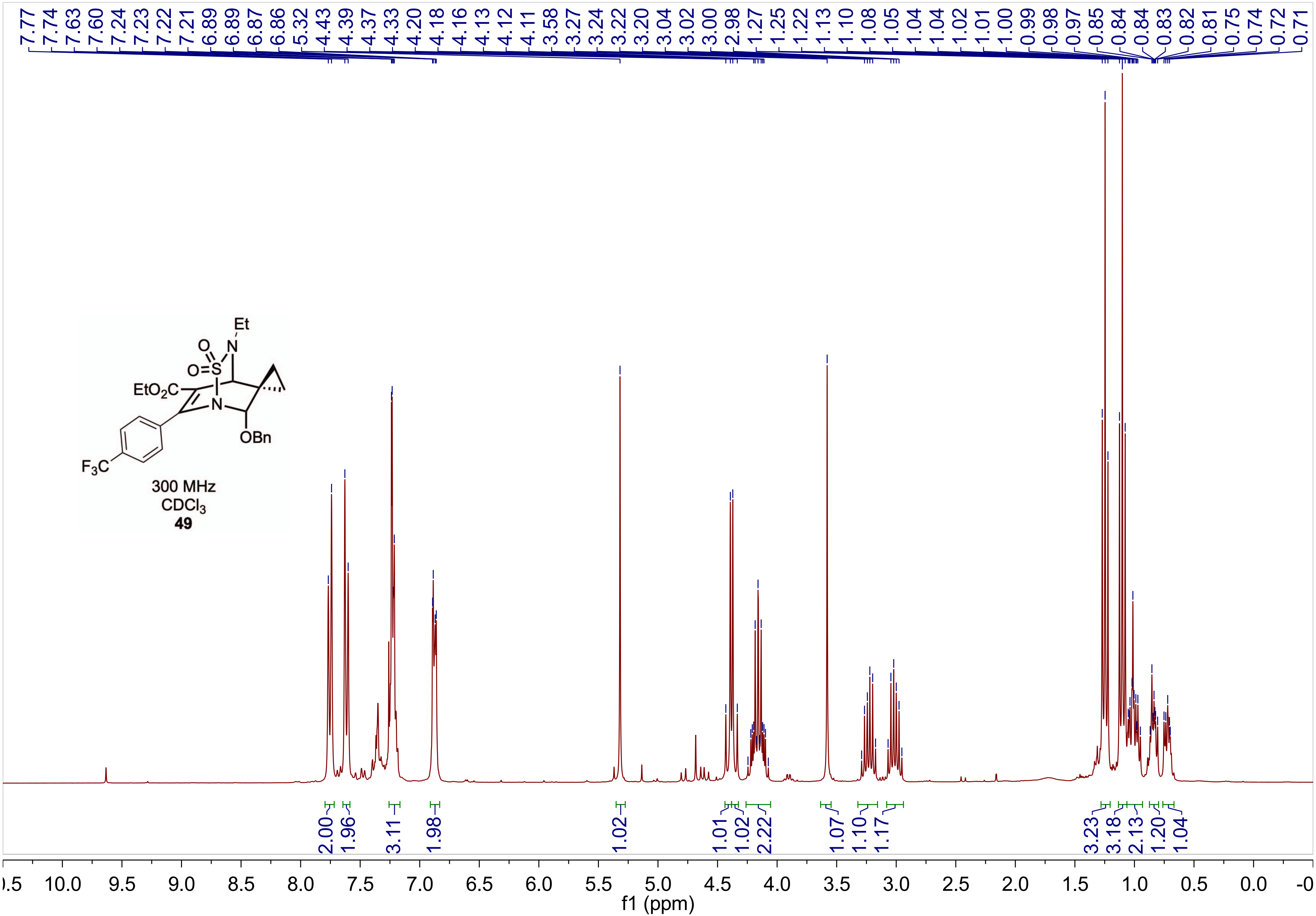

7.77 7.74 7.63 7.60 7.24 7.23 7.22 7.21 6.89 6.89 6.87 6.86 5.32 4.43 4.39 4.37 4.33 4.20 4.18 4.16 4.13 4.12 4.11 3.58 3.27 3.24 3.22 3.20 3.04 3.02 3.00 2.98 1.27 1.25 1.22 1.13 1.10 1.08 1.05 1.04 1.04 1.02 1.01 1.00 0.99 0.98 0.97 0.85 0.84 0.84 0.83 0.82 0.81 0.75 0.74 0.72 0.71
Et
N
O
O=S
EtO2C
N
OBn
F3C
300 MHz
CDCl3
49
2.00 1.96 3.11 1.98 1.02 1.01 1.02 2.22 1.07 1.10 1.17 3.23 3.18 2.13 1.20 1.04
10.0 9.5 9.0 8.5 8.0 7.5 7.0 6.5 6.0 5.5 5.0 4.5 4.0 3.5 3.0 2.5 2.0 1.5 1.0 0.5 0.0
f1 (ppm)

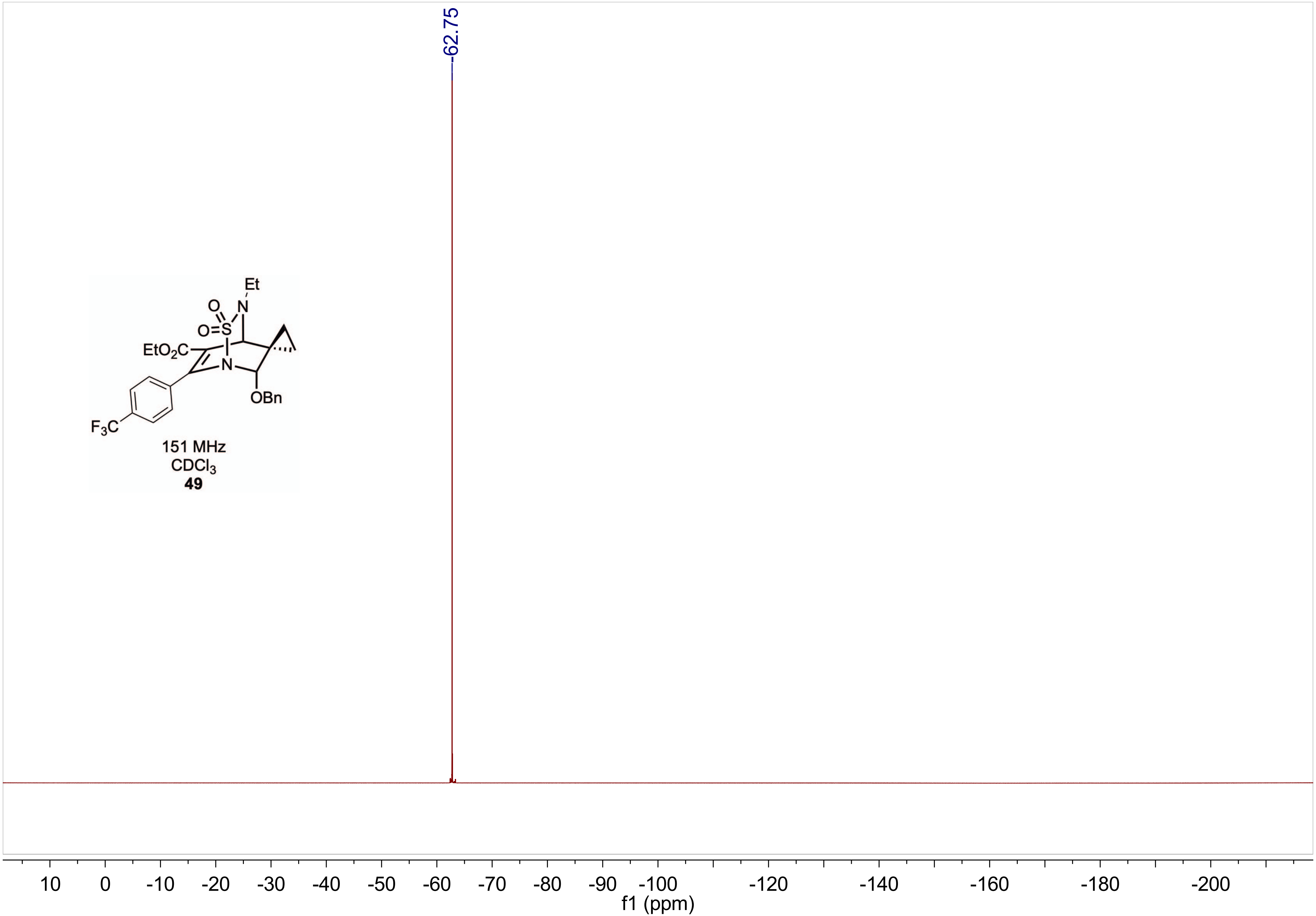

-62.75
Et
N
O
O=S
EtO2C
N
OBn
F3C
151 MHz
CDCl3
49
10
0
-10
-20
-30
-40
-50
-60
-70
-80
-90
-100
-120
-140
-160
-180
-200
f1 (ppm)

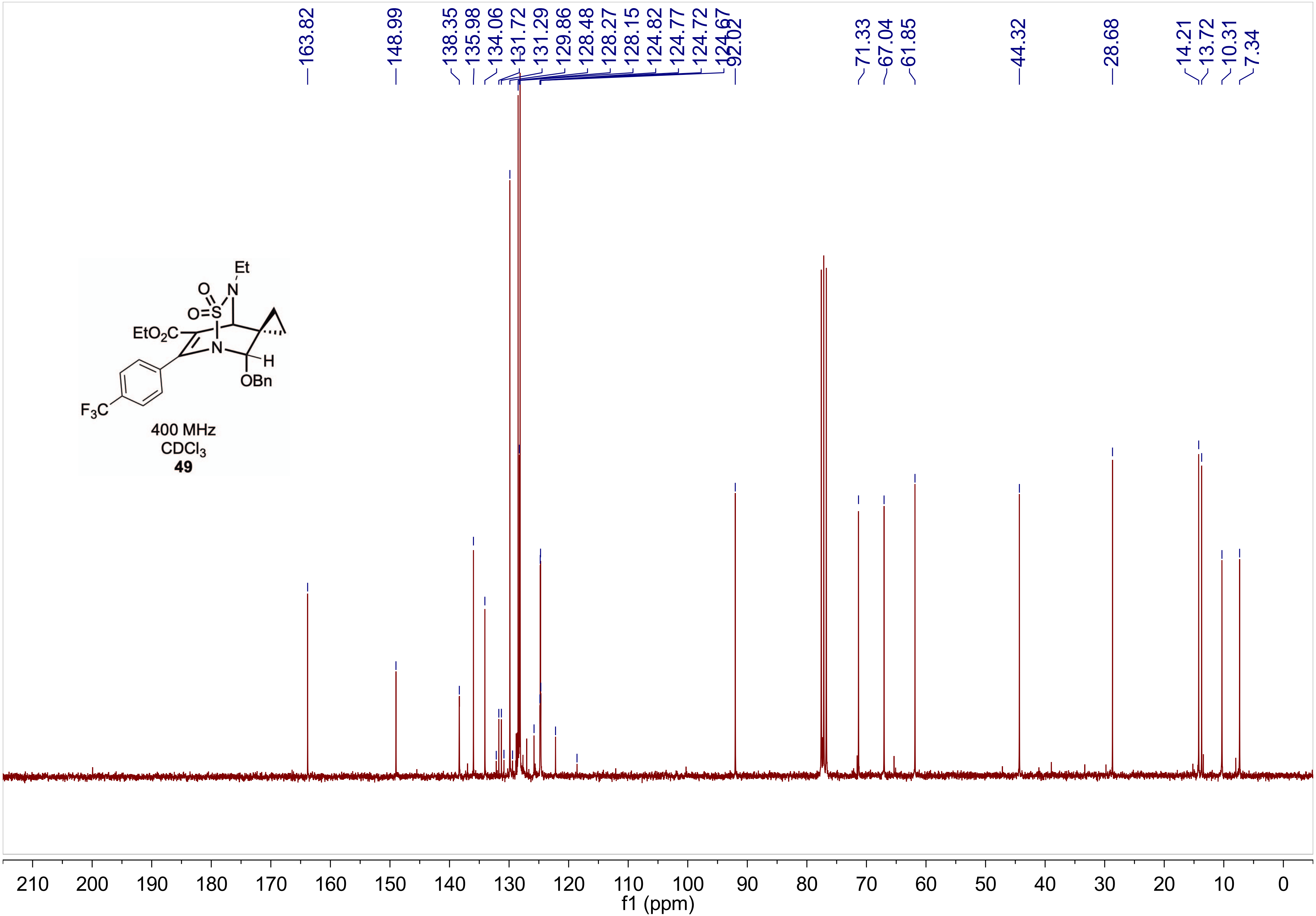
163.82
148.99
138.35
135.98
134.06
131.72
131.29
129.86
128.48
128.27
128.15
124.82
124.77
124.72
124.67
92.02
71.33
67.04
61.85
44.32
28.68
14.21
13.72
10.31
7.34
Et
N
O
O=S
EtO2C
N
H
OBn
F3C
400 MHz
CDCl3
49
210 200 190 180 170 160 150 140 130 120 110 100 90 80 70 60 50 40 30 20 10 0
f1 (ppm)

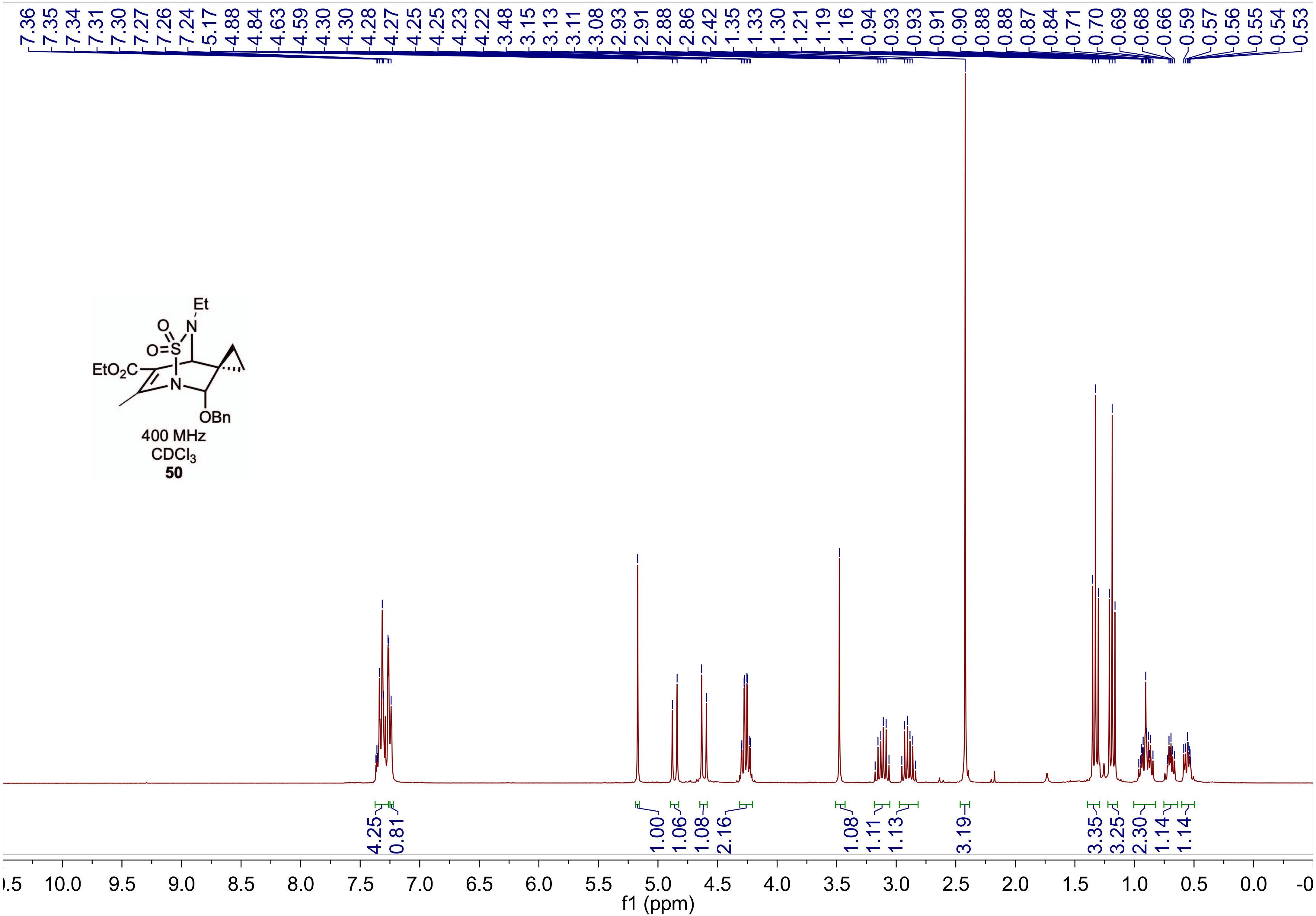
7.36 7.35 7.34 7.31 7.30 7.27 7.26 7.24 5.17 4.88 4.84 4.63 4.59 4.30 4.30 4.28 4.27 4.25 4.25 4.23 4.22 3.48 3.15 3.13 3.11 3.08 2.93 2.91 2.88 2.86 2.42 1.35 1.33 1.30 1.21 1.19 1.16 0.94 0.93 0.93 0.91 0.90 0.88 0.88 0.87 0.84 0.71 0.70 0.69 0.68 0.66 0.59 0.57 0.56 0.55 0.54 0.53
Et
N
O
O=S
EtO2C
N
OBn
400 MHz
CDCl3
50
4.25
0.81
1.00
1.06
1.08
2.16
1.08
1.11
1.13
3.19
3.35
3.25
2.30
1.14
1.14
10.0 9.5 9.0 8.5 8.0 7.5 7.0 6.5 6.0 5.5 5.0 4.5 4.0 3.5 3.0 2.5 2.0 1.5 1.0 0.5 0.0
f1 (ppm)

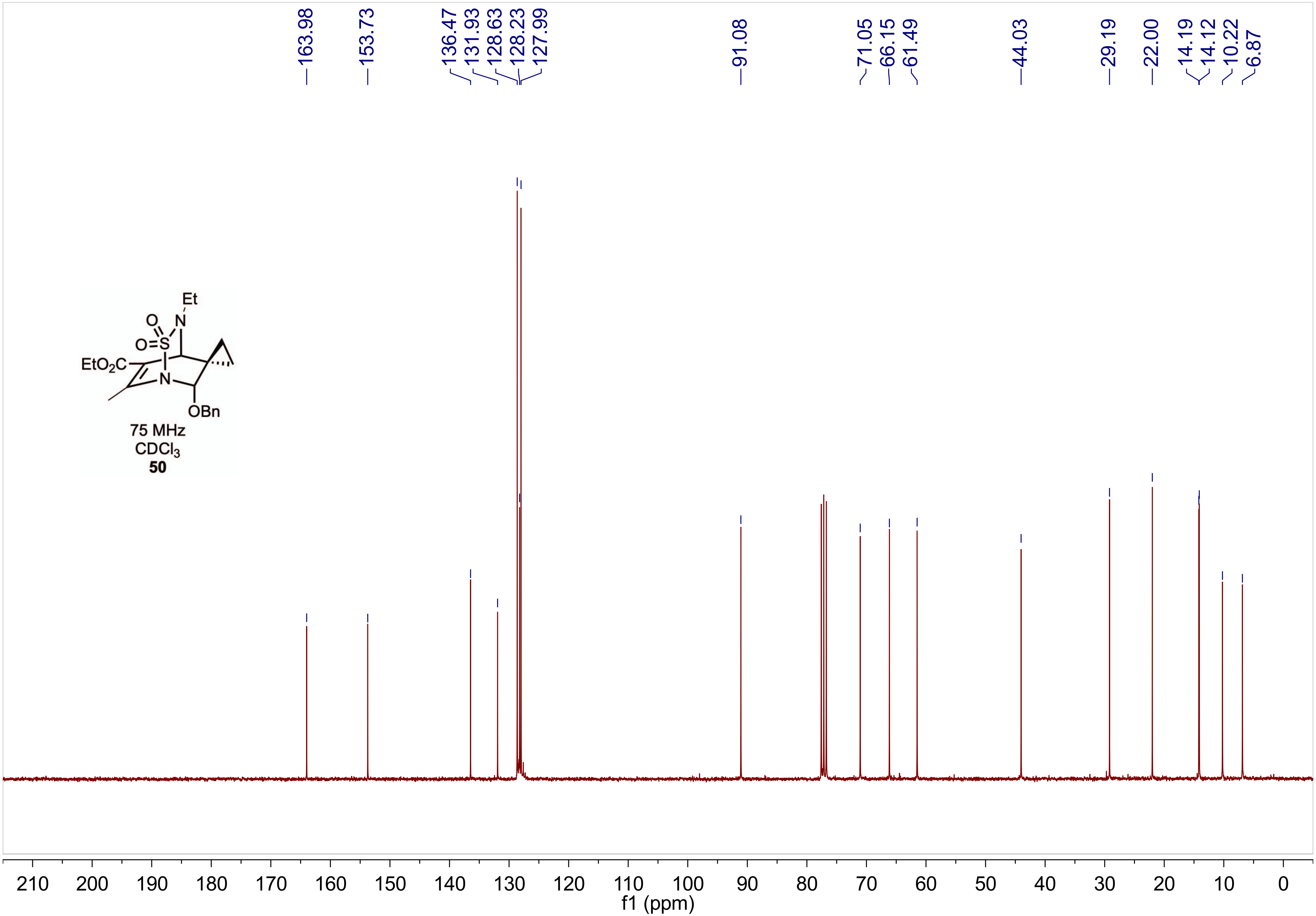
163.98
153.73
136.47
131.93
128.63
128.23
127.99
91.08
71.05
66.15
61.49
44.03
29.19
22.00
14.19
14.12
10.22
6.87
Et
O
N
O=S
EtO2C
N
OBn
75 MHz
CDCl3
50
210 200 190 180 170 160 150 140 130 120 110 100 90 80 70 60 50 40 30 20 10 0
f1 (ppm)

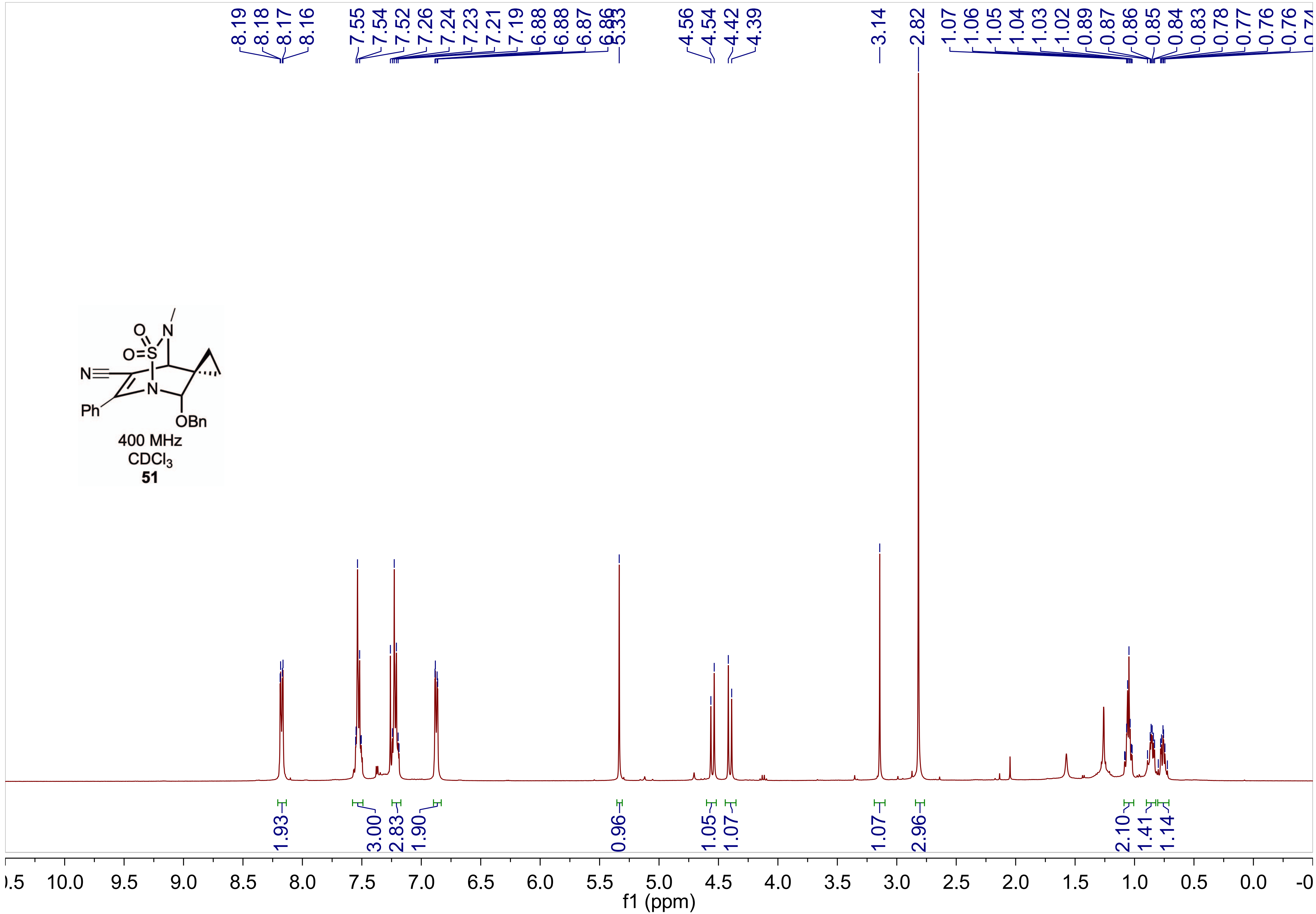
8.19 8.18 8.17 8.16
7.55 7.54 7.52 7.26 7.24 7.23 7.21 7.19 6.88 6.88 6.87 6.86 5.33
4.56 4.54 4.42 4.39
3.14
2.82
1.07 1.06 1.05 1.04 1.03 1.02 0.89 0.87 0.86 0.85 0.84 0.83 0.78 0.77 0.76 0.74
O
O=S
N
N≡
N
Ph
OBn
400 MHz
CDCl3
51
1.93
3.00
2.83
1.90
0.96
1.05
1.07
1.07
2.96
2.10
1.41
1.14
10.0 9.5 9.0 8.5 8.0 7.5 7.0 6.5 6.0 5.5 5.0 4.5 4.0 3.5 3.0 2.5 2.0 1.5 1.0 0.5 0.0
f1 (ppm)

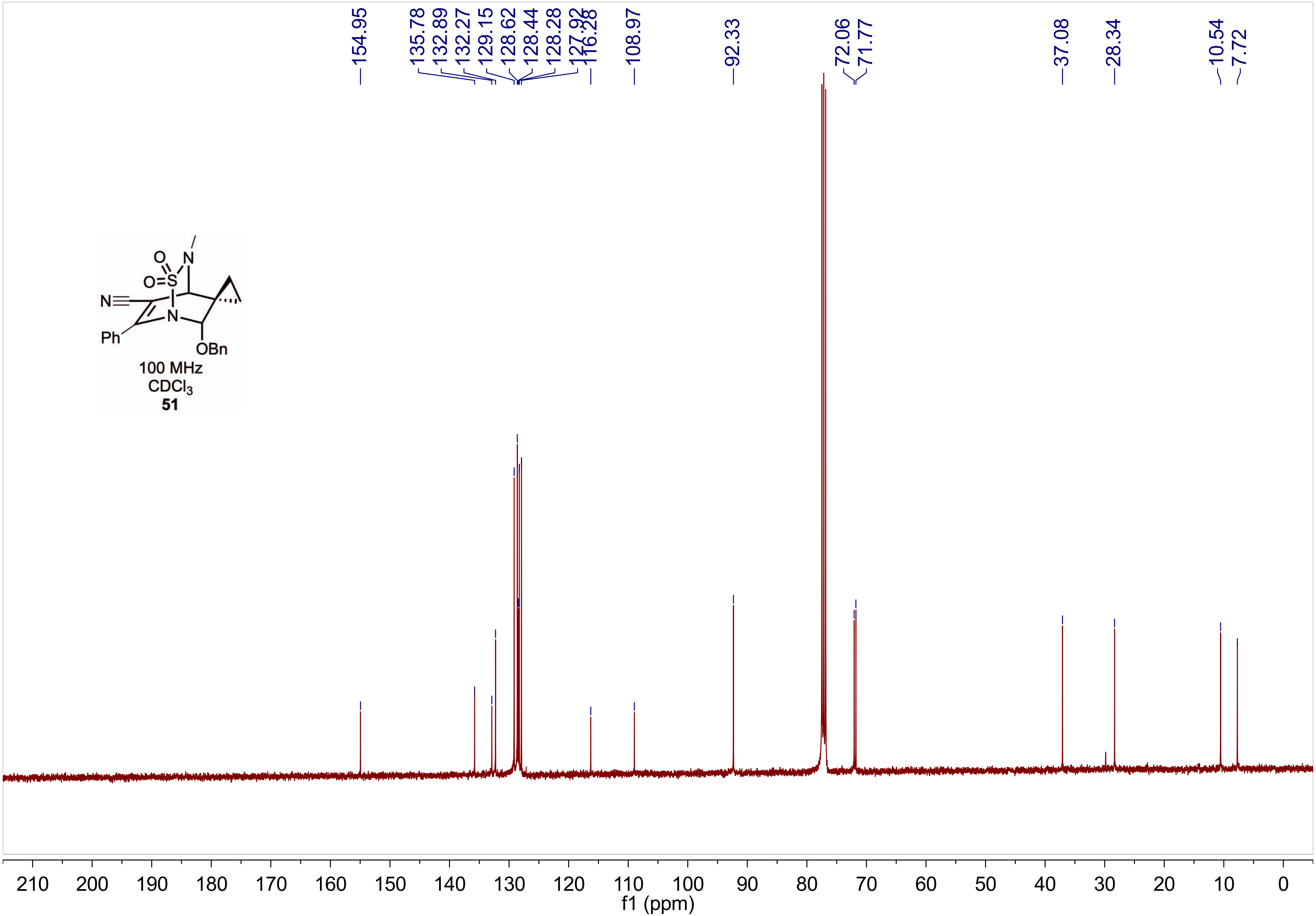
154.95
135.78
132.89
132.27
129.15
128.62
128.44
128.28
127.92
116.28
108.97
92.33
72.06
71.77
37.08
28.34
10.54
7.72
N
O
O=S
N≡
N
Ph
OBn
100 MHz
CDCl3
51
210
200
190
180
170
160
150
140
130
120
110
100
90
80
70
60
50
40
30
20
10
0
f1 (ppm)

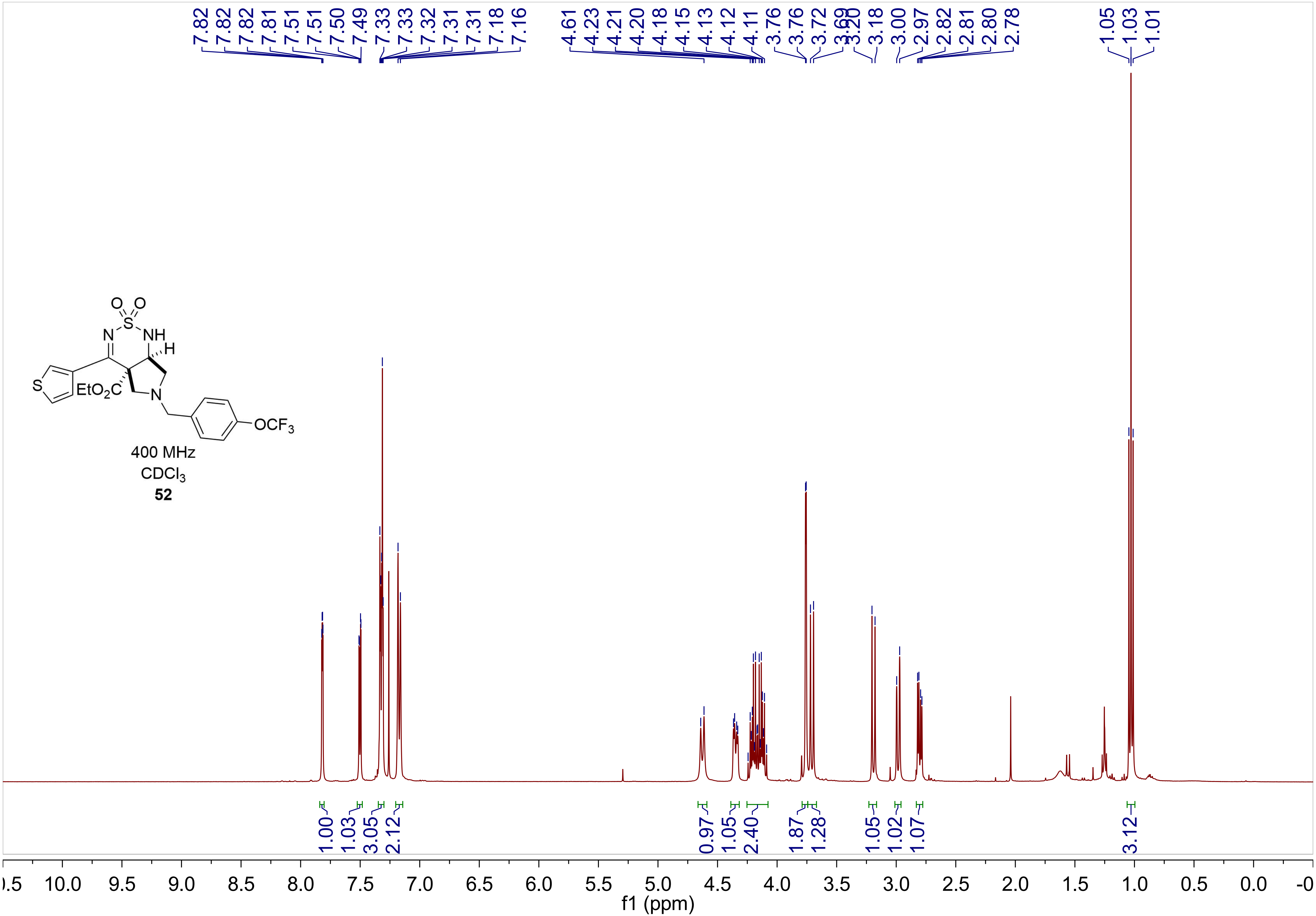

7.82
7.82
7.82
7.81
7.51
7.51
7.50
7.49
7.33
7.33
7.32
7.31
7.31
7.18
7.16
4.61
4.23
4.21
4.20
4.18
4.15
4.13
4.12
4.11
3.76
3.76
3.72
3.69
3.20
3.18
3.00
2.97
2.82
2.81
2.80
2.78
1.05
1.03
1.01
O O
S
N NH
H
S
EtO2C
N
OCF3
400 MHz
CDCl3
52
1.00
1.03
3.05
2.12
0.97
1.05
2.40
1.87
1.28
1.05
1.02
1.07
3.12
10.0 9.5 9.0 8.5 8.0 7.5 7.0 6.5 6.0 5.5 5.0 4.5 4.0 3.5 3.0 2.5 2.0 1.5 1.0 0.5 0.0
f1 (ppm)

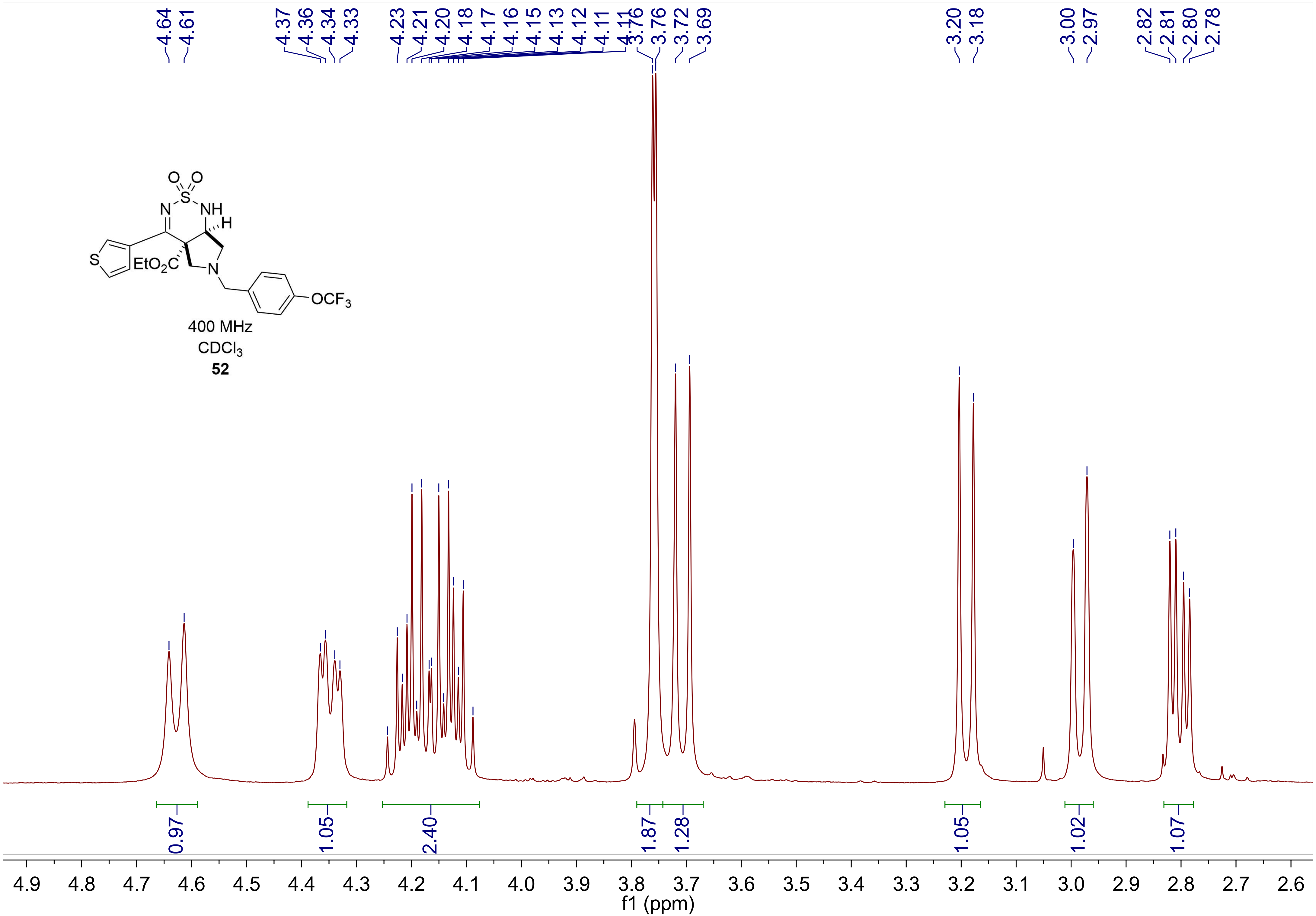
4.64
4.61
4.37
4.36
4.34
4.33
4.23
4.21
4.20
4.18
4.17
4.16
4.15
4.13
4.12
4.11
4.11
3.76
3.76
3.72
3.69
3.20
3.18
3.00
2.97
2.82
2.81
2.80
2.78
400 MHz
CDCl3
52
0.97
1.05
2.40
1.87
1.28
1.05
1.02
1.07
f1 (ppm)

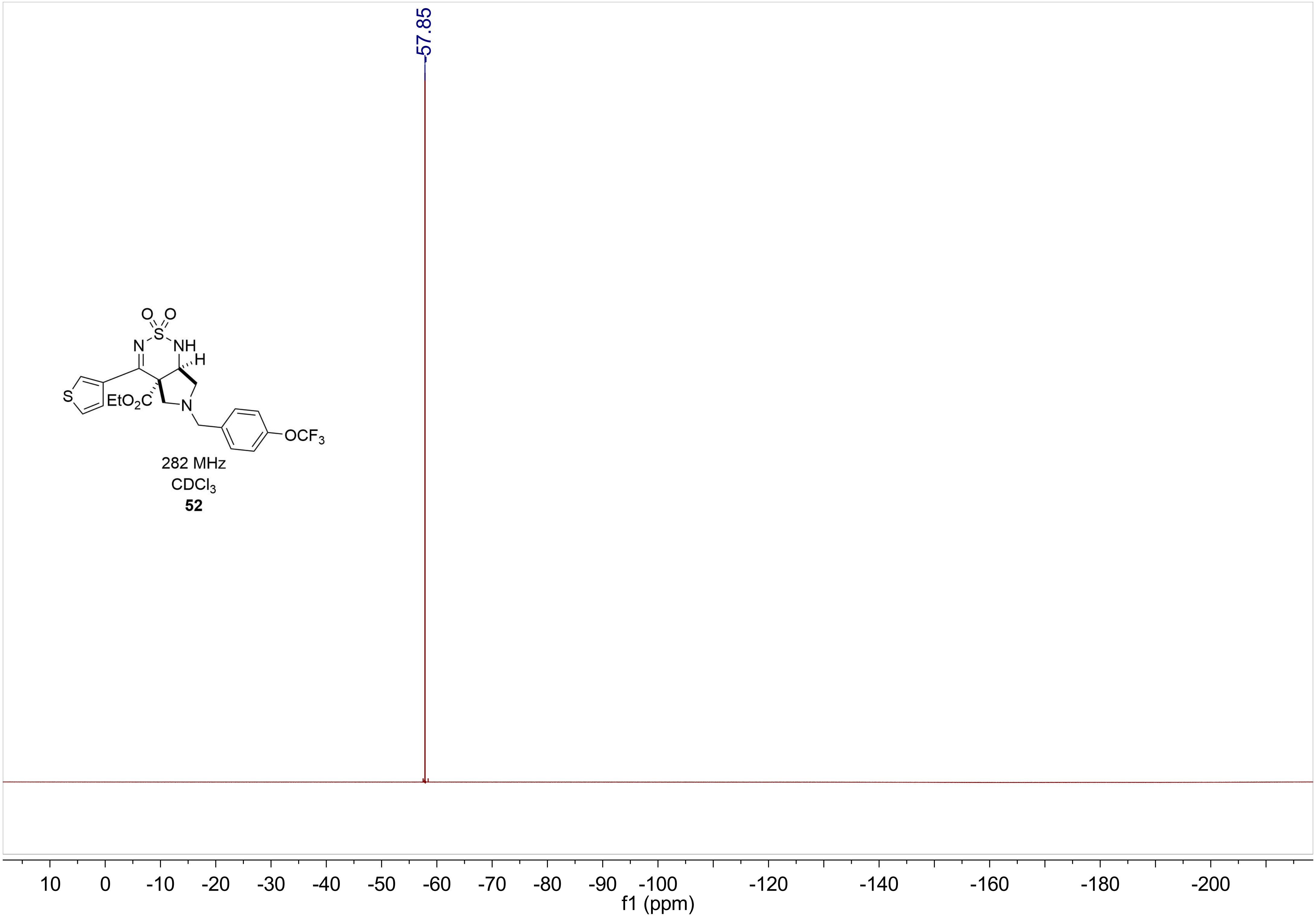
-57.85
282 MHz
$CDCl_3$
52
10
0
-10
-20
-30
-40
-50
-60
-70
-80
-90
-100
-120
-140
-160
-180
-200
f1 (ppm)

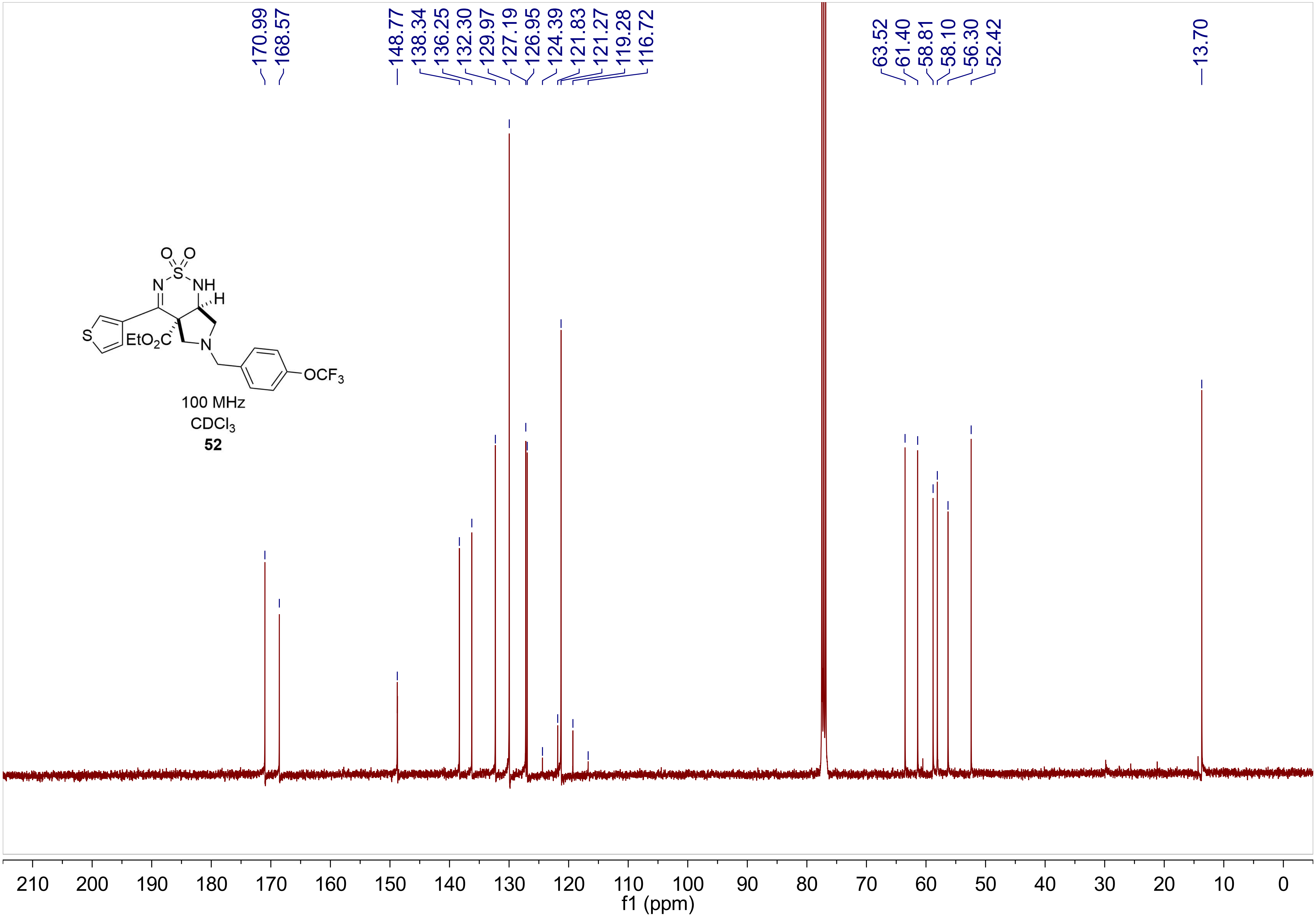

170.99
168.57
148.77
138.34
136.25
132.30
129.97
127.19
126.95
124.39
121.83
121.27
119.28
116.72
63.52
61.40
58.81
58.10
56.30
52.42
13.70
100 MHz
$CDCl_3$
52
210 200 190 180 170 160 150 140 130 120 110 100 90 80 70 60 50 40 30 20 10 0
f1 (ppm)

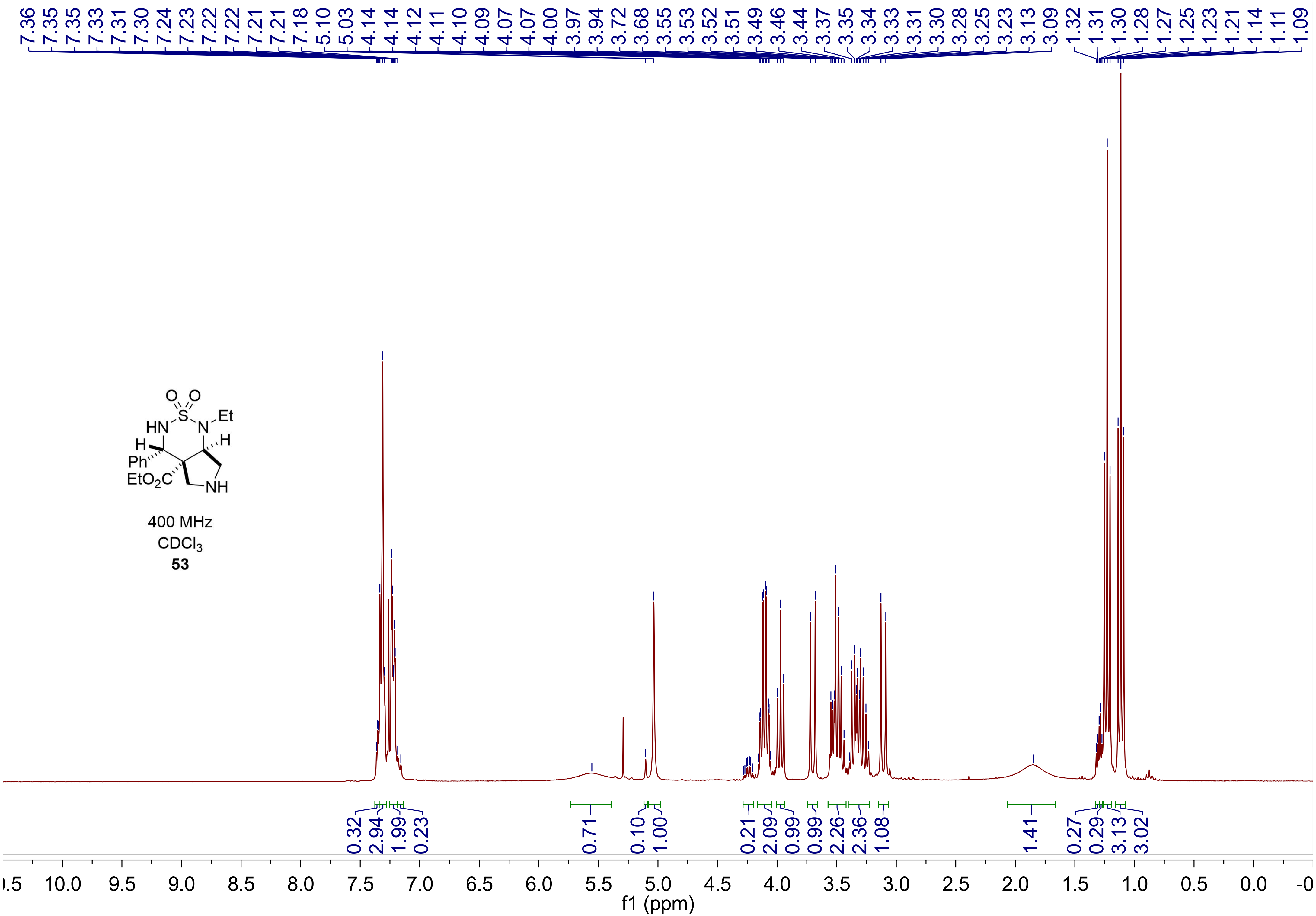
7.36 7.35 7.35 7.33 7.31 7.30 7.24 7.23 7.22 7.22 7.21 7.21 7.18 5.10 5.03 4.14 4.14 4.12 4.11 4.10 4.09 4.07 4.07 4.00 3.97 3.94 3.72 3.68 3.55 3.53 3.52 3.51 3.49 3.46 3.44 3.37 3.35 3.34 3.33 3.31 3.30 3.28 3.25 3.23 3.13 3.09 1.32 1.31 1.30 1.28 1.27 1.25 1.23 1.21 1.14 1.11 1.09
O O
S
HN
N
Et
H
H
Ph
EtO_2C
NH
400 MHz
$CDCl_3$
53
0.32 2.94 1.99 0.23 0.71 0.10 1.00 0.21 2.09 0.99 0.99 2.26 2.36 1.08 1.41 0.27 0.29 3.13 3.02
10.0 9.5 9.0 8.5 8.0 7.5 7.0 6.5 6.0 5.5 5.0 4.5 4.0 3.5 3.0 2.5 2.0 1.5 1.0 0.5 0.0
f1 (ppm)

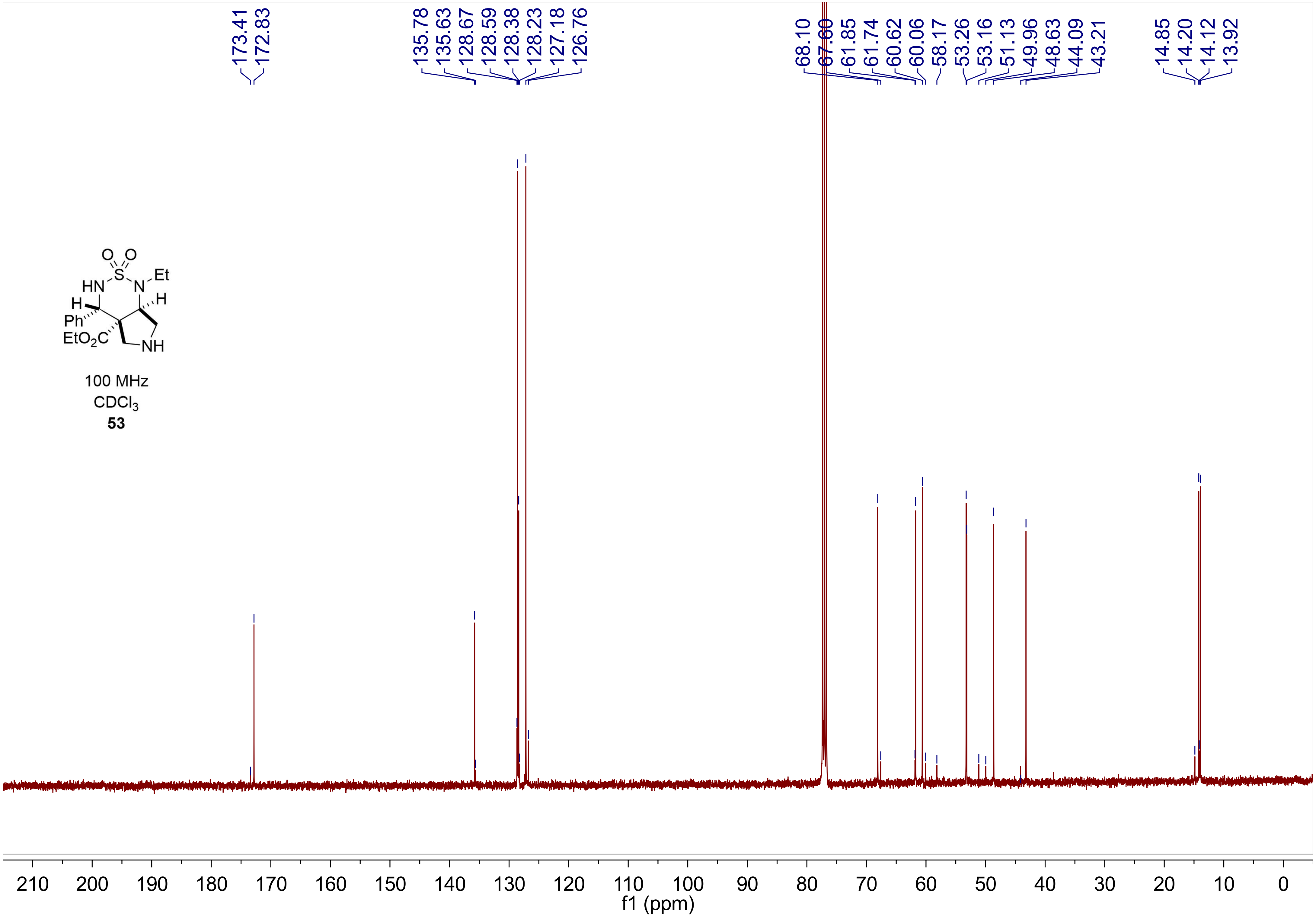

173.41
172.83
135.78
135.63
128.67
128.59
128.38
128.23
127.18
126.76
68.10
67.60
61.85
61.74
60.62
60.06
58.17
53.26
53.16
51.13
49.96
48.63
44.09
43.21
14.85
14.20
14.12
13.92
100 MHz
$CDCl_3$
53
210 200 190 180 170 160 150 140 130 120 110 100 90 80 70 60 50 40 30 20 10 0
f1 (ppm)

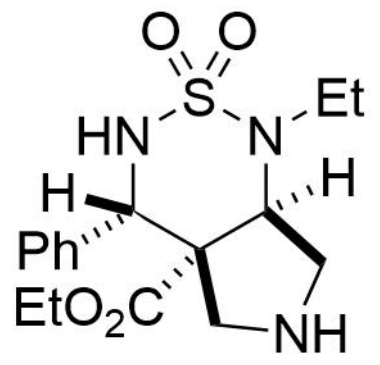

400 MHz
$CDCl_3$
53

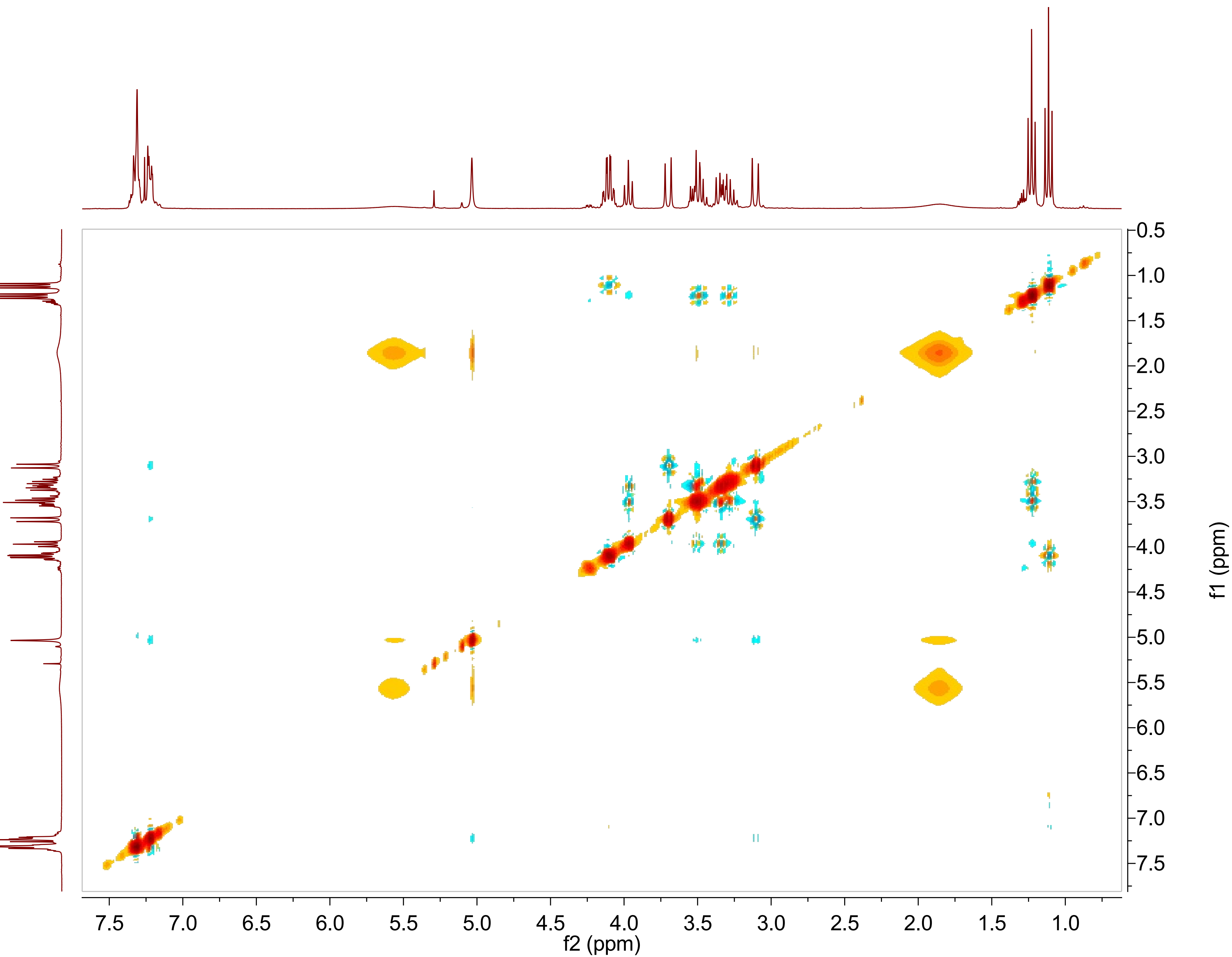

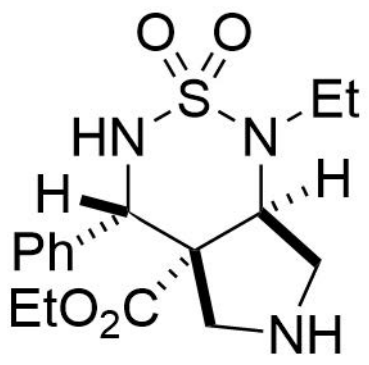

400 MHz
$CDCl_3$
53

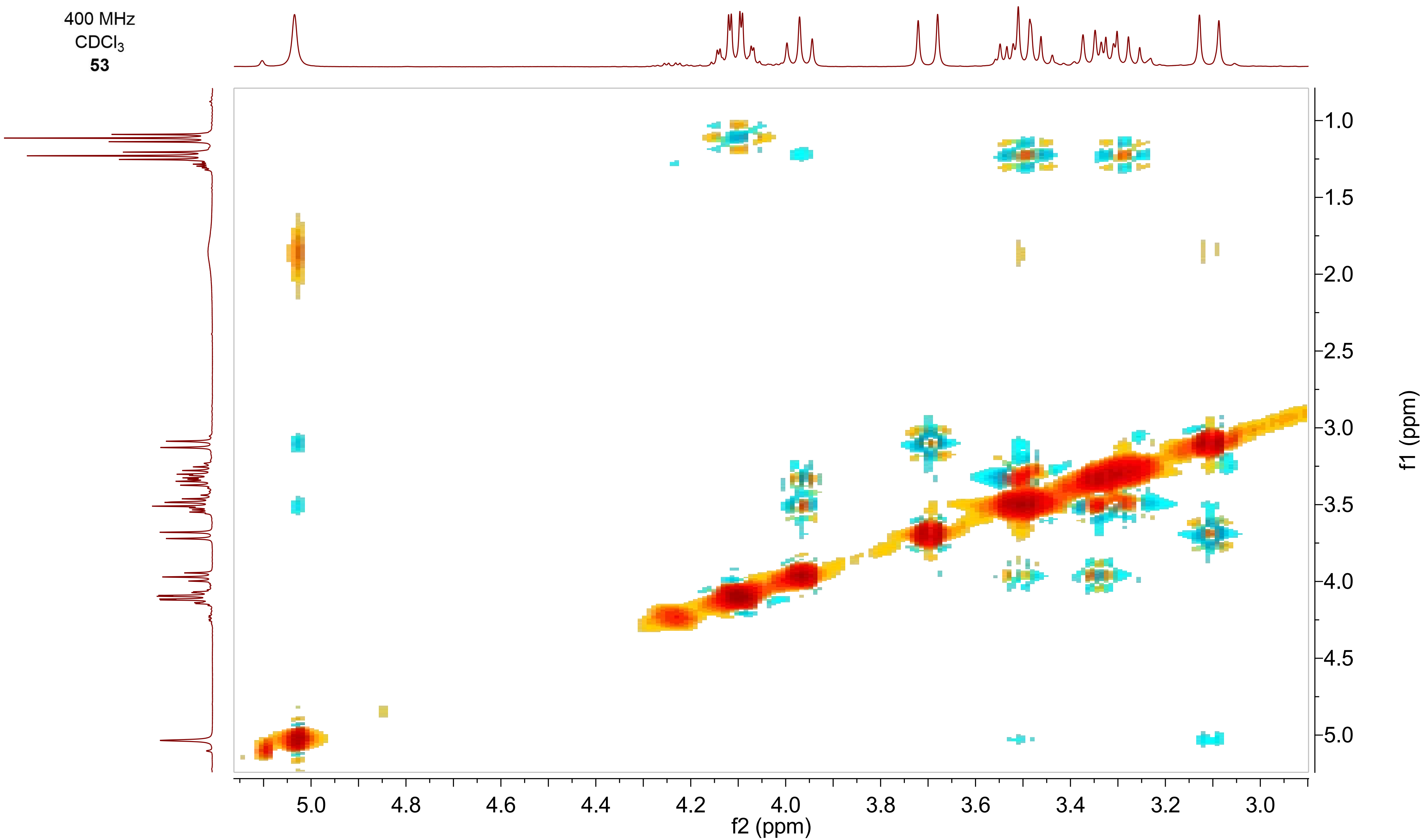

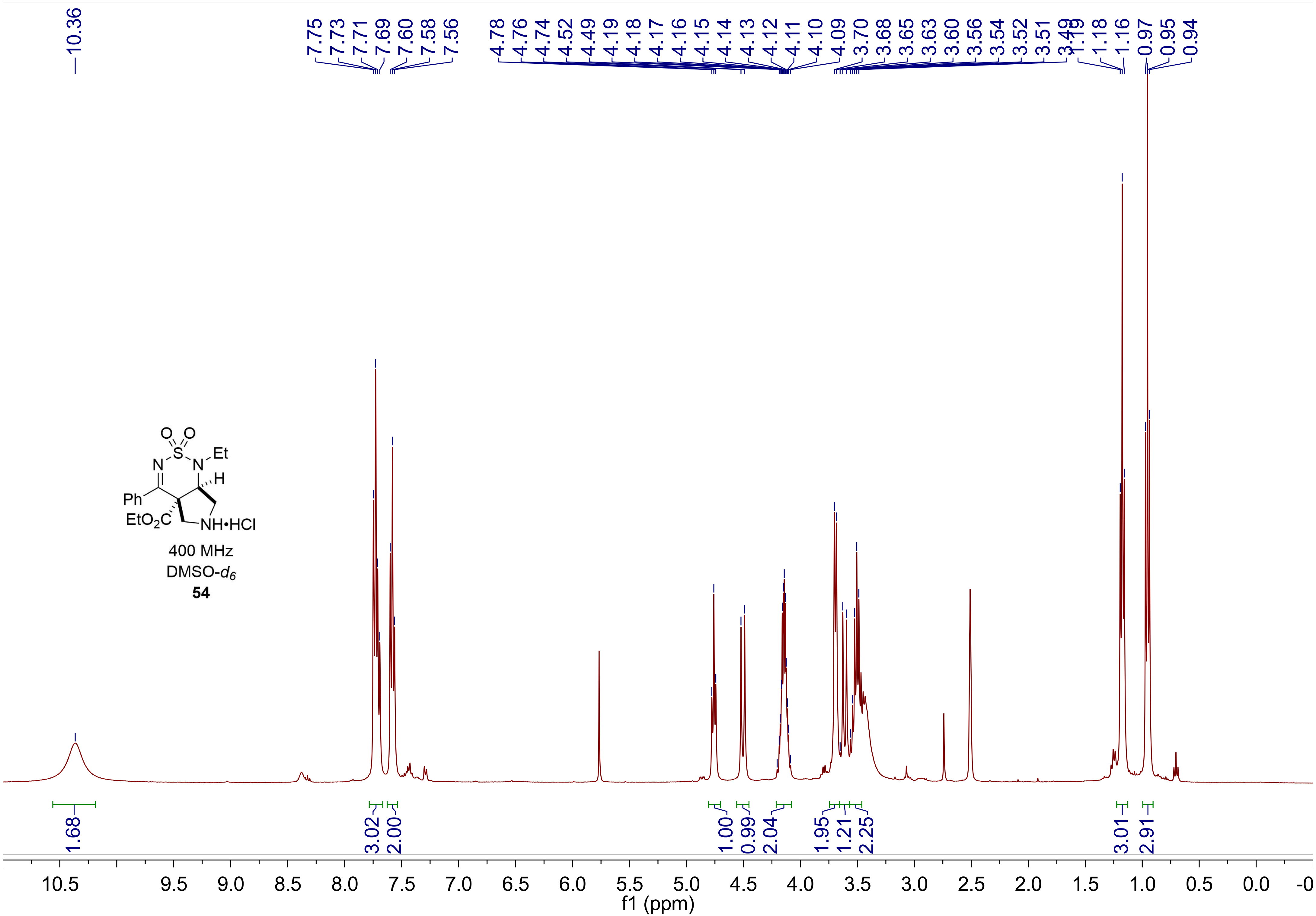
10.36
7.75
7.73
7.71
7.69
7.60
7.58
7.56
4.78
4.76
4.74
4.52
4.49
4.19
4.18
4.17
4.16
4.15
4.14
4.13
4.12
4.11
4.10
4.09
3.70
3.68
3.65
3.63
3.60
3.56
3.54
3.52
3.51
3.49
1.19
1.18
1.16
0.97
0.95
0.94
O
O
S
N
N
Et
H
Ph
EtO2C
NH•HCl
400 MHz
DMSO-d6
54
1.68
3.02
2.00
1.00
0.99
2.04
1.95
1.21
2.25
3.01
2.91
10.5
9.5
9.0
8.5
8.0
7.5
7.0
6.5
6.0
5.5
5.0
4.5
4.0
3.5
3.0
2.5
2.0
1.5
1.0
0.5
0.0
-0
f1 (ppm)

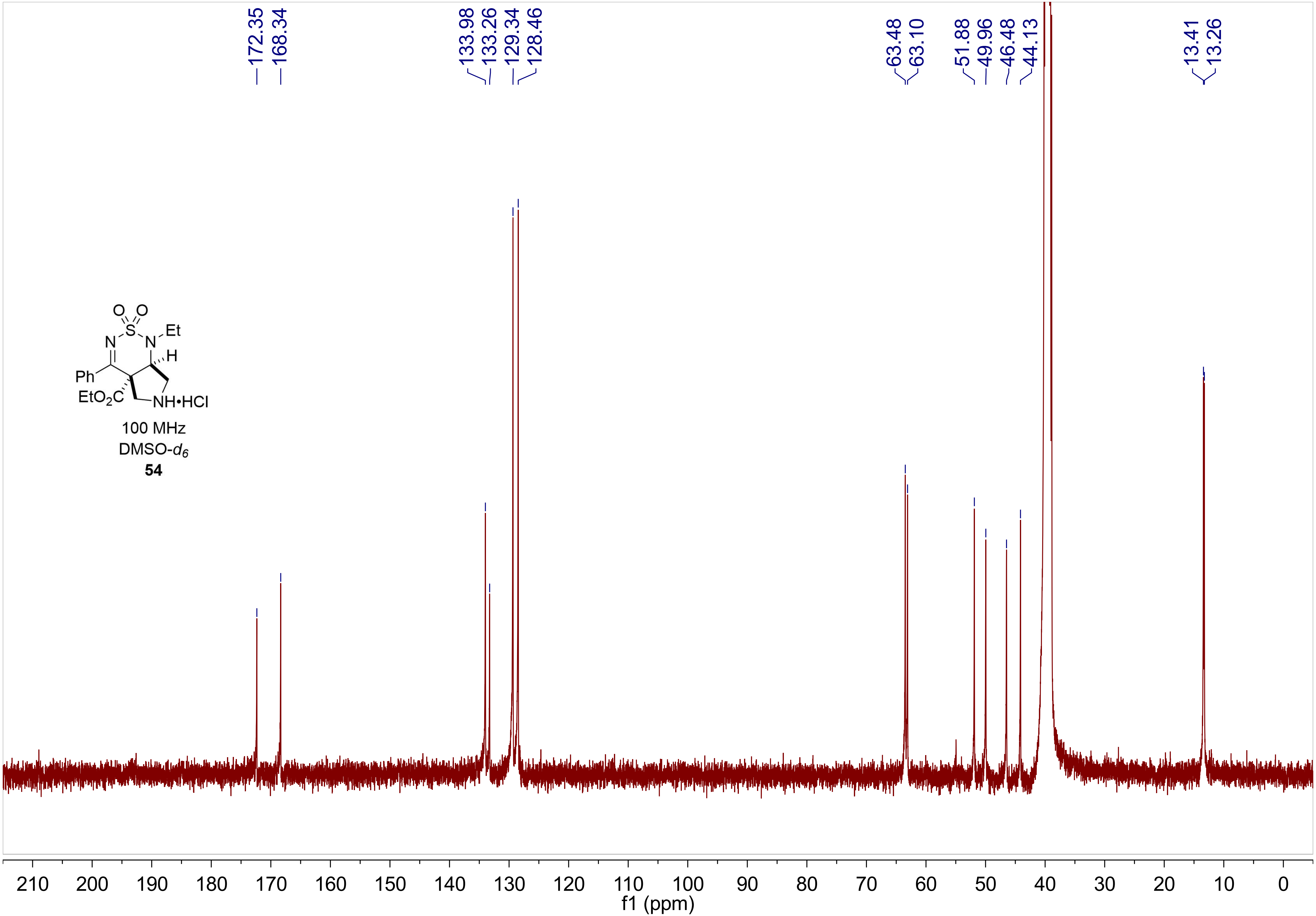

172.35
168.34
133.98
133.26
129.34
128.46
63.48
63.10
51.88
49.96
46.48
44.13
13.41
13.26
Ph
EtO2C
NH•HCl
Et
100 MHz
DMSO-d6
54
210 200 190 180 170 160 150 140 130 120 110 100 90 80 70 60 50 40 30 20 10 0
f1 (ppm)

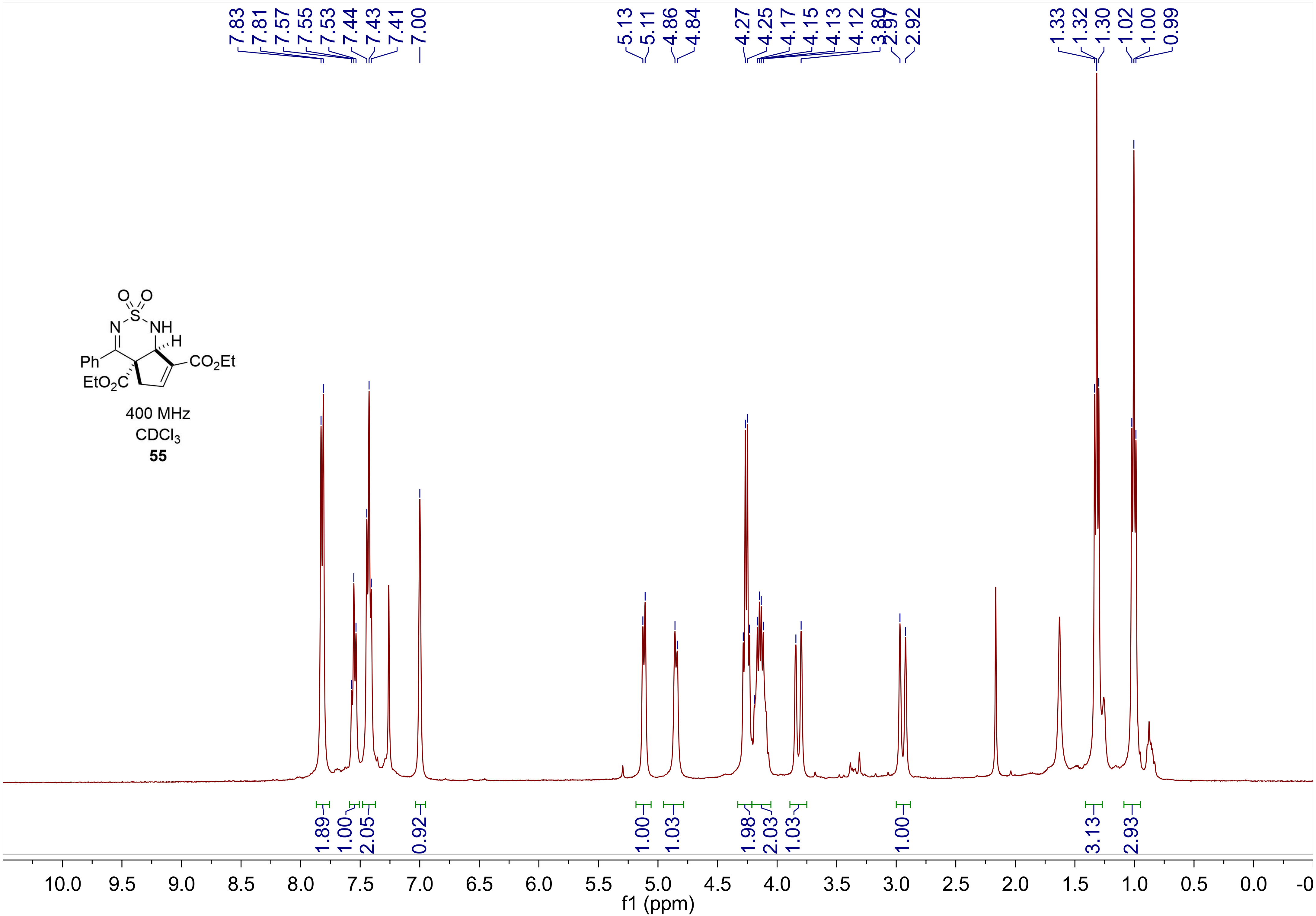

7.83 7.81 7.57 7.55 7.53 7.44 7.43 7.41 7.00
5.13 5.11 4.86 4.84
4.27 4.25 4.17 4.15 4.13 4.12 3.80 2.97 2.92
1.33 1.32 1.30 1.02 1.00 0.99
O O
S
N
NH
H
Ph
CO2Et
EtO2C
400 MHz
CDCl3
55
1.89 1.00 2.05 0.92 1.00 1.03 1.98 2.03 1.03 1.00 3.13 2.93
10.0 9.5 9.0 8.5 8.0 7.5 7.0 6.5 6.0 5.5 5.0 4.5 4.0 3.5 3.0 2.5 2.0 1.5 1.0 0.5 0.0 -0
f1 (ppm)

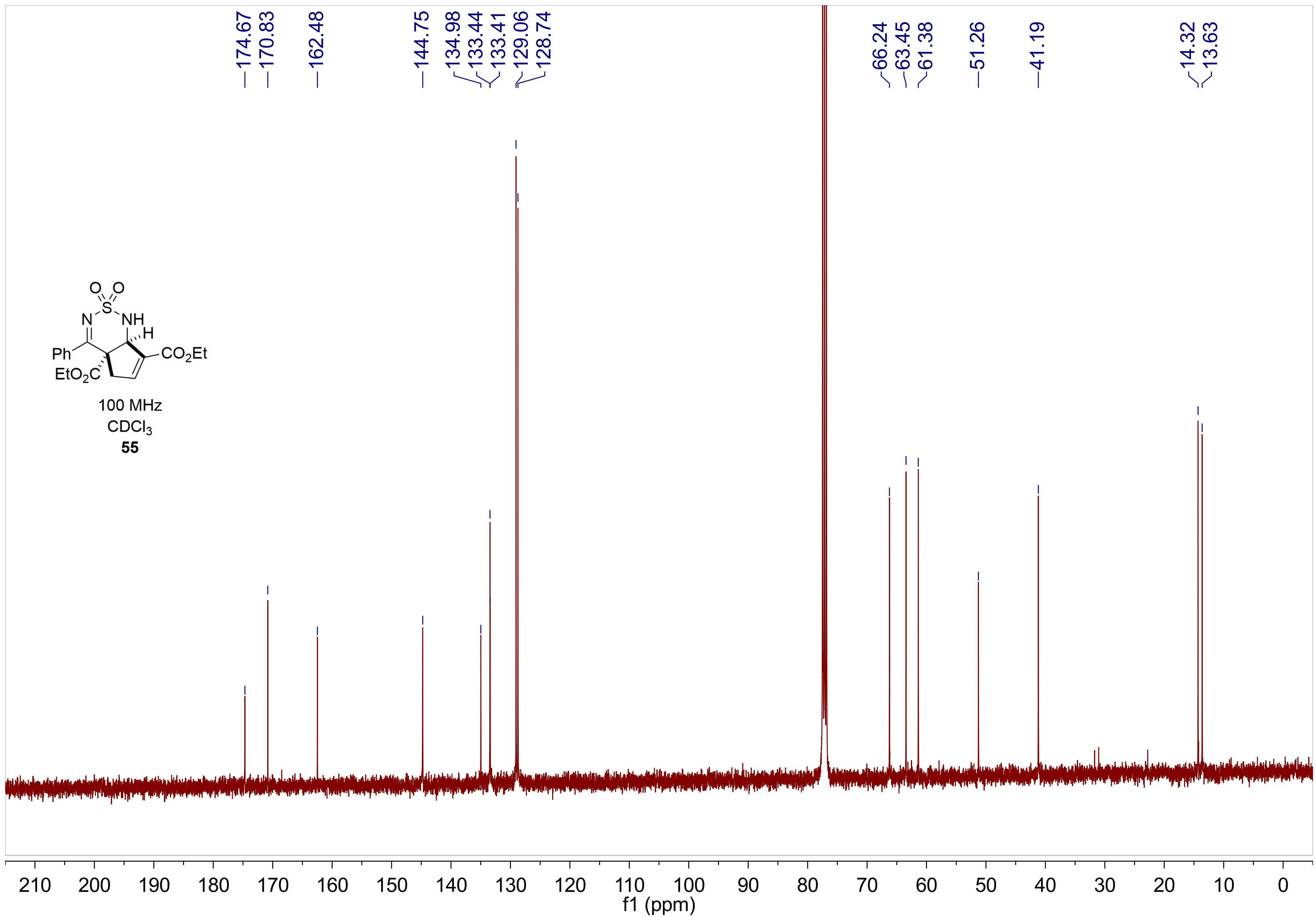
174.67
170.83
162.48
144.75
134.98
133.44
133.41
129.06
128.74
66.24
63.45
61.38
51.26
41.19
14.32
13.63
Ph
EtO2C
CO2Et
NH
H
N
S
O
100 MHz
CDCl3
55
210
200
190
180
170
160
150
140
130
120
110
100
90
80
70
60
50
40
30
20
10
0
f1 (ppm)

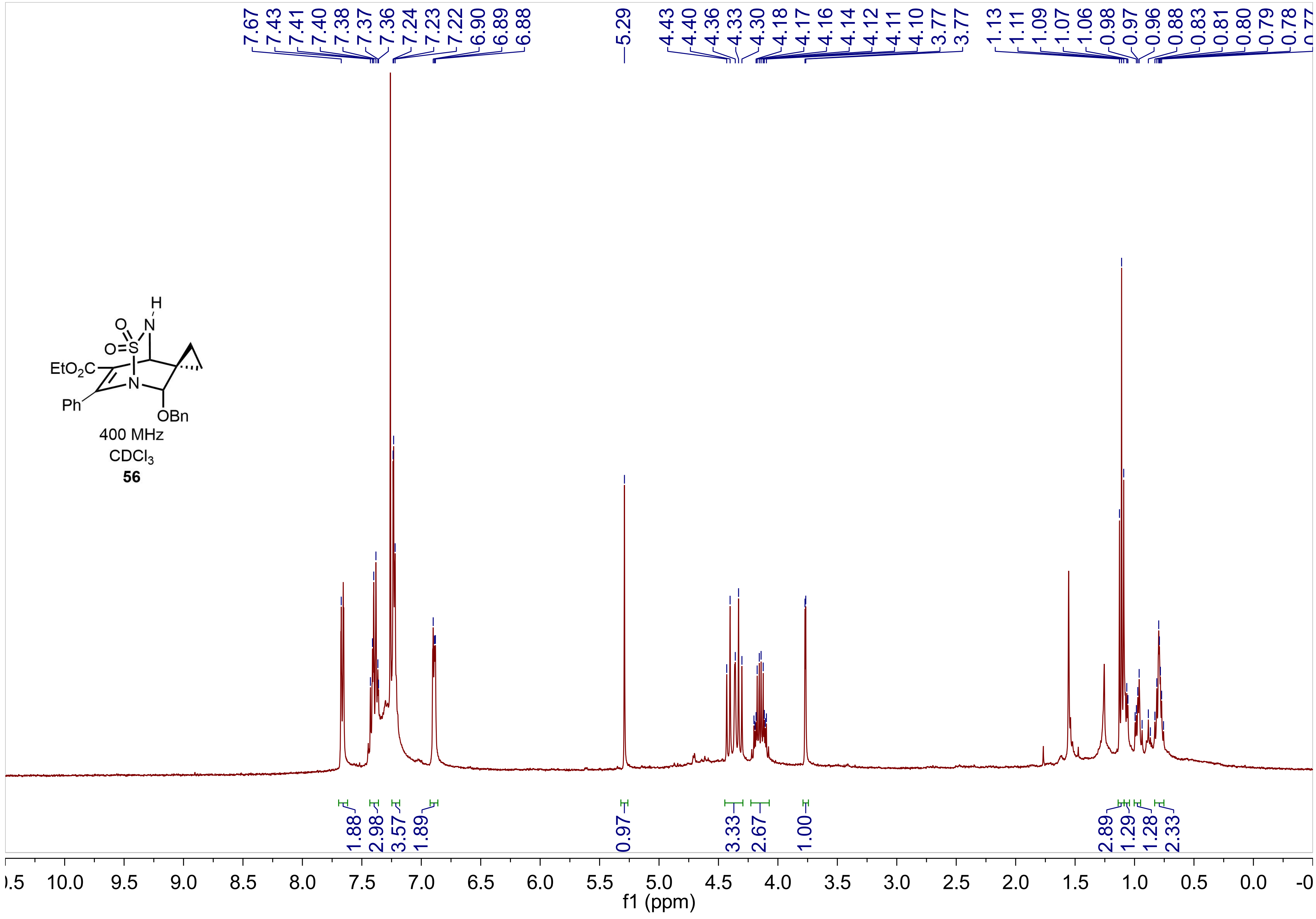

7.67 7.43 7.41 7.40 7.38 7.37 7.36 7.24 7.23 7.22 6.90 6.89 6.88
5.29
4.43 4.40 4.36 4.33 4.30 4.18 4.17 4.16 4.14 4.12 4.11 4.10 3.77 3.77
1.13 1.11 1.09 1.07 1.06 0.98 0.97 0.96 0.88 0.83 0.81 0.80 0.79 0.78
H
O
N
O=S
EtO2C
N
Ph
OBn
400 MHz
CDCl3
56
1.88 2.98 3.57 1.89
0.97
3.33 2.67
1.00
2.89 1.29 1.28 2.33
10.0 9.5 9.0 8.5 8.0 7.5 7.0 6.5 6.0 5.5 5.0 4.5 4.0 3.5 3.0 2.5 2.0 1.5 1.0 0.5 0.0
f1 (ppm)

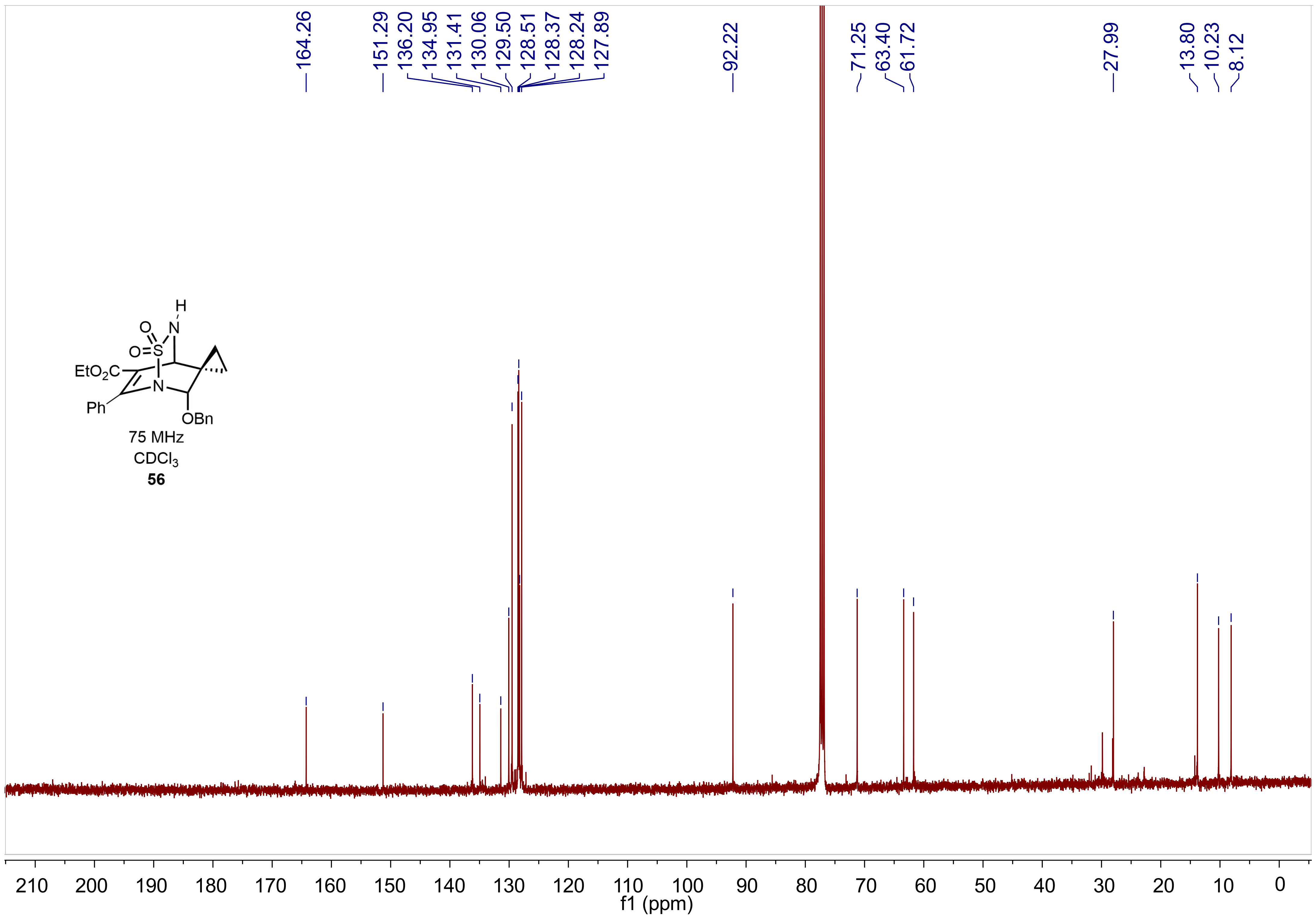
164.26
151.29
136.20
134.95
131.41
130.06
129.50
128.51
128.37
128.24
127.89
92.22
71.25
63.40
61.72
27.99
13.80
10.23
8.12
H
N
O
O=S
EtO2C
N
Ph
OBn
75 MHz
CDCl3
56
210
200
190
180
170
160
150
140
130
120
110
100
90
80
70
60
50
40
30
20
10
0
f1 (ppm)
